# Safety in Numbers: Successful Student-Approved Case-Based Interprofessional Safety Workshop Utilizing Simulated Real-Life Safety Cases

**DOI:** 10.15766/mep_2374-8265.10874

**Published:** 2020-01-31

**Authors:** Lindsay C. Strowd, Erich Grant, Brian Peacock, Kathryn Callahan

**Affiliations:** 1 Assistant Professor, Department of Dermatology, Wake Forest School of Medicine; 2 Assistant Professor, Department of Physician Assistant Studies, Wake Forest School of Medicine; 3 Associate Professor, Department of Internal Medicine, Section on Gerontology, Wake Forest School of Medicine

**Keywords:** Quality Improvement, Interprofessional, Case-Based Learning, Patient Safety

## Abstract

**Introduction:**

Increasing emphasis on medical trainee competence in patient safety and quality improvement processes has led to development of various safety and quality curricula.

**Methods:**

Curriculum surveys indicated our medical school's module-based safety and quality improvement curriculum did not meet student satisfaction benchmarks. We developed a single-day interprofessional patient safety workshop combining students from three different health care training programs (medical doctor, physician assistant, nurse anesthetist). Clinical facilitators from each profession were paired with institutional safety and quality officers. A novel curriculum was created based on a real patient safety case: Students were charged with conducting key interviews of those involved in the event as a root cause analysis (RCA) and developing a process improvement plan based on their RCA findings to present to a panel of institutional executives. Pre- and postevent surveys were completed and analyzed by trainee program.

**Results:**

This workshop improved students’ attitudes regarding interprofessional education and expanded their knowledge of investigating safety events. Overall, assessed knowledge and attitudes improved 53% over the previous safety curriculum. Eighty-one percent of students agreed or strongly agreed that the workshop helped them think about the health care system in a new and different way. One corrective action suggested by a student group during the executive panel was later adopted by our institution and resulted in a permanent change to our health care system.

**Discussion:**

This case-based interprofessional workshop was well received by students and facilitators and allowed purposeful interactions between students in different arenas of medical training.

## Educational Objectives

By the end of this session, learners will be able to:

1. Identify the strengths of an interprofessional health care delivery model.
2. Describe opportunities to improve quality of care within the modern health care system.
3. Apply systems knowledge to perform a root cause analysis on a real safety event.
4. Describe the impact of continual quality improvement on their health care system.
5. Engage current clinical faculty in the process of teaching safety and quality principles.

## Introduction

The 1999 Institute of Medicine report *To Err Is Human: Building a Safer Health System*^1^ highlighted the morbidity and mortality from medical errors that occur within the health care system. The disciplines of patient safety and performance improvement address human factors and systems breakdowns to reduce medical errors and improve outcomes and experiences for patients and their care partners. Human factors focus on environmental and organizational aspects that influence how health care workers interact with health care systems, as well as individual characteristics that influence behavior and health care delivery.^2^ System breakdowns include preventable events that are enabled by the health care system, including technology, equipment, processes, protocols, and culture.

Medical schools are increasingly expected to engage with educational reforms to make patient safety education a central tenet of health care education. The Association of American Medical Colleges (AAMC) now requires medical schools to document individual learner competency for key functions of patient safety, identifying this as one of the 13 AAMC Core Entrustable Professional Activities (EPAs). EPA 13 states that graduates should be able to “identify systems failures and contribute to a culture of safety and improvement.”^3^ Medical schools have to identify the best modalities to teach patient safety functions, provide opportunities for skill acquisition, and assess behaviors that support achievement of EPA 13. Incorporation of education modules and learning opportunities into existing curricula design presents challenges, including competing curricula and faculty oversight.

Only a few limited descriptive studies detail how to achieve this goal. Most courses are optional, not integrated, or focus generally on communication and teamwork skills rather than human factors, systems thinking, organizational change, or skills development.^4,5^ By contrast, optimal educational strategies would include training on communication skills, shared decision making, techniques for working well in teams, and situational awareness.^6^ Curricula have been designed and previously published in *MedEdPORTAL* to adhere to prior residency ACGME Clinical Learning Environment Review guidelines.^7^

The purpose of this curricular activity was to engage interprofessional student teams in a simulation of a patient safety event and follow-up root cause analysis (RCA) and practice improvement plan. Through so doing, we used the specific case of a patient who experienced an adverse event to teach generalizable principles of systems thinking, high-reliability organizations, culture of safety, interprofessional teamwork and roles, and support of second victims in a spirit of fostering resiliency. We chose the safety case presented below because although there were potential diagnostic errors involved, the real causes of the event were related to systems-based errors.

## Methods

We constructed a single-day interprofessional workshop to address the preceding objectives, using pre- and postworkshop REDCap survey data to assess impact (Appendix A). A curricular development committee included clinicians, adult education scholars, and content experts from the physician assistant (PA), nurse anesthetist, and MD programs, as well as the institutional performance improvement and patient safety departments. The committee received support staff from the medical school and was purposefully crafted to include all relevant departments to ensure buy-in from the health care system. The committee met monthly for 6 months, then twice monthly for 3 months preceding the workshop. The committee was responsible for construction of the agenda for the workshop, creation of the curricular content, recruitment of faculty facilitators, and delivery of large-group content. Additional student focus groups were conducted with current health professions students and safety and curricular content experts (Kathryn Callahan) to ensure the curriculum would meet the needs of the learners.

The day's agenda was designed with both a morning and an afternoon program (Figure), with both large- and small-group instruction in each half-day session (Appendix B). For the small-group portion of the workshop, second-year PA students, first-year nurse anesthetist students, and rising third-year MD students were distributed across 22 groups of 10–12 students and at least one clinician facilitator to ensure a balance of perspectives and content exposure. Groups were purposefully constructed to include students from all three schools. Safety and quality officers were on hand to float between groups and address safety content questions. In the morning, sessions featured a large-group setting while four to five presenters introduced specific safety concepts such as principles of safety culture and completing an RCA (Appendices N–R). At the end of the morning large-group session, students were presented with a real hospital safety event and tasked to break into their small groups to conduct an RCA to identify systems errors.

**Figure.**
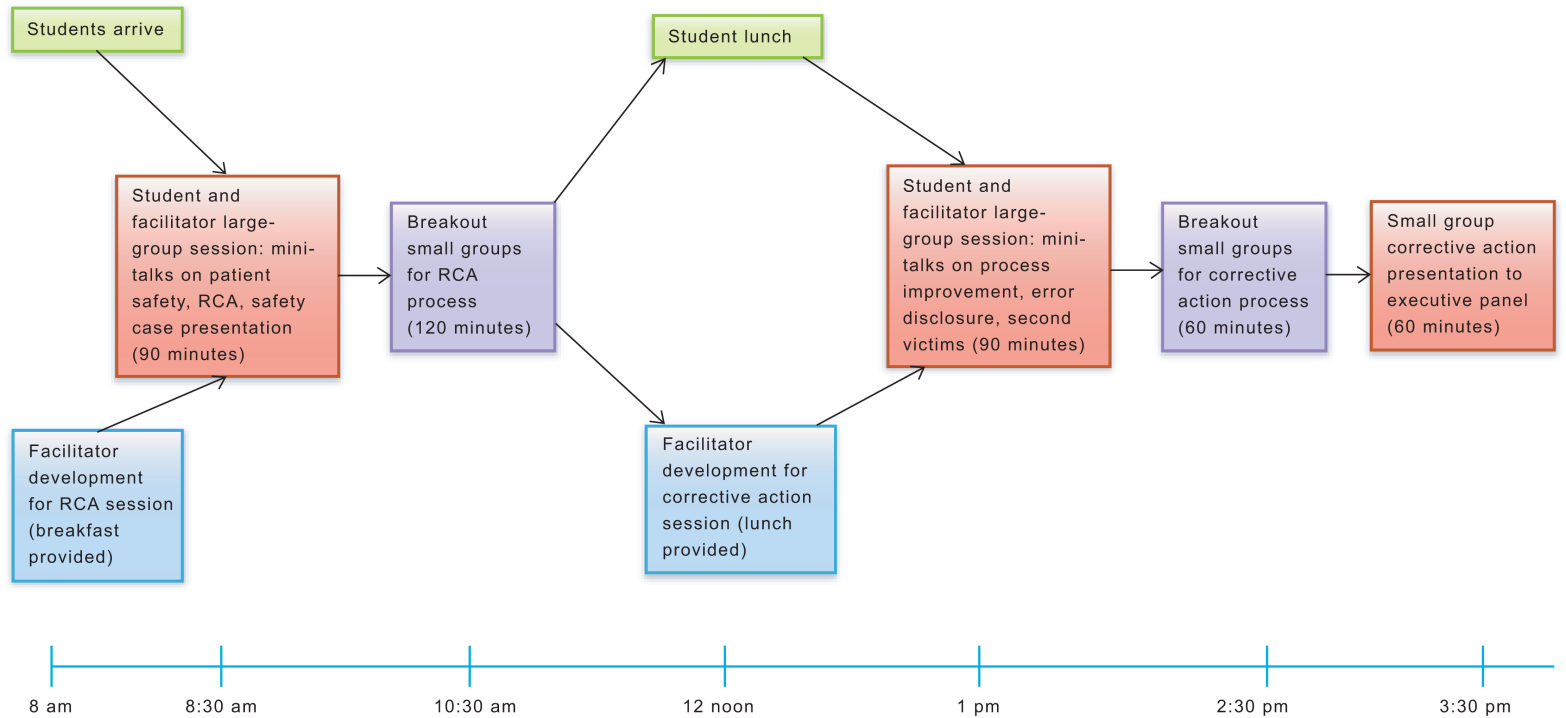
Agenda. Abbreviation: RCA, root cause analysis.

Facilitators were provided with a detailed facilitator guide and annotated case time line prior to the day of the workshop (Appendices C and D). Each small group was given a packet of information regarding the case, which included a student time line of the safety case (Appendix E), transcribed interviews from each health care team member involved in the event (Appendix F), background information on the involved patient and basic information on chronic obstructive pulmonary disease treatment (Appendix G), and two PowerPoint presentations containing media files pertinent to the case, such as images of the radiology suite and oxygen tank storage (Appendices H and I). The students decided who they wanted to interview, and one designated student read aloud the corresponding transcript. Students also had the option to view the media file PowerPoint presentations at any time during the session. Students worked together to flesh out their student time line by going through these interviews and additional files, guided by their facilitator, who had the annotated and complete case time line. By the end of the morning, student groups had identified three to four system errors.

The afternoon session began with the large group being presented with the concepts of error disclosure, second victim experiences, and quality improvement (Appendices N, S–U). The students were tasked to reconvene in their same small groups and develop at least one corrective action based on the identified safety errors of the case (Appendix L). Small-group facilitators received faculty development regarding this afternoon small-group session during lunch and were provided with a detailed afternoon facilitator guide (Appendix J) and a document describing different types of corrective actions (Appendix K). The small groups then reassembled into a large group for the final portion of the workshop, where each small group presented its corrective action to a panel of executive sponsors who were able to give real-time feedback on the feasibility and challenges of implementing corrective actions at an institutional level. Executive sponsors were given a brief summary of the case and real-life corrective actions prior to the workshop (Appendix M). We had surveyed the students via REDCap to assess baseline safety and quality knowledge, as well as attitudes regarding interprofessional education and team-based health care delivery, prior to the workshop (Appendix A). Following the event, we again surveyed the students’ conceptual knowledge and attitudes and garnered feedback regarding student satisfaction with the event (Appendix A). We also surveyed facilitators after the event to determine their enthusiasm for the event (Appendix V). The data were analyzed in aggregate and by training discipline.

Recruited facilitators were provided with a morning facilitator guide (Appendices C and D) and day's agenda (Appendix B), as well as the executive summary of the case (Appendix M), prior to the workshop event. Facilitators were also recommended to review Institute for Healthcare Improvement Module PS 201: Root Cause Analyses and Actions,^8^ which describes basic principles of patient safety and quality to ensure content knowledge. Faculty development for the RCA small-group exercise occurred in the morning over breakfast, prior to the morning large-group session, and facilitators were provided with the afternoon corrective action small-group exercise resources over lunch (Appendices J and K). We emphasized the learning objectives, identified likely pitfalls students could make during the process, and entertained facilitator questions during this time. Some of the pitfalls discussed with facilitators included student propensity to focus on the medical facts of the case instead of the system issues and to place blame on an individual involved in the case instead of the safety gaps in the health care system.

Facilitators were recruited from the participating medical programs, with approximately 60% of the facilitators being physicians, 20% PA faculty, and 20% certified registered nurse anesthetist (CRNA) faculty. Fewer PA and CRNA faculty were used due to the smaller number of their overall faculty. Each clinical facilitator had a patient safety office assigned to help answer safety questions and guide discussion. The executive leaders for the final session of the day included our chief medical officer, chief nursing officer, and chief executive officer, as well as the vice president for quality improvement.

### Equipment/Environment

• Single large-space room to accommodate 200–270 students and facilitators for large-group sessions.
• Twenty small-group classrooms to accommodate 10–12 students and two facilitators.
• Audiovisual equipment for PowerPoint presentations in a large room.

### Materials

• Facilitator guide.
• Interview transcripts for each small-group room.
• Media files for each small-group room.
• Executive summary of case for the executive sponsors.
• Small-group rooms outfitted with a single laptop and whiteboard or paper easel and markers.
• Breakfast for workshop facilitators (optional).
• Boxed lunches for students and facilitators (optional).

### Surveys

• Preworkshop survey on safety and quality knowledge, attitudes toward interprofessional education, and safety culture.
• Postworkshop survey on safety and quality knowledge, attitudes toward interprofessional education, and safety culture.
• Postworkshop survey on event evaluation.
• Facilitator survey on event evaluation.

### Personnel

• Seven to nine presenters for large-group PowerPoint presentations.
• Two assistants to provide help with navigating students from room to room.
• One clinical facilitator (MD, PA, or CRNA) for every 10–12 students.
• One safety/quality officer for every 10–12 students.
• Four executive leaders to serve as executive sponsors at the concluding session.

### Assessment

• Students completed a pre- and postworkshop knowledge-based assessment covering safety and quality principles as outlined in the objectives.
• Students also completed a pre- and postworkshop survey assessing attitudes toward interprofessional education and its importance in health care quality.
• Students and facilitators completed a postworkshop survey on the effectiveness of the workshop in meeting its objectives and small-group case-based learning.
• We plan to survey the medical students who participated in this workshop at the end of their clerkship rotations and reassess their safety and quality knowledge in addition to their attitudes regarding interprofessional education.

### Debriefing

• During the lunch hour, student-identified systems errors from the morning session small groups were collected, tabulated, and then presented to the large group at the beginning of the afternoon session.
• Actual corrective actions implemented after the real safety event occurred were discussed with students at the end of the workshop during the executive sponsor panel.
• We assessed facilitator feedback regarding the day's events immediately postworkshop.

## Results

We have conducted this interprofessional safety workshop for the past 3 years with an average of 125 MD students and 88 PA students, expanding to include 24 nurse anesthetist students for the last 2 years. The number of students participating in the event has ranged from 215 to 240 students depending on the year. Pharmacy students have been involved in past years. The workshop allowed the unique opportunity for students of different medical fields to come together in a purposeful way to work through a real safety event, much the way actual RCAs are performed in the hospital setting. The students were at different stages of their training: MD students preparing to begin their third-year clerkships, PA students at the end of their 2-year training program, pharmacy students at the beginning of their training program, and nurse anesthetist students halfway through their 2-year training program. This was intentional to allow students of different programs to contribute to the conversation in different ways.

A total of 240 students (100%) completed preworkshop assessments, and 148 (62%) completed both pre- and postworkshop assessments for the event. The overall likability of this new case-based learning improved 53% over the previous Institute for Healthcare Improvement module-based safety curriculum when comparing survey data from MD students prior to and after workshop implementation. Based on our assessment, the majority of students (>60%) had no prior formal training in patient safety or quality improvement and had not participated in a previous quality improvement project. More than 90% of all students surveyed stated that the workshop presented them with new content. Sixty-six percent of students felt the workshop environment was either very good or excellent at fostering engaged/interactive learning and promoted collaboration. Eighty-one percent of students agreed or strongly agreed that the workshop helped them think about the health care system in a new and different way.

Student knowledge regarding RCA improved postworkshop, with 82% correctly identifying the purpose of an RCA versus 76% preworkshop. Prior to the workshop, the majority of student participants felt interprofessional care improved the quality of patient care. However, after the workshop, 100% of student participants agreed or strongly agreed with this statement. Student attitudes toward the importance of continual quality improvement in their future careers increased postworkshop as well, with 90% feeling quality improvement was essential or very essential to their future careers.

Ninety-two percent of clinical facilitators gained new safety and quality knowledge from their participation in the workshop, 81% felt more aware of institutional safety and quality issues, and 92% were willing to return as facilitators in the future. The executive leaders also greatly enjoyed the activity and opportunity to interact with the students. One of the corrective actions suggested by a student group during the executive panel was later adopted by our institution and resulted in a permanent change to our health care system. This corrective action was the addition of a “trip slip” for transporting patients off the floor, requiring person-person handoff and a checklist to be signed by both the transporter and the nursing staff.

## Discussion

Safety and quality concepts are embedded within our health care environment, and medical learners need to understand principles of these concepts and how they are applied in real-life scenarios. In fact, one of the EPAs outlined by the AAMC directly relates to this: “Identify system failures and contribute to a culture of safety and improvement.”^3^

This interprofessional event was designed not only to teach safety and quality concepts to health profession learners but also to engage them in an active way through use of simulation. Using a recent and real safety event lent authenticity to the exercise and made the workshop more relatable to students. Carefully selecting a case with multiple root causes was important, as it taught students to look for multiple breakdowns in system processes when a safety event occurs. This case was poignant in that a serious and unexpected bad outcome (patient death) occurred from seemingly simple and preventable events. This resonated with students, and some even became emotional when they discovered what had transpired. Utilizing a real safety event also underscored the core concepts of health care delivery complexities and the contribution of the system to patient care, moving these concepts from theoretical to concrete examples. The event achieved its objectives of performing an RCA and recognizing opportunities for corrective actions by allowing 100% of participants to actively contribute to these exercises in intimate small groups and under the direct facilitation of both clinician educators and institutional safety officers.

Although medical decision-making errors likely also contributed to this safety event, our faculty development sessions specifically asked to focus more on looking for system errors, as students tend to emphasize the errors in medical decision making and feel less experienced in looking beyond those errors to other aspects of the health care system that can fail. Feedback from our facilitators after the event supported this notion, and they reported that students initially needed guidance in identifying other breakdowns in the system.

The small-group exercises were successful in part due to the pairing of clinicians with institutional safety and quality officers. Clinical facilitators could provide guidance on clinical operations within the case context, whereas safety and quality officers could provide insight on the systems processes in place to prevent safety events. This way, clinician volunteers did not have to have a detailed working knowledge of safety and quality concepts prior to participating in the workshop; in fact, some facilitators stated that they learned a great deal regarding patient safety and quality improvement from participating. Another success was in the combination of medical learners at different stages of their training. Having the most clinically experienced students be non-MD students (PA and nurse anesthetist students) allowed those students to take a greater leadership role in the exercise.

This safety workshop should be successful at other institutions if there is a planning committee with representation from all participating schools to ensure a collegial workshop environment and identify knowledge gaps prior to the event. Our planning committee was successful in part due to its heterogeneity and buy-in from all participating schools.

Some challenges encountered during the planning and execution of this event involved the organization and logistics of moving 200+ students from large groups to small groups. We found having a representative from the office of medical education helped anticipate challenges with small-group rooms, audiovisual equipment, and other facilities issues. Another challenge was recruiting faculty to commit to a full-day workshop event. We provided facilitators with a small stipend in the form of educational value units or gift cards. Educational value units are used internally in our institution to incentivize faculty to participate in education initiatives. Compensation for this activity would need to be institution specific.

Facilitator feedback indicated that students demonstrated the common fallacy of viewing this as an individual's fault rather than systems failure. This consistent observation speaks to a gap in medical learner understanding of systems-based health care delivery. We have observed a lack of knowledge with regard to medical students' perception of the scope of practice of their PA and nurse anesthetist student colleagues. Based on this feedback, we incorporated faculty development prior to the workshop on how to address such issues.

This event as outlined has several limitations. One limitation is that evaluation of this workshop primarily measured learner perception of concepts and not actual retention of concepts and real-life application of them in the clinical setting. Future events could be planned to reengage these learners at a later time in their training to see if they retain the knowledge and concepts presented in this workshop. Another limitation is although the event combines medical, PA, and nurse anesthetist students, it does not include many other members of the health care team, such as nurses, medical assistants, and technicians. Inclusion of these students would make the simulation more closely approximate the daily health care team.

Other institutions could use this resource with minimal adjustments, as we believe this resource is detailed and comprehensive, and the case could be applicable to many institutions. Other ways to use this resource would be to structure a workshop in a similar manner but to instead use a different safety case from one's own institution. Future iterations of this event could include the expansion of learners to include other disciplines, inclusion of other faculty and staff within the institution such as environmental engineers for expert content delivery, and a recurrent simulation-based program that would allow students to immerse themselves in the content longitudinally.

This workshop provides an opportunity to introduce students to working in interprofessional teams as they perform a mock RCA and corrective action plan for a real inpatient safety event. Students are able to engage in this simulation event as a way to deepen and broaden their understanding of safety and quality concepts and look for systems-based errors instead of relying upon medical decision-making errors as the main cause for a safety event. This event also provides students with a safe environment for beginning to understand the complexities and benefits of working with their health profession colleagues in an interprofessional manner. The detailed guides, scripts, and course structure provided here will enable other institutions to simulate this event.

## Appendices

A. Pre- & Postevent Surveys.docx
B. IPE Safety Workshop Agenda.docx
C. RCA AM Session Facilitator Guide.docx
D. RCA AM Session Facilitator Annotated Case Time Line.docx
E. RCA AM Session Student Case Time Line.docx
F. RCA AM Session Interviewee Scripts.docx
G. RCA AM Session Patient Background & EWS Info.docx
H. RCA AM Session Media - Radiology.docx
I. RCA AM Session Media - Oxygen Tanks.docx
J. Corrective Action PM Session Facilitator Guide.docx
K. Corrective Action PM Session Effectiveness Chart.docx
L. Corrective Action PM Session Worksheet.docx
M. Executive Case Summary.docx
N. Large-Group Lecture Schedule & Topic List.docx
O. PPT 1 - Contributing to a Culture of Safety.pptx
P. PPT 2 - Systems Improvement.pptx
Q. PPT 3 - Impact of Students and Residents on QI.pptx
R. PPT 4 - Presentation of Safety Case.pptx
S. PPT 5 - Disclosing Medical Errors.pptx
T. PPT 6 - Training for Resilience.pptx
U. PPT 7 - Introduction to Improvement Plans.pptx
V. Facilitator Postworkshop Survey.docx All appendices are peer reviewed as integral parts of the Original Publication.
