# Supplementary material for: Safety in Numbers: Successful Student-Approved Case-Based Interprofessional Safety Workshop Utilizing Simulated Real-Life Safety Cases
Source: MedEdPORTAL. 2020 Jan 31;16:10874. doi: 10.15766/mep_2374-8265.10874 (PMC7065299; doi:10.15766/mep_2374-8265.10874)
Supplement: Supplementary file 1 — A. Pre- & Postevent Surveys.docx B. IPE Safety Workshop Agenda.docx C. RCA AM Session Facilitator Guide.docx D. RCA AM Session Facilitator Annotated Case Time Line.docx E. RCA AM Session Student Case Time Line.docx F. RCA AM Session Interviewee Scripts.docx G. RCA AM Session Patient Background & EWS Info.docx H. RCA AM Session Media - Radiology.docx I. RCA AM Session Media - Oxygen Tanks.docx J. Corrective Action PM Session Facilitator Guide.docx K. Corrective Action PM Session Effectiveness Chart.docx L. Corrective Action PM Session Worksheet.docx M. Executive Case Summary.docx N. Large-Group Lecture Schedule & Topic List.docx O. PPT 1 - Contributing to a Culture of Safety.pptx P. PPT 2 - Systems Improvement.pptx Q. PPT 3 - Impact of Students and Residents on QI.pptx R. PPT 4 - Presentation of Safety Case.pptx S. PPT 5 - Disclosing Medical Errors.pptx T. PPT 6 - Training for Resilience.pptx U. PPT 7 - Introduction to Improvement Plans.pptx V. Facilitator Postworkshop Survey.docx [file mep-16-10874-s001.zip › A. Pre- & Postevent Surveys.docx]

**PREWORKSHOP SURVEY**

**As a participant in this study, please answer the questions below. Thank you!**

First Name

Last Name

Ethnicity

Hispanic or Latino NOT Hispanic or Latino Unknown / Not Reported

Race American Indian/Alaska Native

Asian

Native Hawaiian or Other Pacific Islander

Black or African American

White

More Than One Race

Unknown / Not Reported

Gender Female

Male

Other, please specify

Gender

Please indicate your status PA student CRNA student MD student

If you are a PA student, please indicate the number of clinical hours completed prior to program entry

If you are a CRNA student, please indicate the number of years working as a nurse prior to program entry (please round to the first decimal).

If you are an MD student, please indicate the number of hours of clinical experience completed prior to program entry.

**Pretest - Attitudes Toward Healthcare Teams**

1) The interprofessional approach improves the quality of care to patients/clients.

Strongly disagree

Disagree Neither agree nor disagree

Agree Strongly Agree

2) The interprofessional approach permits health professionals to meet the needs of family caregivers as well as patients.

3) Having to report observations to a team helps team members better understand the work of other health professionals.

4) The interprofessional approach makes the delivery of care more efficient

5) Hospital patients who receive interprofessional team care are better prepared for discharge than other patients.

6) Team meetings foster communication among team members from different professions or disciplines.

7) The give and take among team members helps them make better patient/client care decisions.

8) Patients/clients receiving interprofessional care are more likely than others to be treated as whole persons.

9) Health professionals working as teams are more responsive than others to the emotional and financial needs of

patients/clients

10)

Working in an interprofessional environment keeps most health professionals enthusiastic and interested in their jobs.

11) Developing a patient/client care plan with other team members avoids errors in delivering care.

12) Working in an interprofessional manner unnecessarily complicates things most of the time.

Strongly disagree

Disagree Neither agree nor disagree

Agree Strongly Agree

13) In most instances, the time required for interprofessional consultations could be better spent in other ways.

14) Developing an interprofessional patient/client care plan is excessively time-consuming.

**Longitudinal QI Curriculum Attitudinal Questions**

15) Have you previously received any formal training in Yes quality improvement or patient safety (i.e., the No Institute for Healthcare Improvement Open School)?

16) Have you ever participated in a continuous Yes improvement effort? (e.g. personal improvement No project, workplace initiative, or other experience

that used formal improvement principles and methods)?

17) How essential do you consider continuous improvement Very non-essential in your future professional work? Non-essential

Neither essential nor non-essential

Essential

Very essential

18) How confident are you that you can make a change to Not confident at all improve health care in a local setting? Not confident

Reasonably confident

Confident

Very confident

**Quality Improvement and Patient Safety**

19) What are the three categories of measures that should

Outcome, Process, Balancing

be obtained in a quality improvement process? Process, homogeneity, satisfaction

Diversity, transparency, satisfaction

Equity, autonomy, homogeneity

Process, transparency, satisfaction

20) An intern is cross-covering a large number of Slip patients, and receives two pages in quick succession Latent error regarding patients with similar last names. The Active error

intern mixes up the names and accidentally writes an Knowledge-based error order for patient Hayes to get an antibiotic and

patient Hanes to get a sleeping aide, when it should have been the other way around. When the nurse gives patient Hayes an antibiotic that is not needed,

which type of error is this?

21) What are the four steps in the overarching "life Plan, Do, Study, Act

cycle" of a QI project (the whole project, from Innovation, Plan, Pilot, Act beginning to end)? Plan, Pilot, Implementation, Study

Innovation, Pilot, Implementation, Spread

22) Several incidences occur of errors where a medication Simplification with topical and intravenous applications is used Standardization for the wrong indication (that is, injecting the Redundancies topical version or applying the intravenous version. Constraints After these are reported to the pharmaceutical

company, they redesign the packaging so that they look different: the topical preparation now has a label of a different color. This is an example of:

23) What is the underlying purpose of a root cause To discover what produced an event

analysis: To create a process map or fishbone diagram to describe an event

To define what should have happened in an event

Recommendation

To reconstruct what happened before an event

24) When a medical error occurs, which is the best order Communicate with the patient, report the error to of priorities? appropriate parties, check the medical record,

care for the patient.

Report the error to appropriate parties, check the medical record, care for the patient, communicate with the patient.

Care for the patient, communicate with the patient, report the error to appropriate parties, check the medical record.

Check the medical record, care for the patient, communicate with the patient, report the error to appropriate parties.

25) For a hospital to have an effective culture of People know their concerns will be openly received safety, it's important that employees experience and treated with respect.

psychological safety. Which of the following is the Leaders actively create an environment where all best definition of psychological safety (in contrast staff are comfortable expressing their concerns. to fairness, transparency, and active leadership)? Patient safety problems aren't swept under the

rug. Team members have a high degree of confidence that the organization will learn from problems and

use them to improve the system.

People know they will not be punished or blamed for system-based errors.

26) Select the statement that best reflects taking a Saying, "We could solve this if only we had a leadership stance in health care: computer system that could report on this."

Saying, "Medical students shouldn't be blamed for things that aren't our fault."

Saying, "Patients are being hurt by this problem." Saying, "We should find out how big a problem this is. Let's count how often this is an issue on this unit."

27) Which of the following is NOT an example of Avoiding discussion of patient details while in effectively maintaining patient confidentiality? the elevator

Working with a family member as an interpreter if the patient says it's ok

Logging out of the EMR after you are finished using it

Discussing patient care matters with any staff who are responsible for that patient's care

28) Which of the following is part of a PDSA cycle? Evaluate sentinel events

Plan and pilot test data collection

Root cause analysis

Set the agenda and assign responsibility

29) Which of the following statements about increasing Increased quality is always associated with health care quality and the cost of care is true? increased cost

Technology (a cost) is essential to quality improvement

Processes that enhance quality may also decrease cost

Reducing medical errors does not reduce cost

Answers to Content Questions

19. Outcome, Process, Balancing

20. Slip

21. Plan, Pilot, Implementation, Study

22. Standardization

23. To discover what produced an event

24. Care for the patient, communicate with the patient, report the error to appropriate parties, check the medical record.

25. People know they will not be punished or blamed for system-based errors

26. Saying, "We should find out how big a problem this is. Let's count how often this is an issue on this unit."

27. Working with a family member as an interpreter if the patient says it's ok

28. Plan and pilot test data collection

29. Processes that enhance quality may also decrease cost

**POST WORKSHOP SURVEY**

**Attitudes Toward Healthcare Teams**

**Posttest - Attitudes Toward Healthcare Teams**

1) The interprofessional approach improves the quality of care to patients/clients.

Strongly disagree

Disagree Neither agree nor disagree

Agree Strongly Agree

2) The interprofessional approach permits health professionals to meet the needs of family caregivers as well as patients.

3) Having to report observations to a team helps team members better understand the work of other health professionals.

4) The interprofessional approach makes the delivery of care more efficient

5) Hospital patients who receive interprofessional team care are better prepared for discharge than other patients.

6) Team meetings foster communication among team members from different professions or disciplines.

7) The give and take among team members helps them make better patient/client care decisions.

8) Patients/clients receiving interprofessional care are more likely than others to be treated as whole persons.

9) Health professionals working as teams are more responsive than others to the emotional and financial needs of

patients/clients

10)

Working in an interprofessional environment keeps most health professionals enthusiastic and interested in their jobs.

11) Developing a patient/client care plan with other team members avoids errors in delivering care.

12) Working in an interprofessional manner unnecessarily complicates things most of the time.

Strongly disagree

Disagree Neither agree nor disagree

Agree Strongly Agree

13) In most instances, the time required for interprofessional consultations could be better spent in other ways.

14) Developing an interprofessional patient/client care plan is excessively time-consuming.

**Longitudinal QI Curriculum Attitudinal Questions**

15) Have you previously received any formal training in Yes quality improvement or patient safety (i.e., the No Institute for Healthcare Improvement Open School)?

16) Have you ever participated in a continuous Yes improvement effort? (e.g. personal improvement No project, workplace initiative, or other experience

that used formal improvement principles and methods)?

17) How essential do you consider continuous improvement Very non-essential in your future professional work? Non-essential

Neither essential nor non-essential

Essential

Very essential

18) How confident are you that you can make a change to Not confident at all improve health care in a local setting? Not confident

Reasonably confident

Confident

Very confident

**Quality Improvement and Patient Safety**

19) What are the three categories of measures that should Diversity, transparency, satisfaction be obtained in a quality improvement process? Process, homogeneity, satisfaction

Equity, autonomy, homogeneity

Process, transparency, satisfaction

20) An intern is cross-covering a large number of Slip patients, and receives two pages in quick succession Latent error regarding patients with similar last names. The Active error

intern mixes up the names and accidentally writes an Knowledge-based error order for patient Hayes to get an antibiotic and

patient Hanes to get a sleeping aide, when it should have been the other way around. When the nurse gives patient Hayes an antibiotic that is not needed,

which type of error is this?

21) What are the four steps in the overarching "life Plan, Do, Study, Act

cycle" of a QI project (the whole project, from Innovation, Plan, Pilot, Act beginning to end)? Plan, Pilot, Implementation, Study

Innovation, Pilot, Implementation, Spread

22) Several incidences occur of errors where a medication Simplification with topical and intravenous applications is used Standardization for the wrong indication (that is, injecting the Redundancies topical version or applying the intravenous version. Constraints After these are reported to the pharmaceutical

company, they redesign the packaging so that they look different: the topical preparation now has a label of a different color. This is an example of:

23) What is the underlying purpose of a root cause To discover what produced an event

analysis: To create a process map or fishbone diagram to describe an event

To define what should have happened in an event

Recommendation

To reconstruct what happened before an event

24) When a medical error occurs, which is the best order Communicate with the patient, report the error to of priorities? appropriate parties, check the medical record,

care for the patient.

Report the error to appropriate parties, check the medical record, care for the patient, communicate with the patient.

Care for the patient, communicate with the patient, report the error to appropriate parties, check the medical record.

Check the medical record, care for the patient, communicate with the patient, report the error to appropriate parties.

25) For a hospital to have an effective culture of People know their concerns will be openly received safety, it's important that employees experience and treated with respect.

psychological safety. Which of the following is the Leaders actively create an environment where all best definition of psychological safety (in contrast staff are comfortable expressing their concerns. to fairness, transparency, and active leadership)? Patient safety problems aren't swept under the

rug. Team members have a high degree of confidence that the organization will learn from problems and

use them to improve the system.

People know they will not be punished or blamed for system-based errors.

26) Select the statement that best reflects taking a Saying, "We could solve this if only we had a leadership stance in health care: computer system that could report on this."

Saying, "Medical students shouldn't be blamed for things that aren't our fault."

Saying, "Patients are being hurt by this problem." Saying, "We should find out how big a problem this is. Let's count how often this is an issue on this unit."

27) Which of the following is NOT an example of Avoiding discussion of patient details while in effectively maintaining patient confidentiality? the elevator

Working with a family member as an interpreter if the patient says it's ok

Logging out of the EMR after you are finished using it

Discussing patient care matters with any staff who are responsible for that patient's care

28) Which of the following is part of a PDSA cycle? Evaluate sentinel events

Plan and pilot test data collection

Root cause analysis

Set the agenda and assign responsibility

29) Which of the following statements about increasing Increased quality is always associated with health care quality and the cost of care is true? increased cost

Technology (a cost) is essential to quality improvement

Processes that enhance quality may also decrease cost

Reducing medical errors does not reduce cost

**Feedback From Learning Activity**

30) The workshop contained content that was new to me. Strongly Disagree

Disagree Neutral Agree

Strongly Agree

31) The workshop contained information that will be Strongly Disagree relevant to my future career. Disagree

Neutral

Agree

Strongly Agree

32) The workshop was a comfortable process for me. Strongly Disagree

Disagree Neutral Agree

Strongly Agree

33) The time was adequate for the amount of information. Strongly Disagree

Disagree Neutral Agree

Strongly Agree

34) This content on safety and systems was appropriate Strongly Disagree for my stage of training. Disagree

Neutral

Agree

Strongly Agree

35) This workshop helped me think in a new/different way Strongly Disagree about the healthcare system. Disagree

Neutral

Agree

Strongly Agree

36) This workshop helped me think in a new/different way Strongly Disagree about my role. Disagree

Neutral

Agree

Strongly Agree

37) For the small group breakouts, the tasks were clear. Strongly Disagree

Disagree Neutral Agree

Strongly Agree

38) Our facilitator helped guide us through the Strongly Disagree exercises. Disagree

Neutral

Agree

Strongly Agree

39) Our facilitator helped us connect the exercises to Strongly Disagree our careers. Disagree

Neutral

Agree

Strongly Agree

40) Comments on small group facilitators.

**Feedback**

41) The environment was conducive for engaged, interactive learning.

42) Promotion of group process and

collaboration.

Poor Fair Good Very Good Excellent

43) Opportunity to improve critical thinking skills.

44) Opportunity to improve self-directed learning.

45) Disciplines involved were appropriate for the activity.

46) What aspects of the Interprofessional Activity were most helpful to you?

47) In what ways could the Interprofessional Activity be improved? Be sure to comment on any items rated as "Fair" or "Poor" in above question
