# Supplementary material for: Safety in Numbers: Successful Student-Approved Case-Based Interprofessional Safety Workshop Utilizing Simulated Real-Life Safety Cases
Source: MedEdPORTAL. 2020 Jan 31;16:10874. doi: 10.15766/mep_2374-8265.10874 (PMC7065299; doi:10.15766/mep_2374-8265.10874)
Supplement: Supplementary file 1 — A. Pre- & Postevent Surveys.docx B. IPE Safety Workshop Agenda.docx C. RCA AM Session Facilitator Guide.docx D. RCA AM Session Facilitator Annotated Case Time Line.docx E. RCA AM Session Student Case Time Line.docx F. RCA AM Session Interviewee Scripts.docx G. RCA AM Session Patient Background & EWS Info.docx H. RCA AM Session Media - Radiology.docx I. RCA AM Session Media - Oxygen Tanks.docx J. Corrective Action PM Session Facilitator Guide.docx K. Corrective Action PM Session Effectiveness Chart.docx L. Corrective Action PM Session Worksheet.docx M. Executive Case Summary.docx N. Large-Group Lecture Schedule & Topic List.docx O. PPT 1 - Contributing to a Culture of Safety.pptx P. PPT 2 - Systems Improvement.pptx Q. PPT 3 - Impact of Students and Residents on QI.pptx R. PPT 4 - Presentation of Safety Case.pptx S. PPT 5 - Disclosing Medical Errors.pptx T. PPT 6 - Training for Resilience.pptx U. PPT 7 - Introduction to Improvement Plans.pptx V. Facilitator Postworkshop Survey.docx [file mep-16-10874-s001.zip › B. IPE Safety Workshop Agenda.docx]

***Interprofessional Safety and Systems Improvement Workshop Agenda***

8:00 am Facilitator/Consultant “Faculty Development” for Morning Session
*Faculty Facilitators*

*Consultants from Patient Safety and Performance Improvement*

8:30 am Introductions /Agenda 

8:45 am Contributing to the Safety Culture 

9:15 am Systems Improvement: Safety Reporting, RL-6, and Root Cause Analysis (RCA)

9:45 am Impact of Students and Residents on Quality Improvement

10:15 am Case Presentation

10:30a Root Cause Analysis Small Group Session

*Faculty Facilitators; Consultants from Patient Safety and Performance Improvement*

12 noon LUNCH

1:00 pm Disclosing Medical Errors

1:20 pm Training for Resilience

1:40 pm Introduction to Improvement Plans

2:00 pm Improvement Plan Small Group Session
*Faculty Facilitators; Consultants from Patient Safety and Performance Improvement*

3:30 pm Improvement Plan Presentations to Hospital Executives

*Faculty Facilitators; Consultants from Patient Safety and Performance Improvement, Executives*

4:15 pm Dismissal
