# Supplementary material for: Safety in Numbers: Successful Student-Approved Case-Based Interprofessional Safety Workshop Utilizing Simulated Real-Life Safety Cases
Source: MedEdPORTAL. 2020 Jan 31;16:10874. doi: 10.15766/mep_2374-8265.10874 (PMC7065299; doi:10.15766/mep_2374-8265.10874)
Supplement: Supplementary file 1 — A. Pre- & Postevent Surveys.docx B. IPE Safety Workshop Agenda.docx C. RCA AM Session Facilitator Guide.docx D. RCA AM Session Facilitator Annotated Case Time Line.docx E. RCA AM Session Student Case Time Line.docx F. RCA AM Session Interviewee Scripts.docx G. RCA AM Session Patient Background & EWS Info.docx H. RCA AM Session Media - Radiology.docx I. RCA AM Session Media - Oxygen Tanks.docx J. Corrective Action PM Session Facilitator Guide.docx K. Corrective Action PM Session Effectiveness Chart.docx L. Corrective Action PM Session Worksheet.docx M. Executive Case Summary.docx N. Large-Group Lecture Schedule & Topic List.docx O. PPT 1 - Contributing to a Culture of Safety.pptx P. PPT 2 - Systems Improvement.pptx Q. PPT 3 - Impact of Students and Residents on QI.pptx R. PPT 4 - Presentation of Safety Case.pptx S. PPT 5 - Disclosing Medical Errors.pptx T. PPT 6 - Training for Resilience.pptx U. PPT 7 - Introduction to Improvement Plans.pptx V. Facilitator Postworkshop Survey.docx [file mep-16-10874-s001.zip › C. RCA AM Session Facilitator Guide.docx]

**Facilitator Guide – Mary Thompson Case**

Patient Safety and Systems Improvement Workshop

Root Cause Analysis Case

**Objectives:**

**Workshop and Root Cause Analysis (RCA) Case:**

This activity relates to the following Entrustable Professional Activities (EPAs) as outlined by the AAMC:

**EPA 9: Collaborate as a member of an interprofessional team.**

**EPA 13: Identify system failures and contribute to a culture of safety and improvement**

**Additional Educational Objectives for RCA session:**

1. Identify the strengths of an interprofessional healthcare delivery model
2. Apply systems knowledge to perform a root cause analysis on a real safety event
3. Engage current clinical faculty in the process of teaching safety and quality principles

**Morning Session and RCA Case:**

**Case Objectives:**

1. Review the timeline and events around a serious safety event.
2. Identify key interviews and practice exploring causes: e.g. Five Whys.
3. Connect case events to possible human or systems factors contributing to error.
4. Propose potential root causes of this patient’s death.
5. Recognize major patient safety events which require multiple people committing multiple seemingly innocuous mistakes that breach an organization’s fail-safe mechanisms

**Student participants:** Each group will have 10-12 students containing a mixture of different represented healthcare training programs

**Case Overview:**

The Mary Thompson case highlights principles of human factors and system factors in medical errors. The purpose of the case is to review how multiple errors may line up to produce an adverse event, and to identify potential areas of improvement in order to increase patient safety.

Ms. Thompson’s case follows her from initial admission and diagnostic workup to her adverse event. Students will work through the timeline of her day, and generate questions with the intent to identify potential root causes of her death that could put other patients at risk.

Though a discussion of the differential diagnosis and clinical management of COPD could be achieved, the focus of this session is instead the interactions between the patient and different parts of the *clinical microsystem* – the process by which she receives treatment and the errors that occur in her care.

**Introduction:** *10 minutes*

1. Introductions around the table.
2. Review the objectives, and the “charge” to the group:
   1. Work through the events of the case to identify next steps.
      1. These may include annotating the timeline, interviews with frontline providers, mapping processes, finding out what places look like, etc.
   2. Emphasize the need to look at not only human error but also **latent error** when conducting an RCA

**Case Review and Investigation:** *60 minutes*

1. Help students navigate through the RCA in a similar structure to a problem-based learning case.
2. Assign initial four roles:
   1. Team Leader(s) – *consider a student with more clinical experience for this role*
   2. Scribe 1: keeps a timeline/map of case
   3. Scribe 2: record the group's root causes
   4. Time keeper
3. Direct students to develop a timeline of events surrounding Ms. Thompson’s case. The team should identify information that will help them understand what happened.
4. As facilitator, you will have access to information you may provide them with as the need arises. These include:
   1. **Individuals to interview** (case-relevant providers and staff) (Appendix F)

- They may need guidance to recognize the need to interview some people, such as the radiology tech and radiology holding nurse.
- When a desired interview is requested, assign a student to interview the selected person (e.g., the transporter). Provide the student the “interview notes”.
- “Interviewers” should then summarize the notes for the group.
- You have access to interviews from:
  - - 1. **Senior medical resident (PGY-2)**
      2. **Medical student (M3)**
      3. **Bedside nurse (RN)**
      4. **Transporter**
      5. **Radiology tech**
      6. **Radiology holding area nurse (RN)**
  1. **Additional factual/medical information they need (Appendix F)**
     1. References for “standard” vital signs (on ***Patient*** ***Handout***)
     2. Basic information on COPD, treatments for COPD exacerbations (on ***Patient*** ***Handout***)
     3. Early Warning Score chart and videos (***EWS Handout***)
  2. **Places and objects to “see”**
     1. ***EWS Handout***
        1. Early Warning Score table
        2. Contains weblink to video explaining EWS
        3. Brief summary of current EWS policy
     2. ***Oxygen/RT (Respiratory Therapy) images* (Appendix H)**
        1. Wheelchair with oxygen tank attached
        2. Wheelchair with oxygen tank attached (2)
        3. Oxygen flow rate gauge (how many Liters/minute)
        4. Oxygen storage on nursing unit
        5. Pressure Gauge (how much oxygen present (psi))
        6. “Interview” with Respiratory Therapy
        7. Oxygen supply chart (Time Remaining at X liter flow, Y Pressure Gauge)
     3. ***Radiology images – includes Holding Room and CT scanner*** **(Appendix I)**
        1. Picture of holding room with wall oxygen visible
        2. Picture of holding room (2)
        3. CT-scanner with wall oxygen visible (front)
        4. CT-scanner with wall oxygen visible (side, circled in red)
        5. CT-scanner with wall oxygen visible (closeup, circled in red)
        6. Close-up of wall oxygen with flow rate gauge “Christmas Tree” attachment set up – **this item was missing in this case**
  3. **Facilitators’ Annotated Case Timeline (Appendix D)**
     1. Includes details of key moments pulled from the interviews
     2. Includes key learning points that may be raised when these times are being discussed, related to the “cheat sheet” – these may also be discussed at the end as they assemble their list of root causes.

**Exploration of Root Causes:** *20 minutes*

1. Assemble potential root causes for Ms. Thompson’s death.
   1. Types of identified root causes may include:
      1. Human Factors
         1. Medical knowledge, clinical acumen
         2. Slips or lapses – reliance on “automatic cognition”: tasks which require minimal thinking, "auto-pilot" tasks
      2. System errors
         1. Error due to system issue which predisposes a person to make a mistake
         2. The “reasonable person” rule – a reasonable person could make this error
      3. Normalized deviance
      4. Rule or standard of care is **consistently disregarded** and becomes over time a "normalized" practice pattern
2. **FACILITATORS: please have scribe #2 write root causes on index cards & turn in at lunch.**

**Tips, Tricks, and Trouble-shooting:**

- Keep to a timely pace, as there is only 90 minutes for this RCA session. Aim to have a full timeline of the case generated by the group and “interviews” done in the first 60-70 minutes, and spend the last 20-30 minutes better defining the potential root causes.
- Use the “**Five Whys**” to clarify what questions/information are needed by the interviews.
  - **Five Whys**: Interrogative technique used to explore the cause and effect relationships underlying a problem. The primary goal of the technique is to determine the root cause of a problem by repeating the question "Why?" Each question forms the basis of the next question.
- **USE THE TIMELINE! This has all critical information that is in the Interviews and Media Files in one convenient location.**
- Re-direct students if they get off track with their line of thinking/questioning:
  - *They may want to spend too much time on the medical details of the case, which is not where the crux of the RCA lies. The medical team chose an appropriate diagnostic test in this case; most of the root causes lie within the realm of system errors.*
- Be aware of the emotions this type of exercise can elicit. Students may (appropriately!) feel frustrated, sad, angry, or uncomfortable when addressing flaws in our system and discussing a negative outcome. They may need your support in validating these emotions; remind them that we will talk about the “what’s next” in the afternoon session.

**WHAT’S NEXT:**

**Lunch!!!**

**Facilitators – Please turn in your group’s Root Cause index cards to the event leader as these will be used in the afternoon session.**
