# Supplementary material for: Safety in Numbers: Successful Student-Approved Case-Based Interprofessional Safety Workshop Utilizing Simulated Real-Life Safety Cases
Source: MedEdPORTAL. 2020 Jan 31;16:10874. doi: 10.15766/mep_2374-8265.10874 (PMC7065299; doi:10.15766/mep_2374-8265.10874)
Supplement: Supplementary file 1 — A. Pre- & Postevent Surveys.docx B. IPE Safety Workshop Agenda.docx C. RCA AM Session Facilitator Guide.docx D. RCA AM Session Facilitator Annotated Case Time Line.docx E. RCA AM Session Student Case Time Line.docx F. RCA AM Session Interviewee Scripts.docx G. RCA AM Session Patient Background & EWS Info.docx H. RCA AM Session Media - Radiology.docx I. RCA AM Session Media - Oxygen Tanks.docx J. Corrective Action PM Session Facilitator Guide.docx K. Corrective Action PM Session Effectiveness Chart.docx L. Corrective Action PM Session Worksheet.docx M. Executive Case Summary.docx N. Large-Group Lecture Schedule & Topic List.docx O. PPT 1 - Contributing to a Culture of Safety.pptx P. PPT 2 - Systems Improvement.pptx Q. PPT 3 - Impact of Students and Residents on QI.pptx R. PPT 4 - Presentation of Safety Case.pptx S. PPT 5 - Disclosing Medical Errors.pptx T. PPT 6 - Training for Resilience.pptx U. PPT 7 - Introduction to Improvement Plans.pptx V. Facilitator Postworkshop Survey.docx [file mep-16-10874-s001.zip › D. RCA AM Session Facilitator Annotated Case Time Line.docx]

**Detailed Case Timeline for Facilitators**

**General Notes**: Students should ask questions to discover details of the timeline below. Do not give out facts unless they ask appropriate questions.

Options for information include **Interviews** (Appendix F) with relevant people involved or **Oxygen** **Media** **Files (Appendix I) and Radiology Media Files** (Appendix H) which include images and charts.

If students ask for events or details outside this timeline, they are probably not relevant. You can answer based on your experience or simply say you don’t know.

Key **Learning Points** related to safety culture are noted in the right-hand column as well and are **bolded in RED**.

**Admission Day 1:**

| **Time** | **Event** | **Note(s)** | **Learning Points** |
| --- | --- | --- | --- |
| 3:00 pm | ED Arrival. Treatment begins.  BP 120/80 Pulse 110 RR 24  O2 sat 94% on 5L O2 (home level 3L) | Receives following meds at appropriate doses and times   - Albuterol - Steroids - Antibiotics | Review normal range of vital signs.  Patient received appropriate treatment for COPD. |
| 5:00 pm | Diagnostic studies are at baseline | CBC, electrolytes, chest x-ray.  Blood gas not performed. | Initial workup is typical. ABG may or may not have changed trajectory. |
| 7:00 pm | No response to medication. Admitted to internal medicine | General floor. Early warning score 6 (EWS), which is appropriate for floor admission | **Media:**   - Define *Early Warning Score* - **Chart, weblink in EWS Handout (Appendix G)**   **High-reliability note**: This patient presents as somewhat “typical,” creating the risk of auto-pilot behavior and potential for missing anomalies. |

**Next morning in hospital Day 2:**

| **Time** | **Event** | **Note(s)** | **Learning Points** |
| --- | --- | --- | --- |
| 8:00 AM | Treatment team rounds | Who was present?   - Senior resident, 2 interns, 1 student   Patient vital signs:   - BP 112/70 Pulse 125 Resp Rate 25 - O2 sat 94% still on 5L - Early warning score 8. Rapid response was supposed to be called but is not.   What was the protocol?   - Common at that time for rapid response to not be called for elevated EWS   Did the team recognize change in vital signs?  How might this change management? Might monitoring have made a difference? | **Interview Opportunities: (Appendix F)**   - *Resident (in* ***Interview folder****)* - *Medical Student (in* ***Interview folder****)*   **Media: (Appendix G)**   - *Early Warning Score* - **EWS Chart, weblink in EWS Handout**   What is the protocol now?   - Rapid Response and first-call provider notified for EWS 8+   **High-reliability note:** determining why rapid response wasn’t called?   - Cognitive error? - Normalized deviance? (Rule-based error) - Don’t know to call rapid  (Knowledge-based error) - Know to call but missed it?  (Skill-based error) - **Was there a standard in place? Not routinely followed but there is now**. |
| 9:00 | CT Scan of chest ordered to r/o pulmonary embolism. | Clinical note: Correct differential diagnosis; PE can be a trigger of COPD exacerbation.   - "had the right answer" but still bad outcome | This is to demonstrate appropriate medical reasoning was done. **Avoid spending too much time on this pathway.** |
| 9:30 | Radiology staff schedules CT for 12 pm. | How are radiology studies scheduled and performed?   - Radiology staff schedule studies | **Interview Opportunity: (Appendix F)**   - *Radiology Tech (in* ***Interview folder****)*   **Encourage students to map out the process of how patient gets from point A to point B.** |
| 9:00 | Radiology staff enters order for patient transport at 11 AM | How is transportation set up for radiology?   - Transport office has own computer system for scheduling transports. They request notification at least 1 hour prior to transport time. - Patients taken to radiology holding area 1 hour before study time in order to have smooth schedule in radiology suite, reducing CT scanner downtime. | **Interview Opportunity: (Appendix F)**   - *Radiology Tech (in* ***Interview folder****)* - *Transporter (in* ***Interview folder****)*   Point out duplicate systems for orders.  **High-reliability note:** Introduction of variability and duplication creates opportunity to introduce error |
| 11:00 | Transporter arrives in nursing unit with wheelchair | - Wheelchairs are kept by transport department | **Interview Opportunity: (Appendix F)**   - *Transporter (in* ***Interview folder****)* |
| 11:00 | Transporter gets oxygen tank and places it in bracket in wheelchair | How/Where is oxygen stored?   - Oxygen tanks are kept in supply closet in case, standing upright. 16 tanks per case. - Each day, in the evening, respiratory therapy checks oxygen tanks and any tanks under ½ full are sent to be refilled. - **Full and empty tanks are kept in same case.** - Gauge on tank is small and hard to notice.   Who is in charge of checking oxygen?   - Neither transporter nor nurse specifically charged w/ checking oxygen level in tank. - Nobody knows how full Mrs. Thompson’s portable oxygen tank was on this day | **Interview Opportunity: (Appendix F)**   - *Transporter(in* ***Interview folder****)* - *Respiratory Therapist (in* ***Oxygen*** ***Media Files****)*   **High-reliability note**: Did respiratory check the tanks the previous evening? If not, why?  **Oxygen Media Files: (Appendix I)**   - ***RT/Oxygen images*** - *wheelchair* - *oxygen flow rate gauge* - *oxygen tank storage* - *oxygen tank with pressure gauge (how much O2 is left in the tank)* - *Info from* ***Respiratory Therapy*** - *Chart for O2 tank longevity*   Where is the cross checking on the oxygen level of stored tanks?  **High-reliability note**: Not clear which tanks are full and empty without close inspection  **High-reliability note:** Missed opportunity for cross-checking for reliability.  Why not charged? System error |
| 11:00 | Nurse gets patient into wheelchair. Disconnects patient’s oxygen tubing from wall oxygen, connects the tubing to tank and turns tank to 5 liters | - Oxygen tank at "full" 2000 psi at flow 5L per minute should last 90 minutes - How long was patient gone? - No one knows how much oxygen was in the tank at the start of the trip. | **Interview Opportunity: (Appendix F)**   - *Bedside Nurse (in* ***Interview folder****)* - *Transporter(in* ***Interview folder****)* - *Respiratory Therapist (see* ***Oxygen*** ***Media Files****)*   **Oxygen Media Files: (Appendix I)**   - ***RT/Oxygen images:*** - *wheelchair* - *oxygen flow rate gauge* - *oxygen tank storage* - *oxygen tank with pressure gauge (how much O2 is left in the tank)* - *Info from* ***Respiratory Therapy*** - *Chart for O2 tank longevity* |
| 11:15 | Patient taken to radiology holding area. | - Patients are supposed to be switched from oxygen tank to wall oxygen. But all wall sources of oxygen are taken, so transporter leaves patient on oxygen tank. - Is the transporter prepared for an atypical situation? | **Interview Opportunity: (Appendix F)**   - *Transporter (in* ***Interview folder****)* - *Radiology Nurse (in* ***Interview folder****)*   **Radiology Media Files: (Appendix H)**   - *Image of holding area and oxygen outlets* - *CT scanner and oxygen outlets* - *“Christmas Tree” oxygen flow gauge –* ***this was missing!*** *In CT scanner*   **Normalized deviance**  **High reliability note:** Is there a standard practice in place if patient must stay on tank oxygen? System problem |
| 11:20 | Transporter cannot find the nurse who is supposed to be covering holding area. So he leaves patient in holding area. | Why isn't the nurse available?   - Nurses routinely needed to help with procedures - 2 nurses are assigned to procedures. But the holding area nurse is routinely pulled when a third procedure is being performed. | **Interview Opportunity: (Appendix F)**   - *Transporter (in* ***Interview folder****)* - *Radiology Holding Area Nurse (in* ***Interview folder****)*   **Normalized deviance**  **High-reliability note:** System induced reliability issue when compromising the two-nurse model. What’s driving the compromise? |
| 11:45 | Holding area nurse returns to holding area, speaks briefly to patient. Patient indicates she is OK. Nurse leaves her on oxygen tank. | - Patient not transitioned to wall oxygen at this point. - No vital signs taken - May not be standard practice in non-monitored patients. | **Interview Opportunity: (Appendix F)**   - *Radiology Holding Area Nurse (in* ***Interview folder****)*   **Normalized deviance**  **High-reliability note:** Why didn’t nurse check vitals? Why isn’t it standard practice to check even if patient says she’s OK? Auto-pilot behavior |
| 12:15 | CT Tech gets patient. Puts patient on CT scanner | - There is wall oxygen in CT room. Techs are supposed to disconnect from oxygen tank and connect to wall source. - ***Wall source has no gauge – the whole oxygen flow gauge and Christmas tree was missing***, so oxygen tubing cannot be connected.   **Too few oxygen flow gauges for the wall units so this one had been moved to holding – equipment issue.**   - Patient left on oxygen tank   What is the flow rate of the oxygen? How long will the oxygen supply last? | **Interview Opportunity: (Appendix F)**   - *Radiology tech (in* ***Interview folder****)* - *Respiratory Therapist (in* ***Oxygen*** ***Media Files)***   **Radiology Media Files: (Appendix H)**   - *Picture of holding area and oxygen outlets* - *CT scanner and oxygen outlets* - *“Christmas Tree” oxygen flow gauge –* ***this was missing!*** *In CT scanner* - ***May want to look again at table*** *of oxygen tank longevity in* ***Oxygen media files***   **Normalized deviance**  **High-reliability note**: Why no gauge? Variability introduces opportunity for error. Is there a practice standard when wall oxygen is not available? **System error** |
| 12:30 | CT scan done. Patient put back in chair. Tech remembers patient seemed sleepy | - **Techs are not trained to do clinical assessment and are not expected to identify clinical deterioration** | **Interview Opportunity: (Appendix F)**   - *Radiology tech (in* ***Interview folder****)*   **High-reliability note:** Is there a practice standard or training for techs to notice uncommon or worsening condition and escalate concern? **System error** |
| 12:40 | Placed back in holding. Again, all wall oxygen sources taken. Patient left on oxygen tank |  | **Normalized deviance** |
| 12:40 | Holding area nurse is in another procedure. No documentation of nursing assessment | - No standard policy in place requiring patient's to have nurse assessment prior to leaving holding area | **Interview Opportunity: (Appendix F)**   - *Radiology tech (in* ***Interview folder****)* - *Radiology Holding Area Nurse (in* ***Interview folder****)*   **Normalized deviance** |
| 1:45 | Transporter comes to get patient. He recalls she was sleeping | - Transporters not trained to do any clinical assessment - At this point was likely that the tank had run out of oxygen, but we do not know exactly when that occurred. | **Interview Opportunity: (Appendix F)**   - *Transporter (in* ***Interview folder****)*   **High-reliability note:** Is there a practice standard or training for transporters to notice uncommon or worsening condition and escalate concern? **System error** |
| 2:00 | Transporter arrives in nursing unit. Parks wheelchair in room. Notifies nurse. She is in midst of other task | - No policy requiring immediate nursing assessment of patient when they return to floor | **Interview Opportunity: (Appendix F)**   - *Transporter (in* ***Interview folder****)* - *Bedside Nurse (in* ***Interview folder****)*   **Normalized deviance** |
| 2:20 | Nurse finds patient in arrest. |  |  |

**Potential root causes:**

- Failure of treatment team and nursing to identify worsening vital signs from when she was admitted
- Sending patient with abnormal vitals off unit without monitor or nurse
- Oxygen tank was almost certainly ¼ full or less, based on time sequence and flow rate of oxygen
  - Full and empty tanks stored together
  - Tanks only checked once daily
  - Gauge on tanks small, had to notice if they are empty
  - Nobody assigned to check how full the tanks are before use
- No handoff of care between nurses. This is especially important in patient with unstable vital signs.
- Long wait in holding area before and after study
- Missing nurse in holding area because she was pulled to help with procedure. This was a routine practice but there was no rule against this.
- No vital signs routinely checked in radiology holding area
- Inadequate number of oxygen outlets in holding area. It became a routine practice to leave patients on oxygen tanks
- Missing oxygen flow rate gauge in CT room. Gauges are removable and there were not enough in the radiology area. So they were taken from one room to another.
- Techs decided to leave patient on tank. This had become a routine practice.
- On return to holding area, all problems identified had recurred.
- Delay from time patient returned to room until nurse went to check on patient.

**Important notes:**

- Early Warning Scores are a combination of vital signs, oxygen requirement and alertness that identify patients at risk of deterioration. An EWS of greater than 6 is supposed to trigger activation of Rapid Response team and often results in transfer to ICU. If students need more clarification on the EWS there is a link to a video in the EWS handout.
- Transporters have no medical training and are not expected to identify patients in distress
