# Supplementary material for: Safety in Numbers: Successful Student-Approved Case-Based Interprofessional Safety Workshop Utilizing Simulated Real-Life Safety Cases
Source: MedEdPORTAL. 2020 Jan 31;16:10874. doi: 10.15766/mep_2374-8265.10874 (PMC7065299; doi:10.15766/mep_2374-8265.10874)
Supplement: Supplementary file 1 — A. Pre- & Postevent Surveys.docx B. IPE Safety Workshop Agenda.docx C. RCA AM Session Facilitator Guide.docx D. RCA AM Session Facilitator Annotated Case Time Line.docx E. RCA AM Session Student Case Time Line.docx F. RCA AM Session Interviewee Scripts.docx G. RCA AM Session Patient Background & EWS Info.docx H. RCA AM Session Media - Radiology.docx I. RCA AM Session Media - Oxygen Tanks.docx J. Corrective Action PM Session Facilitator Guide.docx K. Corrective Action PM Session Effectiveness Chart.docx L. Corrective Action PM Session Worksheet.docx M. Executive Case Summary.docx N. Large-Group Lecture Schedule & Topic List.docx O. PPT 1 - Contributing to a Culture of Safety.pptx P. PPT 2 - Systems Improvement.pptx Q. PPT 3 - Impact of Students and Residents on QI.pptx R. PPT 4 - Presentation of Safety Case.pptx S. PPT 5 - Disclosing Medical Errors.pptx T. PPT 6 - Training for Resilience.pptx U. PPT 7 - Introduction to Improvement Plans.pptx V. Facilitator Postworkshop Survey.docx [file mep-16-10874-s001.zip › E. RCA AM Session Student Case Time Line.docx]

**Mrs. Thompson Case Timeline for Students – RCA Breakout Session**

*Below you will find the timeline of events as they were first known at the time of death of Mrs. Thompson. Use this timeline to help figure out what errors occurred in her care and which members of her healthcare team you would like to interview as you being your Root Cause Analysis.*

**Admission Data**

| **Time** | **Event** | **Note(s)** |
| --- | --- | --- |
| 15:00 | ED Arrival. Treatment begins. | Vitals: Pulse 110, RR 24, BP 120/80, 94% on 5L oxygen  Receives following meds at appropriate doses and times: albuterol, steroids, antibiotics |
| 17:00 | Diagnostic studies are at baseline | CBC, electrolytes, chest x-ray.  EWS6 |
| 19:00 | No response to medication. Admitted to internal medicine |  |

**Next morning in hospital**

| **Time** | **Event** | **Note(s)** |
| --- | --- | --- |
| 8:00 | Treatment team rounds | Rounding team: attending, upper level resident, two interns, medical student  Patient vital signs: Pulse 125, RR 25, BP 112/70, 94% on 5L oxygen  EWS 8 |
| 9:00 | CT Scan of chest ordered to r/o pulmonary embolism. |  |
| 11:00 | Transporter arrives to unit with wheelchair. Nurse connects patient to portable oxygen tank. |  |
| 11:15 | Patient taken to radiology holding area. |  |
| 12:15 | CT Tech gets patient. Puts patient on CT scanner | CT PE study performed |
| 1:45 | Transporter comes to get patient. |  |
| 2:00 | Transporter arrives in nursing unit. Parks wheelchair in room. Notifies nurse. |  |
| 2:20 | Nurse enters room and finds patient in arrest. | Medical team runs code and declares patient dead at 2:40 pm. |
