# Supplementary material for: Safety in Numbers: Successful Student-Approved Case-Based Interprofessional Safety Workshop Utilizing Simulated Real-Life Safety Cases
Source: MedEdPORTAL. 2020 Jan 31;16:10874. doi: 10.15766/mep_2374-8265.10874 (PMC7065299; doi:10.15766/mep_2374-8265.10874)
Supplement: Supplementary file 1 — A. Pre- & Postevent Surveys.docx B. IPE Safety Workshop Agenda.docx C. RCA AM Session Facilitator Guide.docx D. RCA AM Session Facilitator Annotated Case Time Line.docx E. RCA AM Session Student Case Time Line.docx F. RCA AM Session Interviewee Scripts.docx G. RCA AM Session Patient Background & EWS Info.docx H. RCA AM Session Media - Radiology.docx I. RCA AM Session Media - Oxygen Tanks.docx J. Corrective Action PM Session Facilitator Guide.docx K. Corrective Action PM Session Effectiveness Chart.docx L. Corrective Action PM Session Worksheet.docx M. Executive Case Summary.docx N. Large-Group Lecture Schedule & Topic List.docx O. PPT 1 - Contributing to a Culture of Safety.pptx P. PPT 2 - Systems Improvement.pptx Q. PPT 3 - Impact of Students and Residents on QI.pptx R. PPT 4 - Presentation of Safety Case.pptx S. PPT 5 - Disclosing Medical Errors.pptx T. PPT 6 - Training for Resilience.pptx U. PPT 7 - Introduction to Improvement Plans.pptx V. Facilitator Postworkshop Survey.docx [file mep-16-10874-s001.zip › F. RCA AM Session Interviewee Scripts.docx]

**BEDSIDE NURSE**

**BACKGROUND INFORMATION:**

Nurse Harris has been an RN since 2010.
Job history: Emergency Dept. nurse (5 years); General Med/Surg unit (2015-present).

Context: “I enjoy my job and taking care of people, but it is a very stressful occupation. We usually cover 4-5 patients at a time. We have been understaffed recently as several nurses are out on sick or maternity leave, so our unit has been asking us to pull extra shifts and cover the max number of patients on the floor. It feels impossible to attend to the needs of all my patients at times.”

**CASE TIMELINE:**

| **Timeline** | **Nursing Observations/Activity** |
| --- | --- |
| 7 AM | Received checkout from night shift Informed Ms. Thompson admitted for COPD exacerbation treated with 5L NC, nebulizers, IV solumedrol, antibiotics |
| 7:30 AM | Checked on Ms. Thompson, asleep in bed Received AM vitals from CNA, notable for tachycardia and tachypnea Made sure she received her AM meds |
| 9 AM | Noticed order in Epic for CT PE study on Ms. Thompson |
| 11 AM | Paged by floor secretary – transport arrived to take patient to the CT scanner Transitioned patient into wheelchair and connected oxygen tubing to portable tank, turned it to 5 liters Did not recheck vitals prior to leaving the floor |
|  | *I remember thinking maybe she should be on a monitor given her abnormal morning vital signs but there wasn’t an available monitor and our floor was so short-staffed, I couldn’t leave the unit to go with her. She was talking though so that made me feel better.* |
| 12 PM | Went to lunch |
| 1 PM | Rounded on my patients Went to help start IV on another nurse’s patient |
| 2 PM | Paged by floor secretary – Ms. Thompson is back in her room from the CT scan |
| 2:20 PM | Found Ms. Thompson unresponsive in her wheelchair Her oxygen was still connected to the portable tank Code Blue called |
| 2:22 PM | Dr. Scott and medical team started CPR and ACLS protocols. After 20 minutes of running the code, Dr. Scott declared the patient deceased |
| 2:42 PM | Time of death |
|  | *I don’t know what happened, I was completely devastated when the patient died. She seemed fine before she left for the CT scan and even made a little joke to me about the weather. She was such a sweet lady. I think about what if that had been my mom, how angry and horrible I would feel. If only I hadn’t been caught up with doing that IV maybe I could have checked on her sooner and she would still be alive.* |

**INTERNAL MEDICINE RESIDENT**

**BACKGROUND INFORMATION:**

Name/Background: Dr. Scott is a second year internal medicine resident.
Attended medical school at Medical University of South Carolina.
Considering a fellowship in cardiology after completing residency.

*This was my first month as the upper level resident on a general medicine ward team. I was nervous about being responsible for twenty sick patients and having to rely upon my interns to relay information about their patients. This was a new leadership role for me.*

**CASE TIMELINE:**

| **Timeline** | **Resident Observations/Activity** |
| --- | --- |
| 6:30 AM | Arrived to hospital to get checkout from overnight upper level |
| 8 AM | First evaluated patient while on morning rounds Patient was tachypneic but able to speak in sentences and oriented; |
| 8:30 AM | We made the decision to order the chest CT PE study as a possible cause of her COPD exacerbation |
|  | *I was concerned the morning on rounds she may eventually need to be moved to a higher level of care for closer monitoring, but wanted to wait and see what the CT scan showed and how she responded to the IV solumedrol and nebulizers. She didn’t seem that far off her baseline.* |
| 11 AM | Intern informed me Ms. Thompson left to go to CT scanner We were still rounding as a team on our morning patient rounds |
| 12:30 PM | Finished morning rounds Went to talk to another patient’s family regarding new diagnosis |
| 2:20 PM | Heard the Code Blue for Ms. Thompson’s room |
| 2:22 PM | Began running the code and performing ACLS Monitor showed asystole and could not get a shockable rhythm |
| 2:42 PM | I made the decision to call off the code and pronounced Ms. Thompson dead |
|  | *This was the first patient that died under my leadership as an upper level resident. I will never forget it. I had to page our attending and tell him what had happened. I listened as he called her family and told them what had happened. Her family was devastated, and couldn’t understand how she had gone downhill so fast. I had no idea when I left the patient’s room that morning on rounds that I would not see her alive again. I felt like if I had just not ordered the CT scan, she would still be here.* |

**MEDICAL STUDENT**

**BACKGROUND INFORMATION:**

Name/Background: Jordan Williams, a third year medical student assigned to the general medicine service as part of the Internal Medicine clerkship.
*I am both delighted and terrified to finally be in the clinical world. Prior to medical school I had a couple of shadowing experiences in a small town pediatrics clinic but no hospital experience. This is my first rotation. After two weeks I feel more comfortable collecting data on my patients and reporting to my team. However, I still feel like I have a lot to learn with regards to my knowledge base and clinical acumen. My team (residents and attending) seem to be very caring and capable physicians – they manage the patients in an effective and compassionate way.*

**CASE TIMELINE:**

| **Timeline** | **Medical Student Observations/Activities** |
| --- | --- |
| 6 AM | Arrived to round on my two patients and learned that Ms. Thompson had been admitted overnight and was assigned to me. I read through her H&P and made myself an index card of information to present later in the morning on rounds. The data included primary diagnosis, vital signs, lab results, radiology results and medication list. |
| 6:45 AM | Checked on Ms. Thompson, asleep in bed. I listened to her heart and lungs without waking her up. Her heart rate seemed fast but she did not appear to be struggling to breathe. |
| 7:45 AM | I popped my head in Ms. Thompson’s room one more time and she was stirring, so I introduced myself and asked if there was anything that she needed. She was very appreciative and reported that she was just really tired from the ordeal of being admitted to the hospital but that she had everything she needed. I was glad to see her awake before rounds started. She seemed a little short of breath but she did not seem too worried about it. |
| 8 AM | We rounded on Mrs. Thompson first since she was a new admit. My resident seemed fairly concerned about her shortness of breath and said that we might need to get imaging to rule out a pulmonary embolus. I need to review pulmonary embolus, have not had a patient with that diagnosis yet but remember learning about it in the pulmonary block. Rounds were very busy this day and we did not finish until just prior to noon when there was a scheduled mandatory medical student conference. |
| Noon | *Attended noon conference* |
| 1 PM | Went to lunch |
| 1:30 PM | Went to the student workroom to review pulmonary embolism in UpToDate |
|  | Paged by floor secretary – Ms. Thompson returned from CT scan |
| 2:22 PM | I heard the overhead page for code blue and recognized the room number as being one of my patients. My heart sank but I joined the team that was in active resuscitation mode. It all happened so fast. Despite heroic efforts, Ms. Thompson was pronounced deceased at 2:42. My team was obviously distressed.  I ask myself if this is something that I can experience and even participate in on a regular basis. I’m not sure. |

**RADIOLOGY HOLDING ROOM NURSE**

**BACKGROUND INFORMATION:**

Name/Background: Nurse Speaks; RN for 3 years. Prior position in Cardiology at another hospital in the area for 2 years; moved here to radiology a year ago. Has a goal to become a nurse practitioner.

*Just like other parts of the hospital, we have been understaffed recently but I love taking care of my patients.*

*Our section usually has three nurses, one who monitors the holding area and two who primarily assist with procedures. It’s been really busy lately, though, and a lot of the time, the holding room nurse is called in to assist with procedures too. I don’t mind because I’m used to the sicker people from my time on Cards, but I sometimes feel like my attention is split.*

**CASE TIMELINE:**

| **Timeline** | **Nursing Observations/Activity** |
| --- | --- |
| 7 AM | Received checkout from night shift. |
| 7:30 AM | Checked in on patients in holding area, recorded vitals for newly arrived patients. |
| 9:15 AM | Revisited radiology and procedure order lists for the morning, discussed division of labor with the other nurse working Radiology (Nurse Riley). |
| 11:15 AM | Called to assist with an unexpected procedure, left holding room. |
| 11:45 AM | Returned to holding room, greeted Ms. Thompson who was scheduled for CT PE study. Attempted to transfer her O2 supply from portable tank to wall source, but other patients in the holding area occupied all wall sources. Spoke to patient, who seemed stable and conversant. She said she was always on O2 |
| 11:50 AM | Paged to procedure suite to assist with additional procedures. |
| 12:00 PM | Went to lunch, asked nurse Riley to cover holding area. |
| 12:40 PM | Paged to assist nurse Riley for add-on procedures, left holding room. |
| 1:50 PM | Returned to holding room, noticed Ms. Thompson was signed out and heading back to the floor per transport log. |

**RADIOLOGY TECHNOLOGIST**

**BACKGROUND INFORMATION:**

Name/Background: Millie Martin, a radiographic technologist. Employed here for the past 2 years. Previously employed at different hospital center for 5 years.

Training/Certification: Associate of Applied Science degree where Ms. Martin completed course work including Intro to Patient Care and Radiography and Radiographic Quality Management. Certified, American Registry of Radiologic Technologists (ARRT).

*I have really enjoyed working here for the past couple years, it is a much better hospital than my previous employer and I get along well with my coworkers.*

CASE TIMELINE:

| **Timeline** | **Radiology Technologist Observations/Activity** |
| --- | --- |
| 7 AM | Received checkout from night shift. |
| 7:20 AM | Checked on the list of inpatients with orders for CT scans and organized by priority identified in the patient order in EHR |
| 7:45 AM | Discussed division of labor with the other technologists working CT Radiology. |
| 8:45 AM | New order for CT chest to r/o PE received (patient - Ms. Thompson), Identified emergency slot at noon and inserted patient into our schedule. |
| 9:00 AM | Using the separate request system for transport, ordered patient transport for 11 am for Ms. Thompson.  We always have them come an hour ahead of time – otherwise the scanner sits empty for too long. |
| 12:15 PM | Brought patient from holding room to CT scanner room. Patient is in a wheelchair and is on oxygen currently supplied by oxygen tank.  In CT scan room attempted to connect oxygen to wall source but flow gauge setup – the Christmas Tree thing - was missing, and I couldn’t attach the tubing properly. This happened a lot – we didn’t have enough and they were removable, so they often got moved from room to room.  Realizing that the CT for PE is a very brief test and that we have other urgent patients waiting, I decided to proceed using the oxygen tank. I assisted Ms. Thompson to the scanner and she seemed fairly comfortable. |
| 12:30 PM | The scan proceeded uneventfully. Ms. Thompson even fell asleep during the procedure. I assisted her back to her wheelchair and returned her to the holding room. The holding room nurse was not present as she had been called to another procedure. I let transport know she was done since the nurse was busy, and then I proceeded to take the next patient back to be scanned. |

**TRANSPORTER**

**BACKGROUND INFORMATION:**

Name/Background: Casey Jones; transporter for about 6 months. Education: GED. Some training on the job. Taking night classes at community college to become a certified nursing assistant.

*The Transport Tracking System (TTS) can be overwhelming. Sometimes there is a lot of pressure because we work all day to get patients all around the hospital to tests and procedures. No one likes waiting. I keep a good attitude and I like talking with my patients and try to make them feel better. I enjoy my job, but it’s tiring. My back and feet hurt, especially at the end of a shift.*

*I have to punch out promptly at the end of the shift, because I can’t do overtime. My shift is 6AM-3PM with an hour lunch usually. That day they asked me to take a 30 minute lunch because it was so busy, and end at 2:30.*

**CASE TIMELINE:**

| **Timeline** | **Transporter Activities/Observations** |
| --- | --- |
| 6 AM | Punch in, receive first assignment. Radiology today. |
| 6:05AM to 10:15AM | Using the TTS, transported multiple patients from all over the hospital from their rooms to radiology and back. |
| 10:15-10:45AM | Took lunch break. |
| 10:45 AM | Received TTS assignment from Ms. Thompson’s floor to radiology. Her test was at 12. They like to have patients there an hour before. TTS said chair was ok, so got a wheelchair. |
| 10:55 AM | Arrived on nursing unit with the wheelchair. The nurse said she was on oxygen, and we would have to get a tank. She said it was in the supply closet. The supply closet had a single case with all the oxygen tanks. I got a tank from the supply closet and put it on the back of the wheelchair. I don’t know how to read the oxygen tank.  Ms. Thompson’s nurse attached the tank to her oxygen tubing and set it up. Ms. Thompson was telling us she’d been up all night in the ED. |
| 11:10-11:20 AM | Brought Ms. Thompson to radiology.  Paged twice for additional transport, including bringing one person from radiology to another part of the hospital.  I know the nurse is supposed to switch the patient from the tank to the wall oxygen, but the nurse wasn’t there when I got there.  I noticed there were no oxygen spots open.  I waited for a few minutes, but I kept getting paged and we are told not to wait more than 5 minutes for a patient.  Ms. Thompson seemed comfortable and she had her oxygen on then. |
| 11:25 to 1:30 PM | Other calls and other transports. |
| 1:45 PM | Arrived to radiology holding area and signed Ms. Thompson out.  She was sleeping. I brought her back to the nursing unit. |
| 2:00 PM | Arrived in nursing unit. Brought her (sleeping) to her room.  Walked out to get nurse to help transfer her back to bed, but she was in another room and said she would get help and move her in a few minutes. I had to leave to do my last call and still punch out on time. |
