# Supplementary material for: Safety in Numbers: Successful Student-Approved Case-Based Interprofessional Safety Workshop Utilizing Simulated Real-Life Safety Cases
Source: MedEdPORTAL. 2020 Jan 31;16:10874. doi: 10.15766/mep_2374-8265.10874 (PMC7065299; doi:10.15766/mep_2374-8265.10874)
Supplement: Supplementary file 1 — A. Pre- & Postevent Surveys.docx B. IPE Safety Workshop Agenda.docx C. RCA AM Session Facilitator Guide.docx D. RCA AM Session Facilitator Annotated Case Time Line.docx E. RCA AM Session Student Case Time Line.docx F. RCA AM Session Interviewee Scripts.docx G. RCA AM Session Patient Background & EWS Info.docx H. RCA AM Session Media - Radiology.docx I. RCA AM Session Media - Oxygen Tanks.docx J. Corrective Action PM Session Facilitator Guide.docx K. Corrective Action PM Session Effectiveness Chart.docx L. Corrective Action PM Session Worksheet.docx M. Executive Case Summary.docx N. Large-Group Lecture Schedule & Topic List.docx O. PPT 1 - Contributing to a Culture of Safety.pptx P. PPT 2 - Systems Improvement.pptx Q. PPT 3 - Impact of Students and Residents on QI.pptx R. PPT 4 - Presentation of Safety Case.pptx S. PPT 5 - Disclosing Medical Errors.pptx T. PPT 6 - Training for Resilience.pptx U. PPT 7 - Introduction to Improvement Plans.pptx V. Facilitator Postworkshop Survey.docx [file mep-16-10874-s001.zip › G. RCA AM Session Patient Background & EWS Info.docx]

**Patient Background Information and Information on Early Warning Score**

**Patient Background Information: Mary Thompson**

| **Vital Signs in the ED** | **Normal Range** | **EWS Points** |
| --- | --- | --- |
| Pulse 110 bpm | 60-100 bpm | 1 |
| Respiratory Rate 24 | 12-20 per minute | 2 |
| Oxygen saturation 94% on 5 liters NC (on 3L at home) | 96-100% | 3 |
| Total EWS |  | 6 |

| **Vital Signs on the floor** | **Normal Range** | **EWS Points** |
| --- | --- | --- |
| Pulse 125 bpm | 60-100 bpm | 2 |
| Respiratory Rate 25 | 12-20 per minute | 3 |
| Oxygen saturation 94% on 5 liters NC | 96-100% | 3 |
| Total EWS |  | 8 |

**COPD (Chronic Obstructive Pulmonary Disease)**

- Chronic inflammatory condition causes thickening of the airways in the lungs
- Emphysema and chronic bronchitis are two sub-types of COPD
- Tobacco use is a major risk factor for the development of COPD
- Patients suffering from COPD have trouble breathing and become short of breath with minimal activity
- Many COPD patients require constant supplemental oxygen to avoid feeling short of breath

**Treatment of COPD**

- Bronchodilators are short-acting and long-acting medications which help relax the smooth muscles around the airways
  - Anti-cholinergics and beta-agonist medications are both bronchodilators
  - Can be given via inhalers or nebulizers
- Corticosteroids reduce inflammation around the lung tissue and airways
  - Can be given via inhalers, as oral pills, or as IV infusion

**Causes of COPD exacerbations**

- Viral infections like influenza, adenovirus, rhinovirus
- Bacterial infections like bacterial pneumonia
- Congestive heart failure
- Pulmonary embolism

**The Early Warning Score, or EWS, is a scoring system designed to detect/screen for deteriorating patients early so they can be triaged appropriately and moved to higher level of care if needed.**

“Author owned”
