# Supplementary material for: Safety in Numbers: Successful Student-Approved Case-Based Interprofessional Safety Workshop Utilizing Simulated Real-Life Safety Cases
Source: MedEdPORTAL. 2020 Jan 31;16:10874. doi: 10.15766/mep_2374-8265.10874 (PMC7065299; doi:10.15766/mep_2374-8265.10874)
Supplement: Supplementary file 1 — A. Pre- & Postevent Surveys.docx B. IPE Safety Workshop Agenda.docx C. RCA AM Session Facilitator Guide.docx D. RCA AM Session Facilitator Annotated Case Time Line.docx E. RCA AM Session Student Case Time Line.docx F. RCA AM Session Interviewee Scripts.docx G. RCA AM Session Patient Background & EWS Info.docx H. RCA AM Session Media - Radiology.docx I. RCA AM Session Media - Oxygen Tanks.docx J. Corrective Action PM Session Facilitator Guide.docx K. Corrective Action PM Session Effectiveness Chart.docx L. Corrective Action PM Session Worksheet.docx M. Executive Case Summary.docx N. Large-Group Lecture Schedule & Topic List.docx O. PPT 1 - Contributing to a Culture of Safety.pptx P. PPT 2 - Systems Improvement.pptx Q. PPT 3 - Impact of Students and Residents on QI.pptx R. PPT 4 - Presentation of Safety Case.pptx S. PPT 5 - Disclosing Medical Errors.pptx T. PPT 6 - Training for Resilience.pptx U. PPT 7 - Introduction to Improvement Plans.pptx V. Facilitator Postworkshop Survey.docx [file mep-16-10874-s001.zip › I. RCA AM Session Media - Oxygen Tanks.docx]

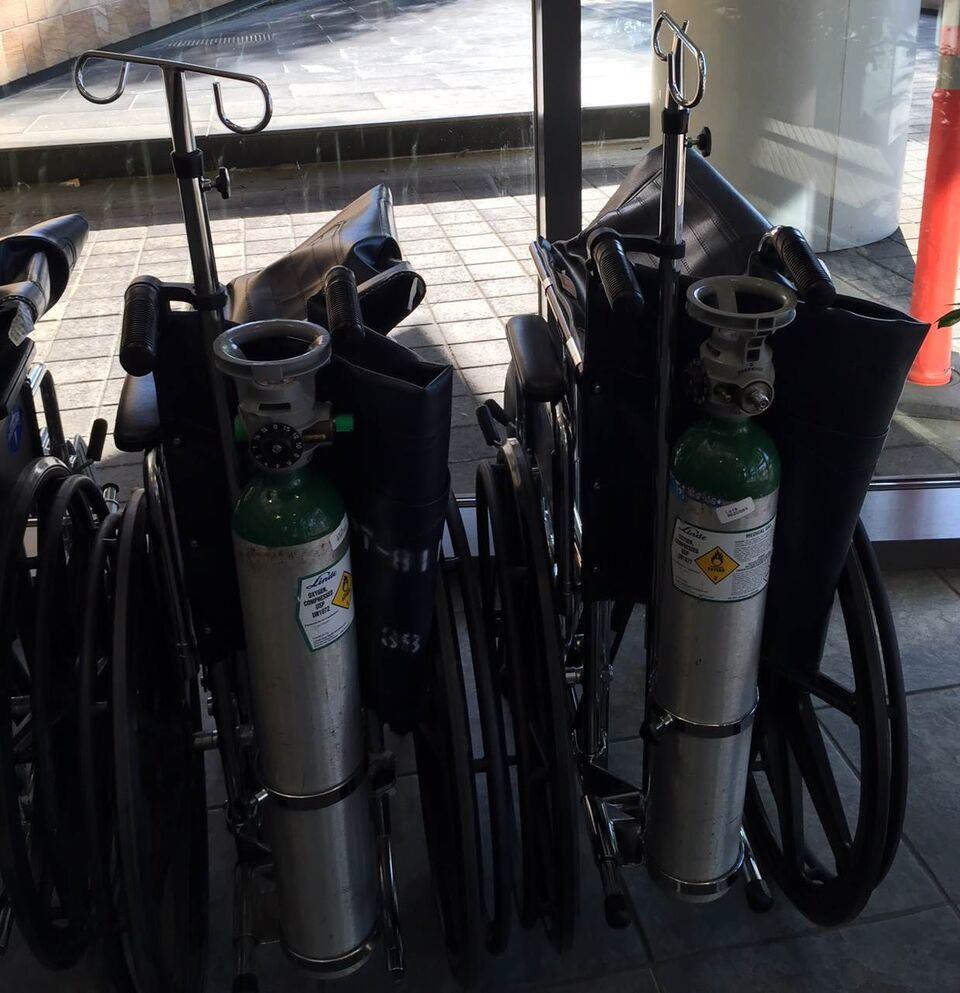


Wheelchairs in

transporter queue

(Author owned image)


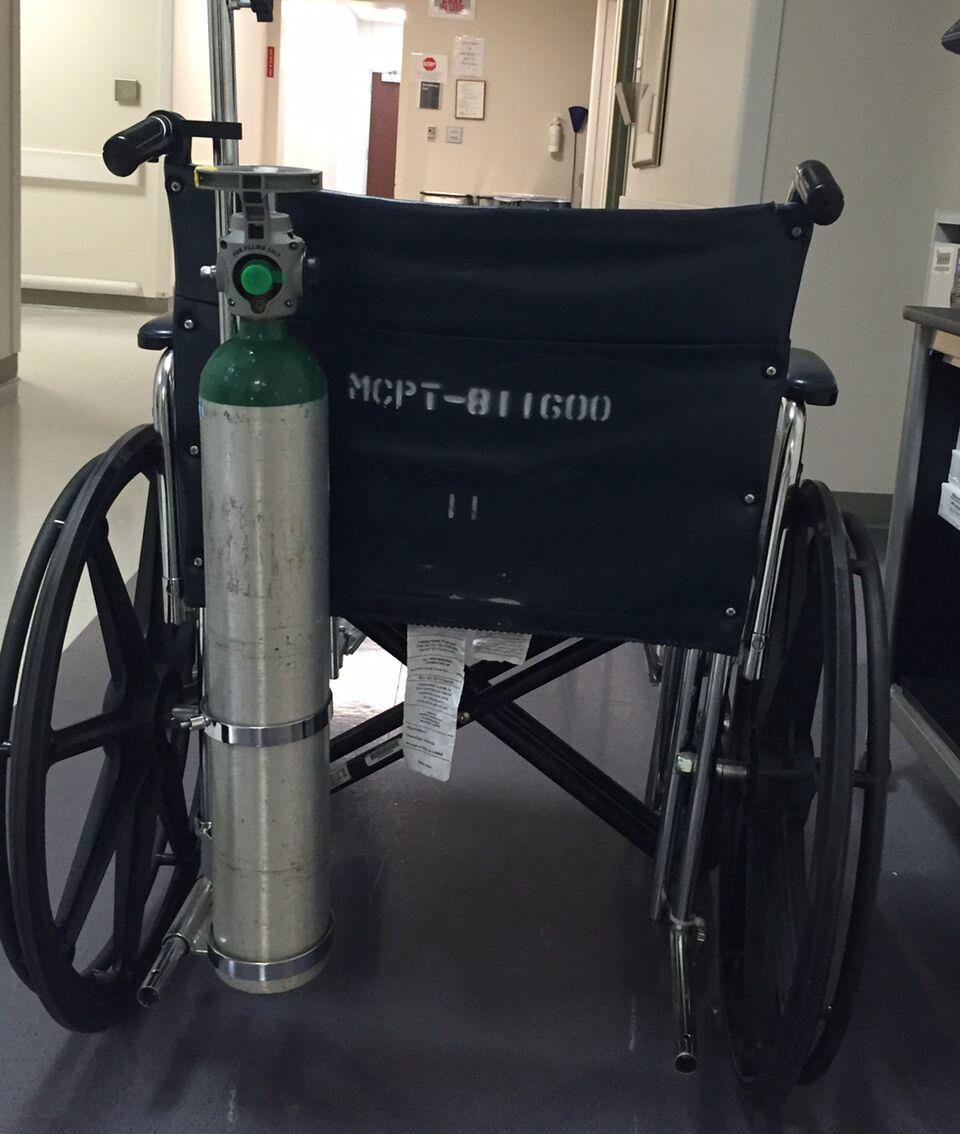


Wheelchair with portable

oxygen tank

(Author owned image)


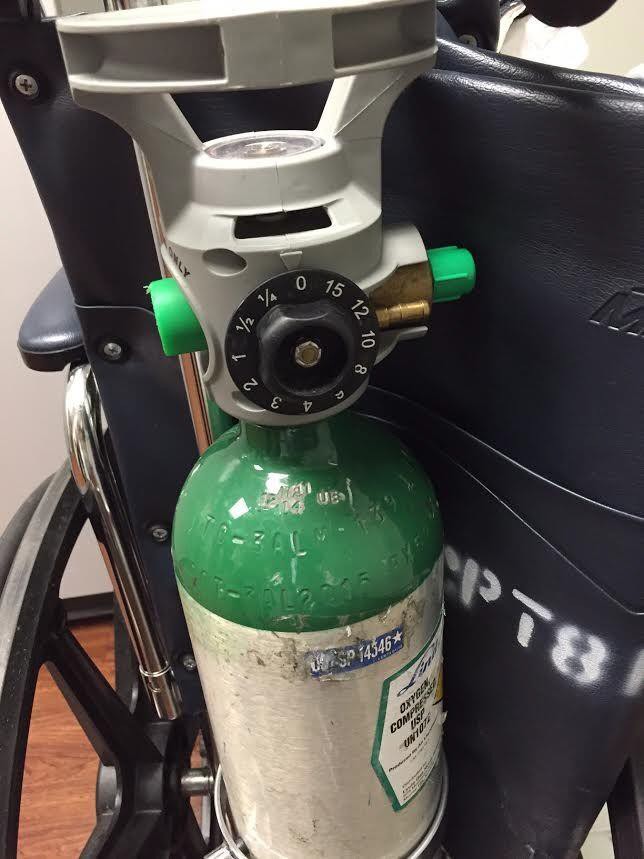


Portable oxygen tank oxygen flow

rate gauge (in liters/minute)

(Author owned image)

[*Please include image of group of oxygen tanks together*]

Representative image of oxygen

tank storage on the inpatient

floors.

Note that full and empty oxygen

tanks are stored together.


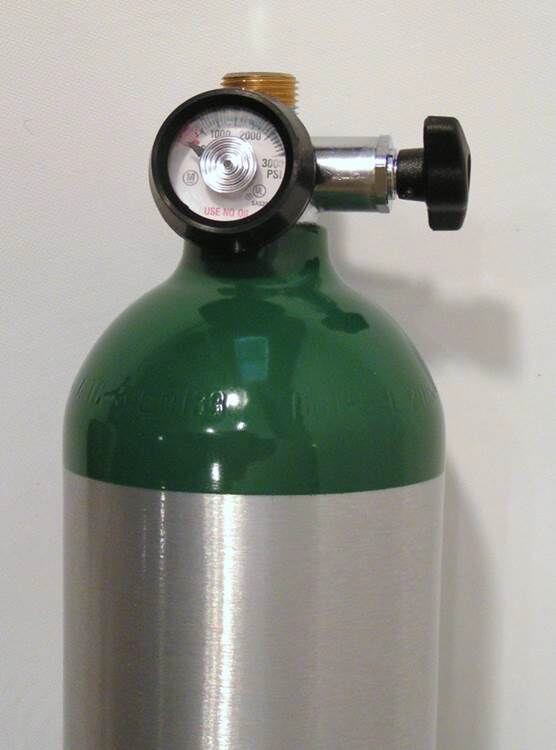


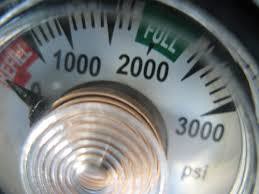


Portable oxygen tank pressure gauge

(in psi)

(Author owned image)

Interview: Respiratory Therapist

• Each day, in the evening, respiratory checks oxygen

tanks and any tanks under ½ full are sent to be refilled.

• One of the respiratory therapists checked the tanks the previous evening – unsure what time.

• Full and empty tanks are kept in same case.

– If a tank is below half early in the day, it would still be in that case.

• Not clear which tanks are full and empty without close

inspection

• The pressure gauge (reading how many PSI are left) is small – can be hard to notice.

**Portable Oxygen Tank Characteristics**

Pressure in oxygen tank

Oxygen tank flow in Liters per minute

|  | **1** | **2** | **3** | **4** | **5** |
| --- | --- | --- | --- | --- | --- |
| 2000 psi | 9 hrs | 4 hrs/30 min. | 3 hrs | 2 hrs | 1 hr/30 min. |
| 1500 psi | 7 hrs | 3 hrs | 2 hrs | 1 hr/30 min. | 1 hr |
| 1000 psi | 4 hrs/30 min. | 2 hrs | 1 hr/15 min. | 1 hr | 30 min |
| 500 psi | 2 hrs | 1 hr | 25 min | 15 min | 5 min |

Approximate length of time left in oxygen tank
