# Supplementary material for: Safety in Numbers: Successful Student-Approved Case-Based Interprofessional Safety Workshop Utilizing Simulated Real-Life Safety Cases
Source: MedEdPORTAL. 2020 Jan 31;16:10874. doi: 10.15766/mep_2374-8265.10874 (PMC7065299; doi:10.15766/mep_2374-8265.10874)
Supplement: Supplementary file 1 — A. Pre- & Postevent Surveys.docx B. IPE Safety Workshop Agenda.docx C. RCA AM Session Facilitator Guide.docx D. RCA AM Session Facilitator Annotated Case Time Line.docx E. RCA AM Session Student Case Time Line.docx F. RCA AM Session Interviewee Scripts.docx G. RCA AM Session Patient Background & EWS Info.docx H. RCA AM Session Media - Radiology.docx I. RCA AM Session Media - Oxygen Tanks.docx J. Corrective Action PM Session Facilitator Guide.docx K. Corrective Action PM Session Effectiveness Chart.docx L. Corrective Action PM Session Worksheet.docx M. Executive Case Summary.docx N. Large-Group Lecture Schedule & Topic List.docx O. PPT 1 - Contributing to a Culture of Safety.pptx P. PPT 2 - Systems Improvement.pptx Q. PPT 3 - Impact of Students and Residents on QI.pptx R. PPT 4 - Presentation of Safety Case.pptx S. PPT 5 - Disclosing Medical Errors.pptx T. PPT 6 - Training for Resilience.pptx U. PPT 7 - Introduction to Improvement Plans.pptx V. Facilitator Postworkshop Survey.docx [file mep-16-10874-s001.zip › J. Corrective Action PM Session Facilitator Guide.docx]

**Facilitator Guide – Mary Thompson Case**

Patient Safety and Systems Improvement Workshop

Improvement Plan Small Group Session

**Objectives:**

**Systems Improvement Workshop:**

This activity relates to the following Entrustable Professional Activities (EPAs) as outlined by the AAMC:

**EPA 9: Collaborate as a member of an interprofessional team.
EPA 13: Identify system failures and contribute to a culture of safety and improvement**

Additional Educational Objectives for Systems Improvement Workshop:

1. Describe opportunities to improve quality of care within the modern healthcare system
2. Describe the impact of continual quality improvement on our healthcare system
3. Engage current clinical faculty in the process of teaching safety and quality principles

*“We cannot change the human condition, but we can change the conditions under which humans work.”*

**Afternoon Breakout Session Objectives:**

- Use one identified root cause from the RCA to brainstorm ideas for corrective action.
- Classify potential corrective action ideas into appropriate system categories:
- (ie. manpower, machines, environment, processes)
- Anticipate potential pitfalls to success of a corrective action:
- Through preparing to explain your ideas to Executive and Patient Safety leaders

**Student participants:**Each group will have the same 12-13 students from the morning RCA breakout session

**Improvement Plan/Corrective Action Plan: Breakout Timeline (total 60 minutes):**

**Introduction**: *10 minutes*

- 1. Group Introductions (if new facilitator)
  2. Address any questions or comments from the students regarding content thus far
  3. Review the objectives for the afternoon breakout session
     - Identify potential corrective actions based on one of your team’s findings from the RCA (or a root cause provided)
     - Pick 1 or 2 corrective actions to discuss in detail:
       - *What has the most impact for amount of resources needed?*
     - Consider some barriers to successful implementation of the corrective action
       - *Anticipate the perspective of the Executive Leadership at wrap-up*
  4. Assign roles: Team Leader; Scribe

**Develop Corrective Action Plan:** 4*0 minutes*

**First Step:** Students select one (1) root cause to focus on for this time period – *5 min*

- - - Quick group vote is ok
    - Draw from the “master list” – nice if it’s also from the team’s own ideas from the morning Root Cause Analysis

**Second Step:** Generate a list of potential corrective actions – *10 min*

- - - Team members brainstorm a list of corrective actions. **No wrong answers here!!** This should be a time for “anything goes” to encourage outside the box thinking.
    - **Scribe** to document ideas *verbatim* on the board.
    - **If students get stuck, refer to handout /included charts: (Appendix K, L)**
      - **Consider different parts of the microsystem** *(see handout)*
      - **Chart on Stronger/Intermediate/Weaker Actions** *(see handout)*
      - **Facilitator’s “Cheat Sheet”** on corrective actions from this case *(p4)*

**Third Step: Facilitator, use two provided handouts** to help students identify: *10 min*

- - - Which components of the clinical microsystem are involved?
    - How “strong” is this systems fix? i.e., to what degree does it rely on humans to remember to perform a task correctly?
    - Is this a remedial corrective action, interim corrective action, or corrective action to prevent recurrence?
    - *The purpose here is to help them with “systems thinking” rather than individual blame – i.e., practice safety culture!*

**Fourth Step:** Select 1-2 actions that have the ***most impact*** for the ***resources required:*** *5 min*

- - - Will this action address the cause that resulted in the event?
    - Is it within management’s control to enact in a timely manner?
    - Will it cost less to implement than the cost associated with the event?
      - Consider cost, feasibility, staff buy-in, need for administrative support, need for new equipment, staff training, or process change

***Ideally the group will choose a corrective action to prevent recurrence.***

**Conclusion**: 1*0 minutes*

1. Choose a representative from the group (not the facilitator) to present the group’s corrective action plan in the closing group session to the Executive Sponsors
   **(see Information about the Final Session, below)**
2. Brainstorm any potential issues with the corrective action
3. Scribe should record main points on an index card for student presenter

**INFORMATION ABOUT THE FINAL SESSION: 3:30pm-4:00pm**

**For the final session, student teams will come together with a panel of Executive Leaders, Patient Safety and Process Improvement delegates.**

**The agenda will include:**

1. Introduction of the panel and a few words about Safety and Systems Improvement at this institution, including the importance of students to this mission;
2. Student teams can volunteer to present:
   1. Their chosen Root Cause
   2. Their proposed Corrective Action Plan(s)
3. The Executive Sponsor will discuss each proposed Correction Action Plan(s) with the student groups, providing feedback regarding the proposal’s feasibility, likelihood of achieving impact, cost, and staying power.
4. Student and Faculty Evaluations (paper/pen, to be completed before departure)

**Tips, Tricks and Troubleshooting:**

- Contributing factors to system-based errors include:
  - Complexity: too many steps, too many people involved
  - Too heavy or too light workload
  - Poor design
  - Interruptions and distractions
  - Culture: lack of responsibility, poor teamwork, poor communication
- Students may want to focus on re-training personnel as their corrective action. Encourage them to look at other areas of the clinical microsystem in this case.
  - Historically, personnel training tends to be the lowest yield for impacting major change
  - Higher yield strategies are located in the handout provided: **High/Intermediate/Low** include: mistake-proofing a process, controlling for error with active safety devices, provide warning devices for manual action, and procedures for reduction of error and control.

**CHEAT SHEET of Corrective Action Plans for the RCA**

Below is the list of corrective actions identified in the real-life case (“Mrs. Thompson”) we have discussed today.

The second column lists the “type” of corrective action and the third column lists the barriers to implementation/success that were encountered.

This sheet is provided to give facilitators more detailed content regarding the outcome of the case.

**This is not an “answer sheet!”** The student teams do NOT need to arrive at the same corrective actions and we do not want you to lead the group to these. Students may come up with novel, outside-the-box ideas – and that is great too!

| **Corrective Action** | **System category** | **Barriers to Success** |
| --- | --- | --- |
| Store empty and near-empty oxygen tanks separate from full tanks. | Methods, Machines | - Identify new location for empty tanks on each unit - Staff training |
| Check oxygen tanks in storage twice/day instead of once. | Manpower, Methods | - Change to current culture/workflow - Staff training |
| Creation of trip slips. Include oxygen tanks on slips | Methods, Mother Nature | - Buy in from nursing and transports - Staff training |
| Put more wall oxygen outlets in the radiology holding area | Machines, “Mother Nature” | - Cost of adding outlets - Support from engineering dept. |
| Enhance nursing coverage of radiology holding area (basically, RN covering holding area cannot be pulled to help with procedures elsewhere) | Manpower, Methods | - Cost of adding additional nurses - Change to current culture/workflow |
| Reinforce use of wall oxygen in CT and Ultrasound rooms | Methods, Machines | - New workflow to have staff check wall outlets - Training staff - Change to current culture |
| This is one of several events that led to mandated use of Early Warning Scores | Measurement, Methods | - Cost of advertising EWS - Hospital-wide buy in - Training all levels of health care providers |
