# Supplementary material for: Safety in Numbers: Successful Student-Approved Case-Based Interprofessional Safety Workshop Utilizing Simulated Real-Life Safety Cases
Source: MedEdPORTAL. 2020 Jan 31;16:10874. doi: 10.15766/mep_2374-8265.10874 (PMC7065299; doi:10.15766/mep_2374-8265.10874)
Supplement: Supplementary file 1 — A. Pre- & Postevent Surveys.docx B. IPE Safety Workshop Agenda.docx C. RCA AM Session Facilitator Guide.docx D. RCA AM Session Facilitator Annotated Case Time Line.docx E. RCA AM Session Student Case Time Line.docx F. RCA AM Session Interviewee Scripts.docx G. RCA AM Session Patient Background & EWS Info.docx H. RCA AM Session Media - Radiology.docx I. RCA AM Session Media - Oxygen Tanks.docx J. Corrective Action PM Session Facilitator Guide.docx K. Corrective Action PM Session Effectiveness Chart.docx L. Corrective Action PM Session Worksheet.docx M. Executive Case Summary.docx N. Large-Group Lecture Schedule & Topic List.docx O. PPT 1 - Contributing to a Culture of Safety.pptx P. PPT 2 - Systems Improvement.pptx Q. PPT 3 - Impact of Students and Residents on QI.pptx R. PPT 4 - Presentation of Safety Case.pptx S. PPT 5 - Disclosing Medical Errors.pptx T. PPT 6 - Training for Resilience.pptx U. PPT 7 - Introduction to Improvement Plans.pptx V. Facilitator Postworkshop Survey.docx [file mep-16-10874-s001.zip › K. Corrective Action PM Session Effectiveness Chart.docx]

***Effectiveness of Error Prevention Strategies:***

*This chart provides examples of weak, intermediate, and strong corrective actions to reduce healthcare safety events. These can serve as inspiration for students during the afternoon corrective action breakout session.*

|  | ***Action Category*** | ***Example*** |
| --- | --- | --- |
| **Weaker Actions: These tasks require more reliance on humans to remember to perform the task correctly** | **Double checks** | One person calculates dosage, another person reviews their calculation. |
|  | **Warnings** | Add audible alarms or caution labels. |
|  | **New procedure/ memorandum/policy** | Remember to check IV sites every 2 hours. |
|  | **Training** | Demonstrate correct usage of hard-to-use medical equipment. |

|  | **Action Category** | **Example** |
| --- | --- | --- |
| **Intermediate Actions** | **Redundancy** | Use two RNs to independently calculate high-risk medication dosages. |
|  | **Increase in staffing/decrease in workload** | Make float staff available to assist when workloads peak during the day. |
|  | **Software enhancements, modifications** | Use computer alerts for drug-drug interactions. |
|  | **Eliminate/reduce distractions** | Provide quiet rooms for programming PCA pumps; remove distractions for nurses when programming medication pumps. |
|  | **Education using simulation based training, with periodic refresher sessions and observations** | Conduct patient handoffs in a simulation lab/environment, with after action critiques and debriefing. |
|  | **Checklist/cognitive aids** | Use pre-induction and pre-incision checklists in operating rooms, as well as a “time out” before all procedures to verify the correct patient, site and procedure being performed. Use a checklist when reprocessing flexible fiber optic endoscopes. |
|  | **Eliminate look- and sound-alikes** | Do not store look-alikes next to one another in the unit medication room. |
|  | **Standardized communication tools** | Use read-back for all critical lab values. Use read-back or repeat-back for all verbal medication orders. Use a standardized patient handoff format. |
|  | **Enhanced documentation, communication** | Highlight medication name and dose on IV bags. Verify right patient, right medication, right dosage, right route. |

|  | **Action Category** | **Example** |
| --- | --- | --- |
| **Stronger Actions: These tasks require less reliance on humans to remember to perform the task correctly** | **Architectural/physical plant changes** | Replace revolving doors at the main patient entrance into the building with powered sliding or swinging doors to reduce patient falls. |
|  | **New devices with usability testing** | Use computer software to test outpatient blood glucose meters and test strips and select the most appropriate for the patient population being served. |
|  | **Engineering control (forcing function)** | Eliminate the use of universal adaptors and peripheral devices for medical equipment and use tubing/fittings that can only be connected the correct way (e.g., IV tubing and connectors that cannot physically be connected to sequential compression devices (SCDs)). |
|  | **Simplify process** | Remove unnecessary steps in a process. |
|  | **Standardize on equipment or process** | Standardize on the make and model of medication pumps used throughout the institution. Use bar coding for medication administration. |
|  | **Tangible involvement by leadership** | Participate in unit patient safety evaluations and interact with staff; support the RCA process; purchase needed equipment; ensure staffing and workload are balanced. |
