# Supplementary material for: Safety in Numbers: Successful Student-Approved Case-Based Interprofessional Safety Workshop Utilizing Simulated Real-Life Safety Cases
Source: MedEdPORTAL. 2020 Jan 31;16:10874. doi: 10.15766/mep_2374-8265.10874 (PMC7065299; doi:10.15766/mep_2374-8265.10874)
Supplement: Supplementary file 1 — A. Pre- & Postevent Surveys.docx B. IPE Safety Workshop Agenda.docx C. RCA AM Session Facilitator Guide.docx D. RCA AM Session Facilitator Annotated Case Time Line.docx E. RCA AM Session Student Case Time Line.docx F. RCA AM Session Interviewee Scripts.docx G. RCA AM Session Patient Background & EWS Info.docx H. RCA AM Session Media - Radiology.docx I. RCA AM Session Media - Oxygen Tanks.docx J. Corrective Action PM Session Facilitator Guide.docx K. Corrective Action PM Session Effectiveness Chart.docx L. Corrective Action PM Session Worksheet.docx M. Executive Case Summary.docx N. Large-Group Lecture Schedule & Topic List.docx O. PPT 1 - Contributing to a Culture of Safety.pptx P. PPT 2 - Systems Improvement.pptx Q. PPT 3 - Impact of Students and Residents on QI.pptx R. PPT 4 - Presentation of Safety Case.pptx S. PPT 5 - Disclosing Medical Errors.pptx T. PPT 6 - Training for Resilience.pptx U. PPT 7 - Introduction to Improvement Plans.pptx V. Facilitator Postworkshop Survey.docx [file mep-16-10874-s001.zip › L. Corrective Action PM Session Worksheet.docx]

**Corrective Action Worksheet for Afternoon Breakout Sessions**

*As your group brainstorms ways to correct the system errors identified in Mrs. Thompson’s Root Cause Analysis (RCA), consider each potential solution as addressing one of the following categories of error:*

1. **Manpower** 🡪 the people involved in the patient’s care
2. **Materials** 🡪 supplies used in the care of Mrs. Thompson
3. **Methods** 🡪 the policies and procedures involved in the care of Mrs. Thompson
4. **Machines** 🡪 equipment used in patient care
5. **Mother** **Nature** 🡪 the environment surrounding Mrs. Thompson’s care
6. **Measurement** 🡪 information used in patient care

These are often referred to as the **6M approach**.

*When reflecting on the case of Mrs. Thompson, you may come up with several different corrective actions. Try to determine the type of corrective actions using the following parameters:*

1. **Remedial corrective action**
   1. Remedial corrective actions are taken immediately after the event occurs to restore conditions to a safe and acceptable level.
   2. Remedial actions are typically broke/fix type actions that can be implemented independent of the cause.
2. **Interim corrective action**
   1. Interim corrective actions are actions taken in the short-term before the root cause of the event is known.
   2. Interim corrective actions mitigate the consequences of inappropriate acts or defective systems.
3. **Corrective Action to Prevent Recurrence (CATPR)**
   1. A fix that addresses the root cause of the event and as a result prevents future events
   2. An accurately defined and well-implemented CATPR should replace the continued need for the temporary interim corrective actions.
