# Supplementary material for: Safety in Numbers: Successful Student-Approved Case-Based Interprofessional Safety Workshop Utilizing Simulated Real-Life Safety Cases
Source: MedEdPORTAL. 2020 Jan 31;16:10874. doi: 10.15766/mep_2374-8265.10874 (PMC7065299; doi:10.15766/mep_2374-8265.10874)
Supplement: Supplementary file 1 — A. Pre- & Postevent Surveys.docx B. IPE Safety Workshop Agenda.docx C. RCA AM Session Facilitator Guide.docx D. RCA AM Session Facilitator Annotated Case Time Line.docx E. RCA AM Session Student Case Time Line.docx F. RCA AM Session Interviewee Scripts.docx G. RCA AM Session Patient Background & EWS Info.docx H. RCA AM Session Media - Radiology.docx I. RCA AM Session Media - Oxygen Tanks.docx J. Corrective Action PM Session Facilitator Guide.docx K. Corrective Action PM Session Effectiveness Chart.docx L. Corrective Action PM Session Worksheet.docx M. Executive Case Summary.docx N. Large-Group Lecture Schedule & Topic List.docx O. PPT 1 - Contributing to a Culture of Safety.pptx P. PPT 2 - Systems Improvement.pptx Q. PPT 3 - Impact of Students and Residents on QI.pptx R. PPT 4 - Presentation of Safety Case.pptx S. PPT 5 - Disclosing Medical Errors.pptx T. PPT 6 - Training for Resilience.pptx U. PPT 7 - Introduction to Improvement Plans.pptx V. Facilitator Postworkshop Survey.docx [file mep-16-10874-s001.zip › M. Executive Case Summary.docx]

**Case Overview – Mary Thompson**

**This is a real case from Wake Forest School of Medicine that inspired several safety measures, including use of “Trip Slips” and changes to the radiology suite.**

**Brief Case Description:** The patient is a 60 year old woman with severe COPD on 3L NC at home. She presented with a COPD exacerbation that did not respond to antibiotics, steroids, and nebulizers. Her medical team appropriately considered pulmonary embolism in the differential diagnosis and ordered a CT scan – PE protocol.

**Sequence of Events**:

Her VS worsened on hospital day #2 – in today’s classification, Early Warning Score (EWS) of 8 – but since that classification was not routine at the time, she did not travel monitored. Transporter brought her via wheelchair to radiology with an oxygen tank; It is unclear how full her oxygen tank was (full and empty tanks stored together in past).

In the radiology holding area, she was not transferred to wall O2 (not enough outlets). She was not transferred to wall O2 in the CT scanner (O2 flow gauge missing). All outlets were again full in the radiology holding area after the CT. She became “sleepy” in the holding area per the radiology tech and transporter. Since the radiology nurse was pulled for a procedure, she wasn’t assessed clinically in radiology holding area.

The transporter returned her to her nursing unit. Her nurse was in another patient room doing a procedure. The transporter left Mrs. Thompson in her room in her wheelchair after notifying nurse. The nurse found the patient apneic and unresponsive. A Code Blue was called. The patient died. The cause of death was determined to be hypoxemia. Oxygen tank on her wheelchair was found to be empty.

**Potential root causes:**

- Failure of treatment team and nursing to identify worsening vital signs from when she was admitted
- Sending patient with abnormal vitals off unit without monitor or nurse
- Oxygen tank was almost certainly ¼ full or less, based on time sequence and flow rate of oxygen
  - Full and empty tanks stored together
  - Tanks only checked once daily
  - Gauge on tanks small, difficult to read
  - Nobody assigned to check how full the tanks are before use
- No handoff of care between nurses. This is especially important in patient with unstable vital signs.
- Long wait in holding area before and after CT study
- Missing nurse in holding area because she was pulled to help with procedure. This was a routine practice but there was no rule against this.
- No vital signs routinely checked in radiology holding area
- Inadequate number of oxygen outlets in holding area. It became a routine practice to leave patients on oxygen tanks
- Missing oxygen flow rate gauge in CT room. Gauges are removable and there were not enough in the radiology area. So they were taken from one room to another.
- Techs decided to leave patient on tank. This had become a routine practice.
- On return to holding area, all problems identified above had recurred.
- Delay from time patient returned to room until nurse went to check on patient

This case illustrates the “Swiss Cheese” model of major safety events, where multiple barriers to prevent harm failed to protect the patient that day and aligned to result in a serious adverse patient outcome.
