# Supplementary material for: Safety in Numbers: Successful Student-Approved Case-Based Interprofessional Safety Workshop Utilizing Simulated Real-Life Safety Cases
Source: MedEdPORTAL. 2020 Jan 31;16:10874. doi: 10.15766/mep_2374-8265.10874 (PMC7065299; doi:10.15766/mep_2374-8265.10874)
Supplement: Supplementary file 1 — A. Pre- & Postevent Surveys.docx B. IPE Safety Workshop Agenda.docx C. RCA AM Session Facilitator Guide.docx D. RCA AM Session Facilitator Annotated Case Time Line.docx E. RCA AM Session Student Case Time Line.docx F. RCA AM Session Interviewee Scripts.docx G. RCA AM Session Patient Background & EWS Info.docx H. RCA AM Session Media - Radiology.docx I. RCA AM Session Media - Oxygen Tanks.docx J. Corrective Action PM Session Facilitator Guide.docx K. Corrective Action PM Session Effectiveness Chart.docx L. Corrective Action PM Session Worksheet.docx M. Executive Case Summary.docx N. Large-Group Lecture Schedule & Topic List.docx O. PPT 1 - Contributing to a Culture of Safety.pptx P. PPT 2 - Systems Improvement.pptx Q. PPT 3 - Impact of Students and Residents on QI.pptx R. PPT 4 - Presentation of Safety Case.pptx S. PPT 5 - Disclosing Medical Errors.pptx T. PPT 6 - Training for Resilience.pptx U. PPT 7 - Introduction to Improvement Plans.pptx V. Facilitator Postworkshop Survey.docx [file mep-16-10874-s001.zip › N. Large-Group Lecture Schedule & Topic List.docx]

**Large Group Lecture Schedule and Topic List**

Morning Large Group Session (90 minutes):

Educational Objectives Met:

1. Identify the strengths of an interprofessional healthcare delivery model
2. Apply systems knowledge to perform a root cause analysis on a real safety event
3. Understand the impact of continual quality improvement on our healthcare system

- **Contributing to a culture of safety**
  - Presented by Institutional Director of Patient Safety
  - *Discussed basic concepts of patient safety, systems errors and how errors are identified and studied*
- **Systems Improvement: Safety Reporting and Root Cause Analysis (RCA)**
  - Presented by a senior surgical resident who is involved in RCA process at our institution
  - *Gave examples of safety error and how to submit an incident report, as well as process of an RCA and who is involved*
- **Impact of Students and Residents on Quality Improvement**
  - Co-Presented by a medical student from our Institute for Healthcare Improvement (IHI) interest group and a resident representative from our Institutional Residency Council
  - *Provided examples of how student and resident reporting of safety events led to systems improvement*
- **Presentation of the day’s Safety Case**
  - Presented by our Medical Director of Risk Management
  - *Brief description of the case and charge to the small groups*

Afternoon Large Group Session (90 minutes):

Educational Objectives Met:

1. Identify the strengths of an interprofessional healthcare delivery model
2. Recognize opportunities to improve quality of care within the modern healthcare system
3. Understand the impact of continual quality improvement on our healthcare system

- **Disclosing Medical Errors**
  - Presented by Institutional Director of Patient Safety
  - *Discussed proper process for disclosing errors and who should be doing the disclosure*
- **Training for Resilience**
  - Presented by Institutional Director of Student Wellness
  - *Provided strategies for recognizing signs of second-victim experience and resources available to students and providers who are part of a safety event*
- **Introduction to Improvement Plans**
  - Presented by a Senior Performance Improvement Advisor
  - *Introduced concepts of improvement plans and corrective actions to prevent reoccurrence, as well as emphasizing focus on systems change*
