# Supplementary material for: Safety in Numbers: Successful Student-Approved Case-Based Interprofessional Safety Workshop Utilizing Simulated Real-Life Safety Cases
Source: MedEdPORTAL. 2020 Jan 31;16:10874. doi: 10.15766/mep_2374-8265.10874 (PMC7065299; doi:10.15766/mep_2374-8265.10874)
Supplement: Supplementary file 1 — A. Pre- & Postevent Surveys.docx B. IPE Safety Workshop Agenda.docx C. RCA AM Session Facilitator Guide.docx D. RCA AM Session Facilitator Annotated Case Time Line.docx E. RCA AM Session Student Case Time Line.docx F. RCA AM Session Interviewee Scripts.docx G. RCA AM Session Patient Background & EWS Info.docx H. RCA AM Session Media - Radiology.docx I. RCA AM Session Media - Oxygen Tanks.docx J. Corrective Action PM Session Facilitator Guide.docx K. Corrective Action PM Session Effectiveness Chart.docx L. Corrective Action PM Session Worksheet.docx M. Executive Case Summary.docx N. Large-Group Lecture Schedule & Topic List.docx O. PPT 1 - Contributing to a Culture of Safety.pptx P. PPT 2 - Systems Improvement.pptx Q. PPT 3 - Impact of Students and Residents on QI.pptx R. PPT 4 - Presentation of Safety Case.pptx S. PPT 5 - Disclosing Medical Errors.pptx T. PPT 6 - Training for Resilience.pptx U. PPT 7 - Introduction to Improvement Plans.pptx V. Facilitator Postworkshop Survey.docx [file mep-16-10874-s001.zip › O. PPT 1 - Contributing to a Culture of Safety.pptx]

## Slide 1
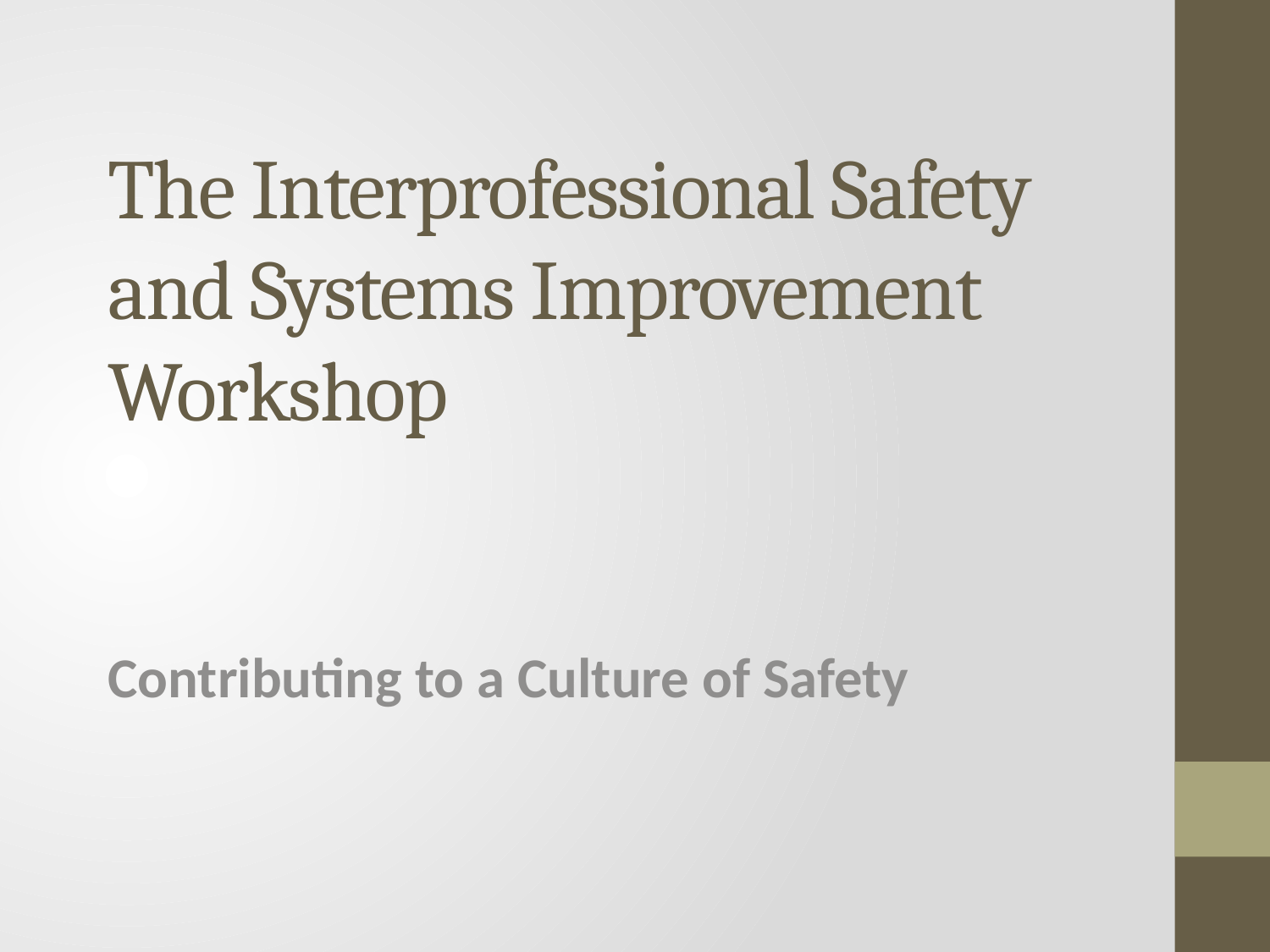

# The Interprofessional Safety and Systems Improvement Workshop
Contributing to a Culture of Safety

## Slide 2
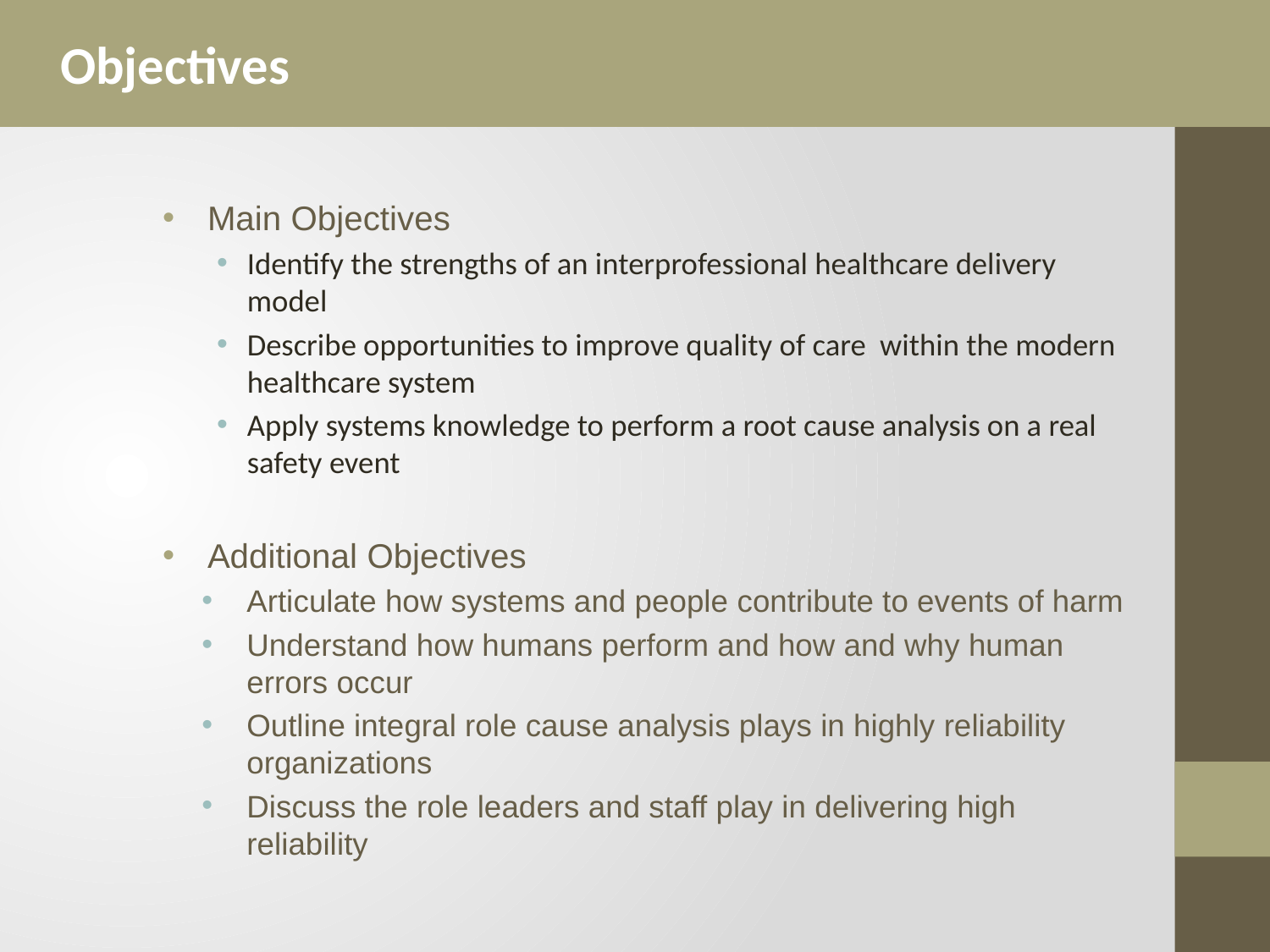

Objectives
Main Objectives
Identify the strengths of an interprofessional healthcare delivery model
Describe opportunities to improve quality of care within the modern healthcare system
Apply systems knowledge to perform a root cause analysis on a real safety event
Additional Objectives
Articulate how systems and people contribute to events of harm
Understand how humans perform and how and why human errors occur
Outline integral role cause analysis plays in highly reliability organizations
Discuss the role leaders and staff play in delivering high reliability

## Slide 3
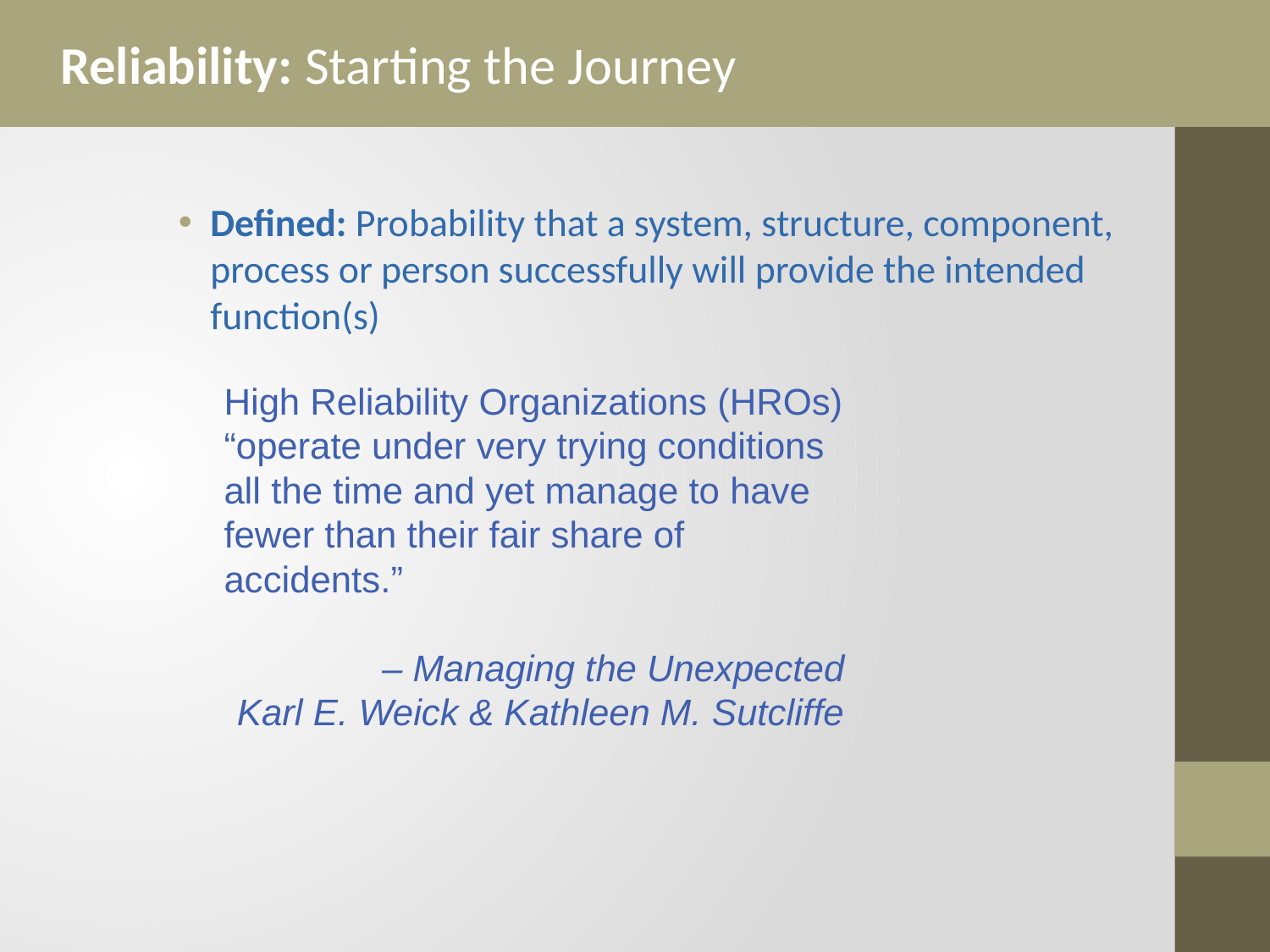

Reliability: Starting the Journey
Defined: Probability that a system, structure, component, process or person successfully will provide the intended function(s)
High Reliability Organizations (HROs) “operate under very trying conditions all the time and yet manage to have fewer than their fair share of accidents.”
– Managing the Unexpected
Karl E. Weick & Kathleen M. Sutcliffe

## Slide 4
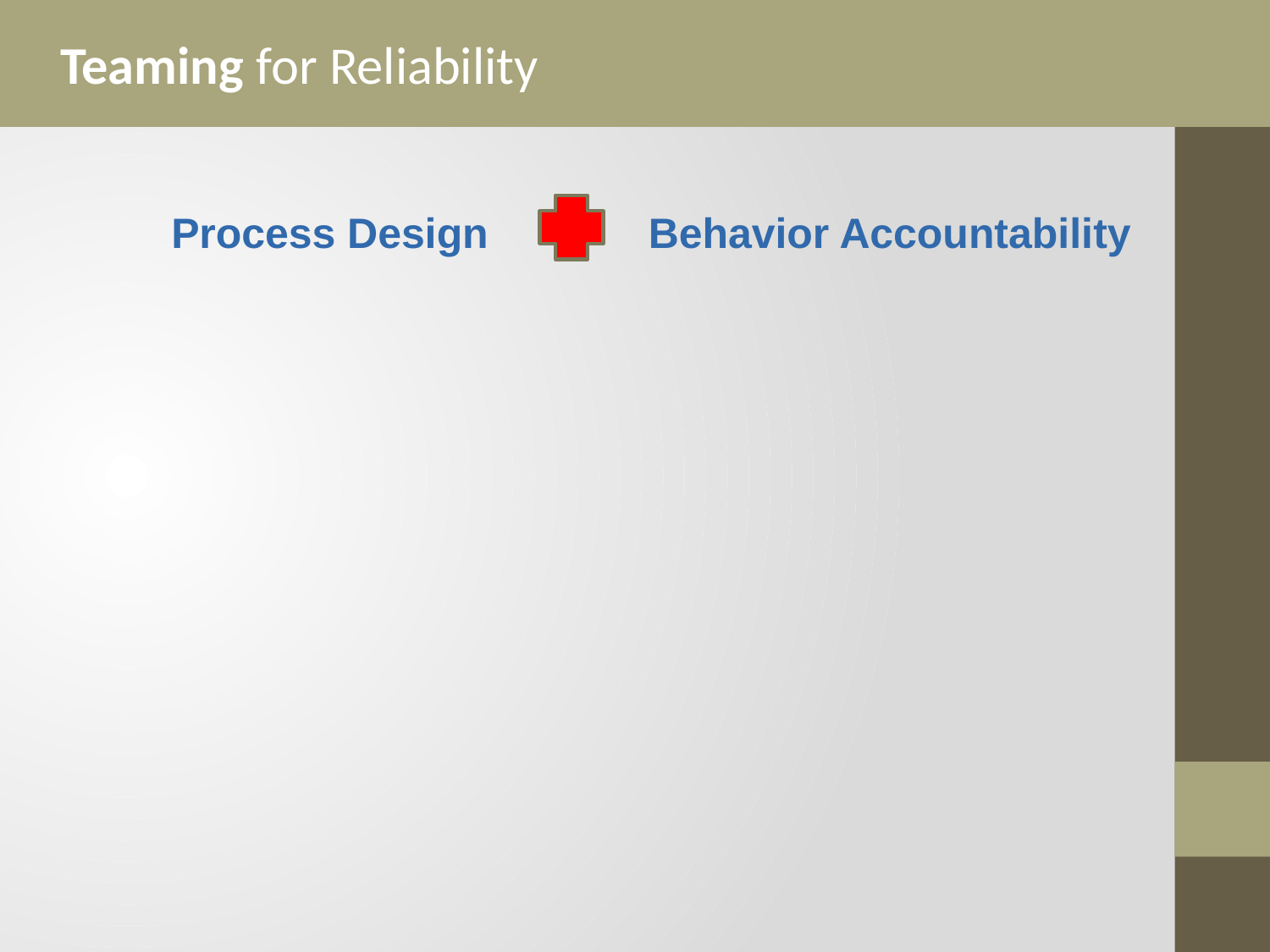

Teaming for Reliability
Process Design
Behavior Accountability

## Slide 5
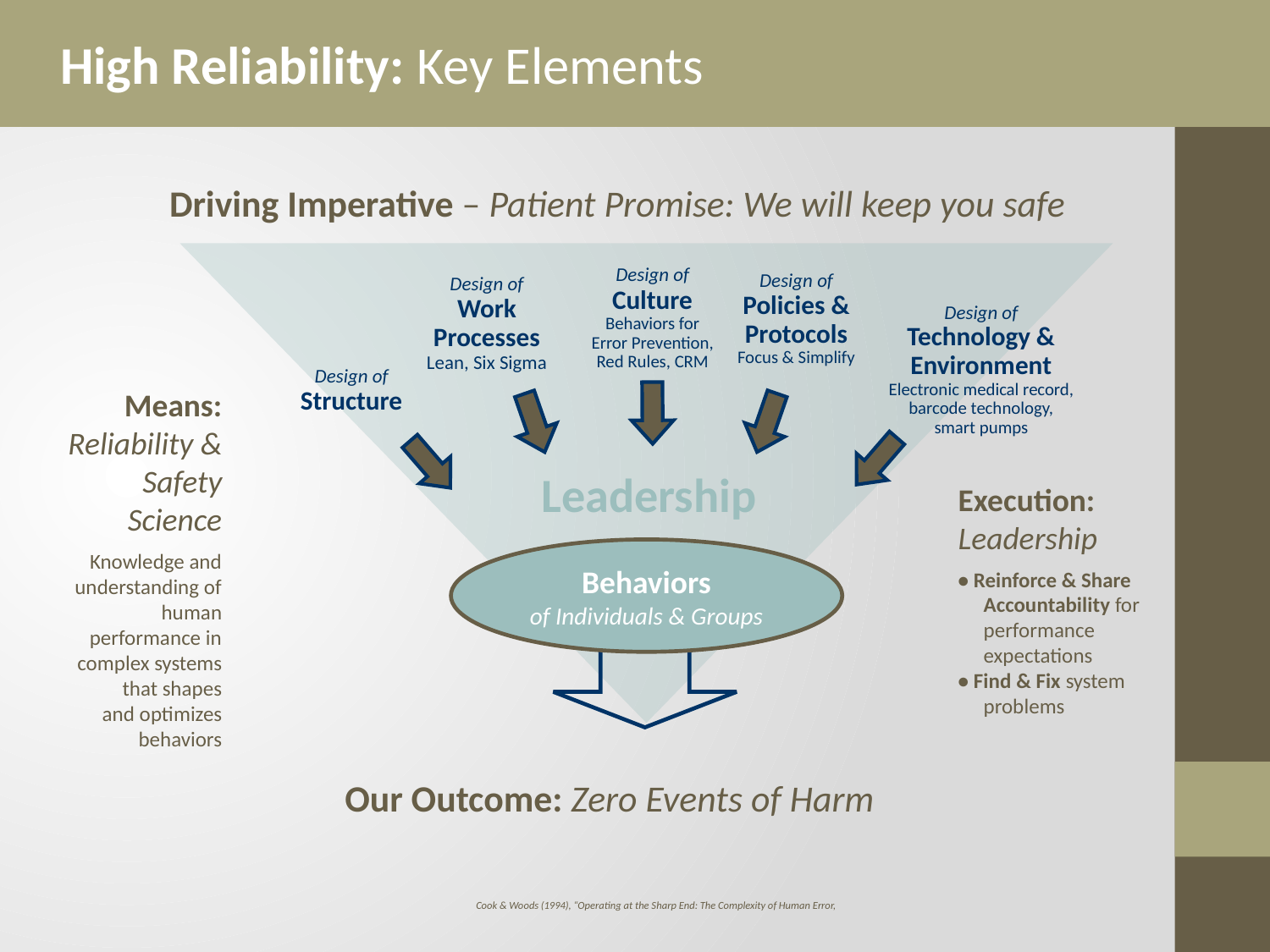

High Reliability: Key Elements
Driving Imperative – Patient Promise: We will keep you safe
Design of
Culture
Behaviors for
Error Prevention,
Red Rules, CRM
Design of
Policies &
Protocols
Focus & Simplify
Design of
Work
Processes
Lean, Six Sigma
Design of
Technology & Environment
Electronic medical record,
barcode technology,
smart pumps
Design of
Structure
Means:
Reliability &
Safety Science
Knowledge and understanding of human performance in complex systems that shapes
and optimizes behaviors
Leadership
Execution:
Leadership
• Reinforce & Share Accountability for performance expectations
• Find & Fix system problems
Behaviors
of Individuals & Groups
Our Outcome: Zero Events of Harm
Cook & Woods (1994), “Operating at the Sharp End: The Complexity of Human Error,

## Slide 6
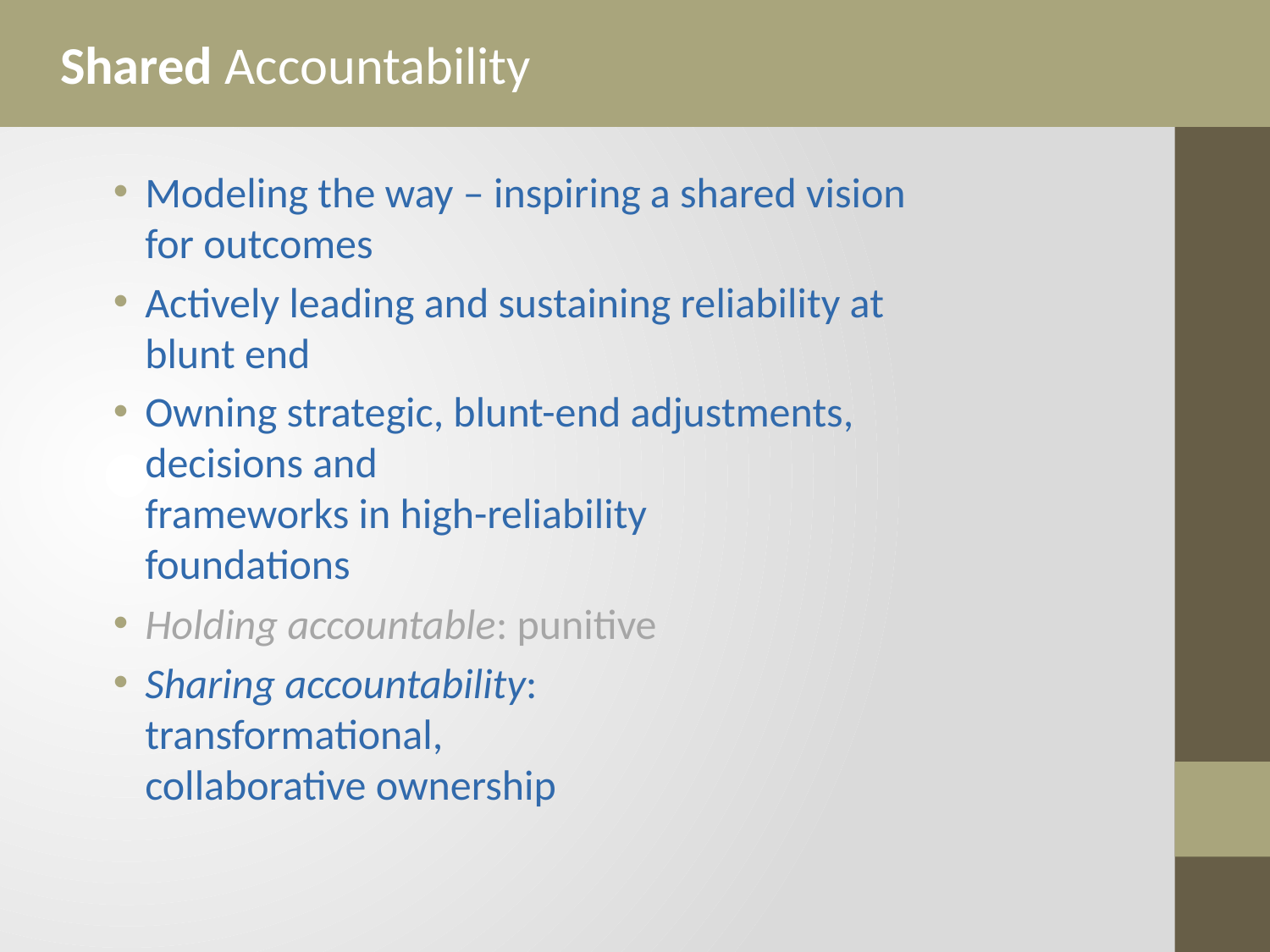

Shared Accountability
Modeling the way – inspiring a shared vision for outcomes
Actively leading and sustaining reliability at blunt end
Owning strategic, blunt-end adjustments, decisions and
frameworks in high-reliability
foundations
Holding accountable: punitive
Sharing accountability:
transformational,
collaborative ownership

## Slide 7
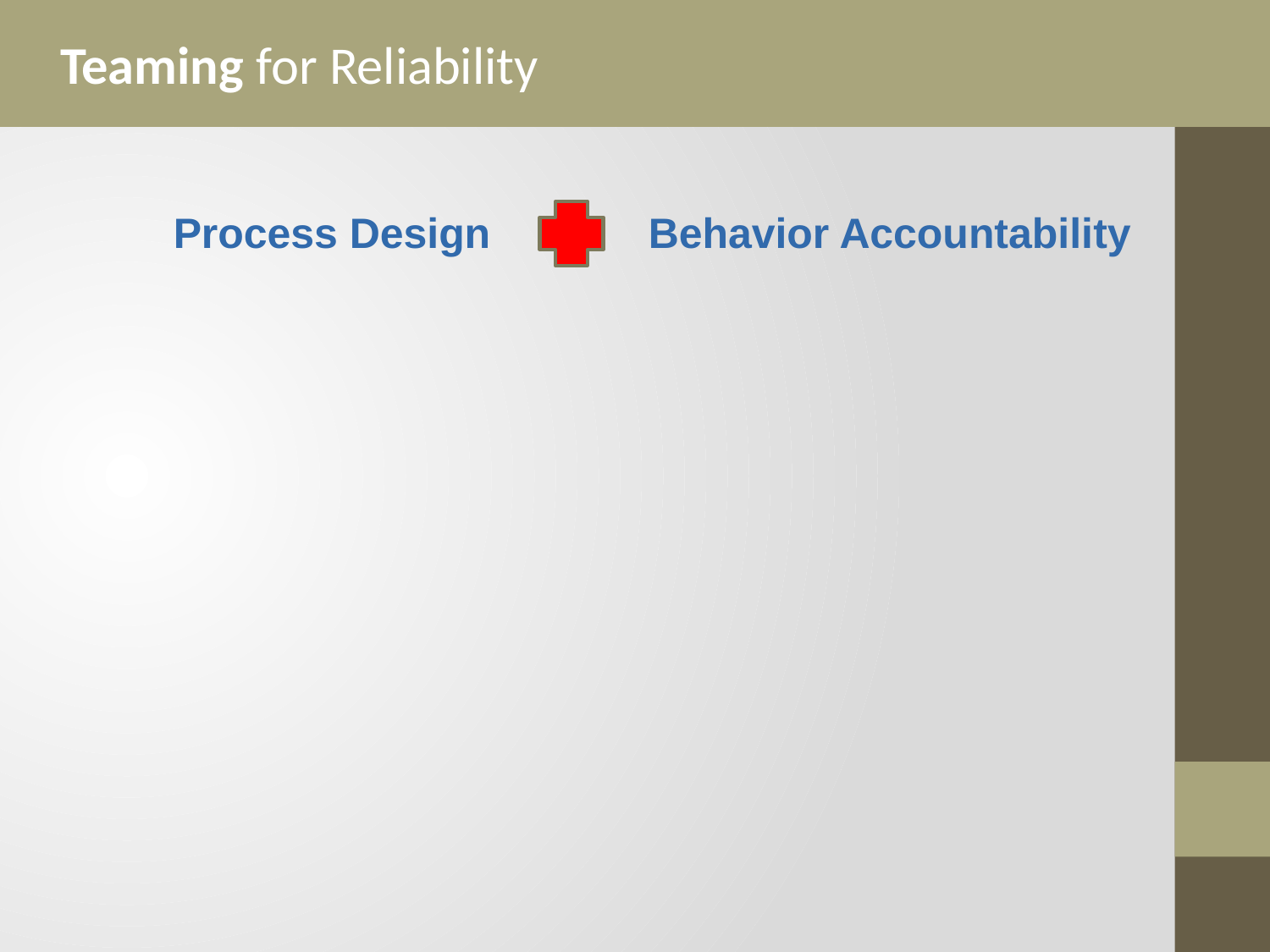

Teaming for Reliability
Process Design
Behavior Accountability

## Slide 8
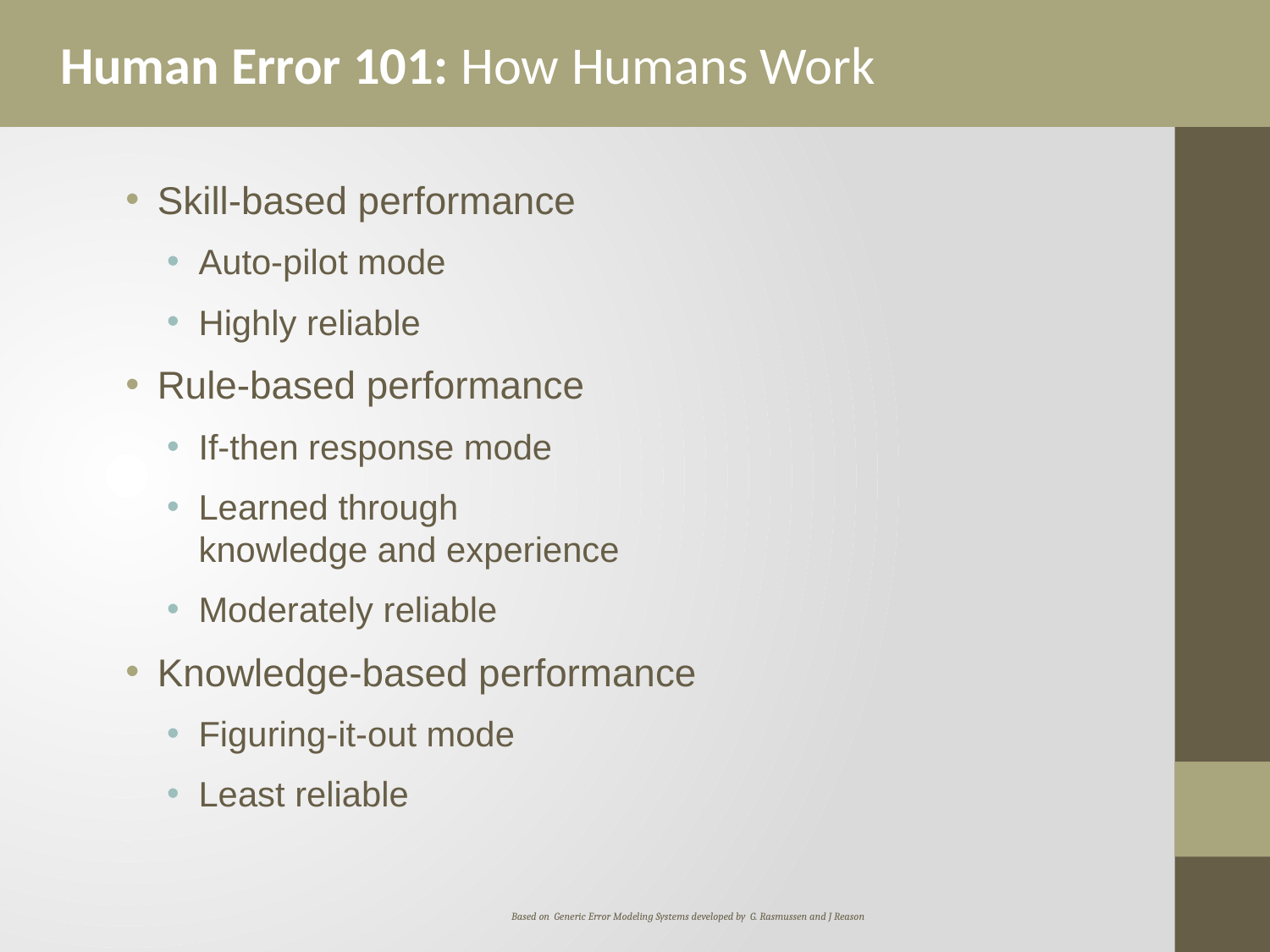

Human Error 101: How Humans Work
Skill-based performance
Auto-pilot mode
Highly reliable
Rule-based performance
If-then response mode
Learned through
knowledge and experience
Moderately reliable
Knowledge-based performance
Figuring-it-out mode
Least reliable
Based on Generic Error Modeling Systems developed by G. Rasmussen and J Reason

## Slide 9
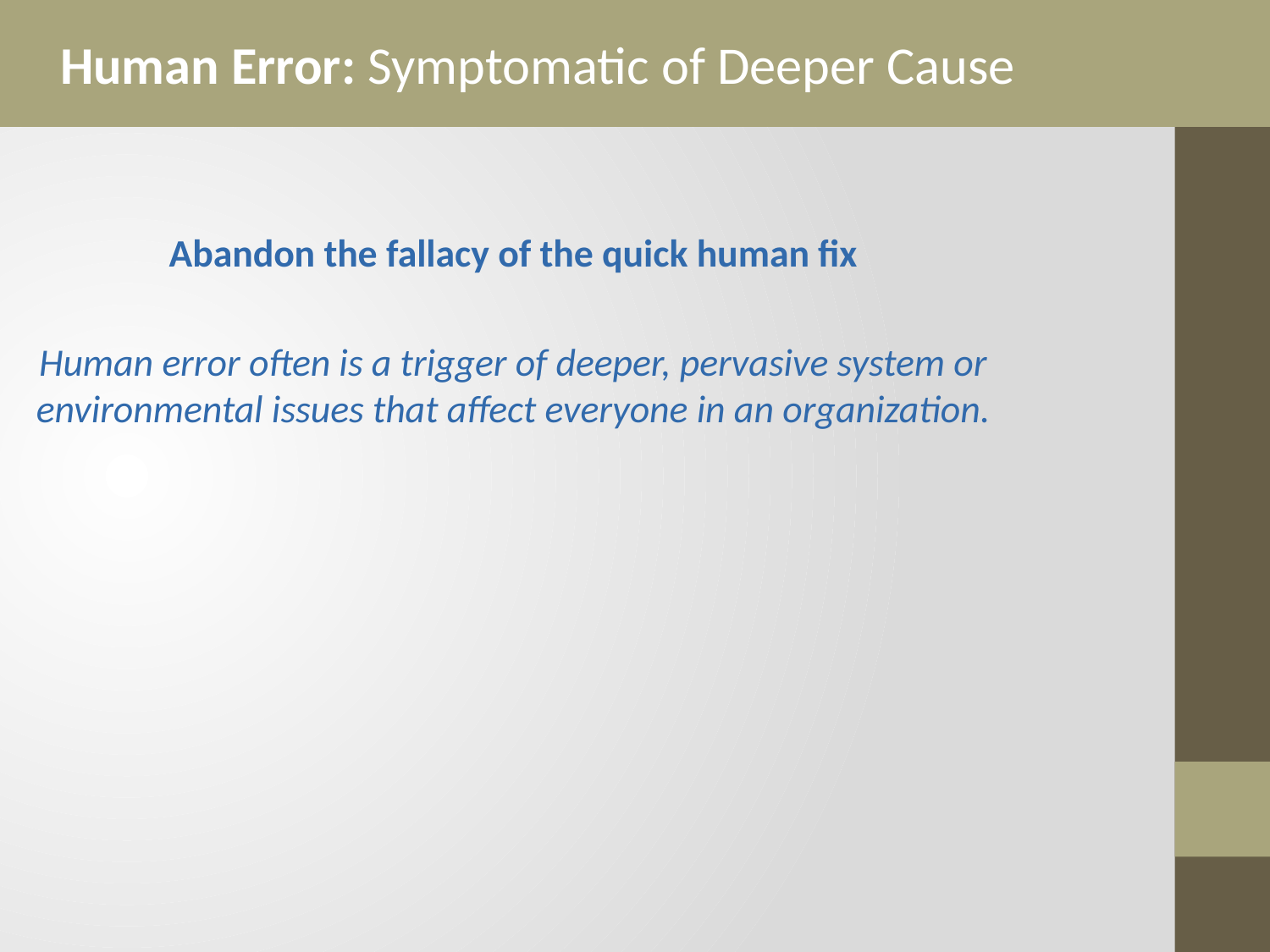

Human Error: Symptomatic of Deeper Cause
Abandon the fallacy of the quick human fix
Human error often is a trigger of deeper, pervasive system or environmental issues that affect everyone in an organization.

## Slide 10
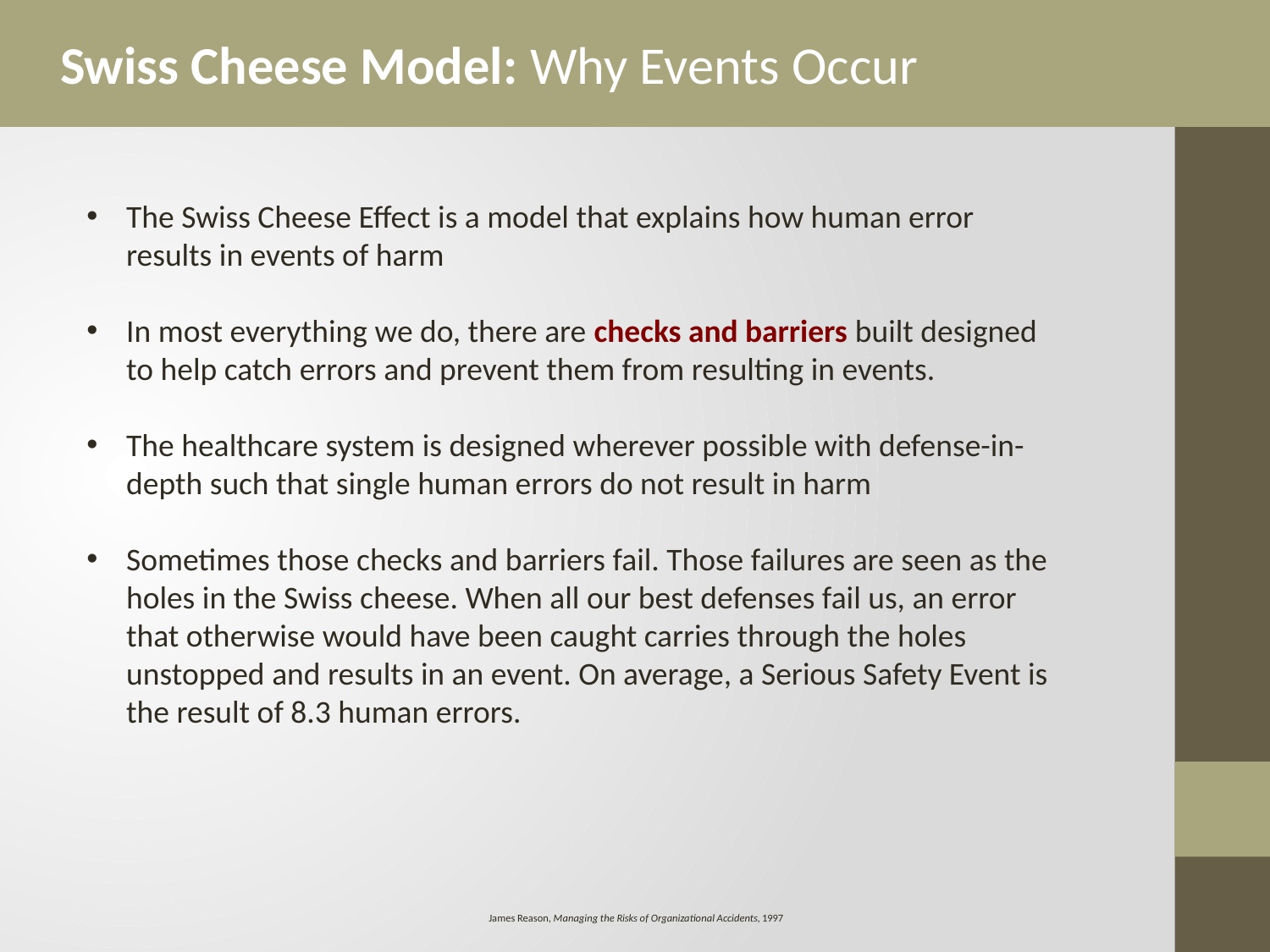

Swiss Cheese Model: Why Events Occur
The Swiss Cheese Effect is a model that explains how human error results in events of harm
In most everything we do, there are checks and barriers built designed to help catch errors and prevent them from resulting in events.
The healthcare system is designed wherever possible with defense-in-depth such that single human errors do not result in harm
Sometimes those checks and barriers fail. Those failures are seen as the holes in the Swiss cheese. When all our best defenses fail us, an error that otherwise would have been caught carries through the holes unstopped and results in an event. On average, a Serious Safety Event is the result of 8.3 human errors.
James Reason, Managing the Risks of Organizational Accidents, 1997

## Slide 11
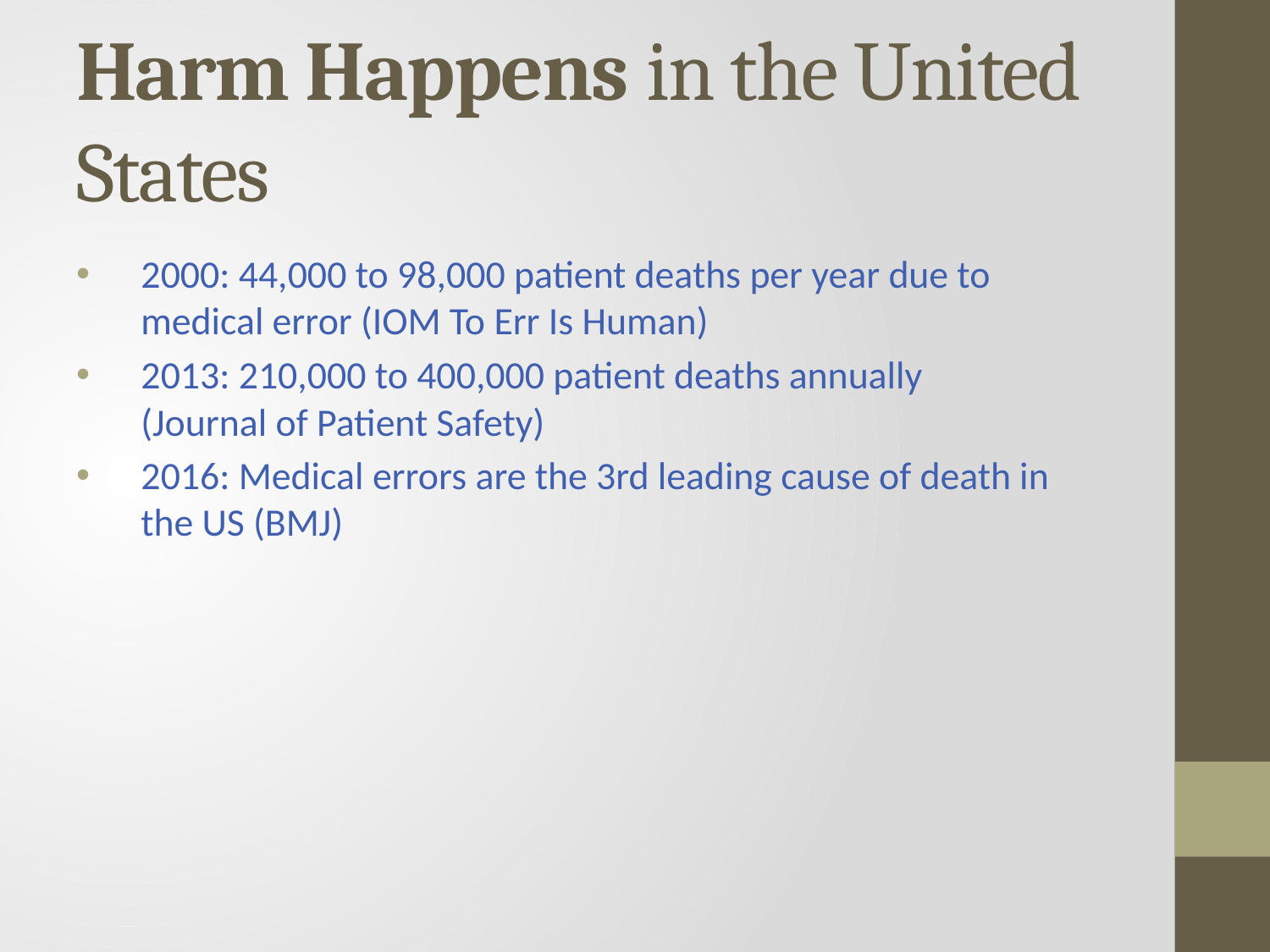

# Harm Happens in the United States
2000: 44,000 to 98,000 patient deaths per year due to medical error (IOM To Err Is Human)
2013: 210,000 to 400,000 patient deaths annually
(Journal of Patient Safety)
2016: Medical errors are the 3rd leading cause of death in the US (BMJ)

## Slide 12
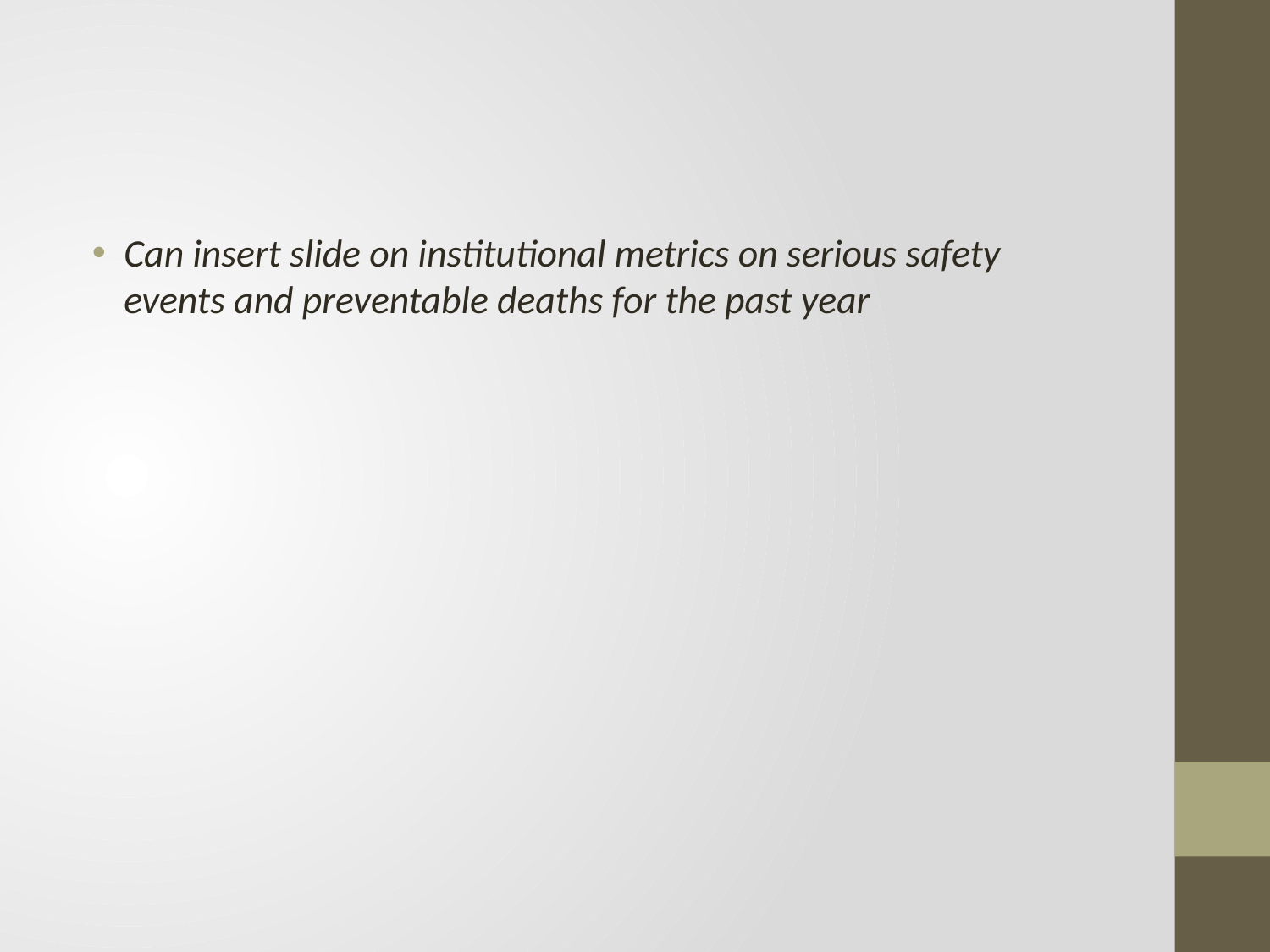

#
Can insert slide on institutional metrics on serious safety events and preventable deaths for the past year

## Slide 13
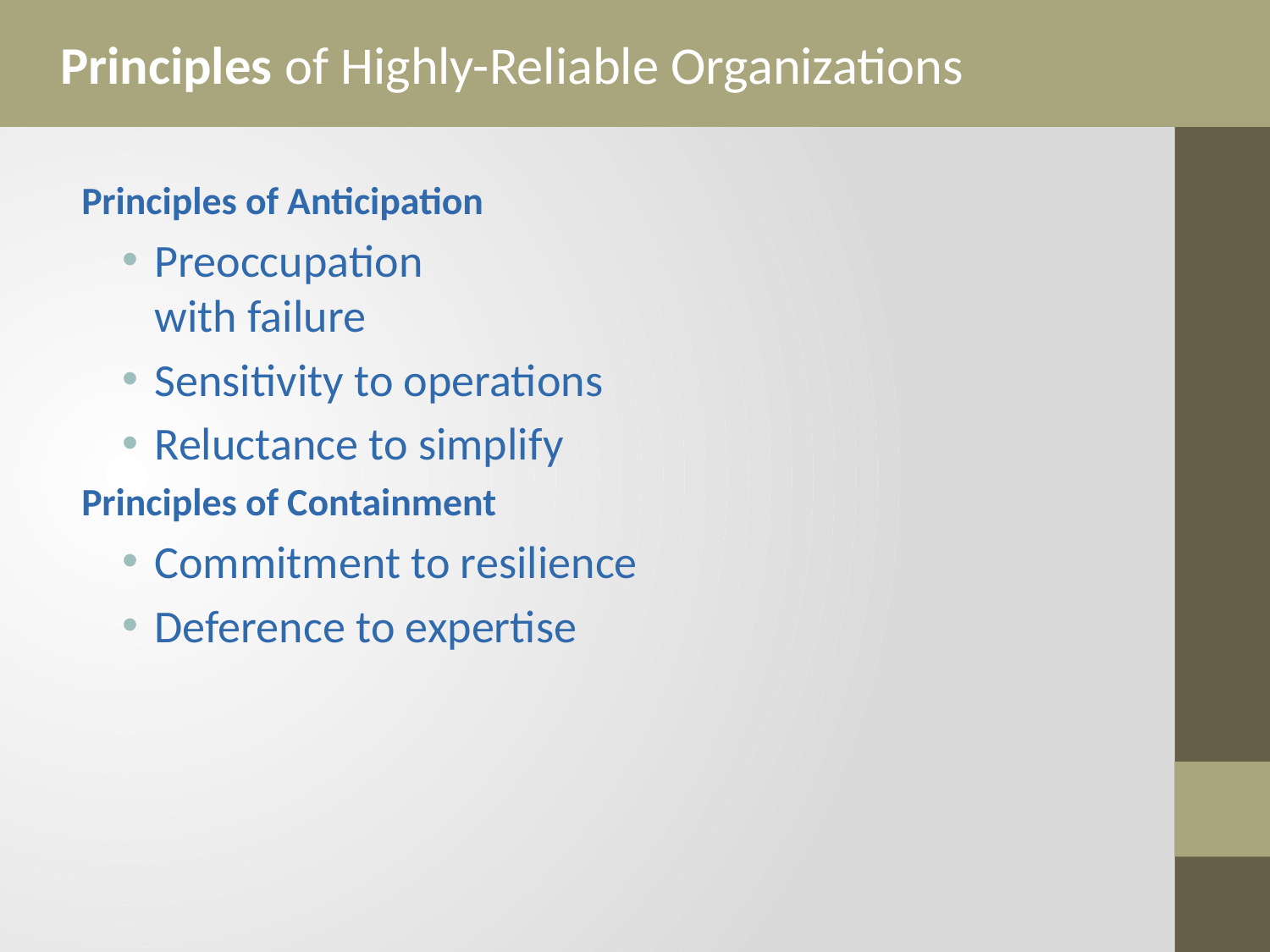

Principles of Highly-Reliable Organizations
Principles of Anticipation
Preoccupation
with failure
Sensitivity to operations
Reluctance to simplify
Principles of Containment
Commitment to resilience
Deference to expertise

## Slide 14
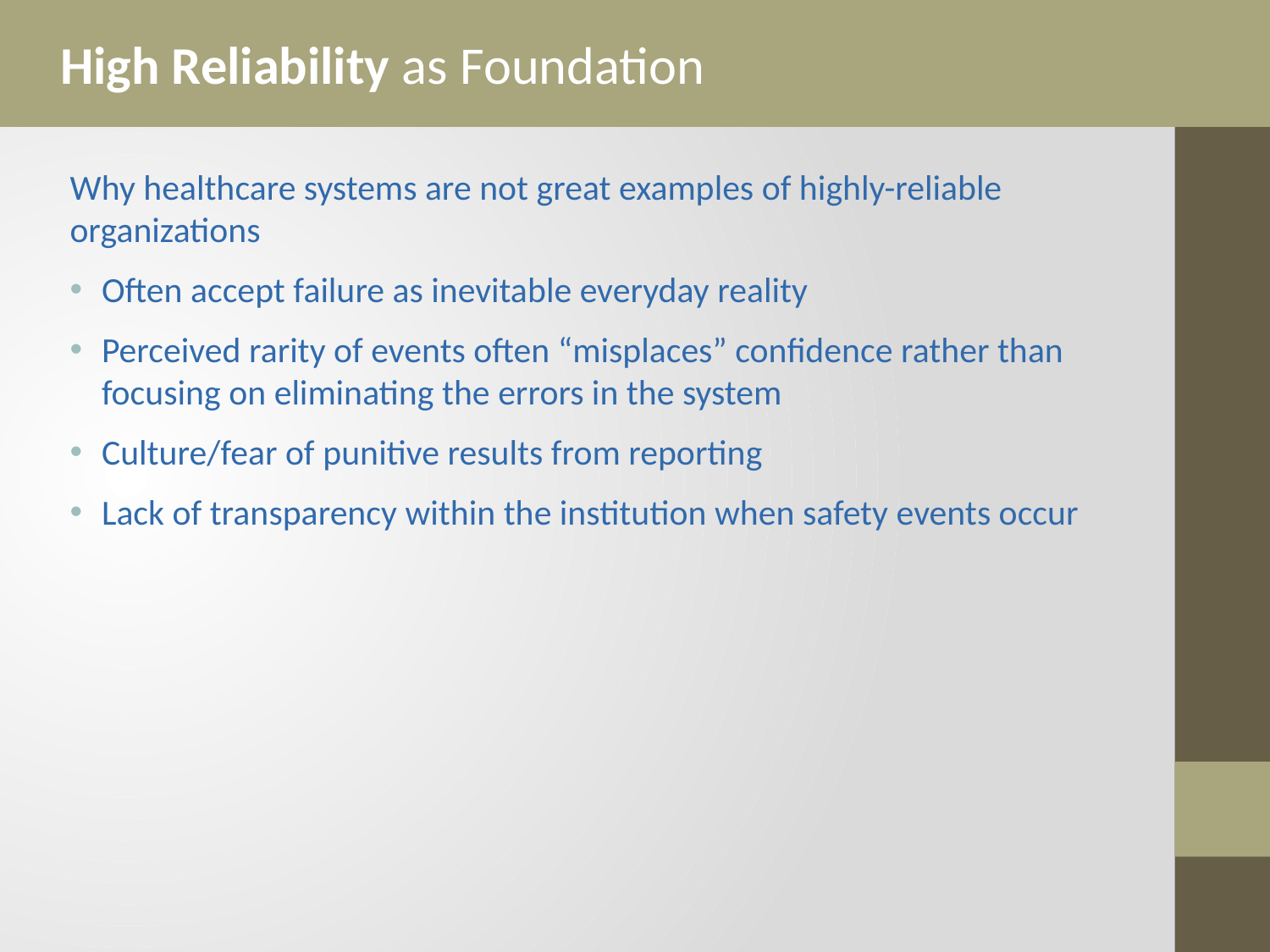

High Reliability as Foundation
Why healthcare systems are not great examples of highly-reliable organizations
Often accept failure as inevitable everyday reality
Perceived rarity of events often “misplaces” confidence rather than focusing on eliminating the errors in the system
Culture/fear of punitive results from reporting
Lack of transparency within the institution when safety events occur

## Slide 15
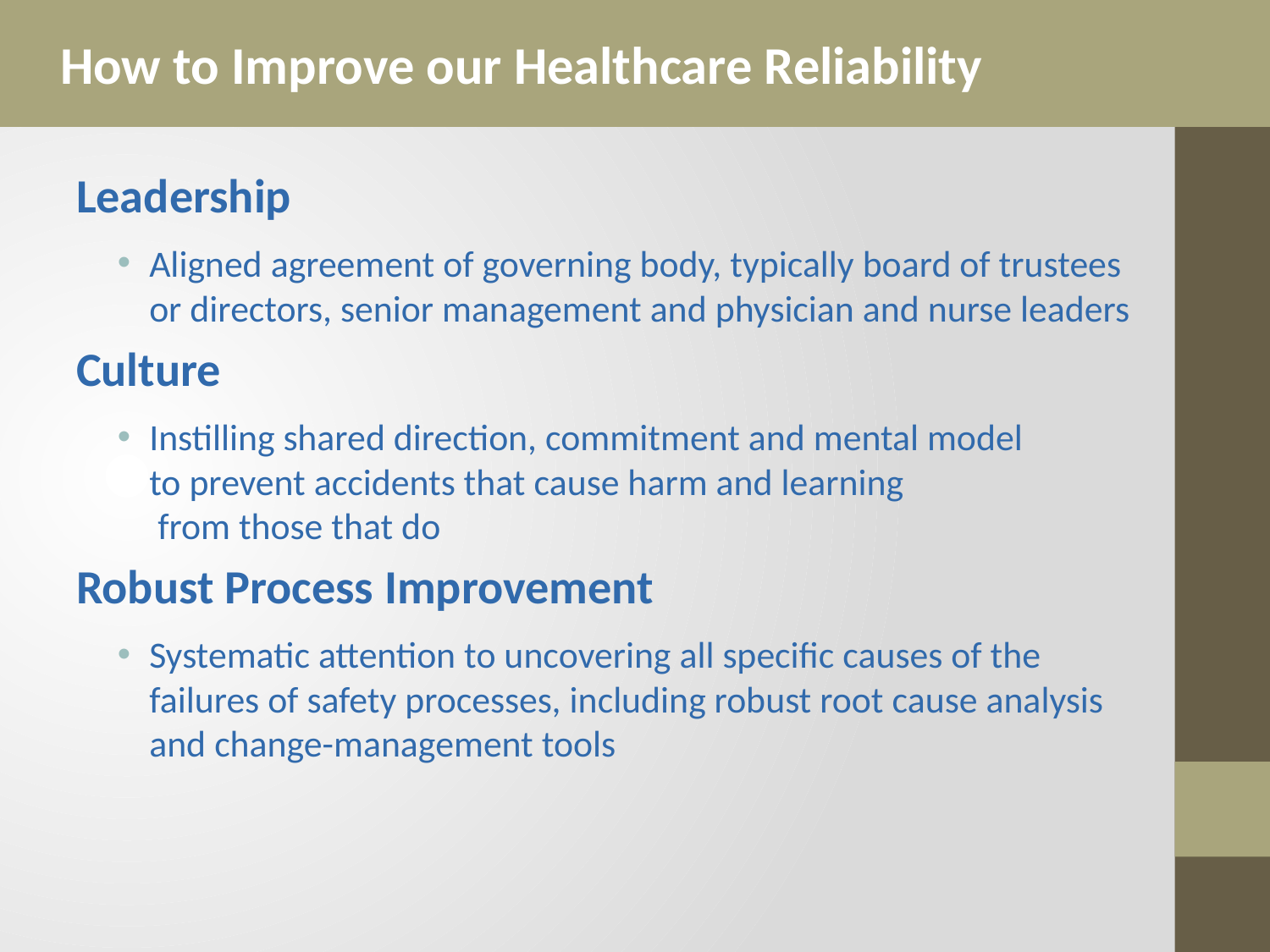

How to Improve our Healthcare Reliability
Leadership
Aligned agreement of governing body, typically board of trustees
or directors, senior management and physician and nurse leaders
Culture
Instilling shared direction, commitment and mental model
to prevent accidents that cause harm and learning
 from those that do
Robust Process Improvement
Systematic attention to uncovering all specific causes of the
failures of safety processes, including robust root cause analysis
and change-management tools

## Slide 16
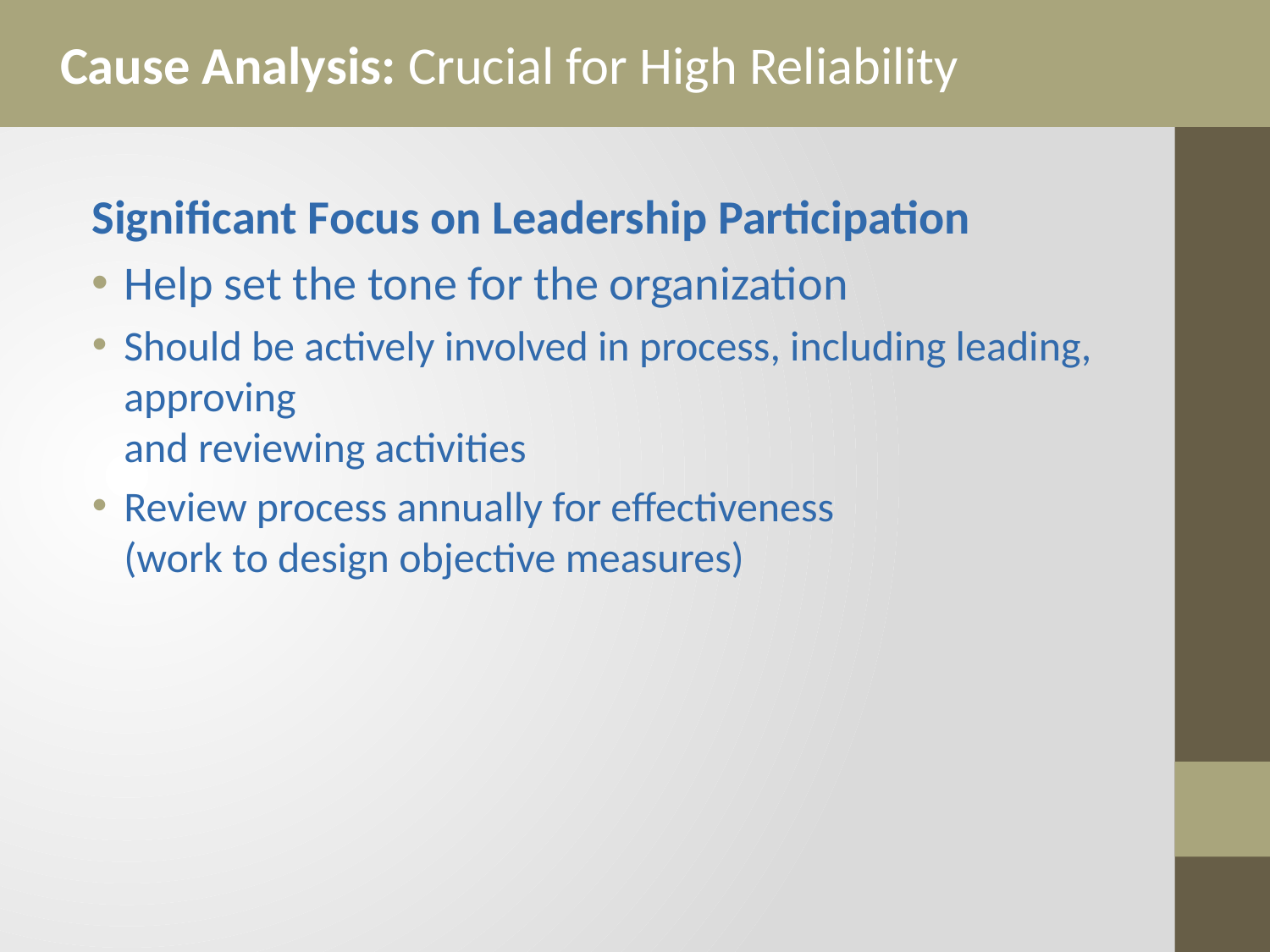

Cause Analysis: Crucial for High Reliability
Significant Focus on Leadership Participation
Help set the tone for the organization
Should be actively involved in process, including leading, approving
and reviewing activities
Review process annually for effectiveness
(work to design objective measures)

## Slide 17
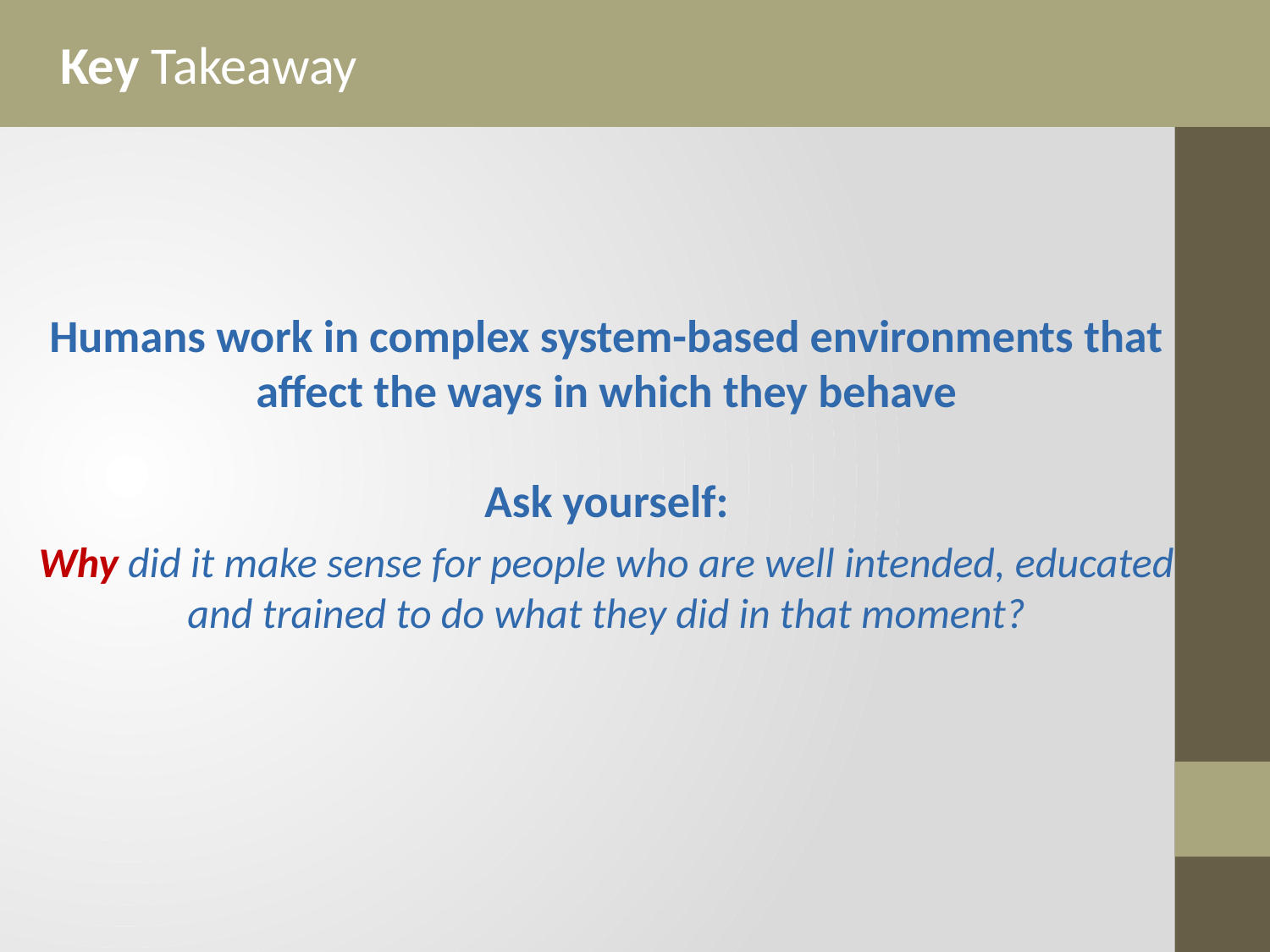

Key Takeaway
Humans work in complex system-based environments that affect the ways in which they behave
Ask yourself:
Why did it make sense for people who are well intended, educated and trained to do what they did in that moment?

## Slide 18
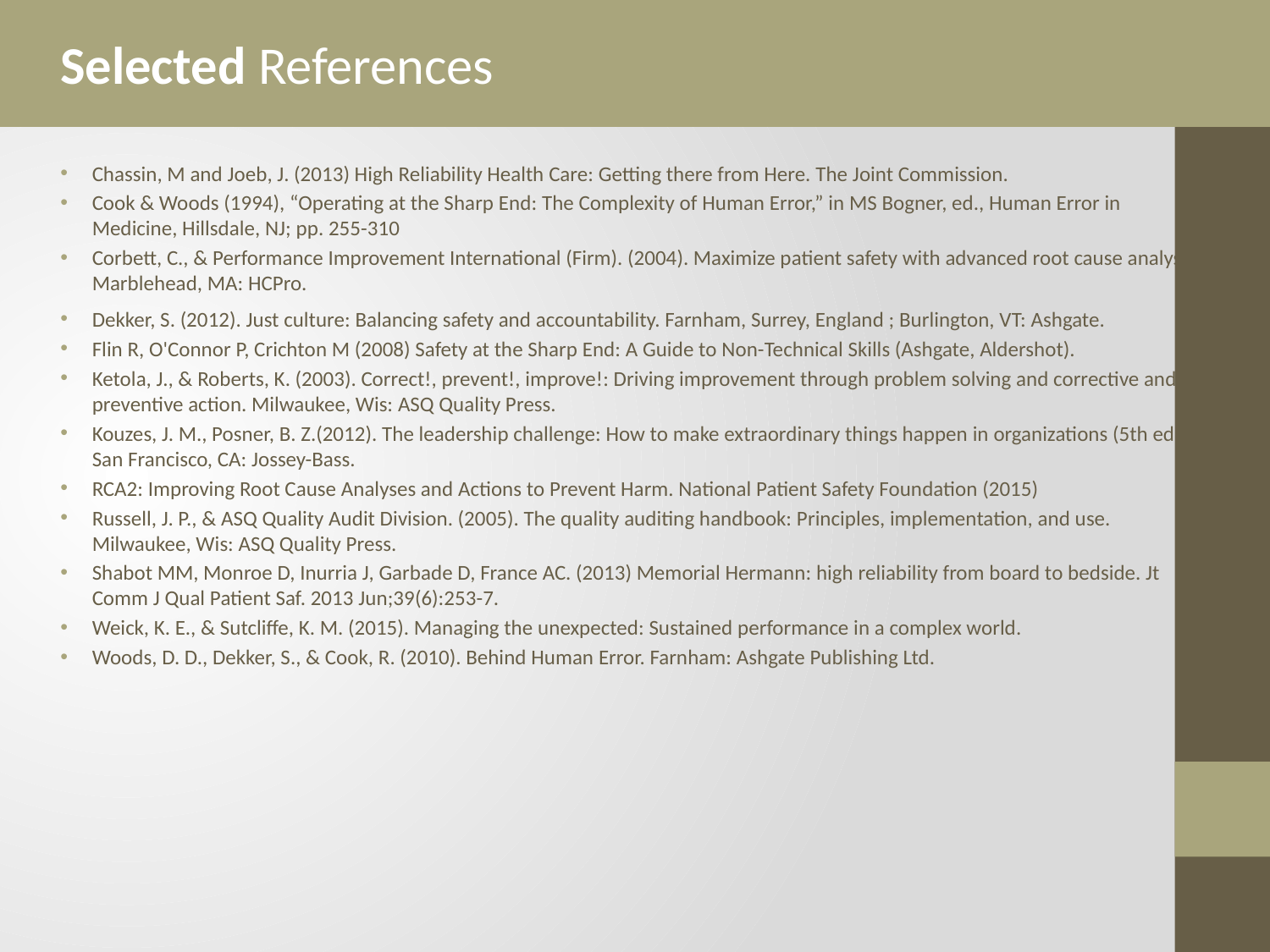

Selected References
Chassin, M and Joeb, J. (2013) High Reliability Health Care: Getting there from Here. The Joint Commission.
Cook & Woods (1994), “Operating at the Sharp End: The Complexity of Human Error,” in MS Bogner, ed., Human Error in Medicine, Hillsdale, NJ; pp. 255-310
Corbett, C., & Performance Improvement International (Firm). (2004). Maximize patient safety with advanced root cause analysis. Marblehead, MA: HCPro.
Dekker, S. (2012). Just culture: Balancing safety and accountability. Farnham, Surrey, England ; Burlington, VT: Ashgate.
Flin R, O'Connor P, Crichton M (2008) Safety at the Sharp End: A Guide to Non-Technical Skills (Ashgate, Aldershot).
Ketola, J., & Roberts, K. (2003). Correct!, prevent!, improve!: Driving improvement through problem solving and corrective and preventive action. Milwaukee, Wis: ASQ Quality Press.
Kouzes, J. M., Posner, B. Z.(2012). The leadership challenge: How to make extraordinary things happen in organizations (5th ed.). San Francisco, CA: Jossey-Bass.
RCA2: Improving Root Cause Analyses and Actions to Prevent Harm. National Patient Safety Foundation (2015)
Russell, J. P., & ASQ Quality Audit Division. (2005). The quality auditing handbook: Principles, implementation, and use. Milwaukee, Wis: ASQ Quality Press.
Shabot MM, Monroe D, Inurria J, Garbade D, France AC. (2013) Memorial Hermann: high reliability from board to bedside. Jt Comm J Qual Patient Saf. 2013 Jun;39(6):253-7.
Weick, K. E., & Sutcliffe, K. M. (2015). Managing the unexpected: Sustained performance in a complex world.
Woods, D. D., Dekker, S., & Cook, R. (2010). Behind Human Error. Farnham: Ashgate Publishing Ltd.
