# Supplementary material for: Safety in Numbers: Successful Student-Approved Case-Based Interprofessional Safety Workshop Utilizing Simulated Real-Life Safety Cases
Source: MedEdPORTAL. 2020 Jan 31;16:10874. doi: 10.15766/mep_2374-8265.10874 (PMC7065299; doi:10.15766/mep_2374-8265.10874)
Supplement: Supplementary file 1 — A. Pre- & Postevent Surveys.docx B. IPE Safety Workshop Agenda.docx C. RCA AM Session Facilitator Guide.docx D. RCA AM Session Facilitator Annotated Case Time Line.docx E. RCA AM Session Student Case Time Line.docx F. RCA AM Session Interviewee Scripts.docx G. RCA AM Session Patient Background & EWS Info.docx H. RCA AM Session Media - Radiology.docx I. RCA AM Session Media - Oxygen Tanks.docx J. Corrective Action PM Session Facilitator Guide.docx K. Corrective Action PM Session Effectiveness Chart.docx L. Corrective Action PM Session Worksheet.docx M. Executive Case Summary.docx N. Large-Group Lecture Schedule & Topic List.docx O. PPT 1 - Contributing to a Culture of Safety.pptx P. PPT 2 - Systems Improvement.pptx Q. PPT 3 - Impact of Students and Residents on QI.pptx R. PPT 4 - Presentation of Safety Case.pptx S. PPT 5 - Disclosing Medical Errors.pptx T. PPT 6 - Training for Resilience.pptx U. PPT 7 - Introduction to Improvement Plans.pptx V. Facilitator Postworkshop Survey.docx [file mep-16-10874-s001.zip › P. PPT 2 - Systems Improvement.pptx]

## Slide 1
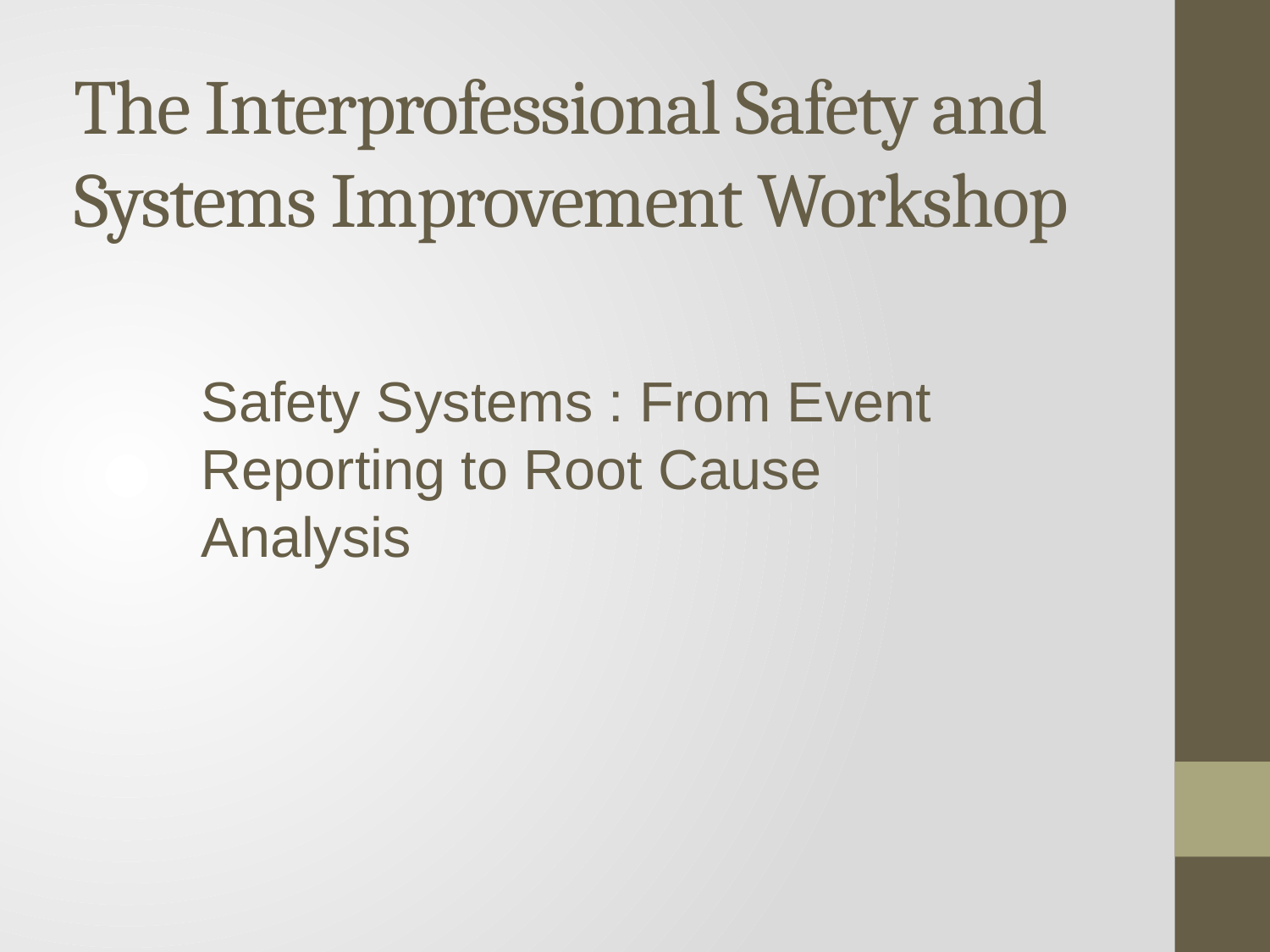

# The Interprofessional Safety and Systems Improvement Workshop
Safety Systems : From Event Reporting to Root Cause Analysis

## Slide 2
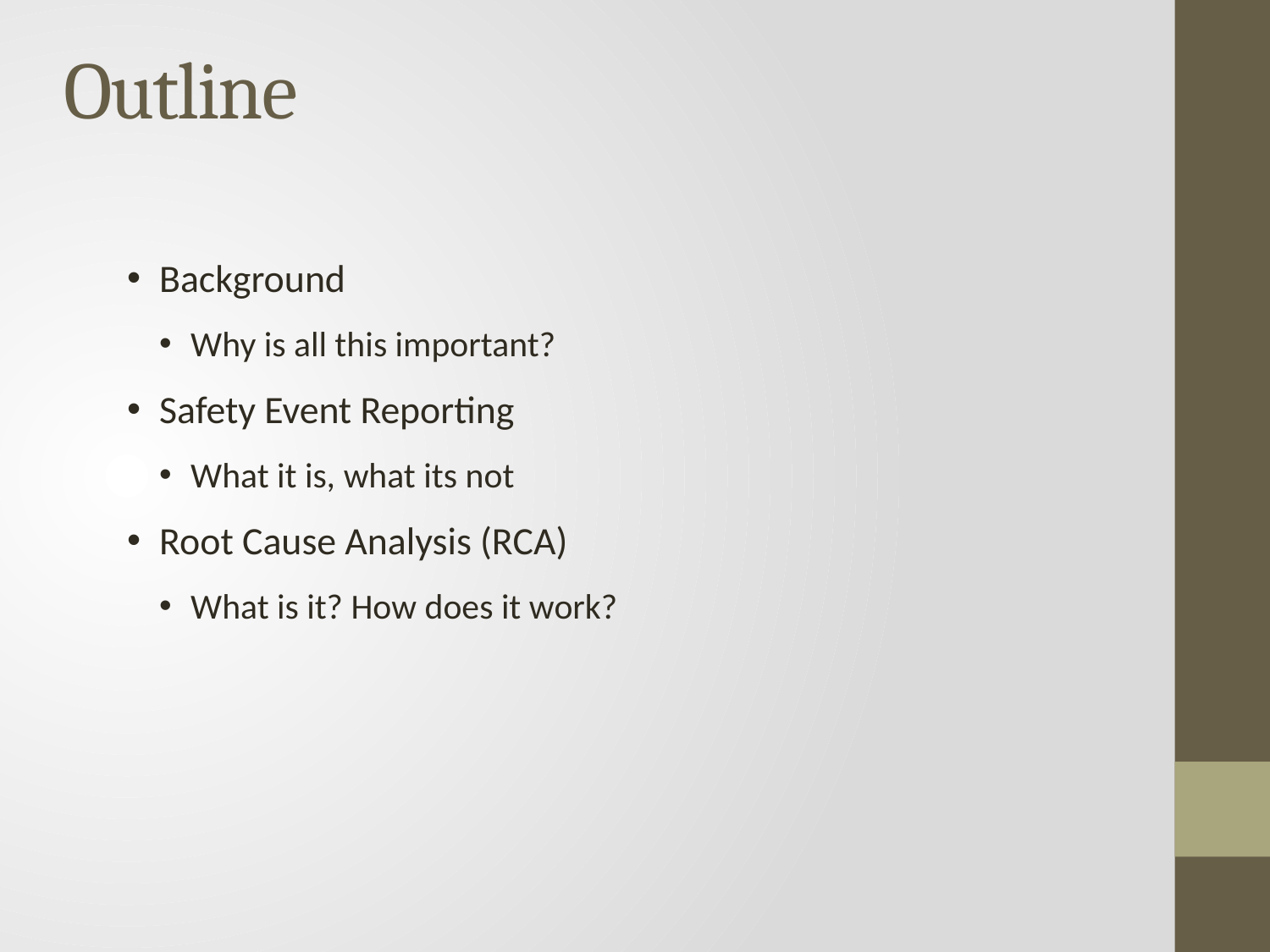

# Outline
Background
Why is all this important?
Safety Event Reporting
What it is, what its not
Root Cause Analysis (RCA)
What is it? How does it work?

## Slide 3
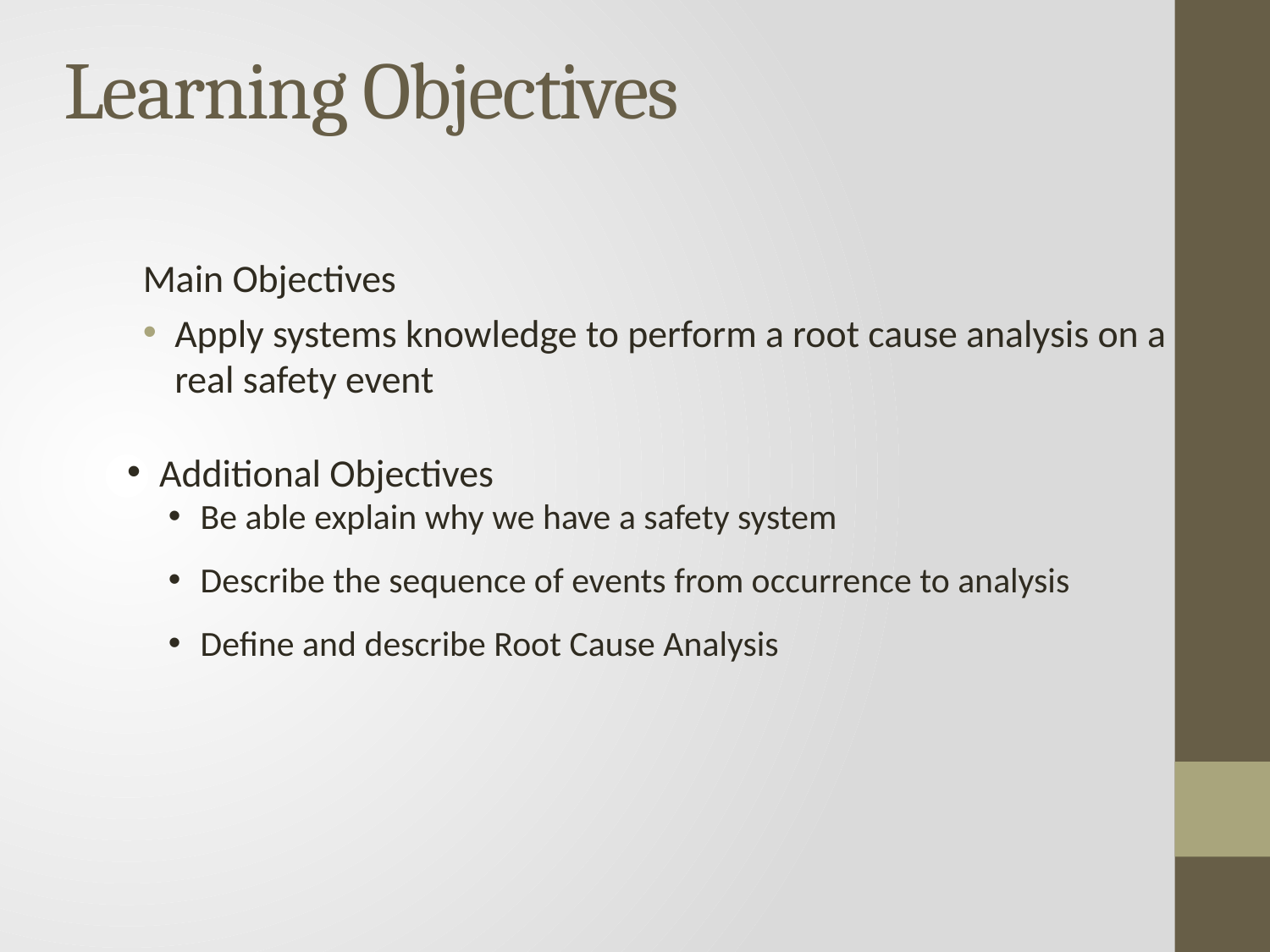

# Learning Objectives
Main Objectives
Apply systems knowledge to perform a root cause analysis on a real safety event
Additional Objectives
Be able explain why we have a safety system
Describe the sequence of events from occurrence to analysis
Define and describe Root Cause Analysis

## Slide 4
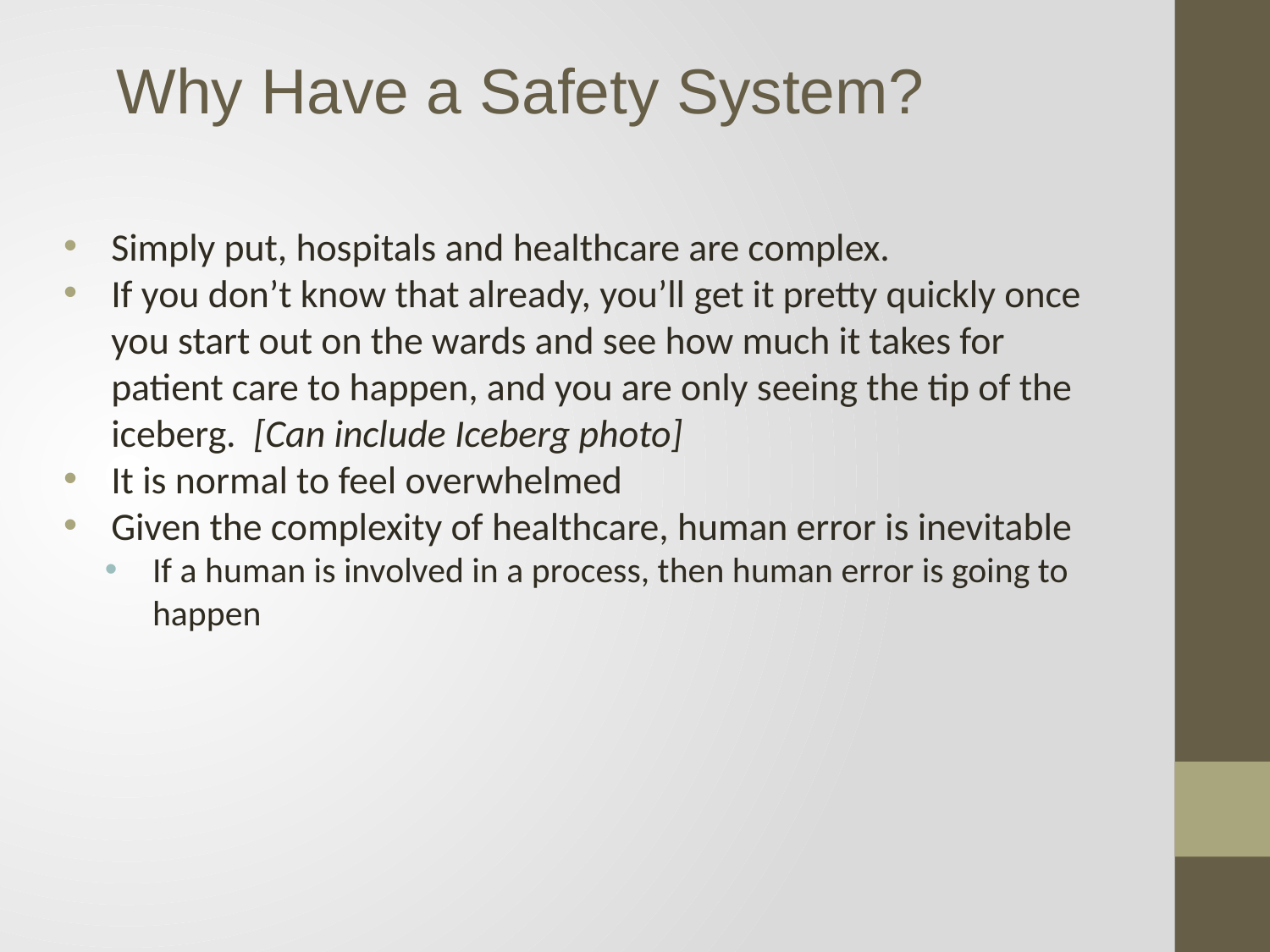

Why Have a Safety System?
Simply put, hospitals and healthcare are complex.
If you don’t know that already, you’ll get it pretty quickly once you start out on the wards and see how much it takes for patient care to happen, and you are only seeing the tip of the iceberg. [Can include Iceberg photo]
It is normal to feel overwhelmed
Given the complexity of healthcare, human error is inevitable
If a human is involved in a process, then human error is going to happen

## Slide 5
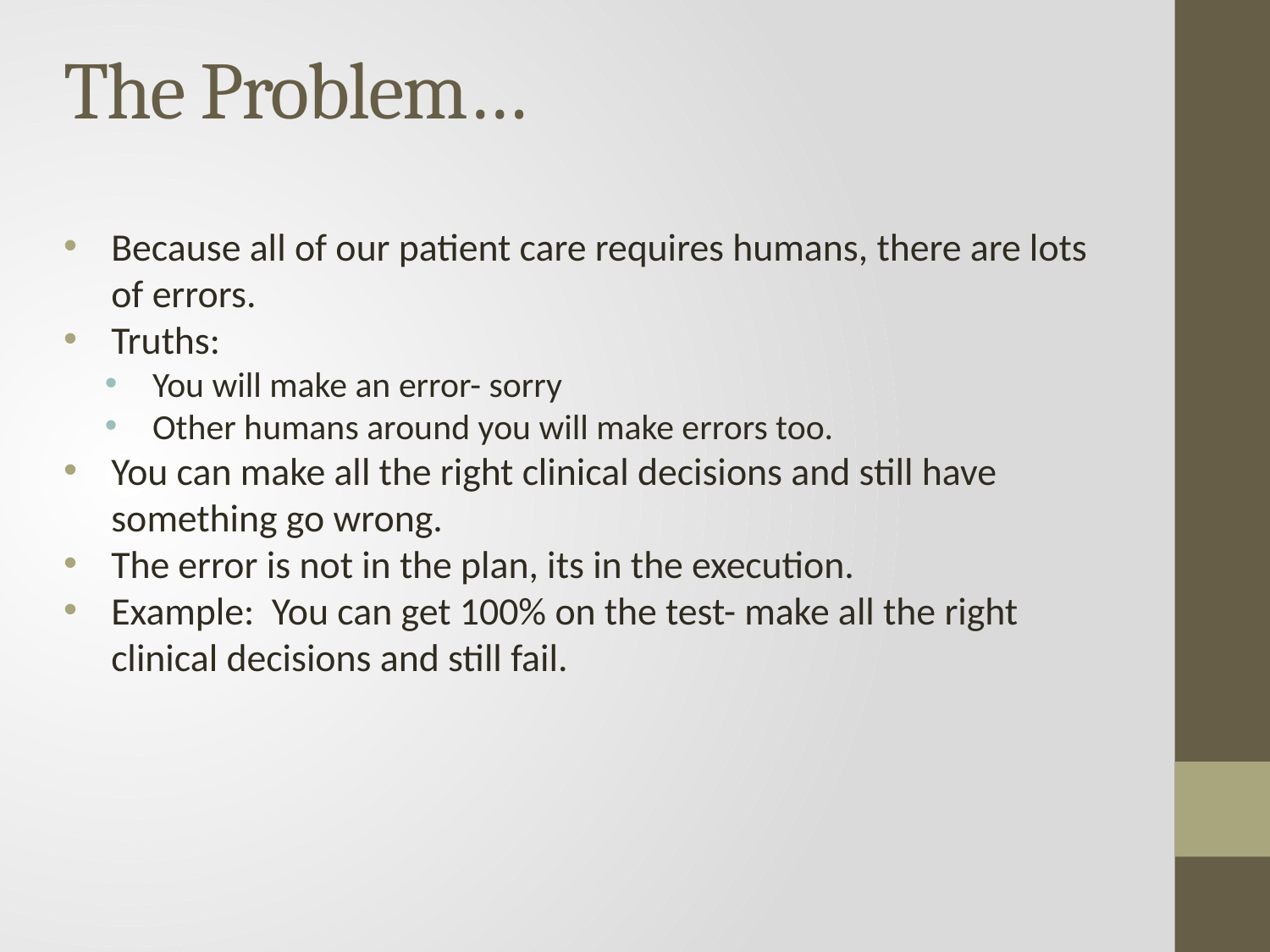

# The Problem…
Because all of our patient care requires humans, there are lots of errors.
Truths:
You will make an error- sorry
Other humans around you will make errors too.
You can make all the right clinical decisions and still have something go wrong.
The error is not in the plan, its in the execution.
Example: You can get 100% on the test- make all the right clinical decisions and still fail.

## Slide 6
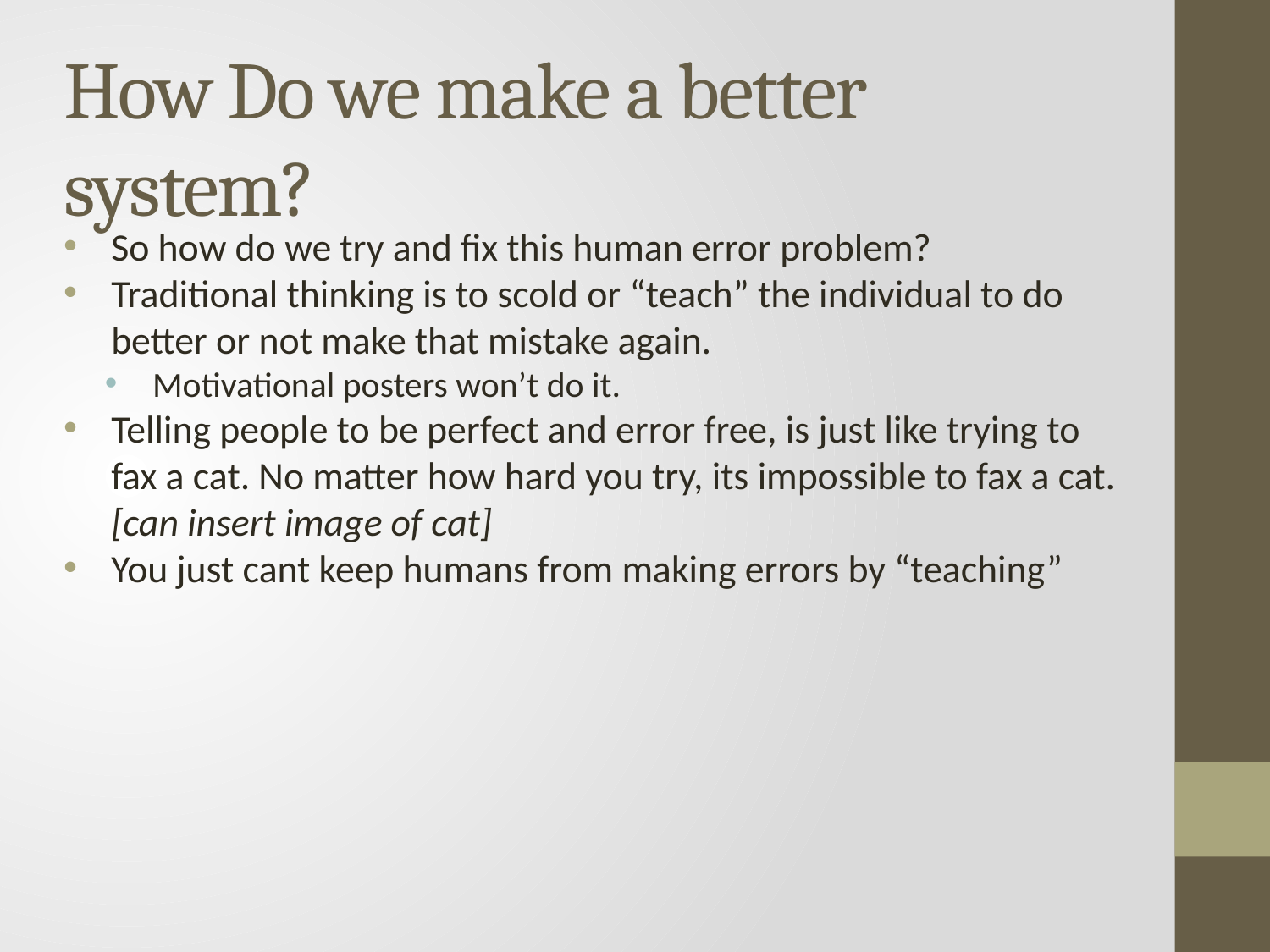

# How Do we make a better system?
So how do we try and fix this human error problem?
Traditional thinking is to scold or “teach” the individual to do better or not make that mistake again.
Motivational posters won’t do it.
Telling people to be perfect and error free, is just like trying to fax a cat. No matter how hard you try, its impossible to fax a cat.
[can insert image of cat]
You just cant keep humans from making errors by “teaching”

## Slide 7
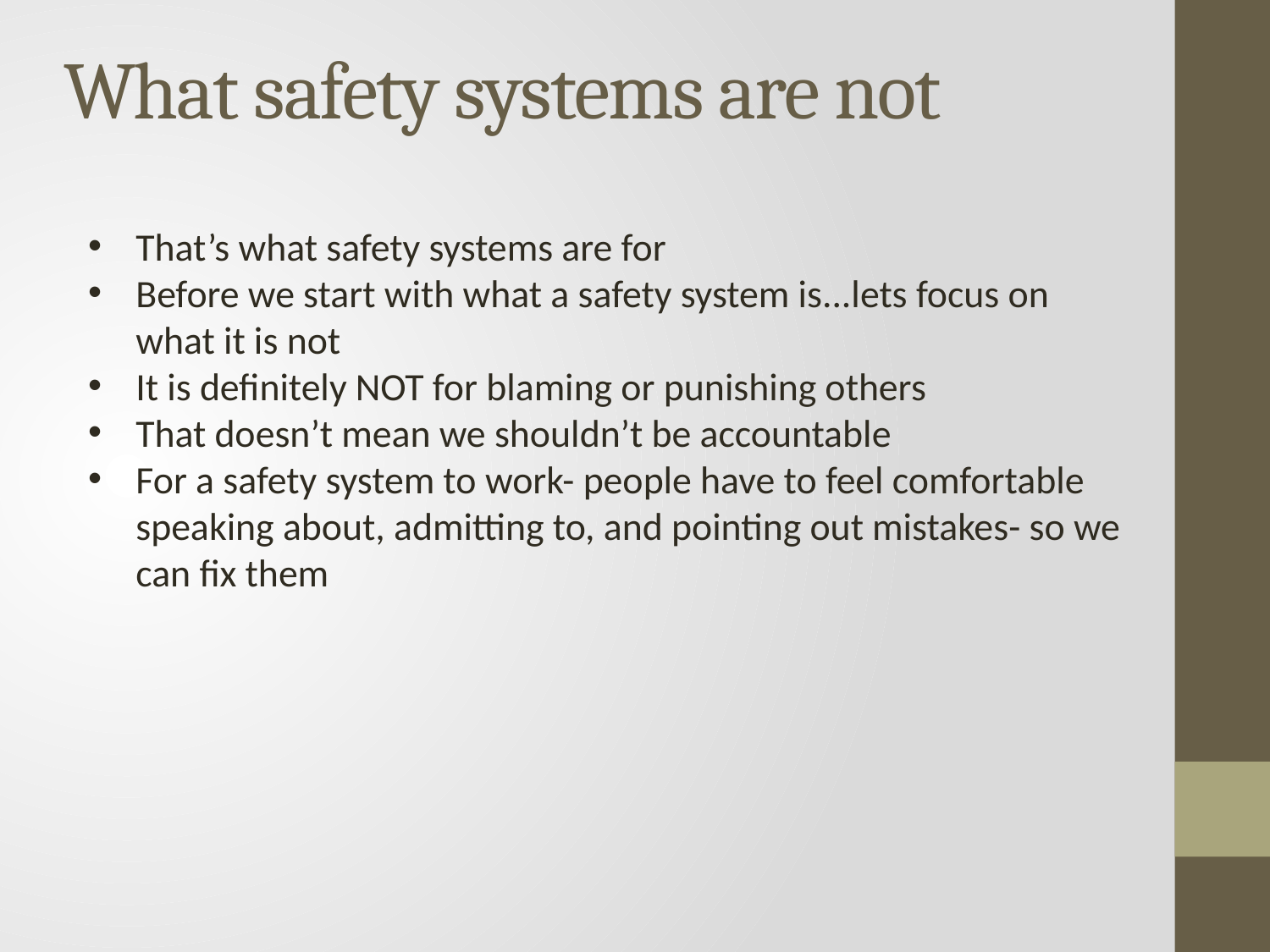

# What safety systems are not
That’s what safety systems are for
Before we start with what a safety system is...lets focus on what it is not
It is definitely NOT for blaming or punishing others
That doesn’t mean we shouldn’t be accountable
For a safety system to work- people have to feel comfortable speaking about, admitting to, and pointing out mistakes- so we can fix them

## Slide 8
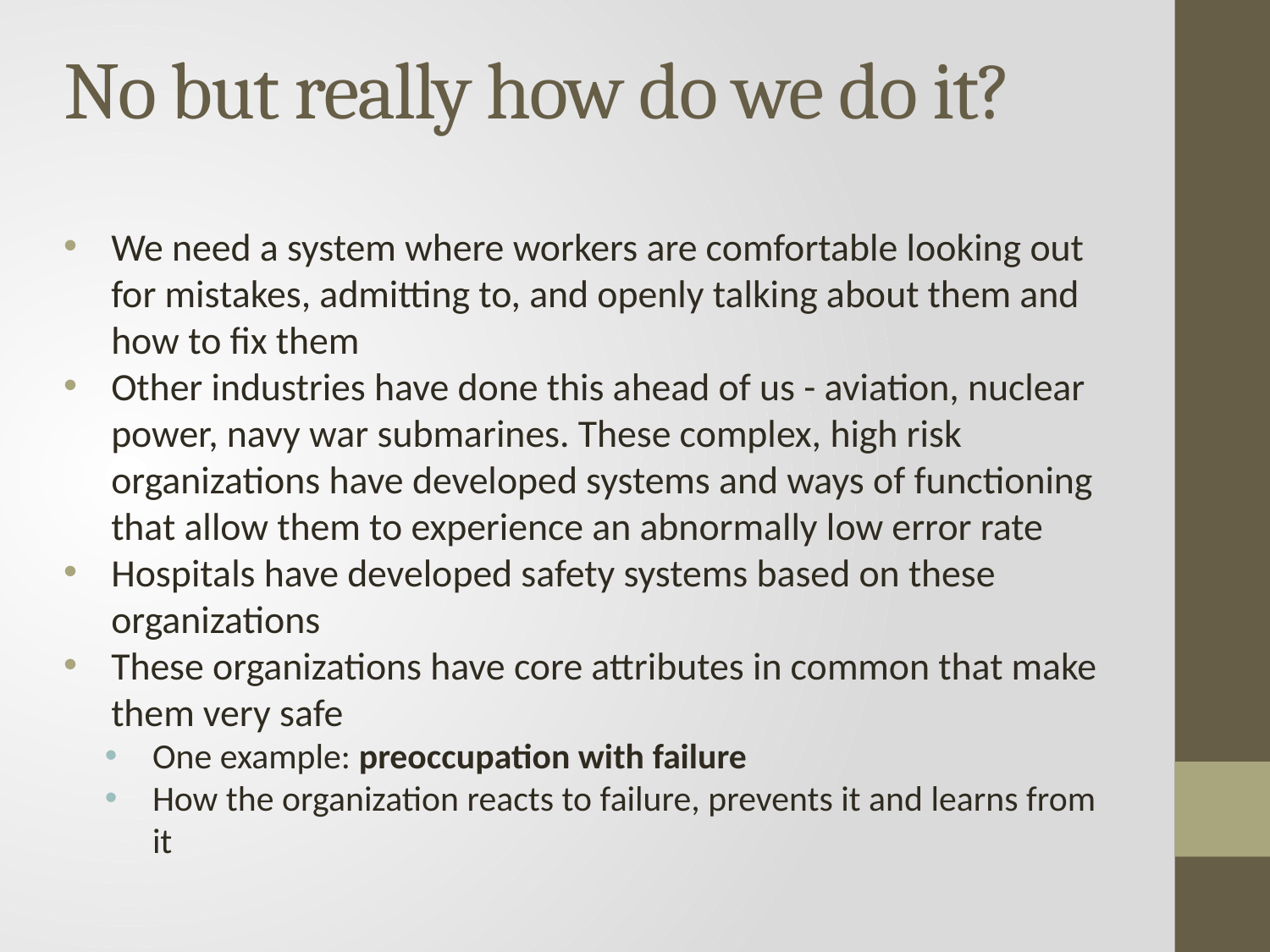

# No but really how do we do it?
We need a system where workers are comfortable looking out for mistakes, admitting to, and openly talking about them and how to fix them
Other industries have done this ahead of us - aviation, nuclear power, navy war submarines. These complex, high risk organizations have developed systems and ways of functioning that allow them to experience an abnormally low error rate
Hospitals have developed safety systems based on these organizations
These organizations have core attributes in common that make them very safe
One example: preoccupation with failure
How the organization reacts to failure, prevents it and learns from it

## Slide 9
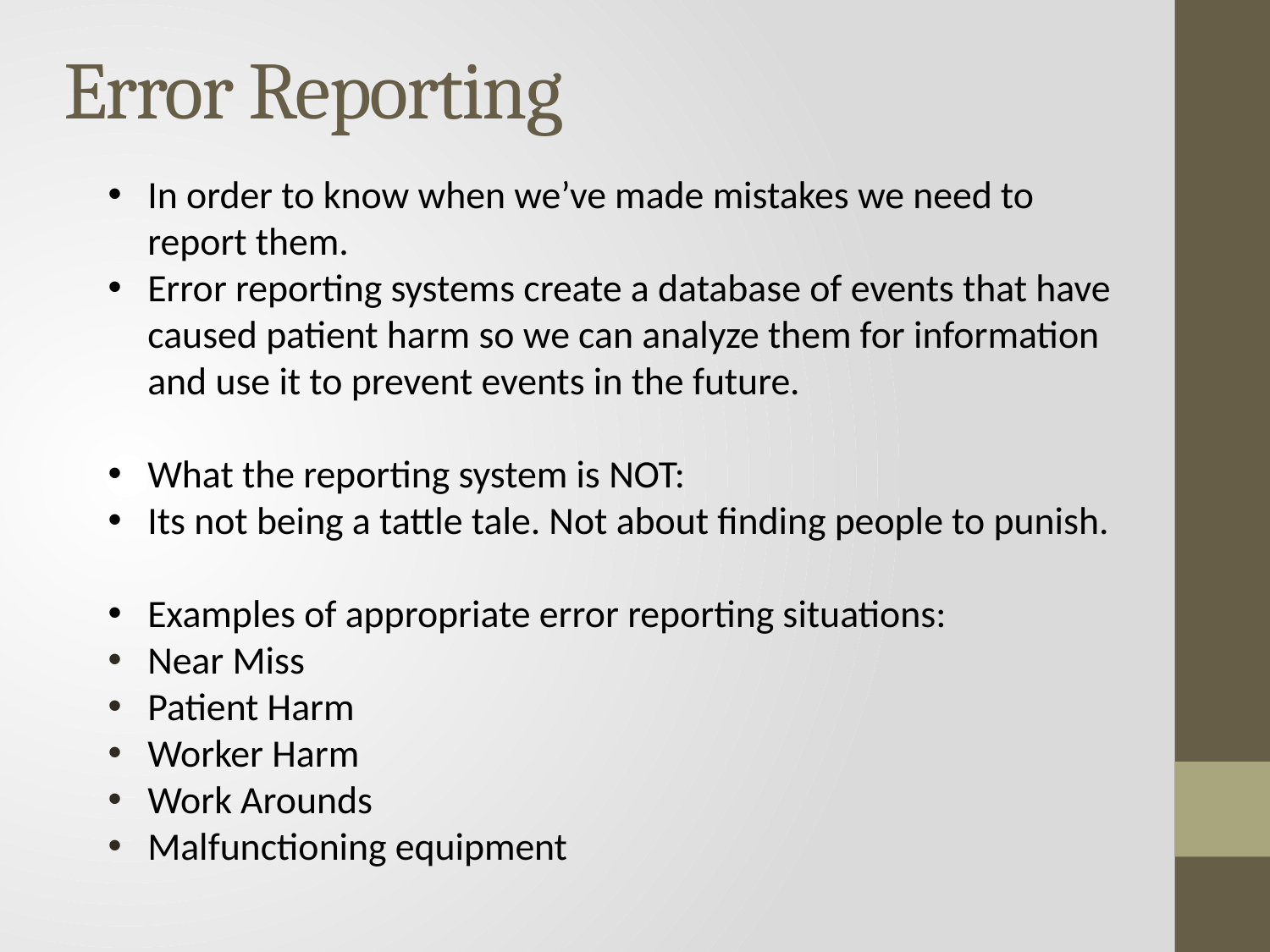

# Error Reporting
In order to know when we’ve made mistakes we need to report them.
Error reporting systems create a database of events that have caused patient harm so we can analyze them for information and use it to prevent events in the future.
What the reporting system is NOT:
Its not being a tattle tale. Not about finding people to punish.
Examples of appropriate error reporting situations:
Near Miss
Patient Harm
Worker Harm
Work Arounds
Malfunctioning equipment

## Slide 10
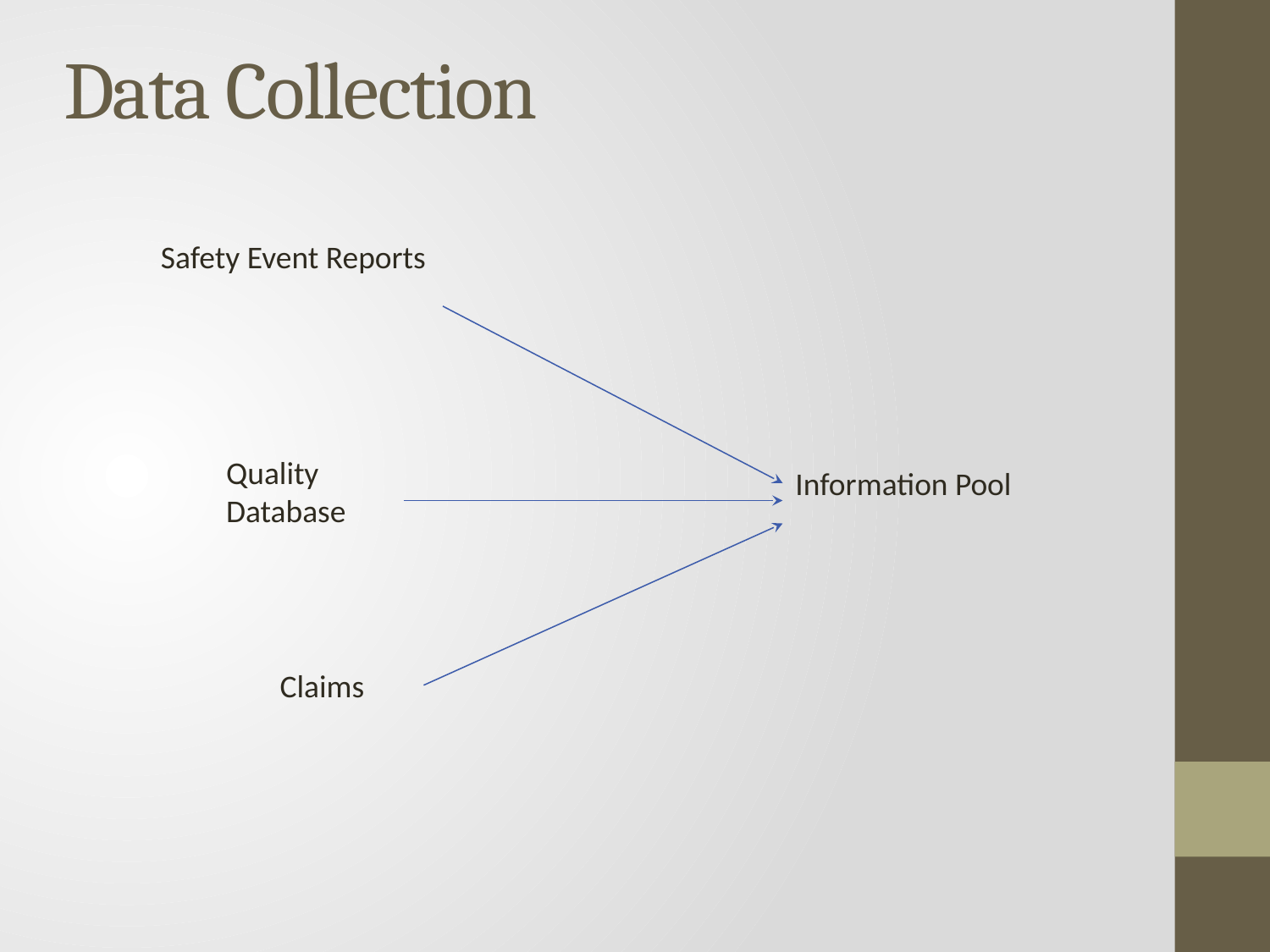

# Data Collection
Safety Event Reports
Quality Database
Information Pool
Claims

## Slide 11
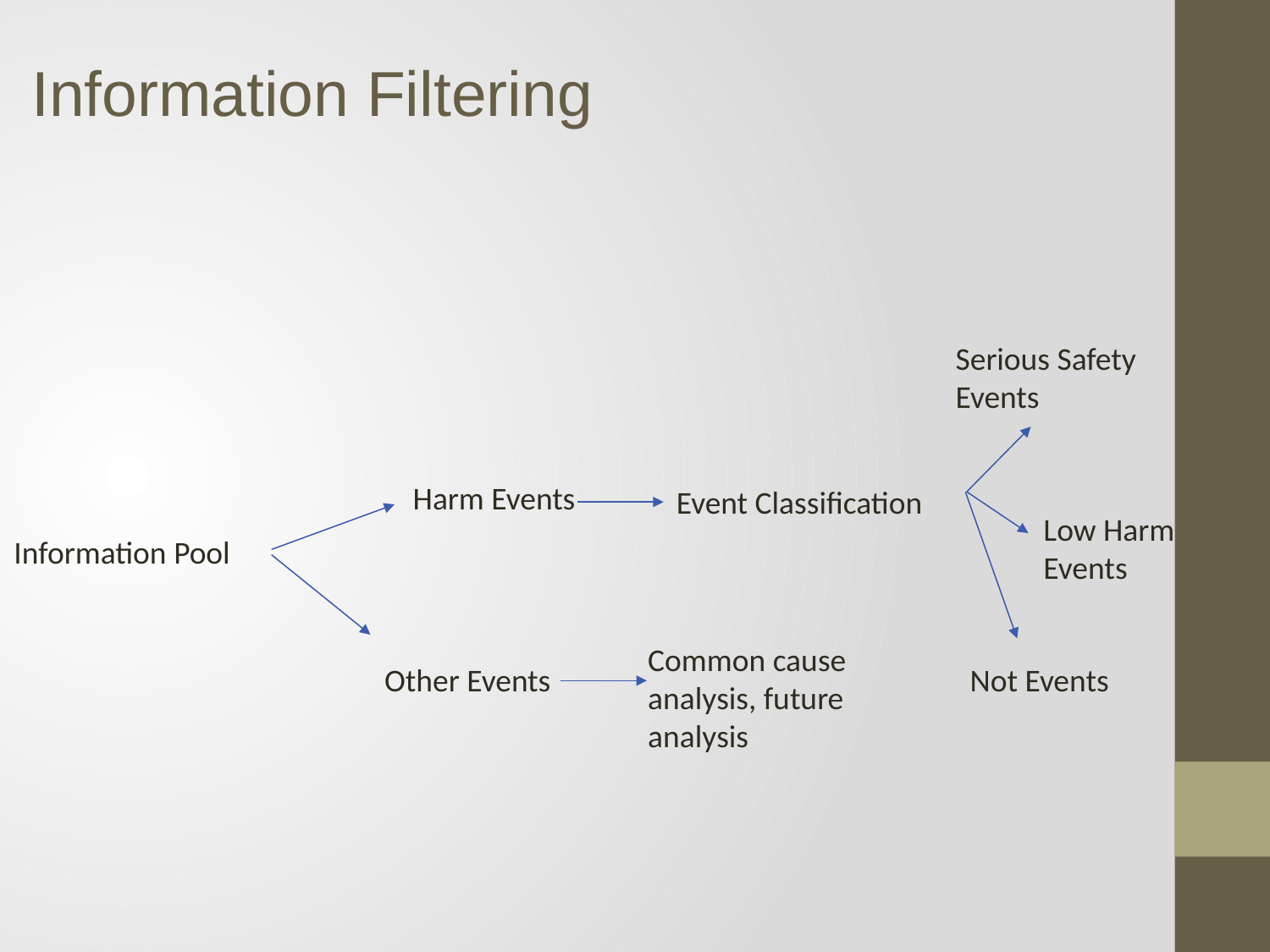

Information Filtering
Serious Safety Events
Harm Events
Event Classification
Low Harm Events
Information Pool
Common cause analysis, future analysis
Not Events
Other Events

## Slide 12
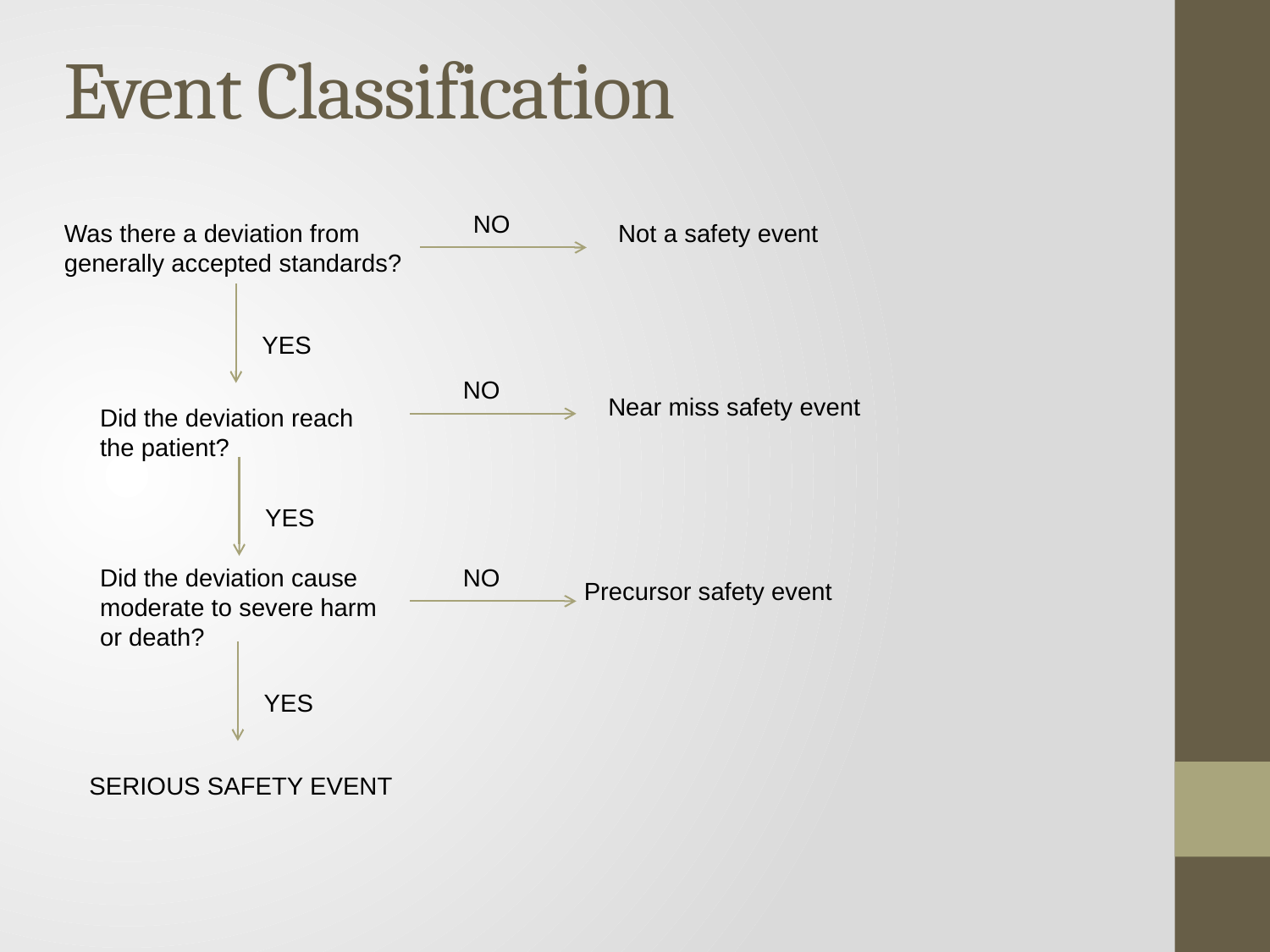

# Event Classification
NO
Was there a deviation from generally accepted standards?
Not a safety event
YES
NO
Near miss safety event
Did the deviation reach the patient?
YES
Did the deviation cause moderate to severe harm or death?
NO
Precursor safety event
YES
SERIOUS SAFETY EVENT

## Slide 13
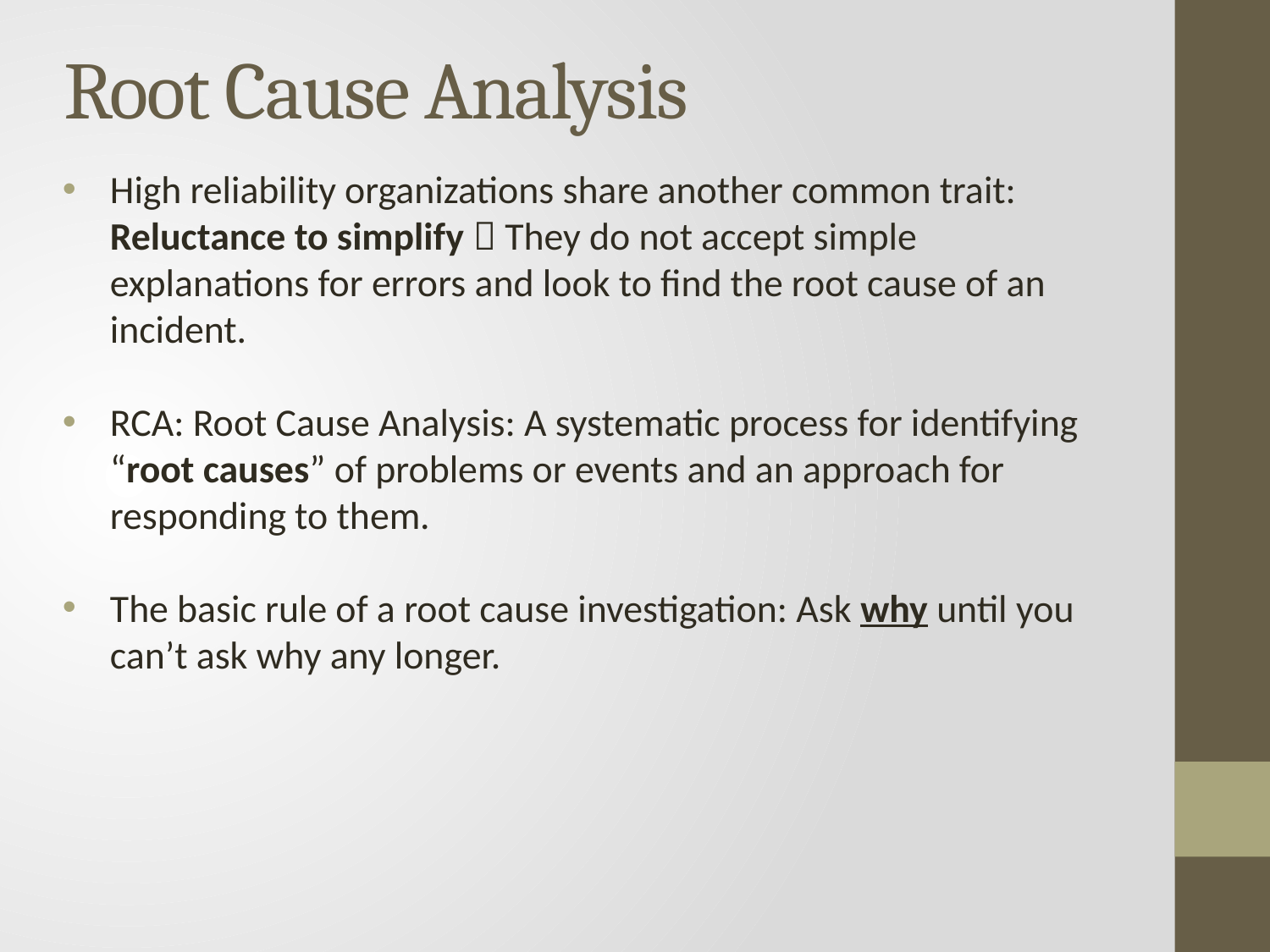

# Root Cause Analysis
High reliability organizations share another common trait: Reluctance to simplify  They do not accept simple explanations for errors and look to find the root cause of an incident.
RCA: Root Cause Analysis: A systematic process for identifying “root causes” of problems or events and an approach for responding to them.
The basic rule of a root cause investigation: Ask why until you can’t ask why any longer.

## Slide 14
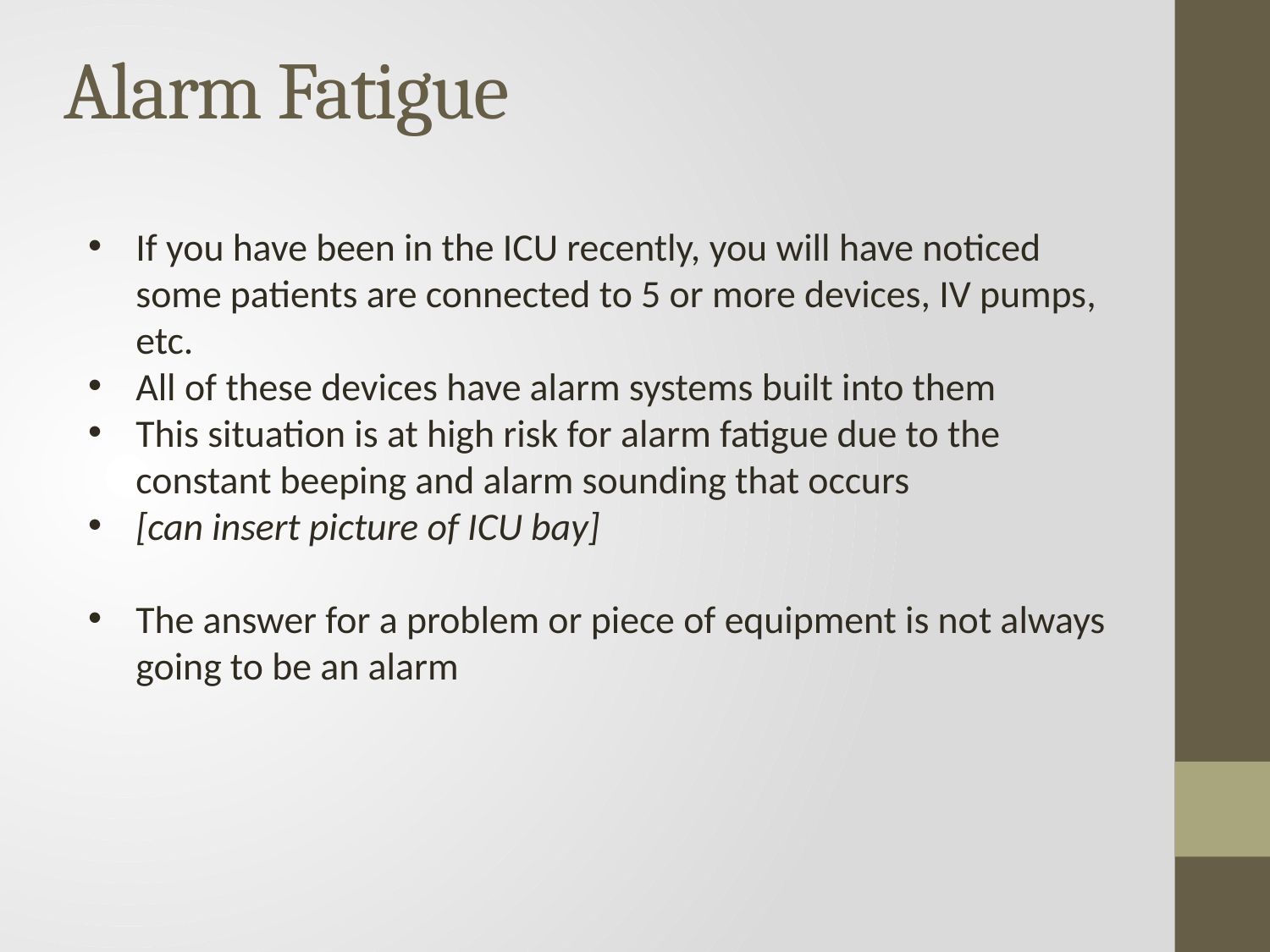

# Alarm Fatigue
If you have been in the ICU recently, you will have noticed some patients are connected to 5 or more devices, IV pumps, etc.
All of these devices have alarm systems built into them
This situation is at high risk for alarm fatigue due to the constant beeping and alarm sounding that occurs
[can insert picture of ICU bay]
The answer for a problem or piece of equipment is not always going to be an alarm

## Slide 15
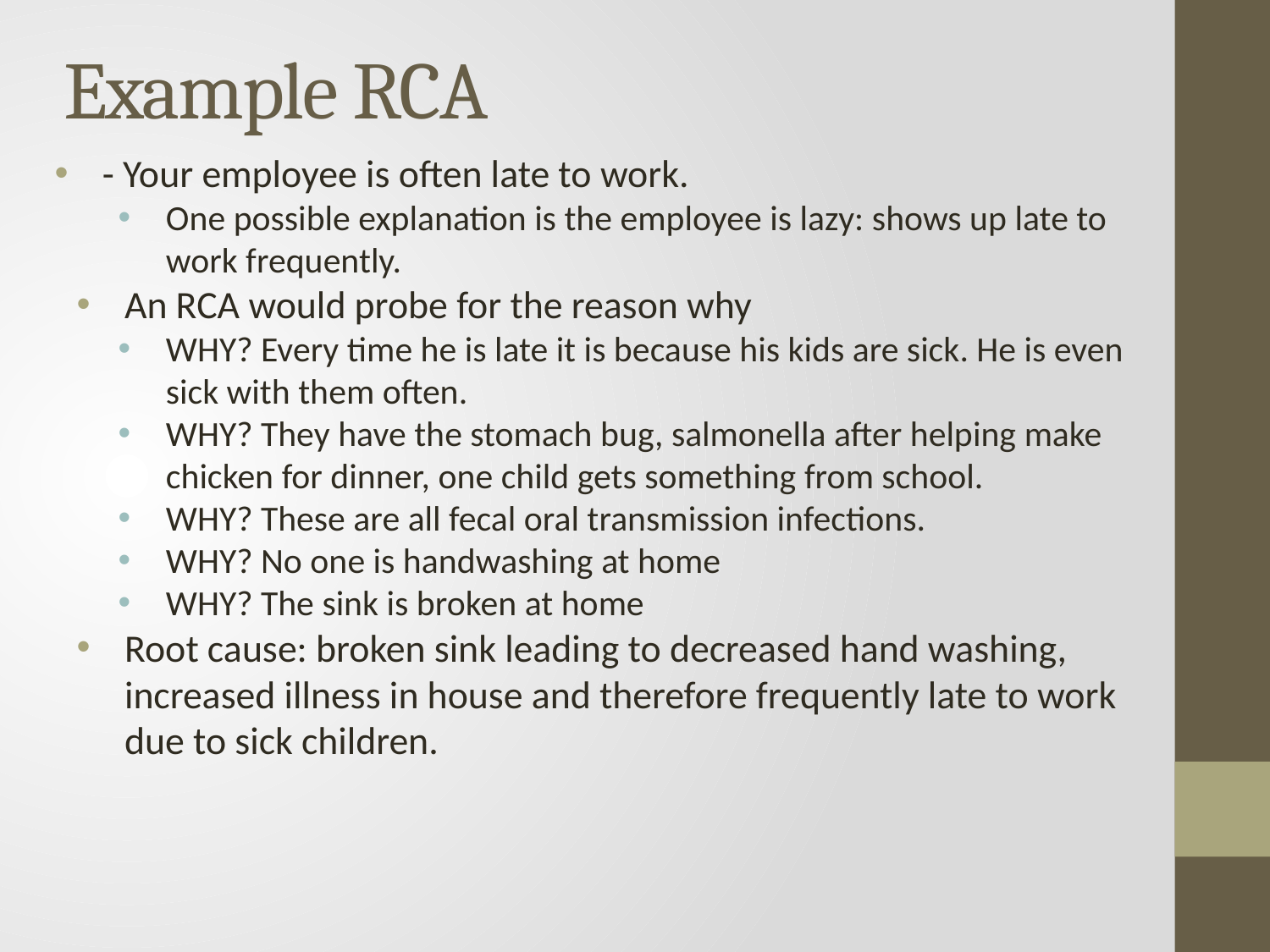

# Example RCA
- Your employee is often late to work.
One possible explanation is the employee is lazy: shows up late to work frequently.
An RCA would probe for the reason why
WHY? Every time he is late it is because his kids are sick. He is even sick with them often.
WHY? They have the stomach bug, salmonella after helping make chicken for dinner, one child gets something from school.
WHY? These are all fecal oral transmission infections.
WHY? No one is handwashing at home
WHY? The sink is broken at home
Root cause: broken sink leading to decreased hand washing, increased illness in house and therefore frequently late to work due to sick children.

## Slide 16
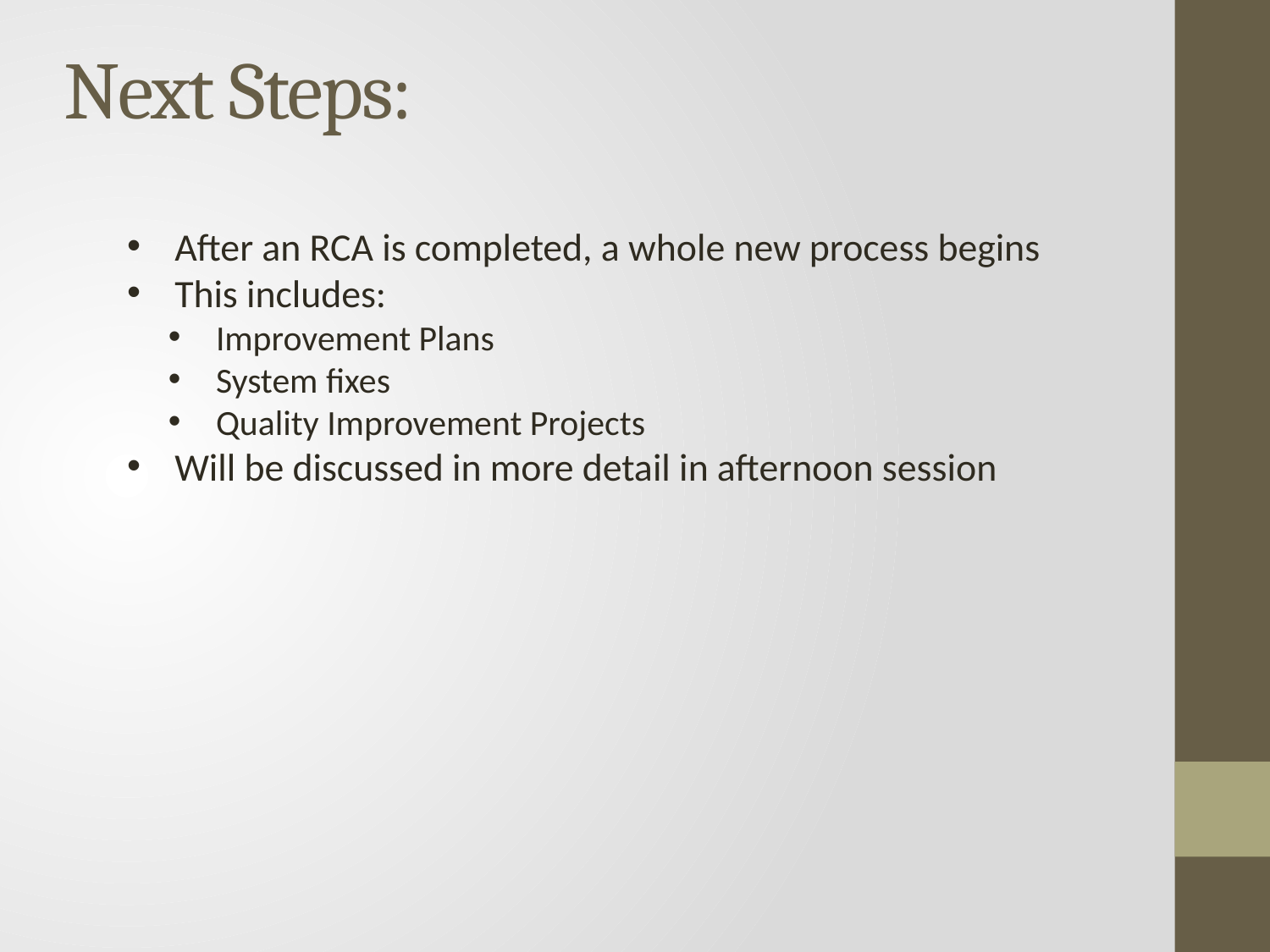

# Next Steps:
After an RCA is completed, a whole new process begins
This includes:
Improvement Plans
System fixes
Quality Improvement Projects
Will be discussed in more detail in afternoon session

## Slide 17
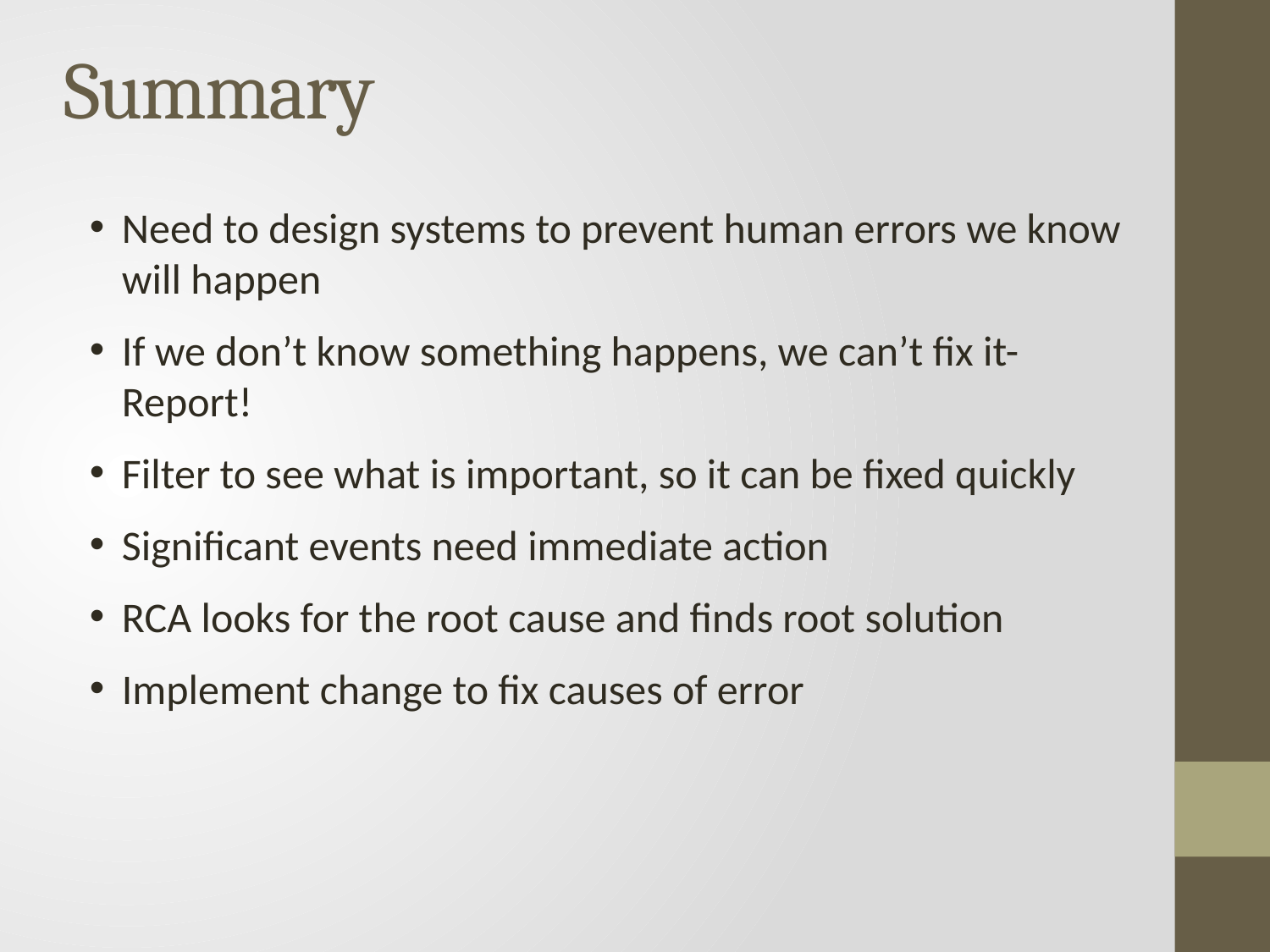

# Summary
Need to design systems to prevent human errors we know will happen
If we don’t know something happens, we can’t fix it- Report!
Filter to see what is important, so it can be fixed quickly
Significant events need immediate action
RCA looks for the root cause and finds root solution
Implement change to fix causes of error

## Slide 18
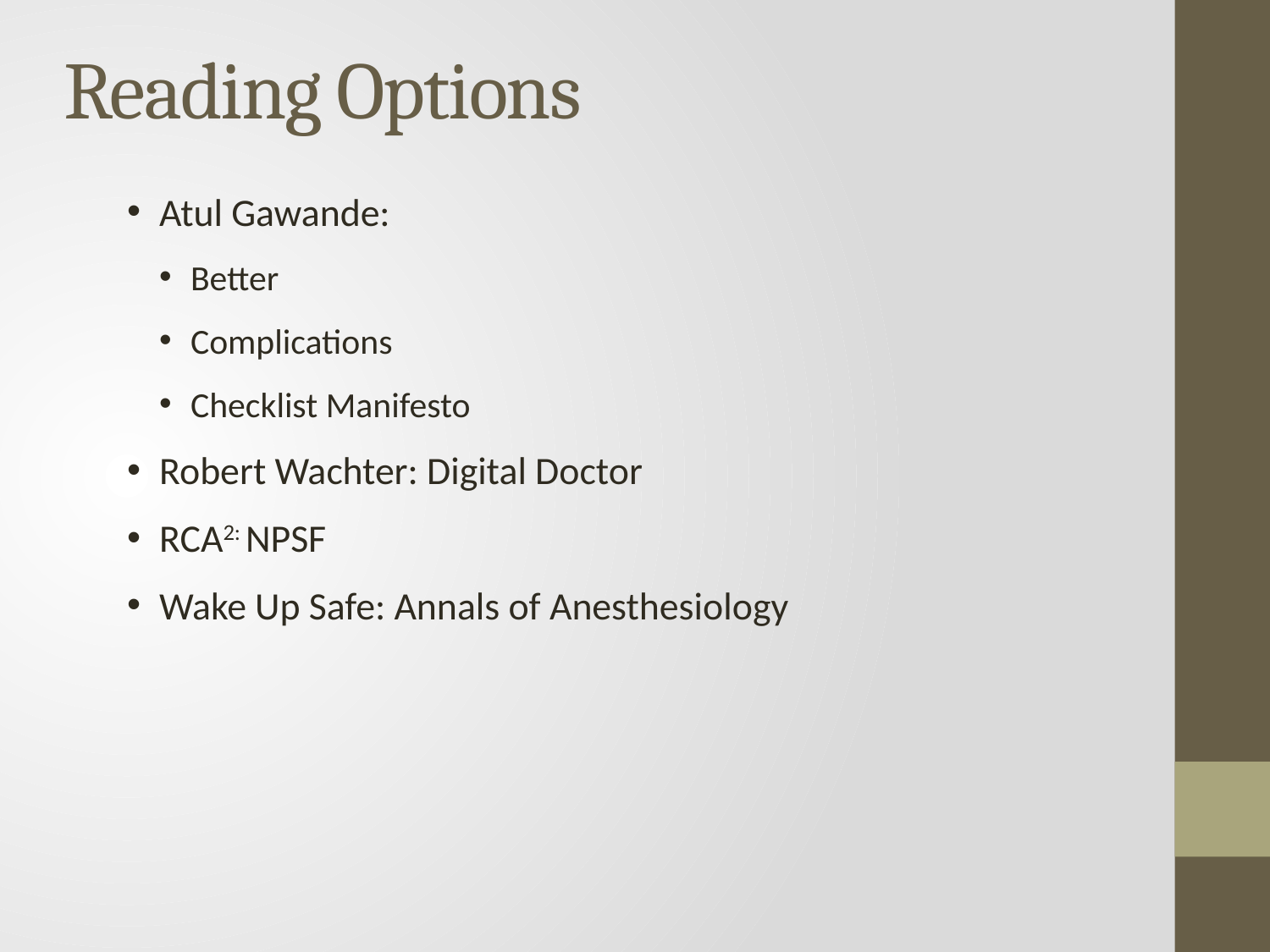

# Reading Options
Atul Gawande:
Better
Complications
Checklist Manifesto
Robert Wachter: Digital Doctor
RCA2: NPSF
Wake Up Safe: Annals of Anesthesiology
