# Supplementary material for: Safety in Numbers: Successful Student-Approved Case-Based Interprofessional Safety Workshop Utilizing Simulated Real-Life Safety Cases
Source: MedEdPORTAL. 2020 Jan 31;16:10874. doi: 10.15766/mep_2374-8265.10874 (PMC7065299; doi:10.15766/mep_2374-8265.10874)
Supplement: Supplementary file 1 — A. Pre- & Postevent Surveys.docx B. IPE Safety Workshop Agenda.docx C. RCA AM Session Facilitator Guide.docx D. RCA AM Session Facilitator Annotated Case Time Line.docx E. RCA AM Session Student Case Time Line.docx F. RCA AM Session Interviewee Scripts.docx G. RCA AM Session Patient Background & EWS Info.docx H. RCA AM Session Media - Radiology.docx I. RCA AM Session Media - Oxygen Tanks.docx J. Corrective Action PM Session Facilitator Guide.docx K. Corrective Action PM Session Effectiveness Chart.docx L. Corrective Action PM Session Worksheet.docx M. Executive Case Summary.docx N. Large-Group Lecture Schedule & Topic List.docx O. PPT 1 - Contributing to a Culture of Safety.pptx P. PPT 2 - Systems Improvement.pptx Q. PPT 3 - Impact of Students and Residents on QI.pptx R. PPT 4 - Presentation of Safety Case.pptx S. PPT 5 - Disclosing Medical Errors.pptx T. PPT 6 - Training for Resilience.pptx U. PPT 7 - Introduction to Improvement Plans.pptx V. Facilitator Postworkshop Survey.docx [file mep-16-10874-s001.zip › Q. PPT 3 - Impact of Students and Residents on QI.pptx]

## Slide 1
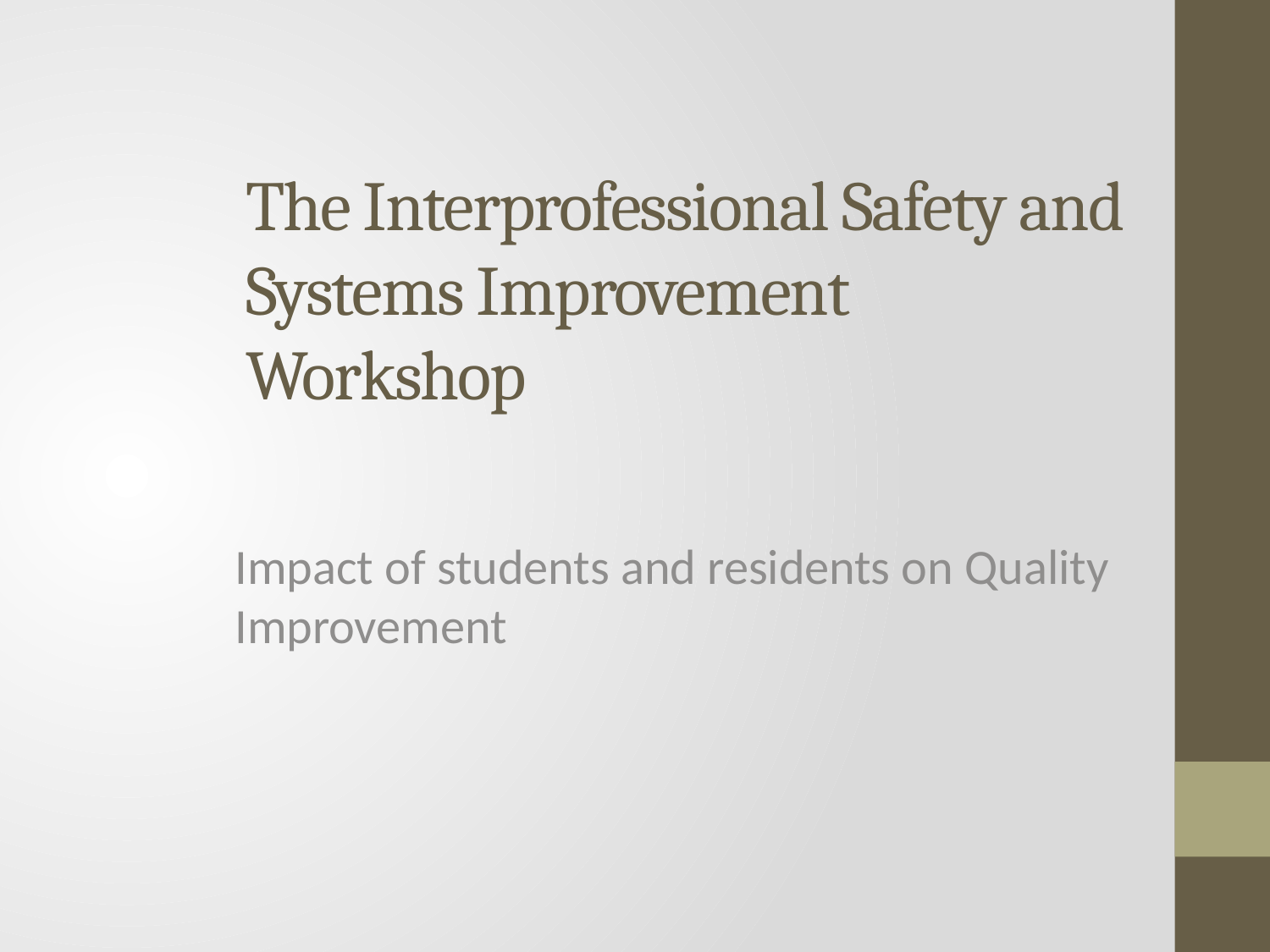

# The Interprofessional Safety and Systems Improvement Workshop
Impact of students and residents on Quality Improvement

## Slide 2
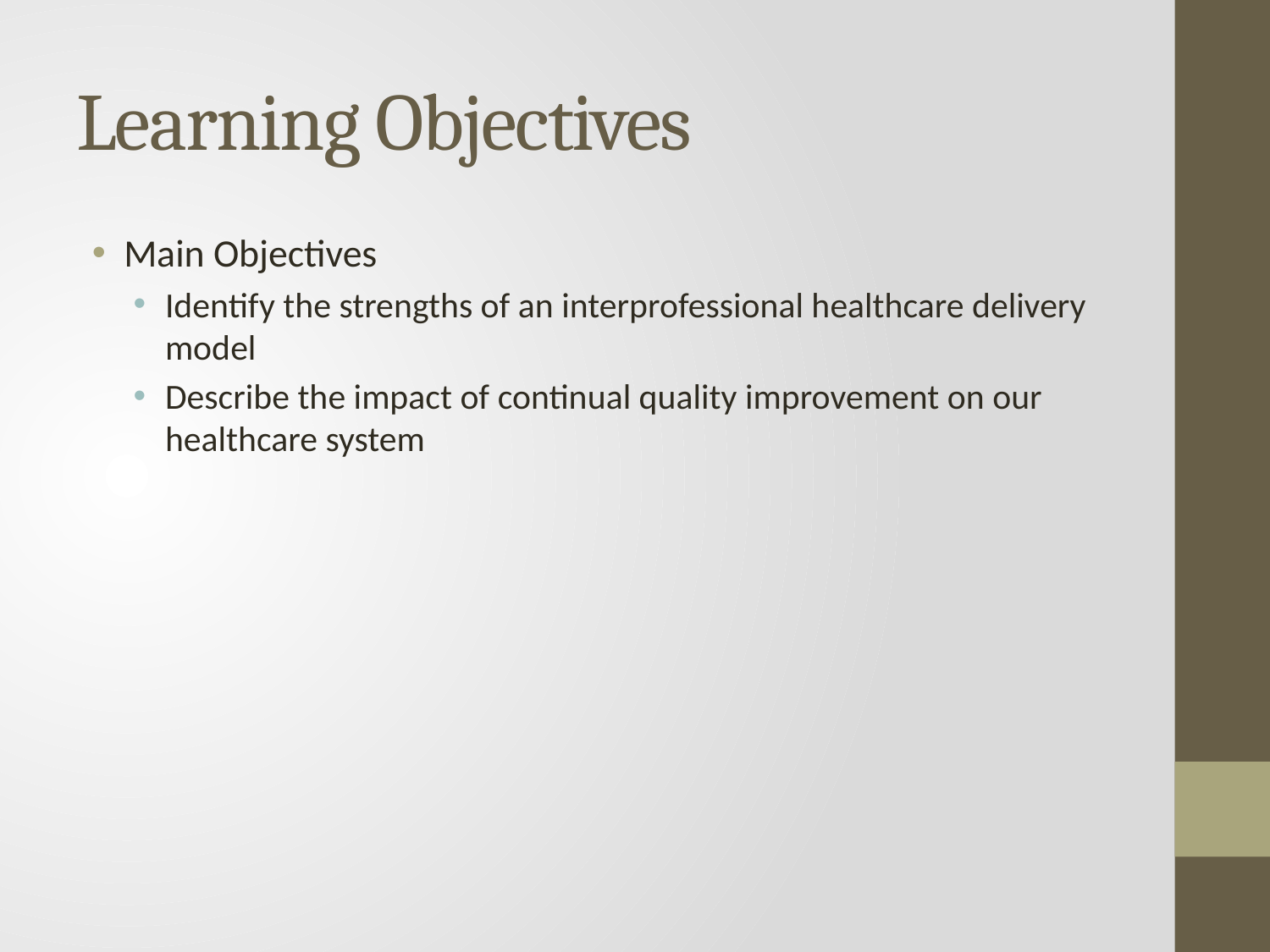

# Learning Objectives
Main Objectives
Identify the strengths of an interprofessional healthcare delivery model
Describe the impact of continual quality improvement on our healthcare system

## Slide 3
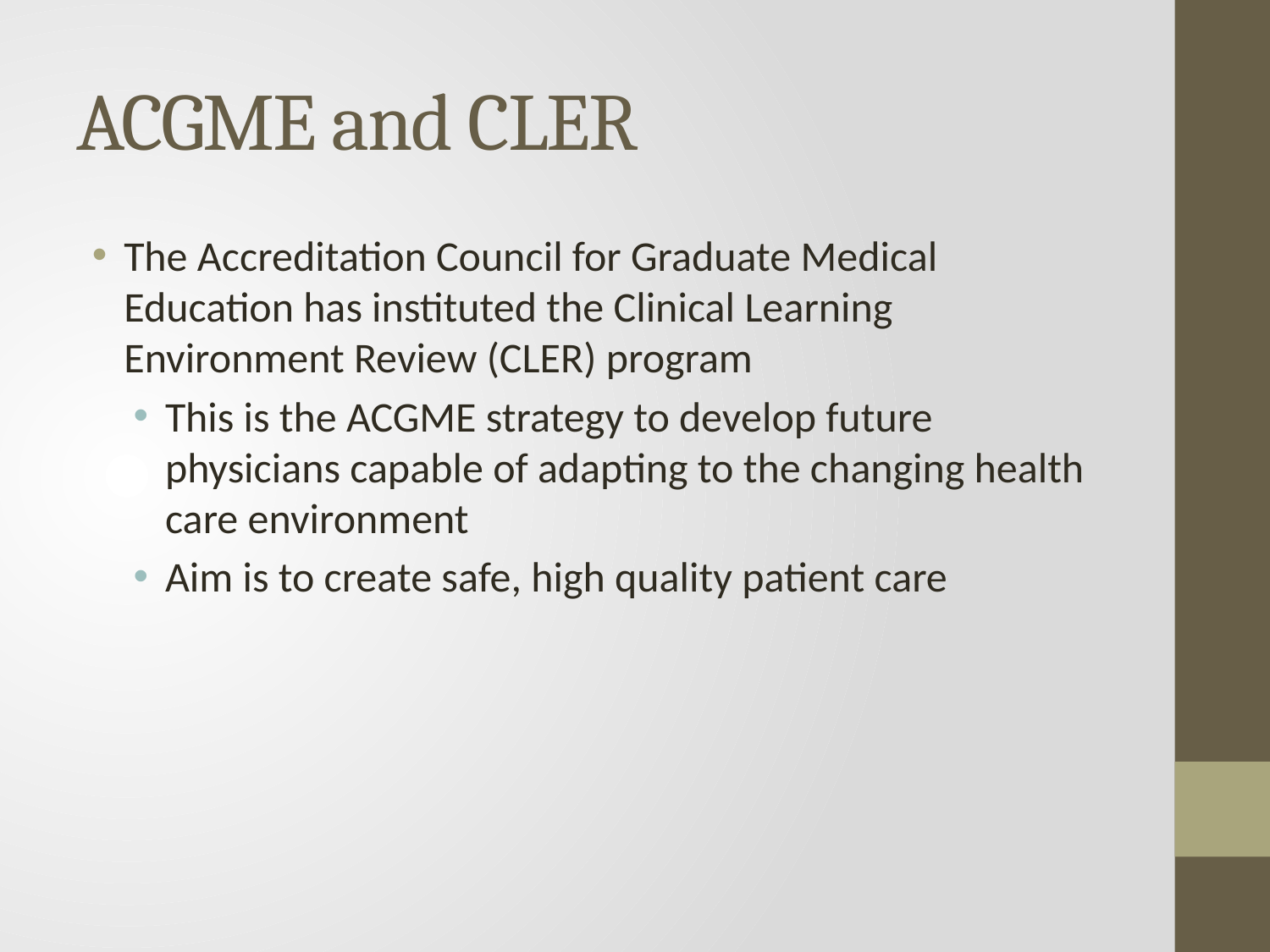

# ACGME and CLER
The Accreditation Council for Graduate Medical Education has instituted the Clinical Learning Environment Review (CLER) program
This is the ACGME strategy to develop future physicians capable of adapting to the changing health care environment
Aim is to create safe, high quality patient care

## Slide 4
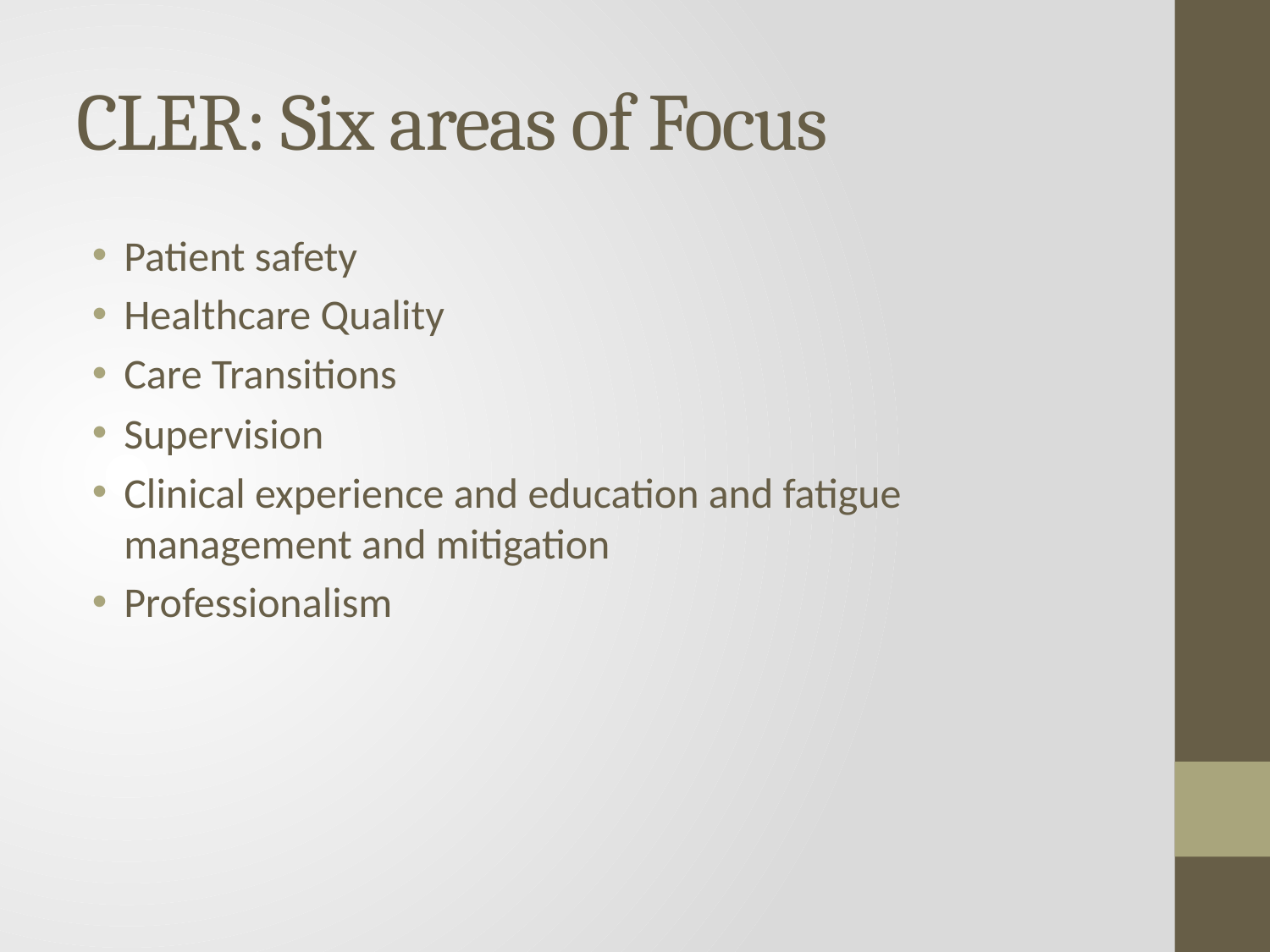

# CLER: Six areas of Focus
Patient safety
Healthcare Quality
Care Transitions
Supervision
Clinical experience and education and fatigue management and mitigation
Professionalism

## Slide 5
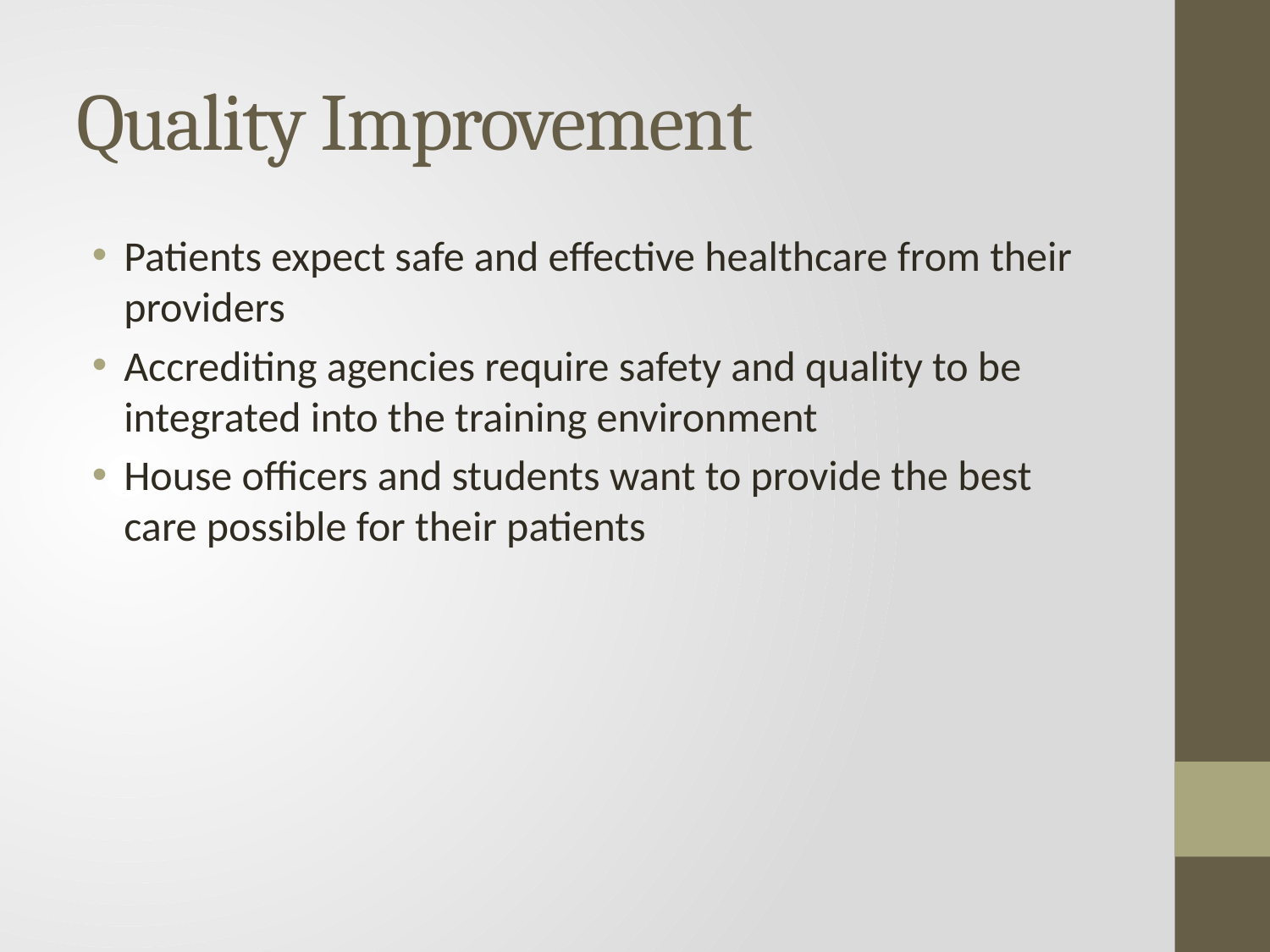

# Quality Improvement
Patients expect safe and effective healthcare from their providers
Accrediting agencies require safety and quality to be integrated into the training environment
House officers and students want to provide the best care possible for their patients

## Slide 6
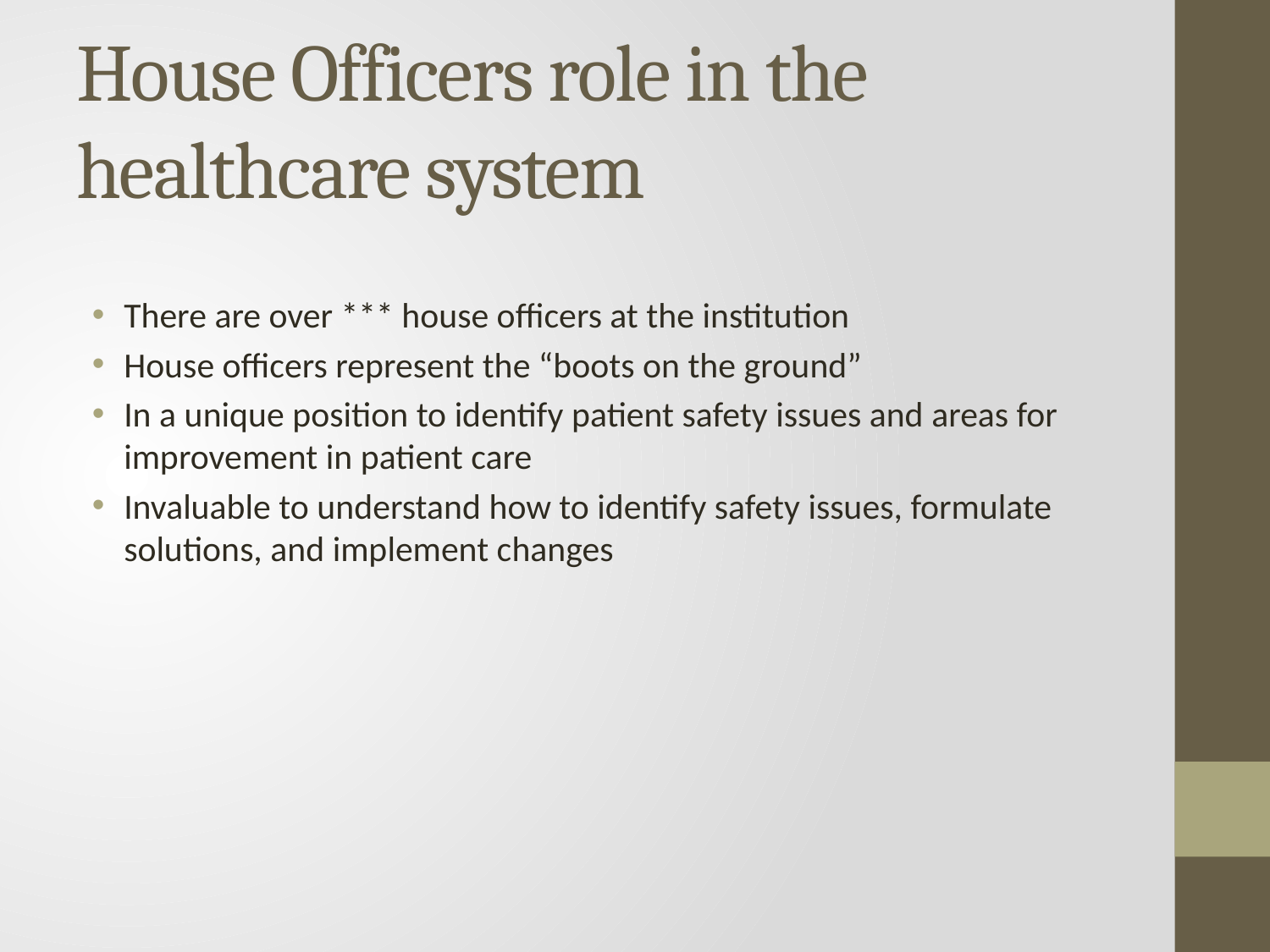

# House Officers role in the healthcare system
There are over *** house officers at the institution
House officers represent the “boots on the ground”
In a unique position to identify patient safety issues and areas for improvement in patient care
Invaluable to understand how to identify safety issues, formulate solutions, and implement changes

## Slide 7
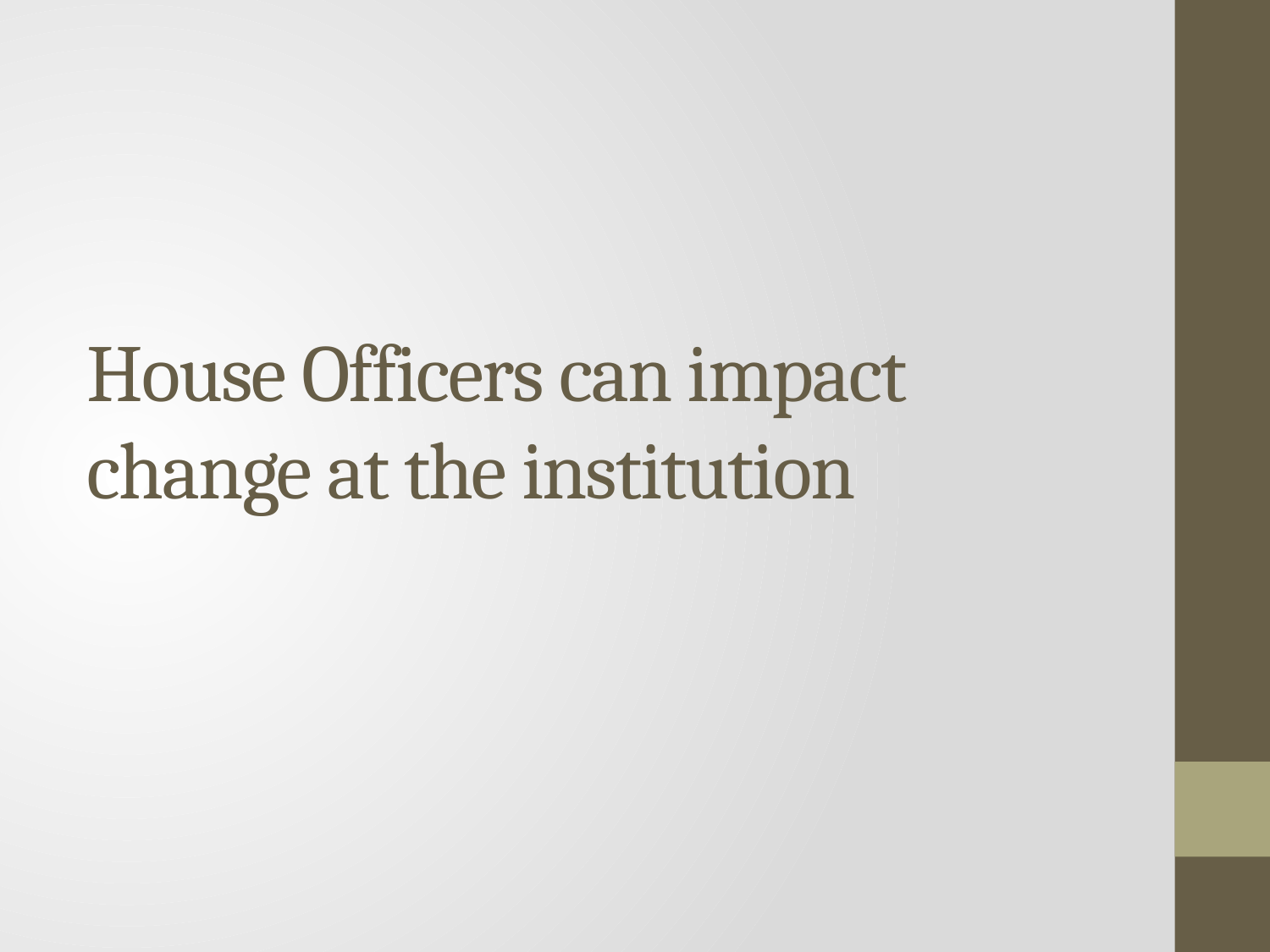

# House Officers can impact change at the institution

## Slide 8
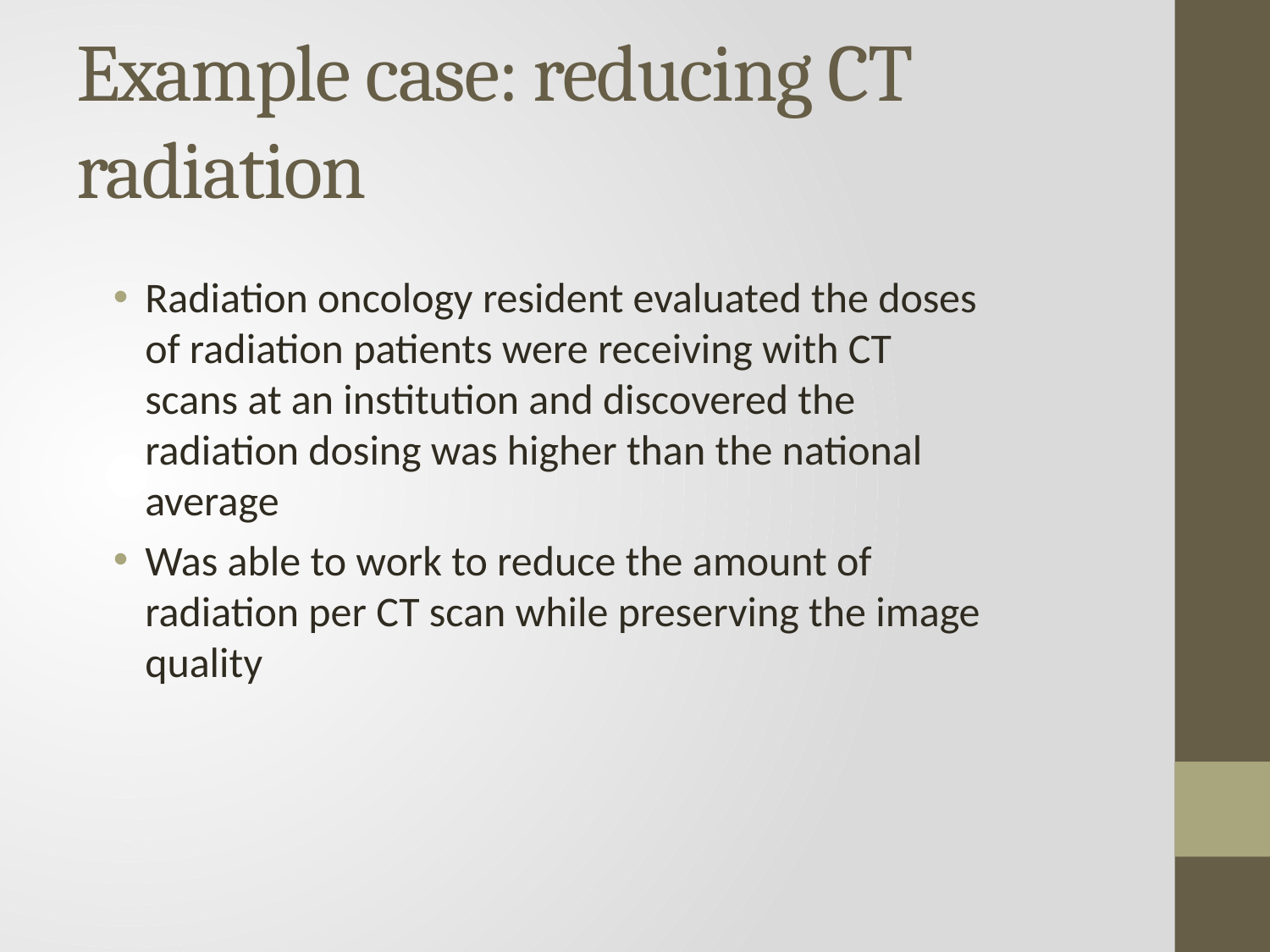

# Example case: reducing CT radiation
Radiation oncology resident evaluated the doses of radiation patients were receiving with CT scans at an institution and discovered the radiation dosing was higher than the national average
Was able to work to reduce the amount of radiation per CT scan while preserving the image quality

## Slide 9
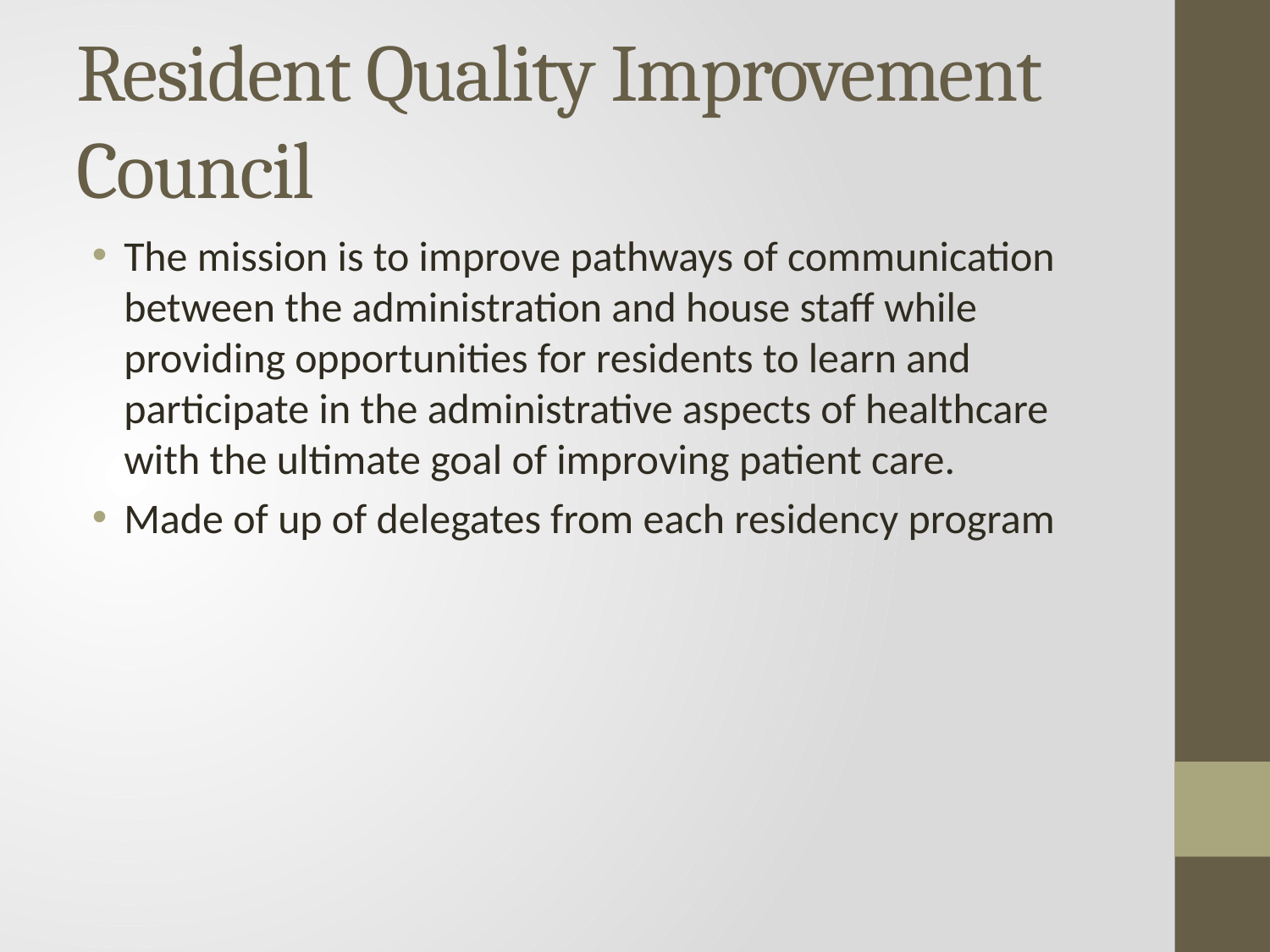

# Resident Quality Improvement Council
The mission is to improve pathways of communication between the administration and house staff while providing opportunities for residents to learn and participate in the administrative aspects of healthcare with the ultimate goal of improving patient care.
Made of up of delegates from each residency program

## Slide 10
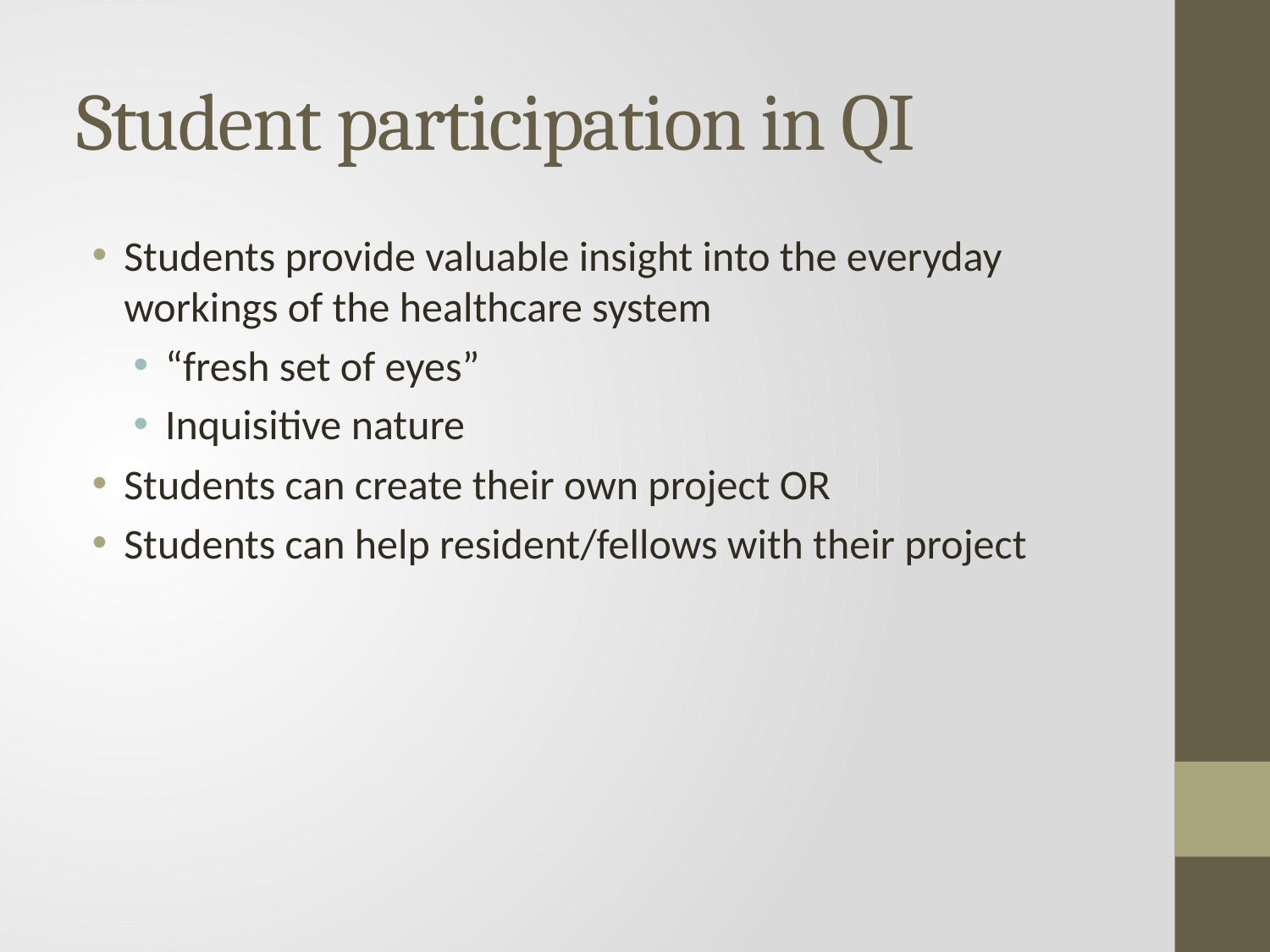

# Student participation in QI
Students provide valuable insight into the everyday workings of the healthcare system
“fresh set of eyes”
Inquisitive nature
Students can create their own project OR
Students can help resident/fellows with their project

## Slide 11
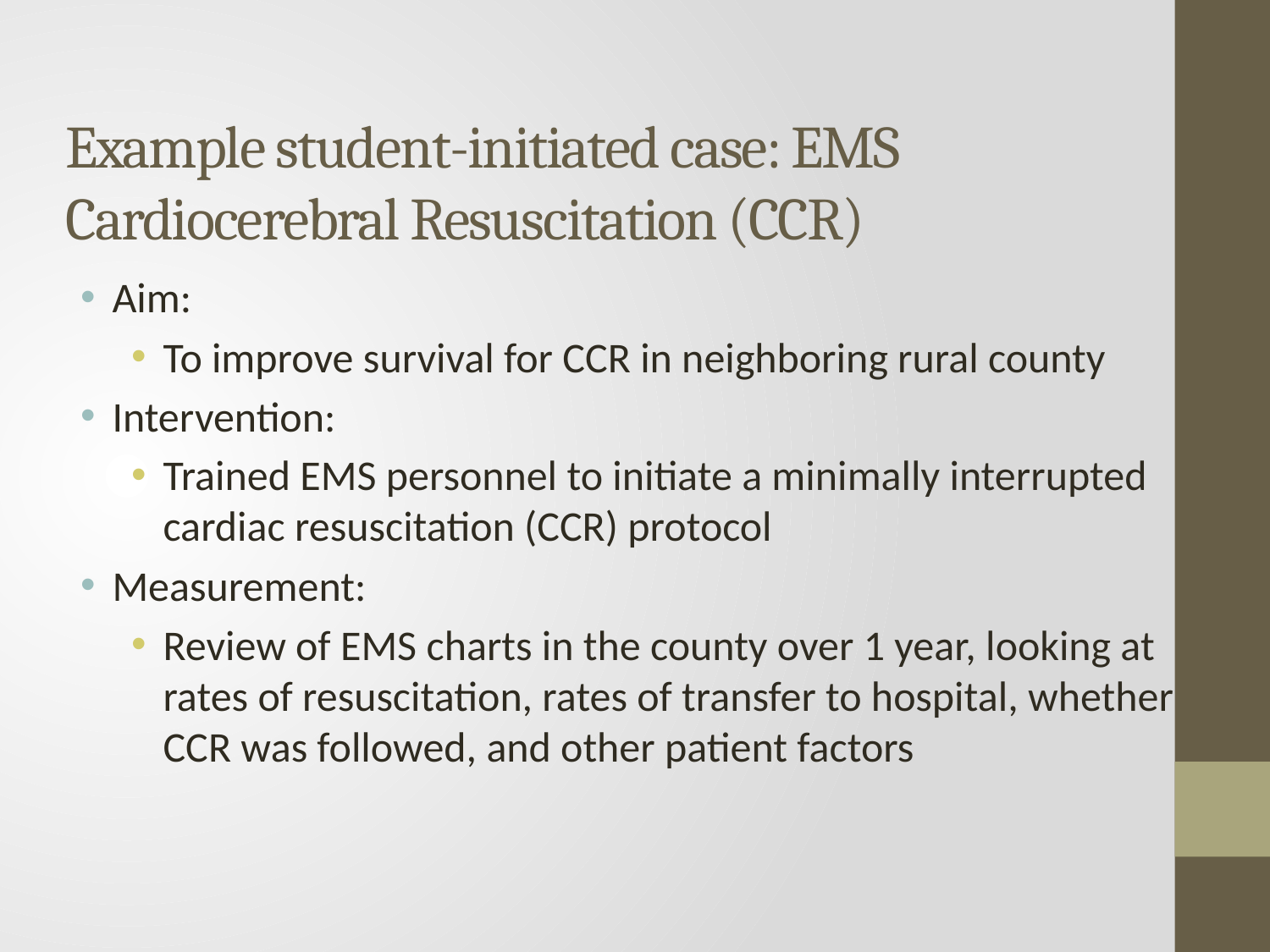

# Example student-initiated case: EMS Cardiocerebral Resuscitation (CCR)
Aim:
To improve survival for CCR in neighboring rural county
Intervention:
Trained EMS personnel to initiate a minimally interrupted cardiac resuscitation (CCR) protocol
Measurement:
Review of EMS charts in the county over 1 year, looking at rates of resuscitation, rates of transfer to hospital, whether CCR was followed, and other patient factors

## Slide 12
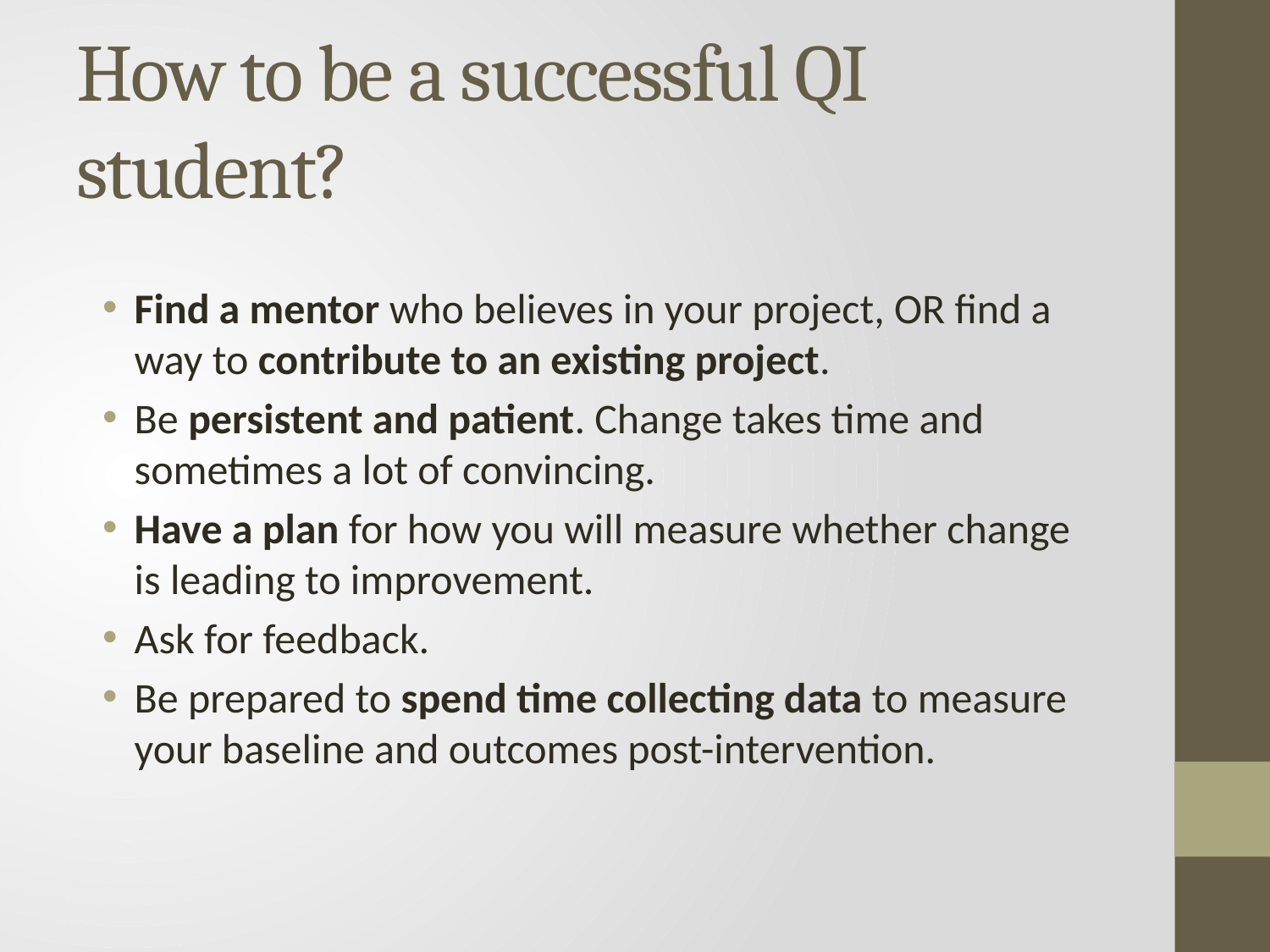

# How to be a successful QI student?
Find a mentor who believes in your project, OR find a way to contribute to an existing project.
Be persistent and patient. Change takes time and sometimes a lot of convincing.
Have a plan for how you will measure whether change is leading to improvement.
Ask for feedback.
Be prepared to spend time collecting data to measure your baseline and outcomes post-intervention.
