# Supplementary material for: Safety in Numbers: Successful Student-Approved Case-Based Interprofessional Safety Workshop Utilizing Simulated Real-Life Safety Cases
Source: MedEdPORTAL. 2020 Jan 31;16:10874. doi: 10.15766/mep_2374-8265.10874 (PMC7065299; doi:10.15766/mep_2374-8265.10874)
Supplement: Supplementary file 1 — A. Pre- & Postevent Surveys.docx B. IPE Safety Workshop Agenda.docx C. RCA AM Session Facilitator Guide.docx D. RCA AM Session Facilitator Annotated Case Time Line.docx E. RCA AM Session Student Case Time Line.docx F. RCA AM Session Interviewee Scripts.docx G. RCA AM Session Patient Background & EWS Info.docx H. RCA AM Session Media - Radiology.docx I. RCA AM Session Media - Oxygen Tanks.docx J. Corrective Action PM Session Facilitator Guide.docx K. Corrective Action PM Session Effectiveness Chart.docx L. Corrective Action PM Session Worksheet.docx M. Executive Case Summary.docx N. Large-Group Lecture Schedule & Topic List.docx O. PPT 1 - Contributing to a Culture of Safety.pptx P. PPT 2 - Systems Improvement.pptx Q. PPT 3 - Impact of Students and Residents on QI.pptx R. PPT 4 - Presentation of Safety Case.pptx S. PPT 5 - Disclosing Medical Errors.pptx T. PPT 6 - Training for Resilience.pptx U. PPT 7 - Introduction to Improvement Plans.pptx V. Facilitator Postworkshop Survey.docx [file mep-16-10874-s001.zip › R. PPT 4 - Presentation of Safety Case.pptx]

## Slide 1
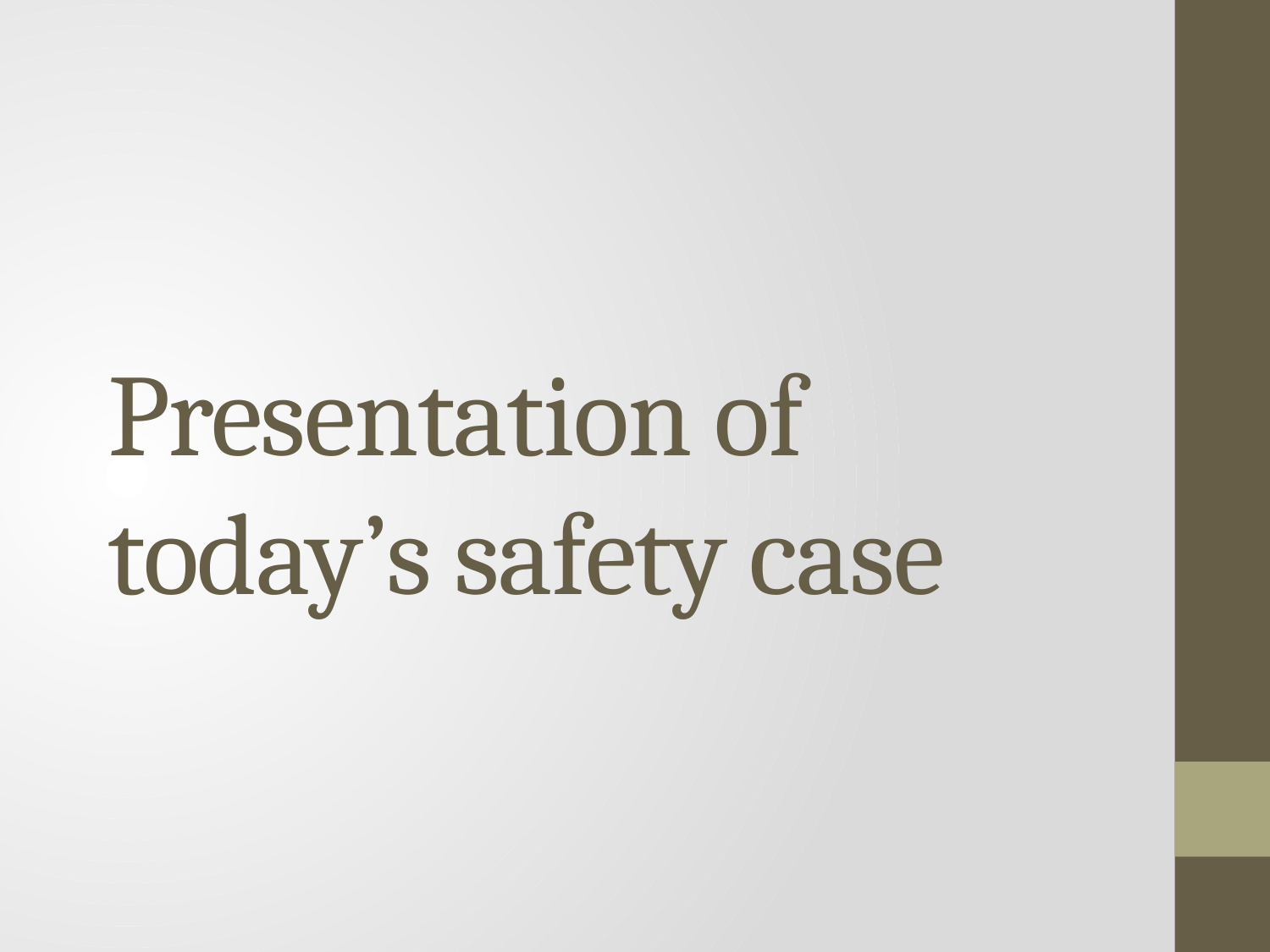

# Presentation of today’s safety case

## Slide 2
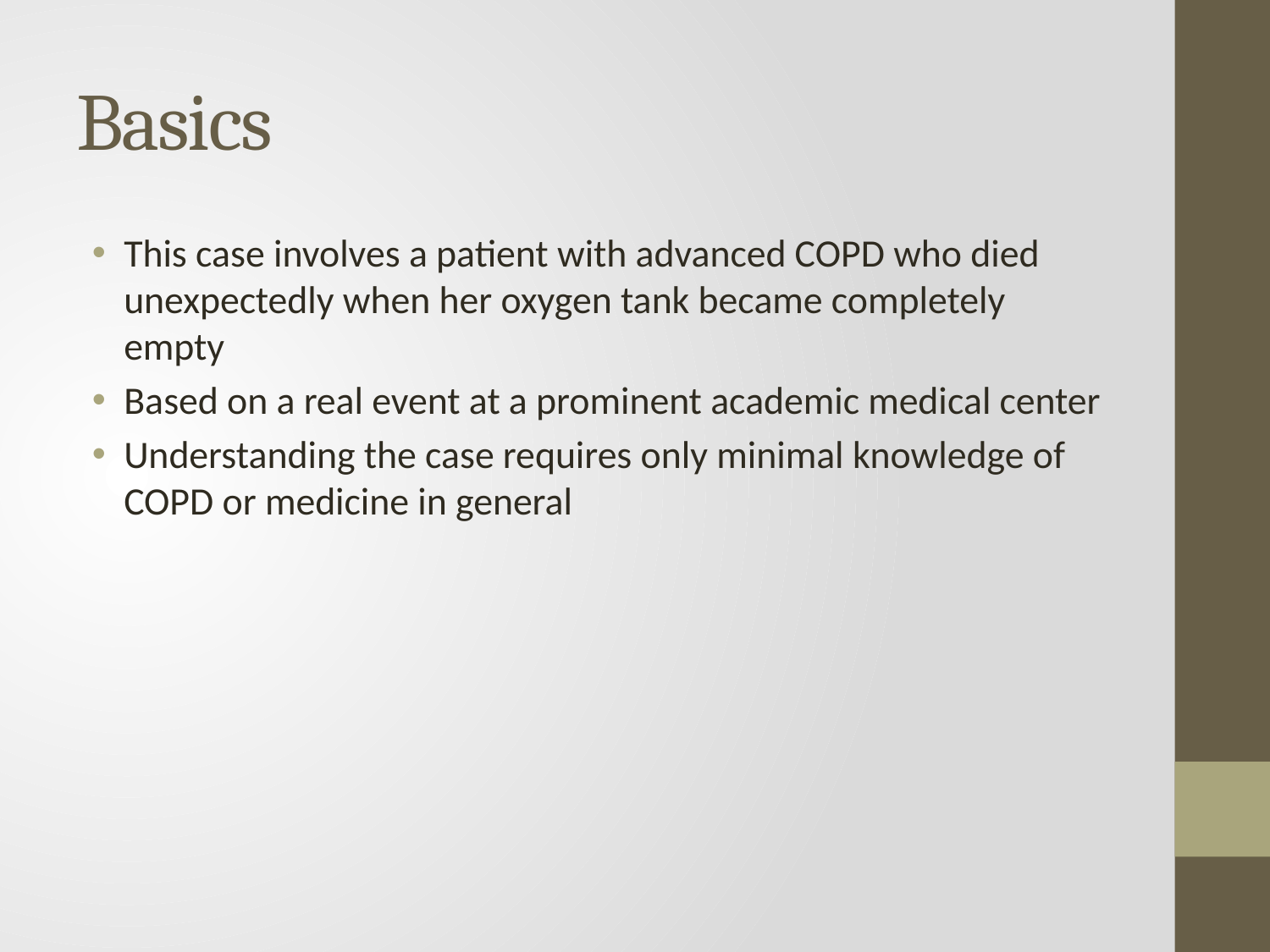

# Basics
This case involves a patient with advanced COPD who died unexpectedly when her oxygen tank became completely empty
Based on a real event at a prominent academic medical center
Understanding the case requires only minimal knowledge of COPD or medicine in general

## Slide 3
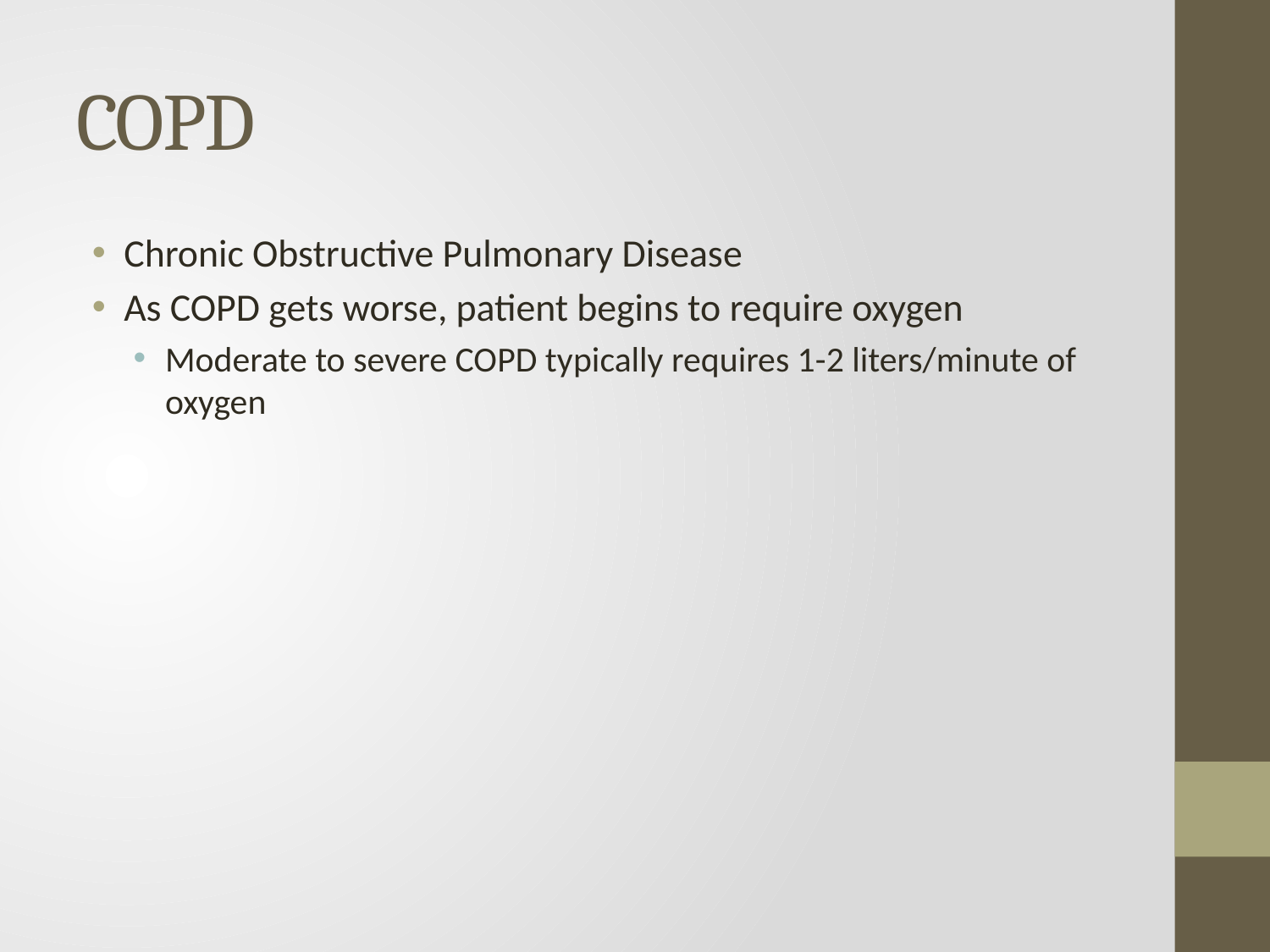

# COPD
Chronic Obstructive Pulmonary Disease
As COPD gets worse, patient begins to require oxygen
Moderate to severe COPD typically requires 1-2 liters/minute of oxygen

## Slide 4
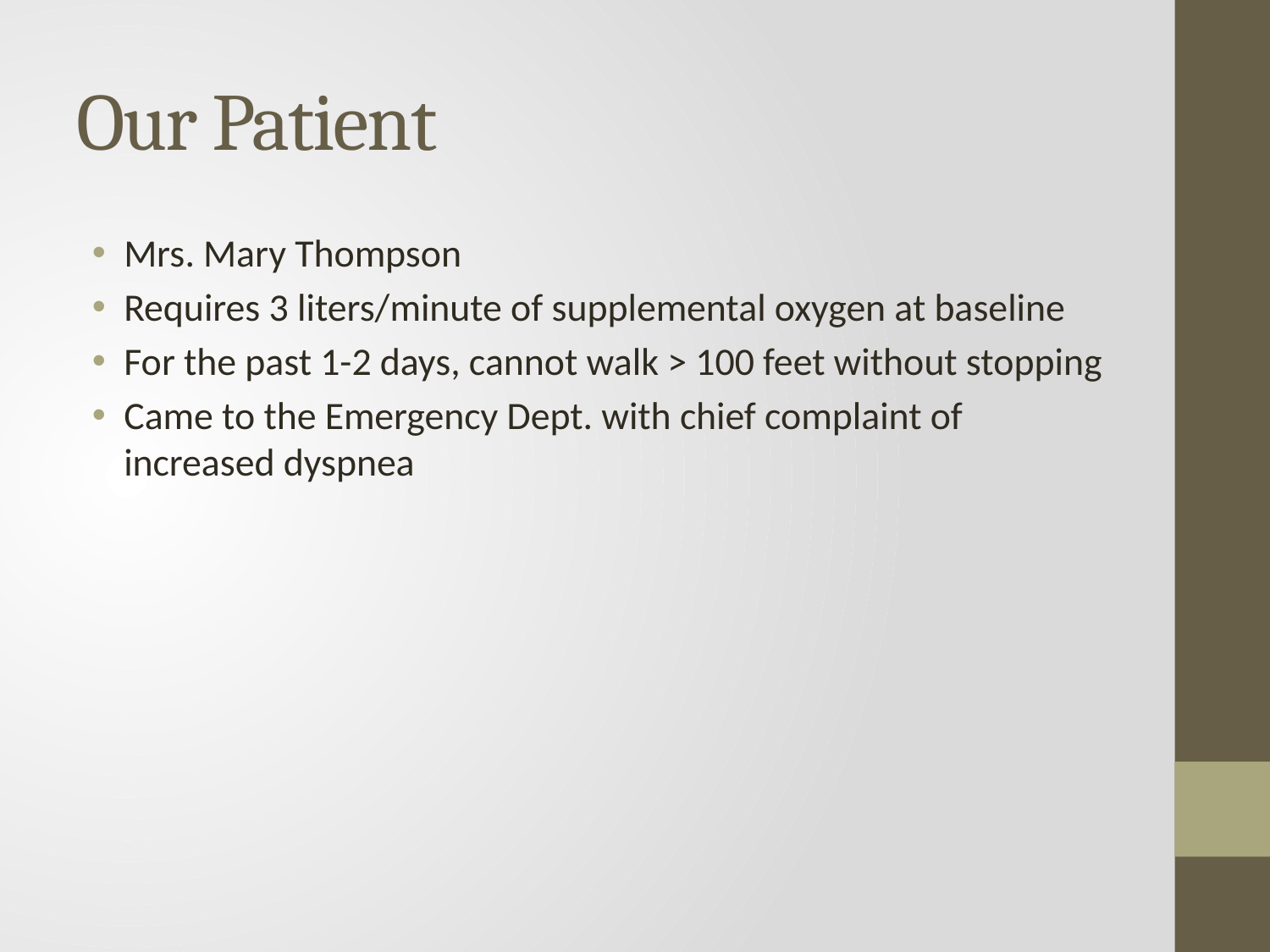

# Our Patient
Mrs. Mary Thompson
Requires 3 liters/minute of supplemental oxygen at baseline
For the past 1-2 days, cannot walk > 100 feet without stopping
Came to the Emergency Dept. with chief complaint of increased dyspnea

## Slide 5
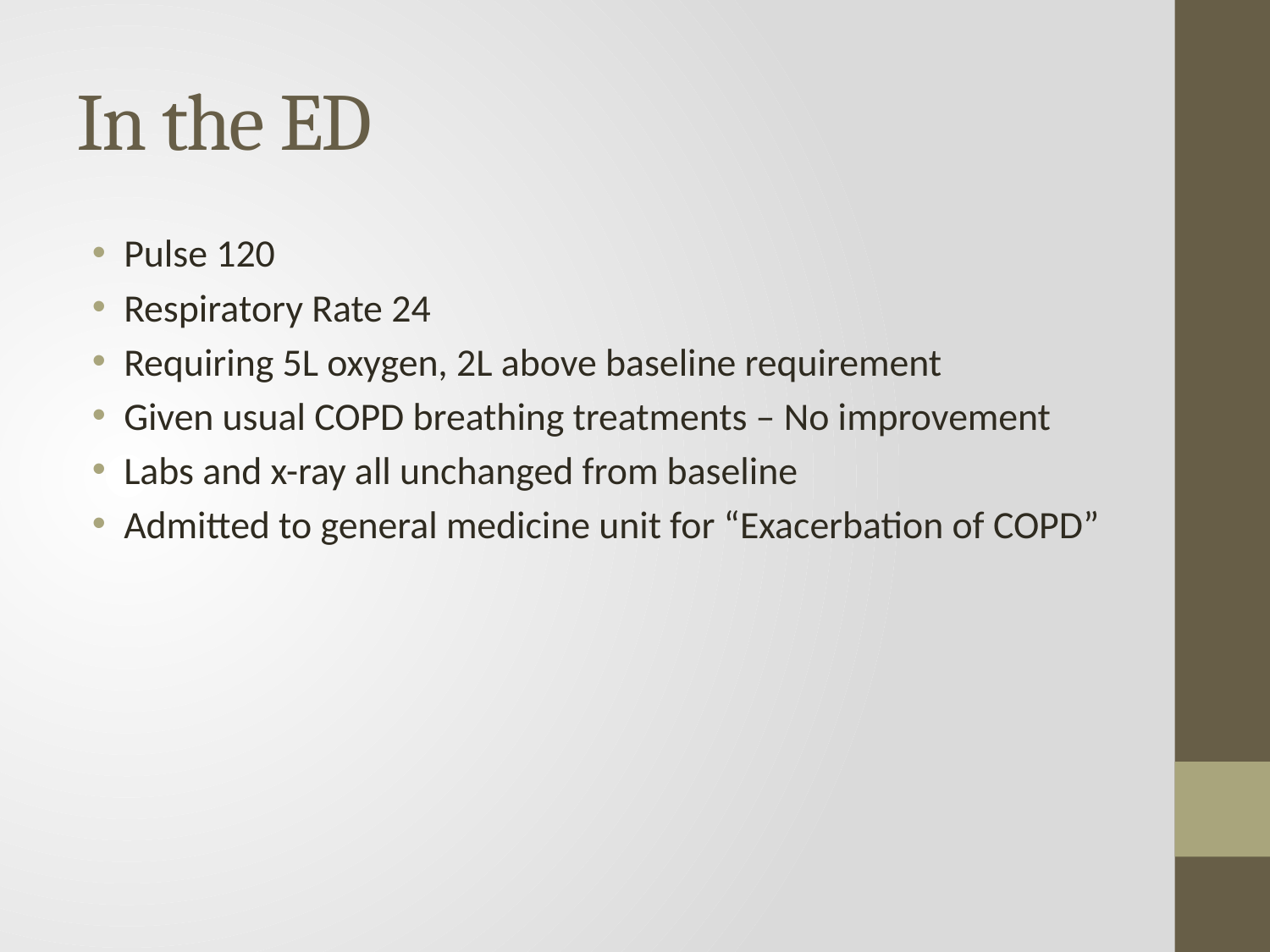

# In the ED
Pulse 120
Respiratory Rate 24
Requiring 5L oxygen, 2L above baseline requirement
Given usual COPD breathing treatments – No improvement
Labs and x-ray all unchanged from baseline
Admitted to general medicine unit for “Exacerbation of COPD”

## Slide 6
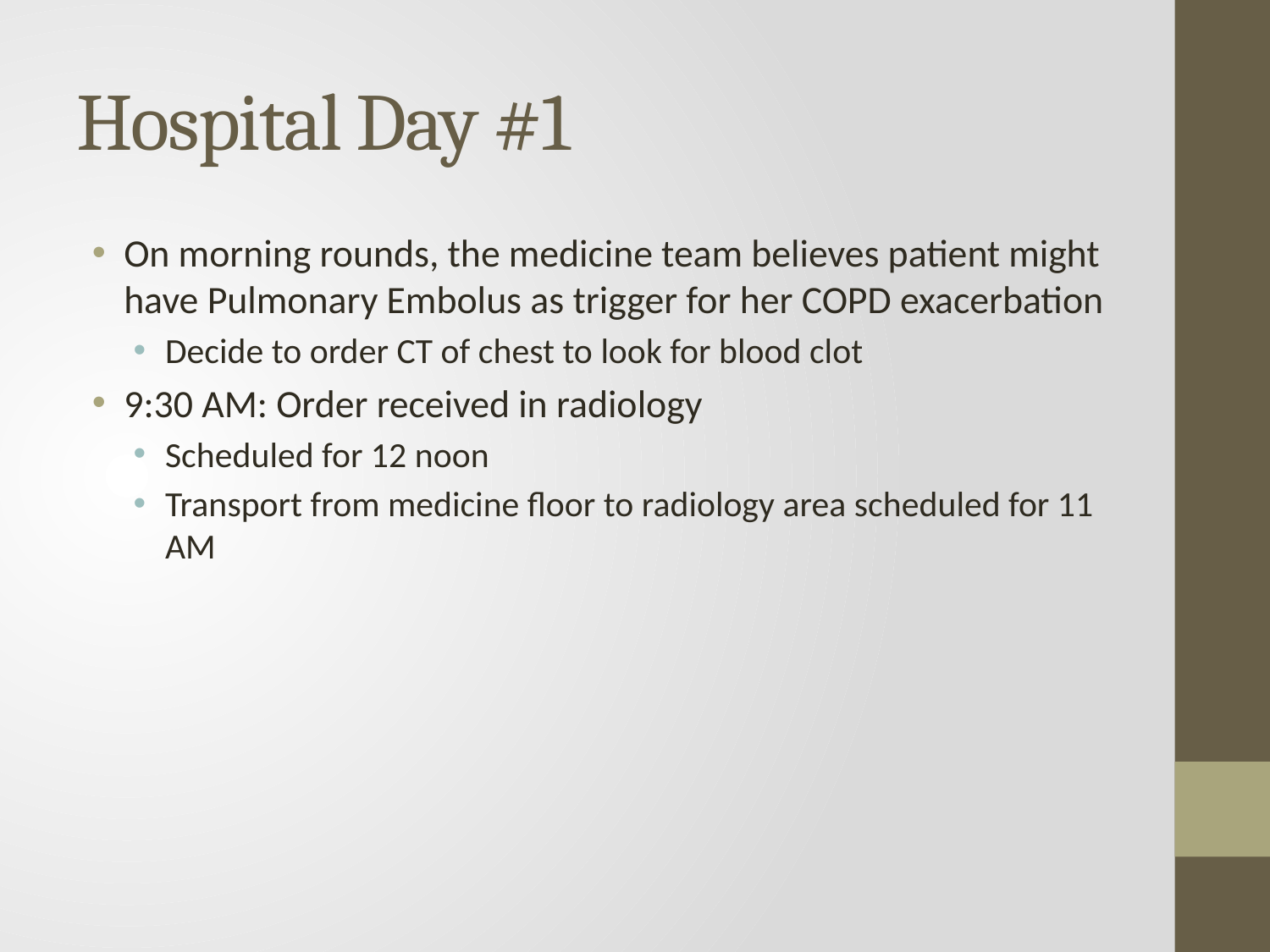

# Hospital Day #1
On morning rounds, the medicine team believes patient might have Pulmonary Embolus as trigger for her COPD exacerbation
Decide to order CT of chest to look for blood clot
9:30 AM: Order received in radiology
Scheduled for 12 noon
Transport from medicine floor to radiology area scheduled for 11 AM

## Slide 7
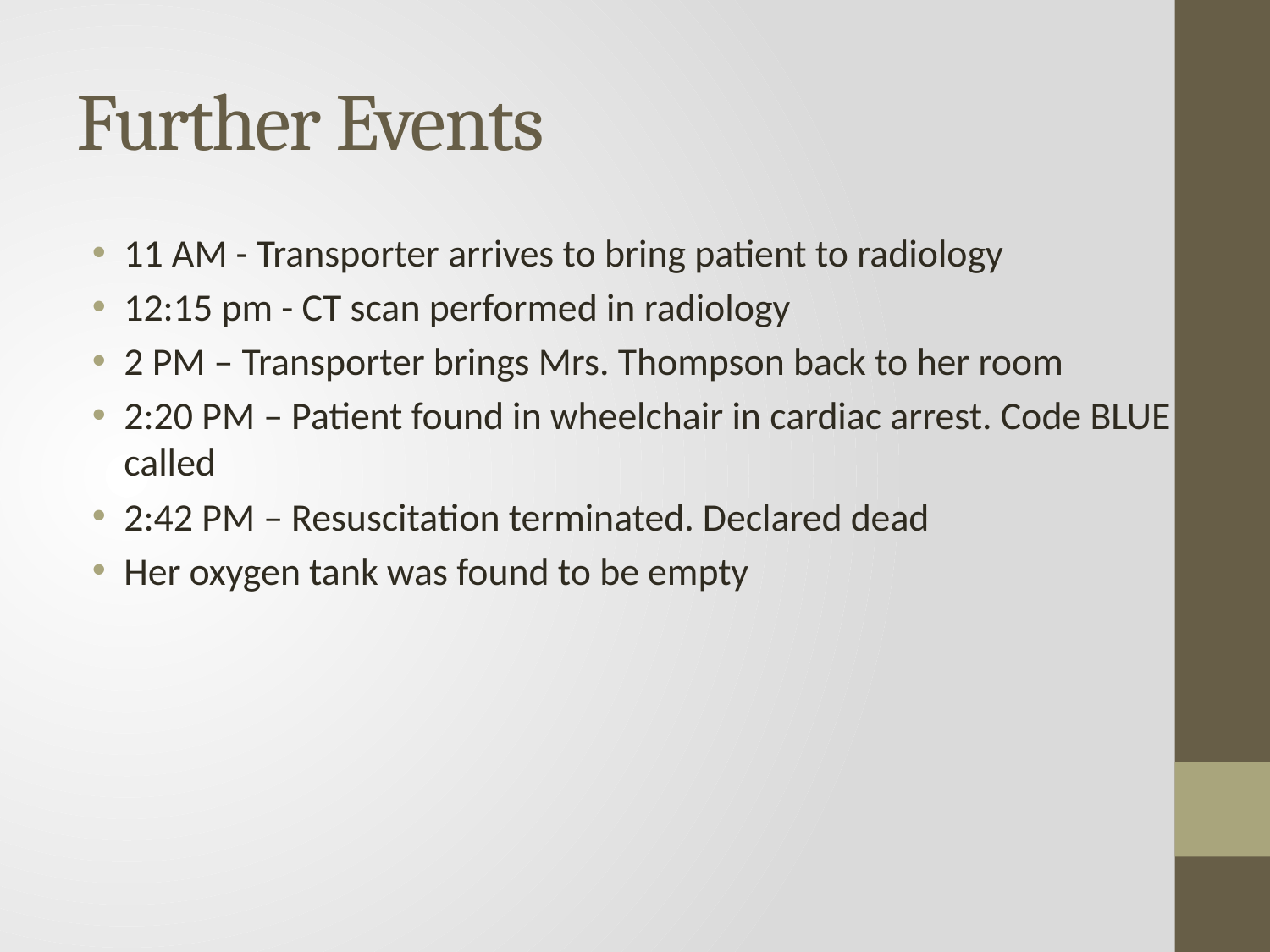

# Further Events
11 AM - Transporter arrives to bring patient to radiology
12:15 pm - CT scan performed in radiology
2 PM – Transporter brings Mrs. Thompson back to her room
2:20 PM – Patient found in wheelchair in cardiac arrest. Code BLUE called
2:42 PM – Resuscitation terminated. Declared dead
Her oxygen tank was found to be empty

## Slide 8
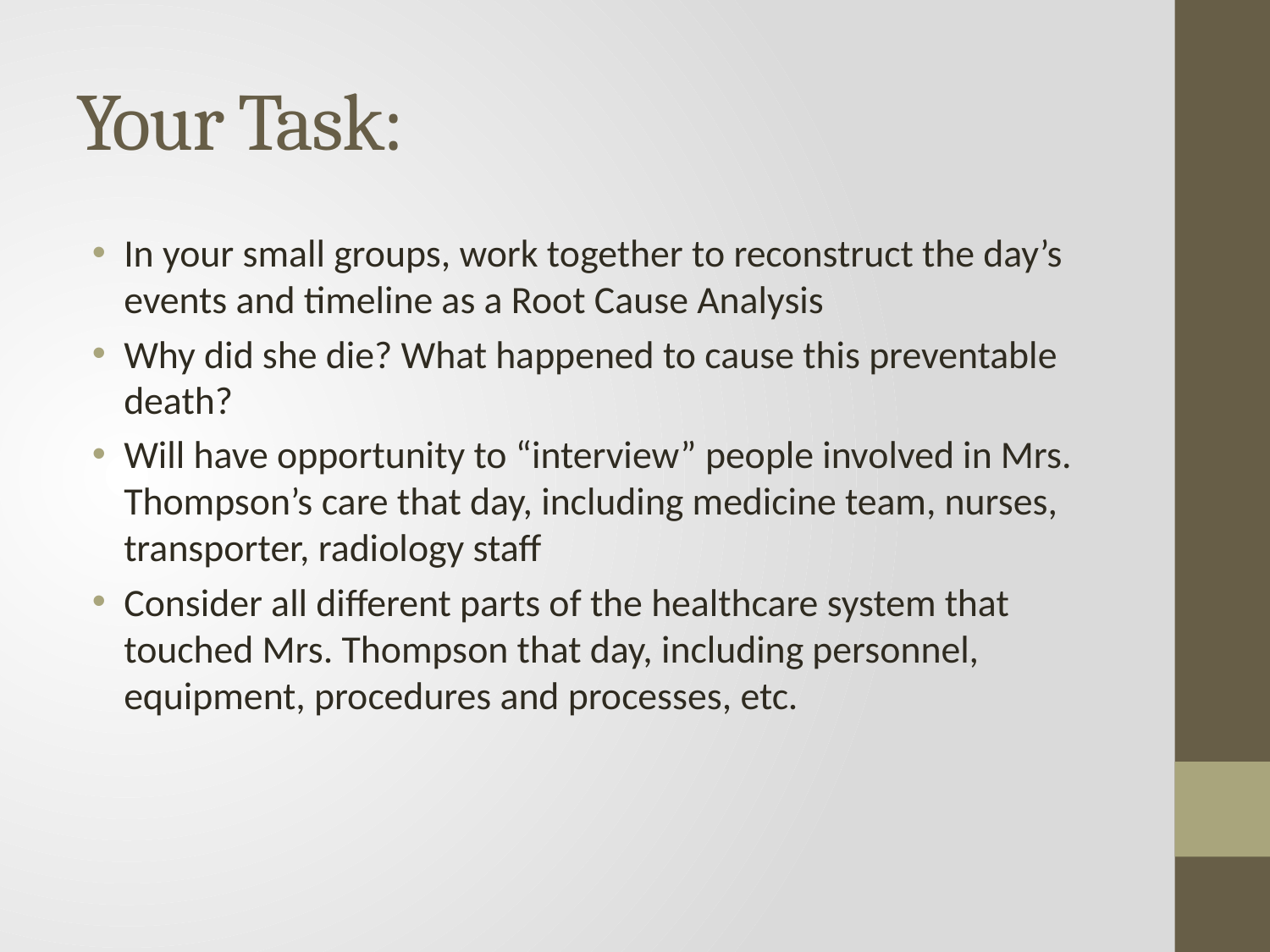

# Your Task:
In your small groups, work together to reconstruct the day’s events and timeline as a Root Cause Analysis
Why did she die? What happened to cause this preventable death?
Will have opportunity to “interview” people involved in Mrs. Thompson’s care that day, including medicine team, nurses, transporter, radiology staff
Consider all different parts of the healthcare system that touched Mrs. Thompson that day, including personnel, equipment, procedures and processes, etc.
