# Supplementary material for: Safety in Numbers: Successful Student-Approved Case-Based Interprofessional Safety Workshop Utilizing Simulated Real-Life Safety Cases
Source: MedEdPORTAL. 2020 Jan 31;16:10874. doi: 10.15766/mep_2374-8265.10874 (PMC7065299; doi:10.15766/mep_2374-8265.10874)
Supplement: Supplementary file 1 — A. Pre- & Postevent Surveys.docx B. IPE Safety Workshop Agenda.docx C. RCA AM Session Facilitator Guide.docx D. RCA AM Session Facilitator Annotated Case Time Line.docx E. RCA AM Session Student Case Time Line.docx F. RCA AM Session Interviewee Scripts.docx G. RCA AM Session Patient Background & EWS Info.docx H. RCA AM Session Media - Radiology.docx I. RCA AM Session Media - Oxygen Tanks.docx J. Corrective Action PM Session Facilitator Guide.docx K. Corrective Action PM Session Effectiveness Chart.docx L. Corrective Action PM Session Worksheet.docx M. Executive Case Summary.docx N. Large-Group Lecture Schedule & Topic List.docx O. PPT 1 - Contributing to a Culture of Safety.pptx P. PPT 2 - Systems Improvement.pptx Q. PPT 3 - Impact of Students and Residents on QI.pptx R. PPT 4 - Presentation of Safety Case.pptx S. PPT 5 - Disclosing Medical Errors.pptx T. PPT 6 - Training for Resilience.pptx U. PPT 7 - Introduction to Improvement Plans.pptx V. Facilitator Postworkshop Survey.docx [file mep-16-10874-s001.zip › S. PPT 5 - Disclosing Medical Errors.pptx]

## Slide 1
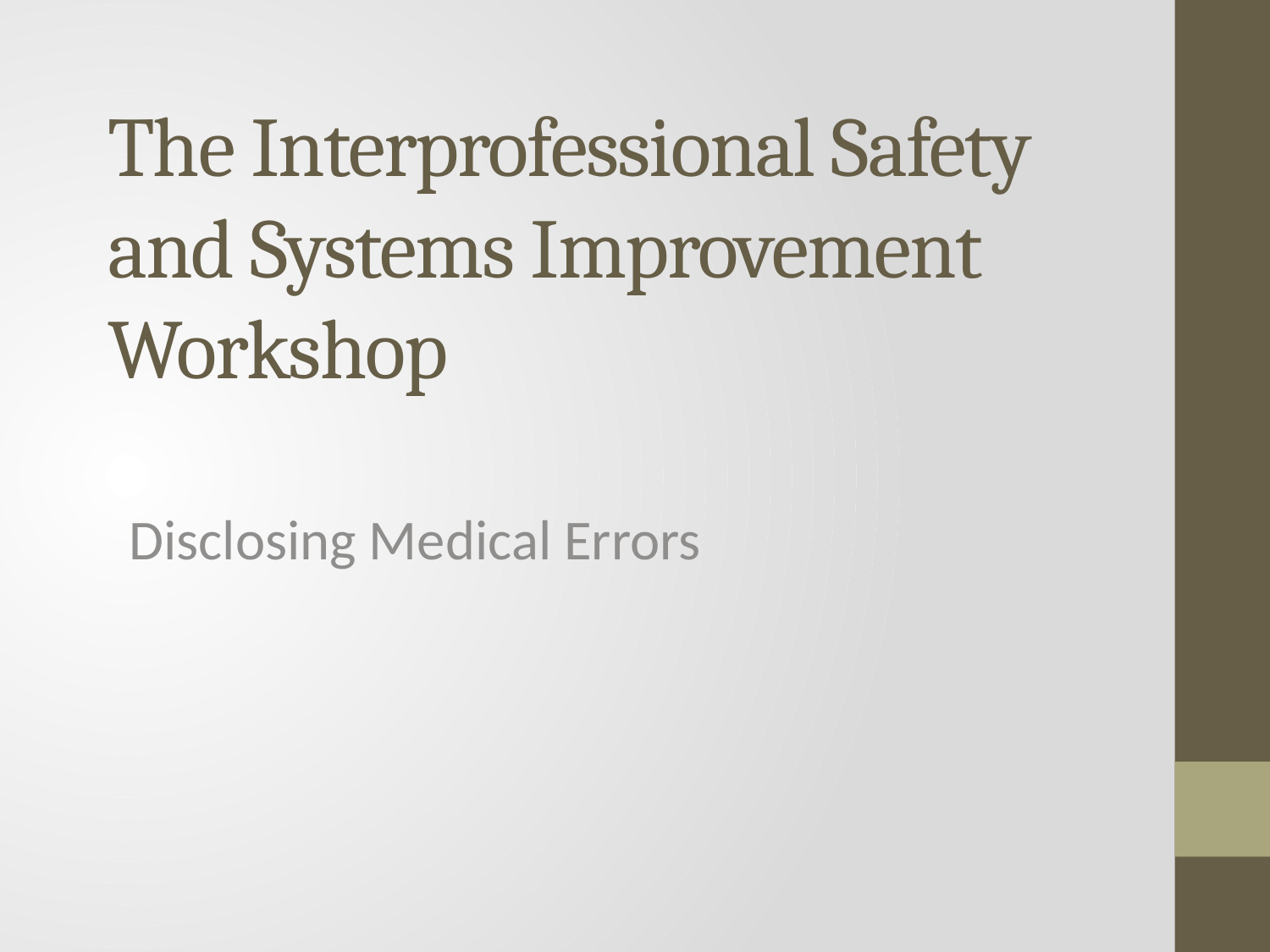

# The Interprofessional Safety and Systems Improvement Workshop
Disclosing Medical Errors

## Slide 2
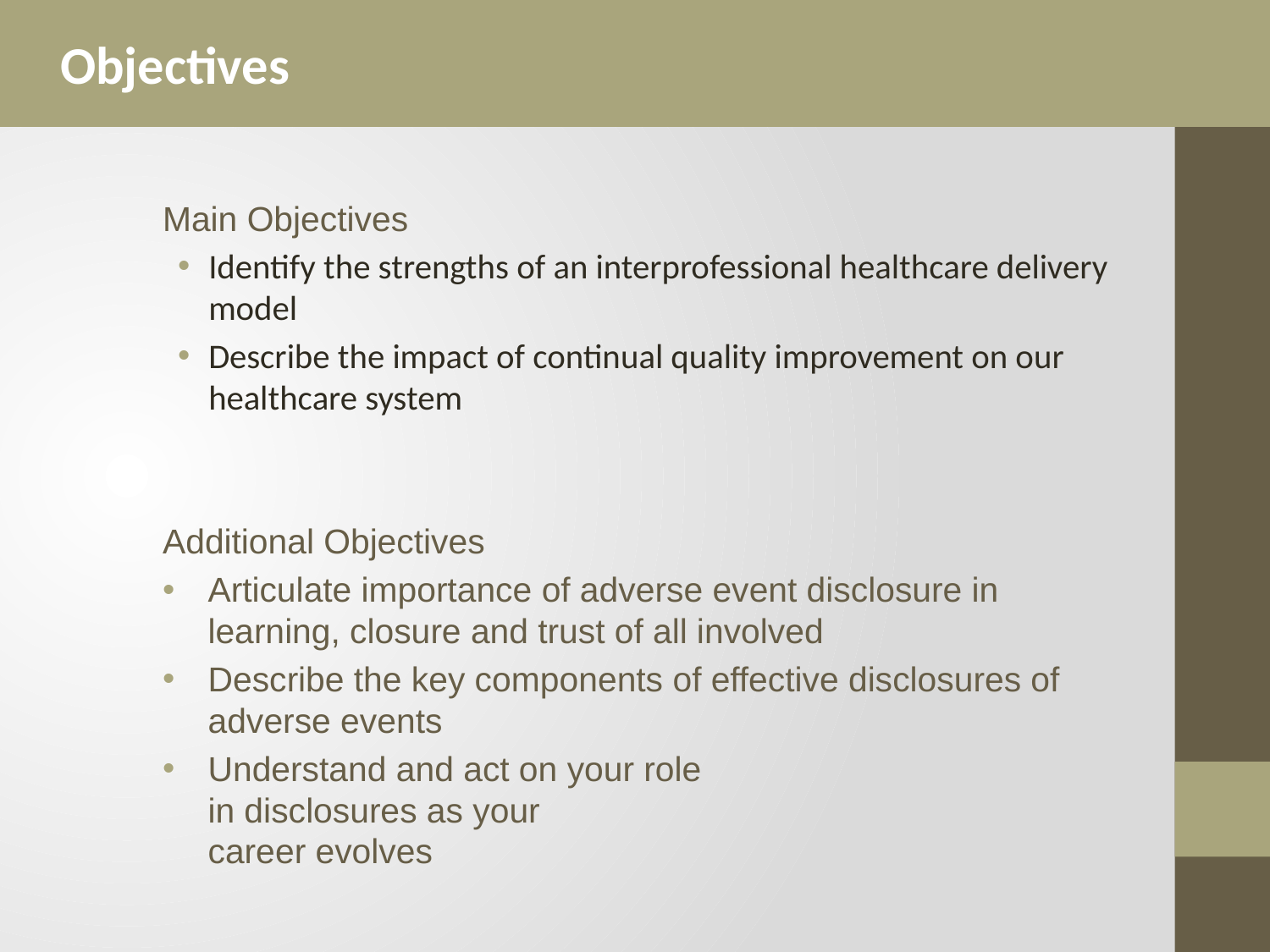

Objectives
Main Objectives
Identify the strengths of an interprofessional healthcare delivery model
Describe the impact of continual quality improvement on our healthcare system
Additional Objectives
Articulate importance of adverse event disclosure in learning, closure and trust of all involved
Describe the key components of effective disclosures of adverse events
Understand and act on your role
in disclosures as your
career evolves

## Slide 3
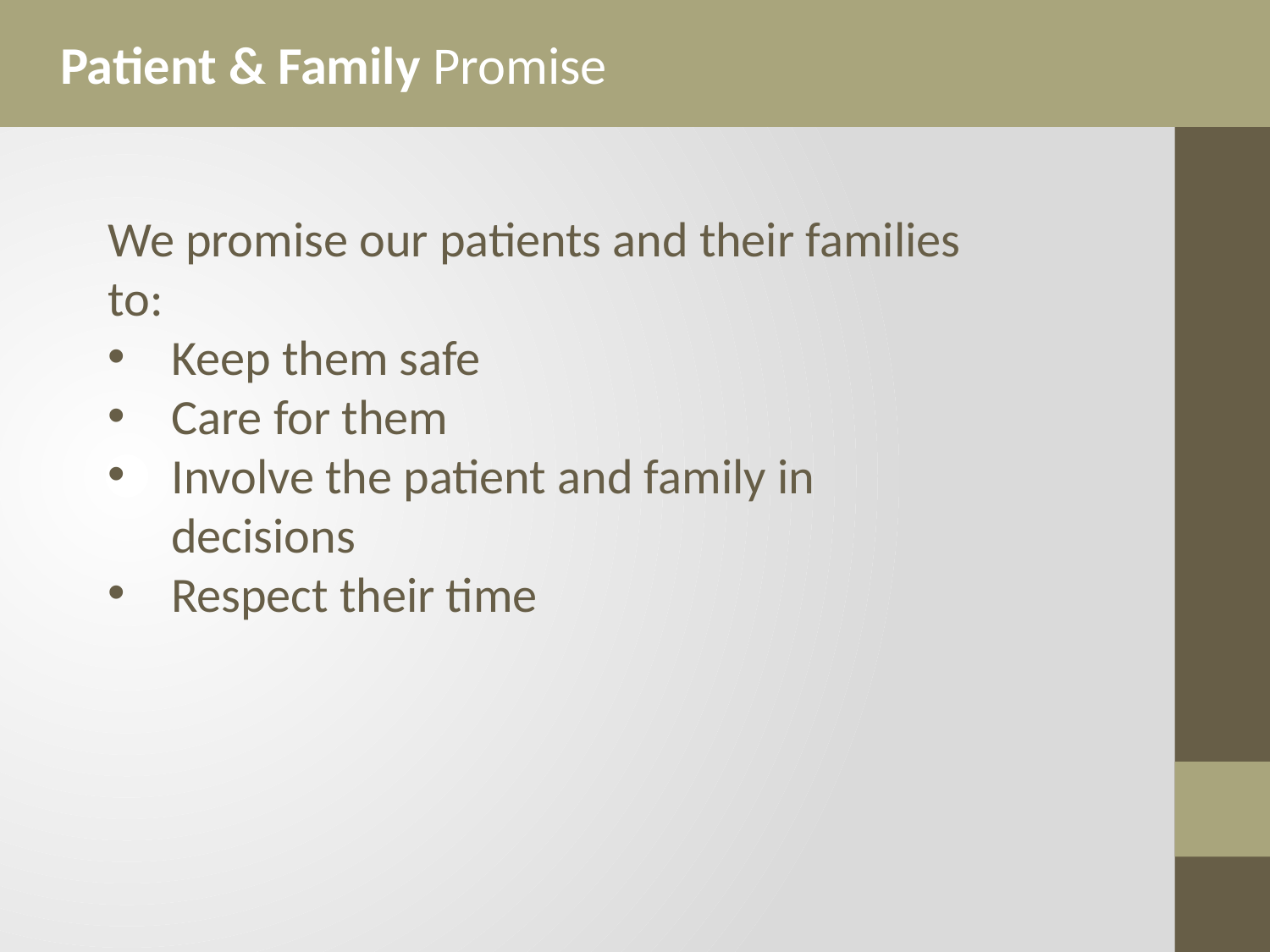

Patient & Family Promise
We promise our patients and their families to:
Keep them safe
Care for them
Involve the patient and family in decisions
Respect their time

## Slide 4
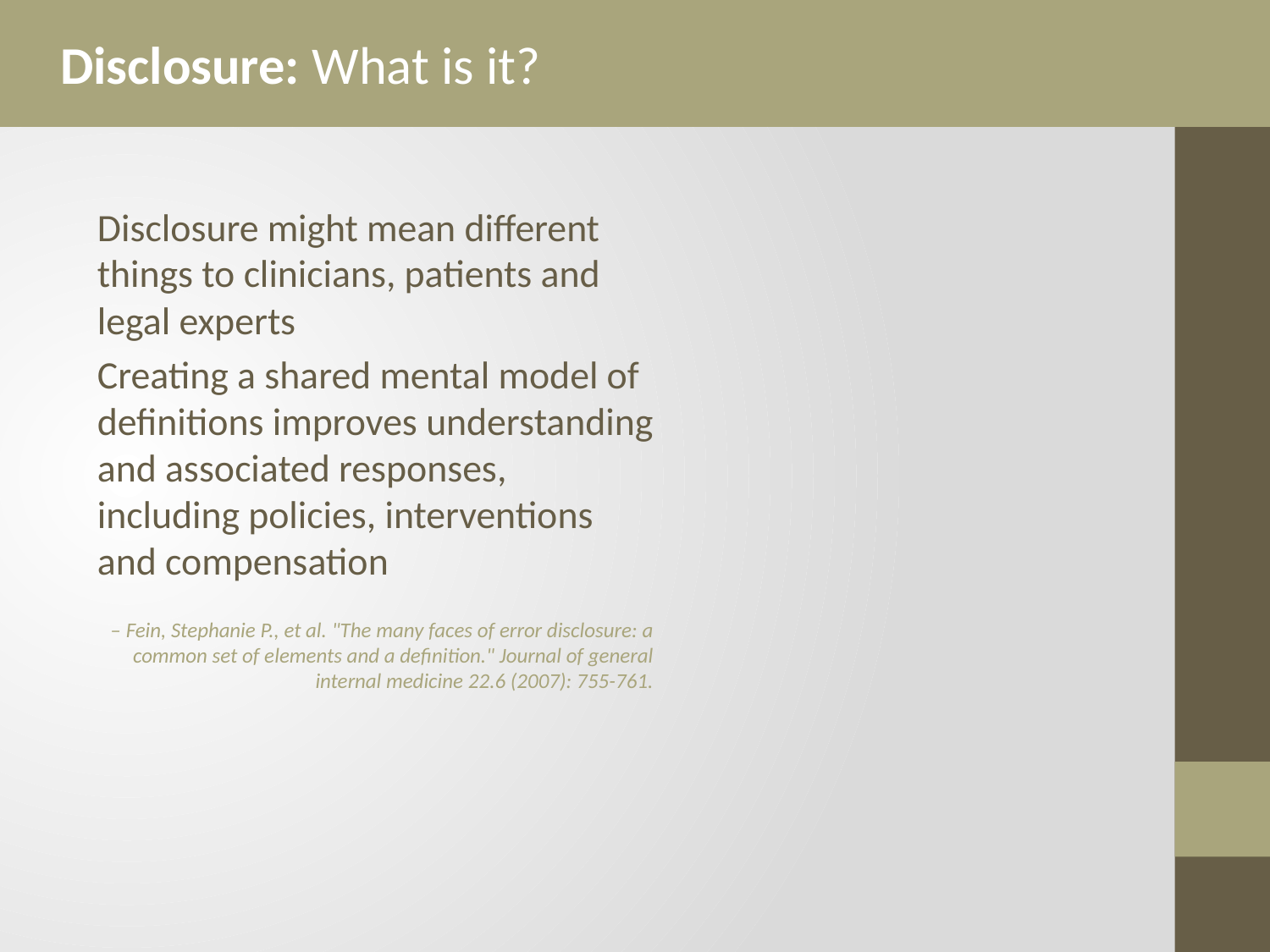

Disclosure: What is it?
Disclosure might mean different things to clinicians, patients and legal experts
Creating a shared mental model of definitions improves understanding and associated responses, including policies, interventions and compensation
– Fein, Stephanie P., et al. "The many faces of error disclosure: a common set of elements and a definition." Journal of general internal medicine 22.6 (2007): 755-761.

## Slide 5
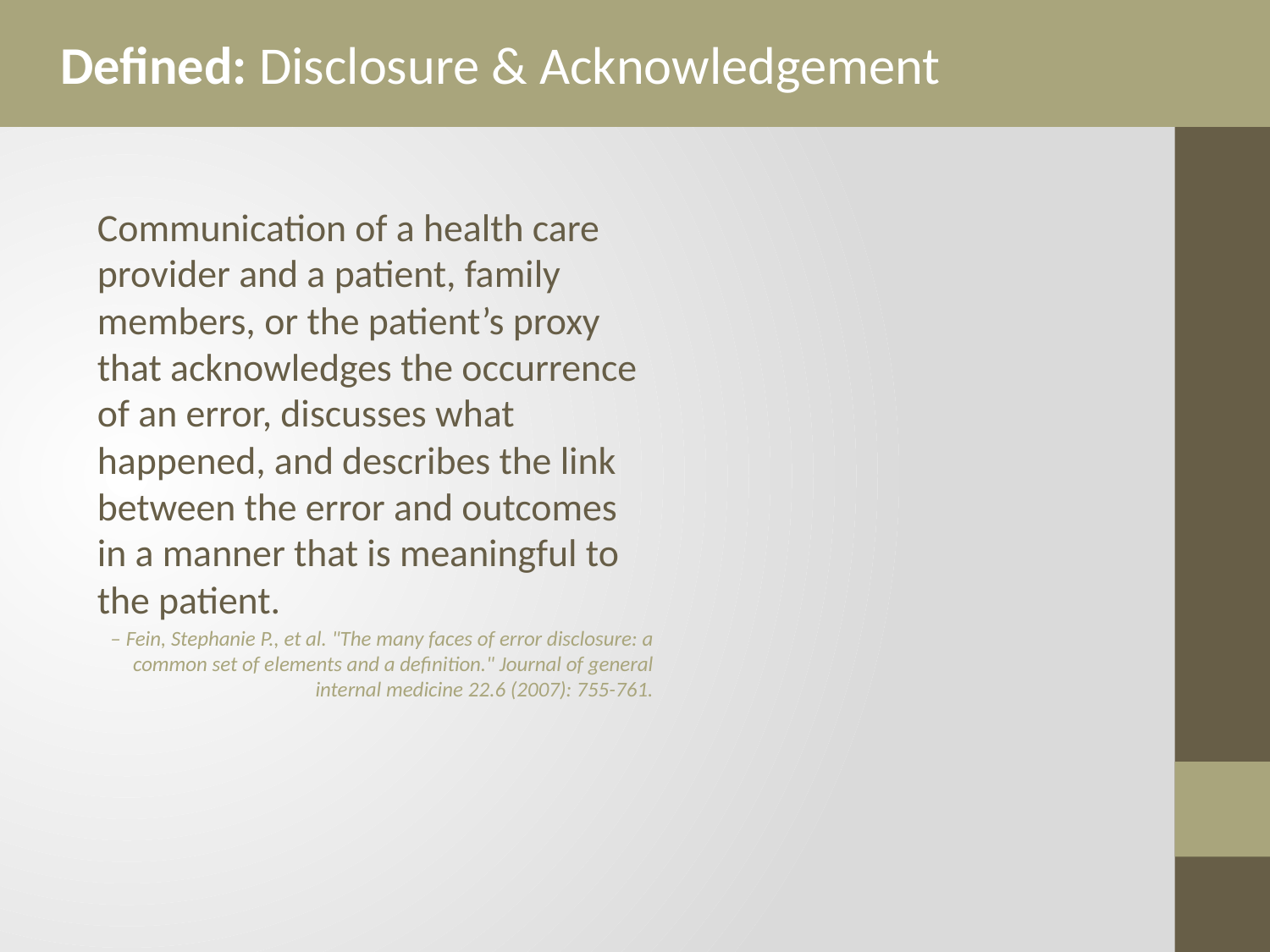

Defined: Disclosure & Acknowledgement
Communication of a health care provider and a patient, family members, or the patient’s proxy that acknowledges the occurrence of an error, discusses what happened, and describes the link between the error and outcomes in a manner that is meaningful to the patient.
– Fein, Stephanie P., et al. "The many faces of error disclosure: a common set of elements and a definition." Journal of general internal medicine 22.6 (2007): 755-761.

## Slide 6
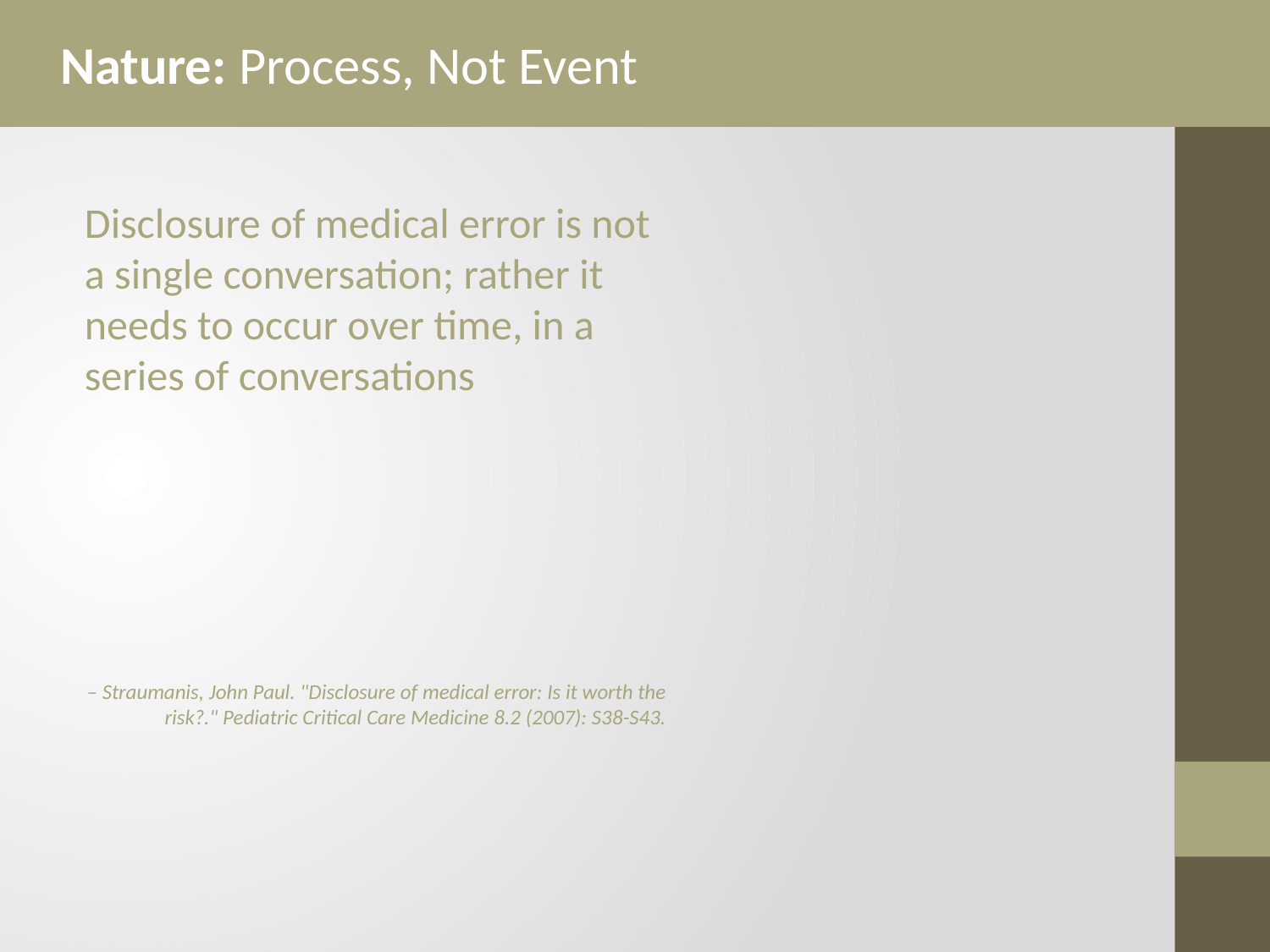

Nature: Process, Not Event
Disclosure of medical error is not a single conversation; rather it needs to occur over time, in a series of conversations
– Straumanis, John Paul. "Disclosure of medical error: Is it worth the risk?." Pediatric Critical Care Medicine 8.2 (2007): S38-S43.

## Slide 7
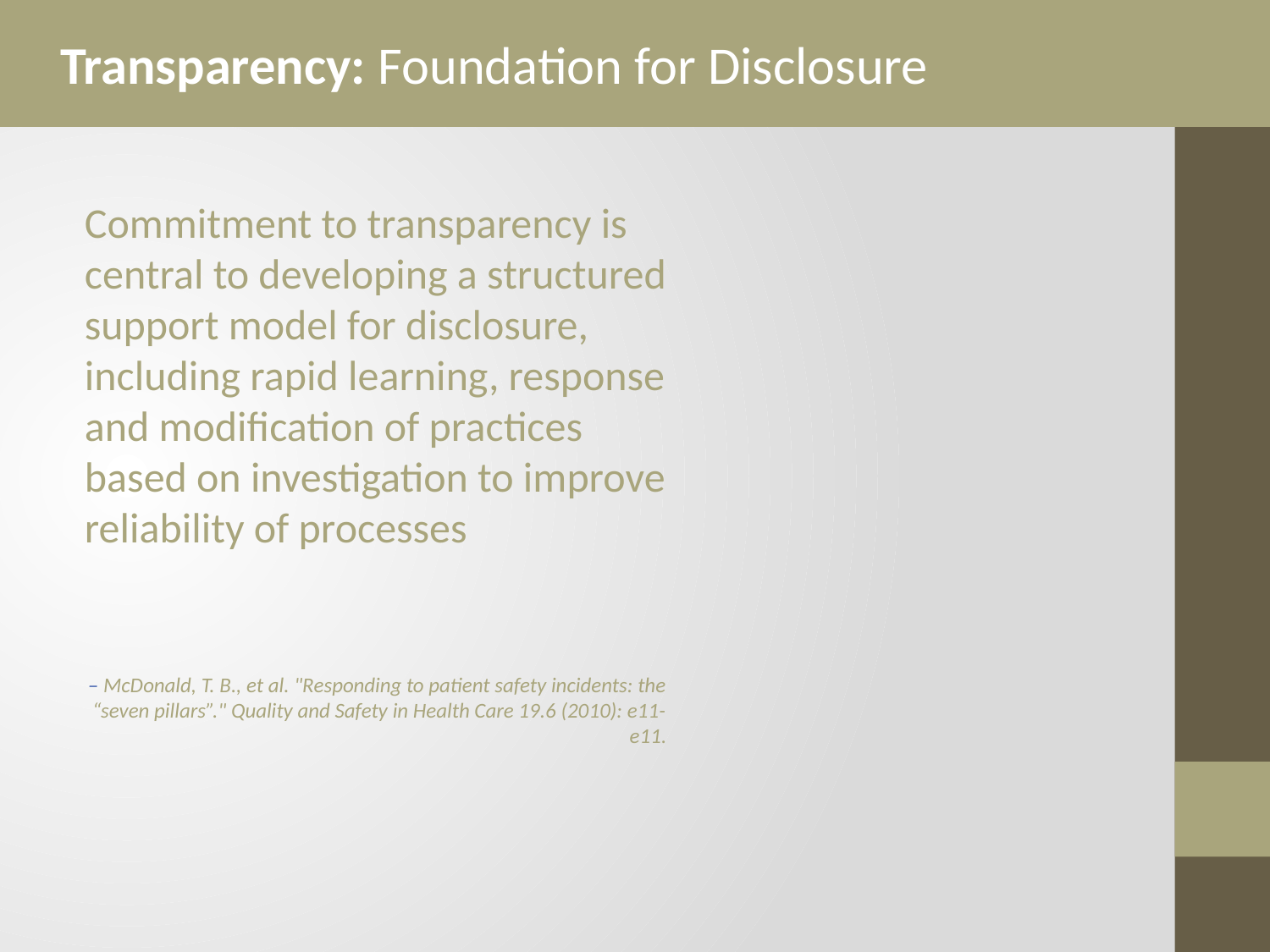

Transparency: Foundation for Disclosure
Commitment to transparency is central to developing a structured support model for disclosure, including rapid learning, response and modification of practices based on investigation to improve reliability of processes
– McDonald, T. B., et al. "Responding to patient safety incidents: the “seven pillars”." Quality and Safety in Health Care 19.6 (2010): e11-e11.

## Slide 8
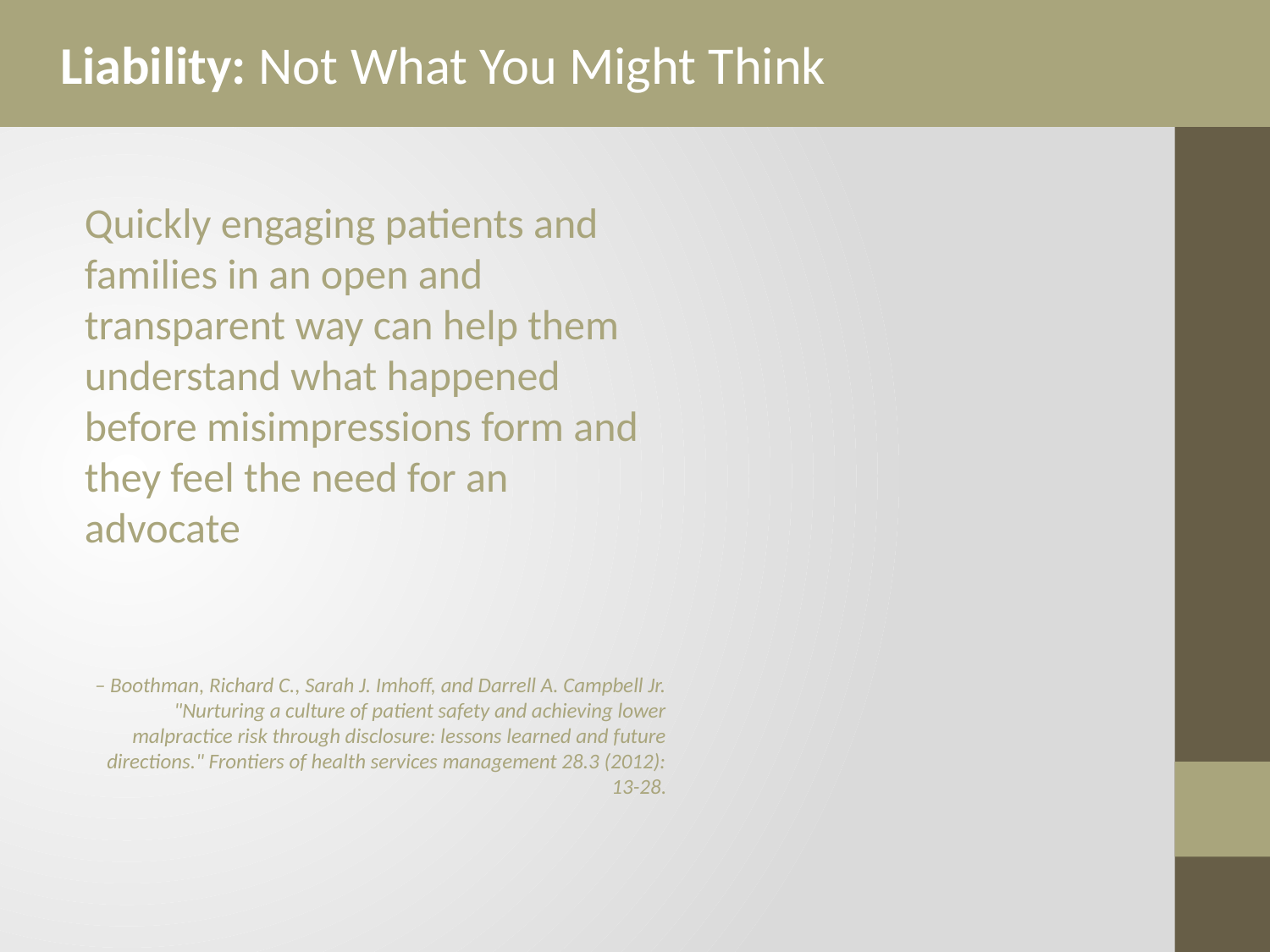

Liability: Not What You Might Think
Quickly engaging patients and families in an open and transparent way can help them understand what happened before misimpressions form and they feel the need for an advocate
– Boothman, Richard C., Sarah J. Imhoff, and Darrell A. Campbell Jr. "Nurturing a culture of patient safety and achieving lower malpractice risk through disclosure: lessons learned and future directions." Frontiers of health services management 28.3 (2012): 13-28.

## Slide 9
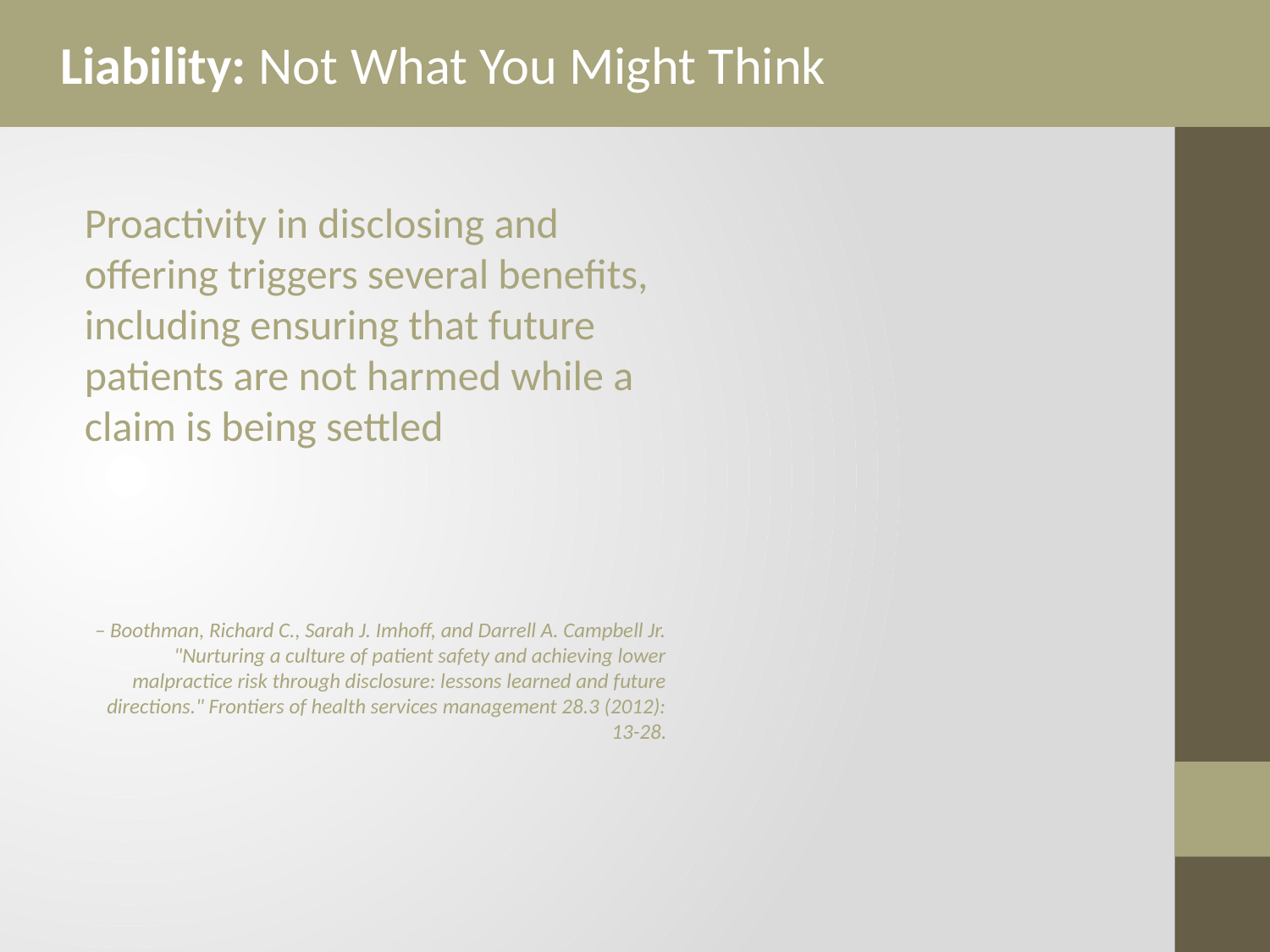

Liability: Not What You Might Think
Proactivity in disclosing and offering triggers several benefits, including ensuring that future patients are not harmed while a claim is being settled
– Boothman, Richard C., Sarah J. Imhoff, and Darrell A. Campbell Jr. "Nurturing a culture of patient safety and achieving lower malpractice risk through disclosure: lessons learned and future directions." Frontiers of health services management 28.3 (2012): 13-28.

## Slide 10
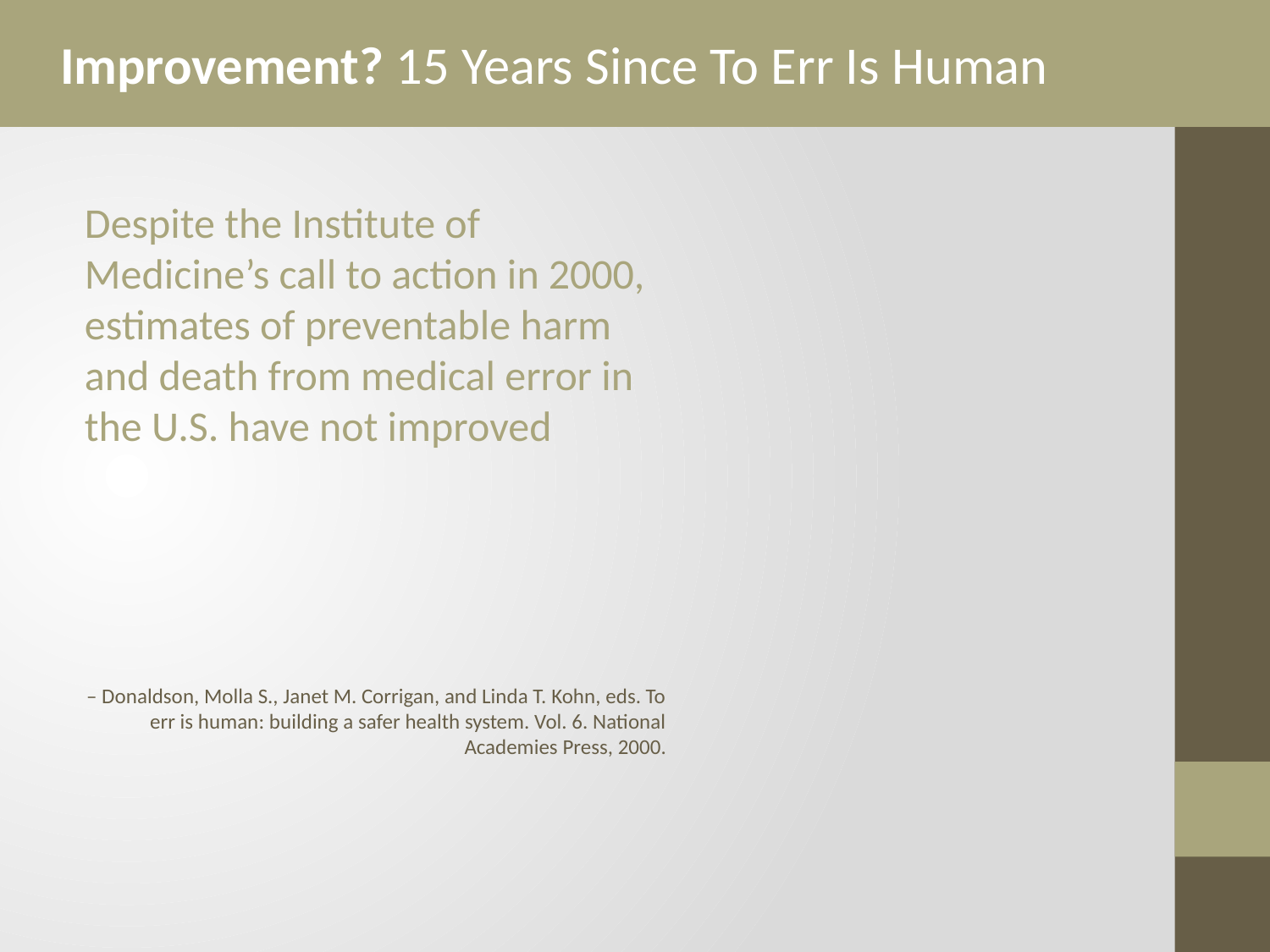

Improvement? 15 Years Since To Err Is Human
Despite the Institute of Medicine’s call to action in 2000, estimates of preventable harm and death from medical error in the U.S. have not improved
– Donaldson, Molla S., Janet M. Corrigan, and Linda T. Kohn, eds. To err is human: building a safer health system. Vol. 6. National Academies Press, 2000.

## Slide 11
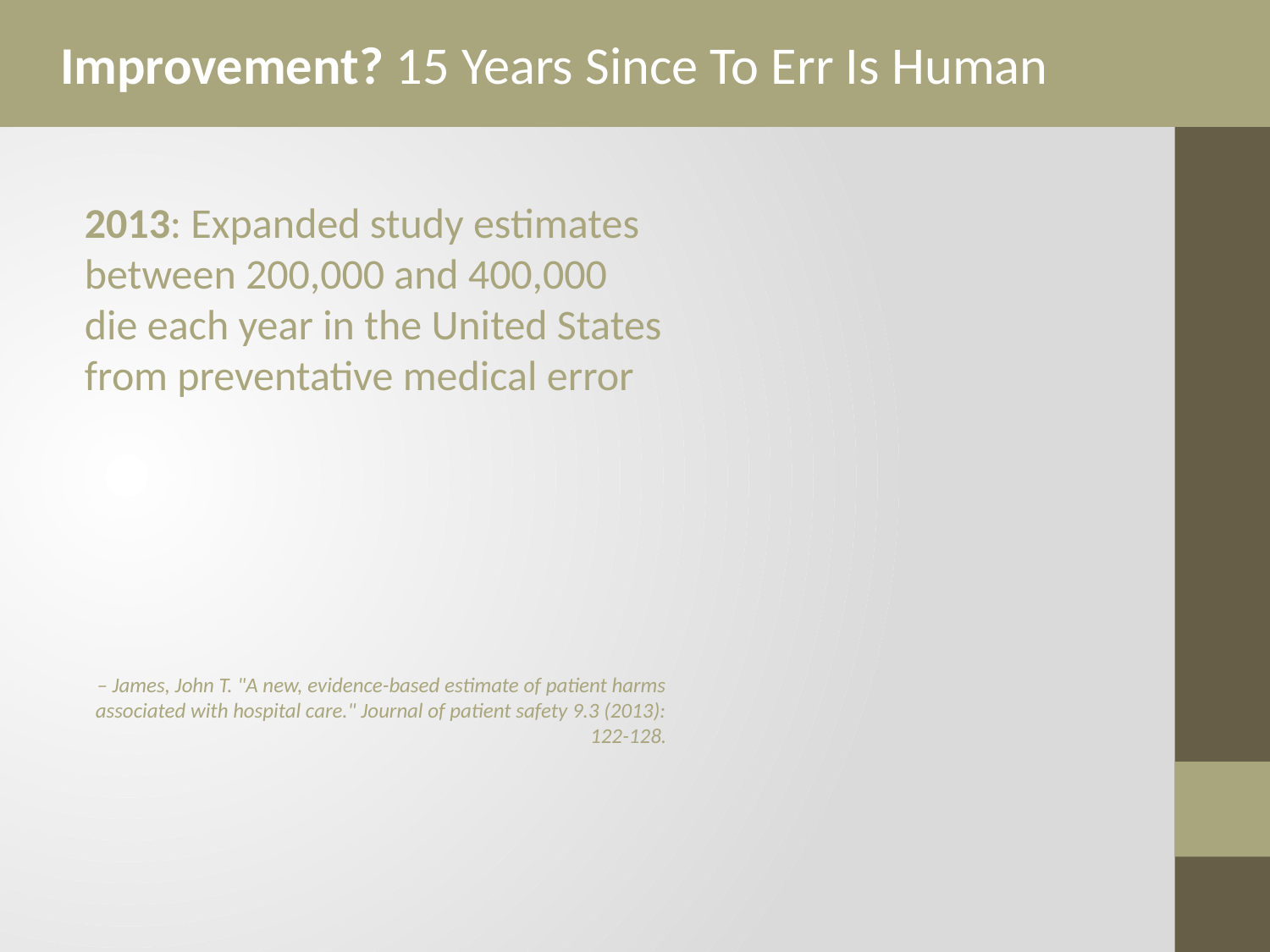

Improvement? 15 Years Since To Err Is Human
2013: Expanded study estimates between 200,000 and 400,000 die each year in the United States from preventative medical error
– James, John T. "A new, evidence-based estimate of patient harms associated with hospital care." Journal of patient safety 9.3 (2013): 122-128.

## Slide 12
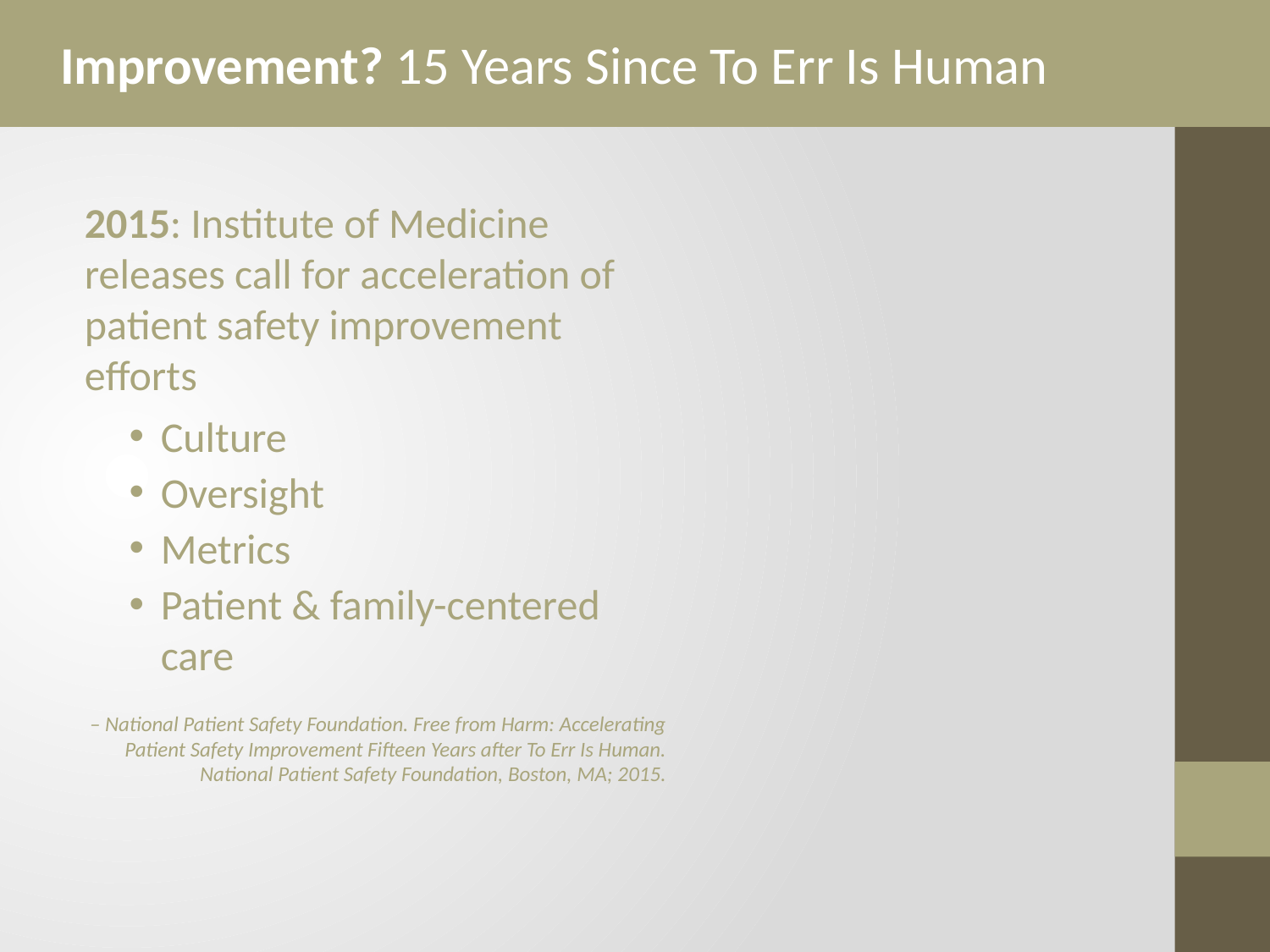

Improvement? 15 Years Since To Err Is Human
2015: Institute of Medicine releases call for acceleration of patient safety improvement efforts
Culture
Oversight
Metrics
Patient & family-centered care
– National Patient Safety Foundation. Free from Harm: Accelerating Patient Safety Improvement Fifteen Years after To Err Is Human. National Patient Safety Foundation, Boston, MA; 2015.

## Slide 13
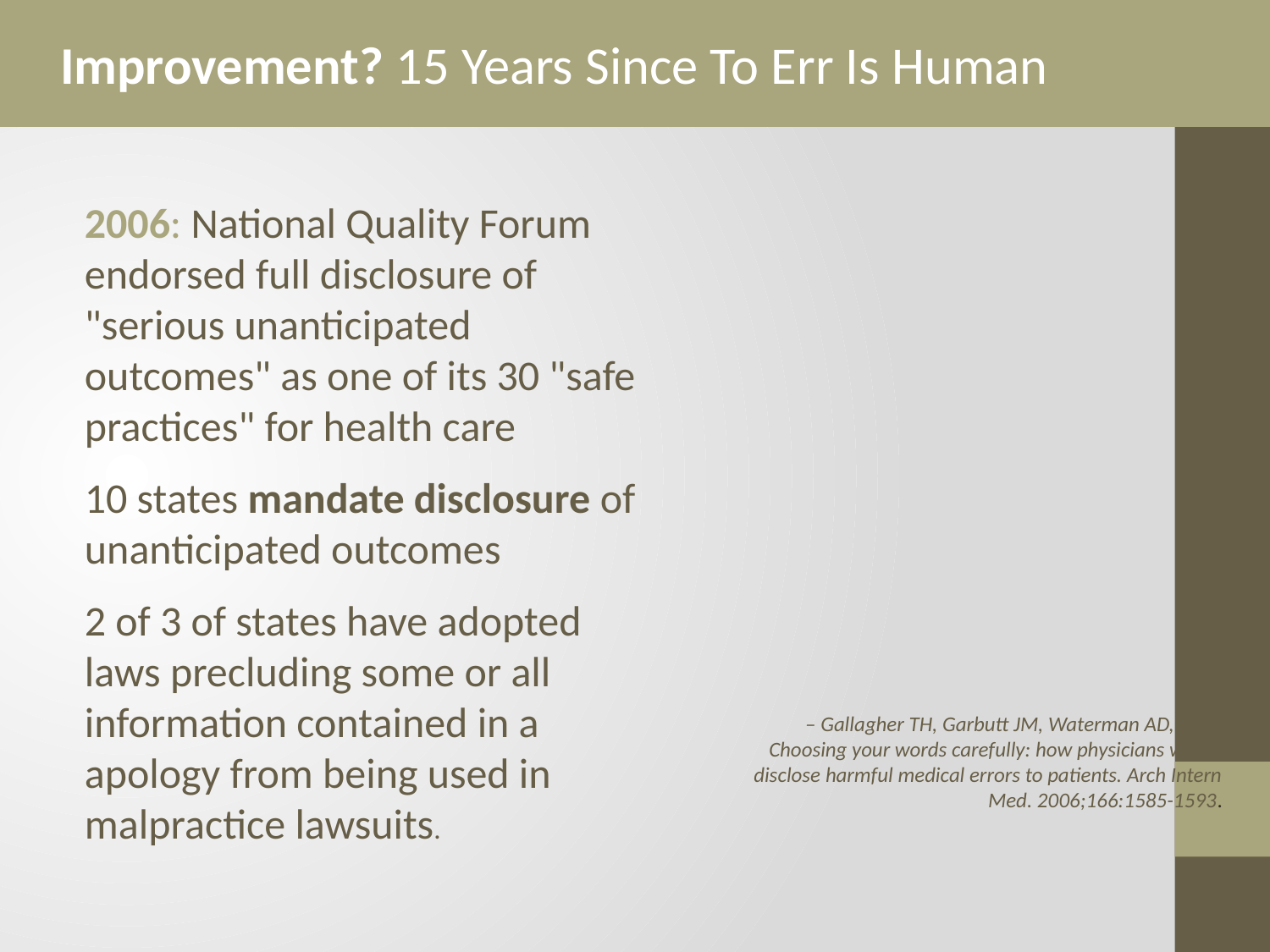

Improvement? 15 Years Since To Err Is Human
2006: National Quality Forum endorsed full disclosure of "serious unanticipated outcomes" as one of its 30 "safe practices" for health care
10 states mandate disclosure of unanticipated outcomes
2 of 3 of states have adopted laws precluding some or all information contained in a apology from being used in malpractice lawsuits.
– Gallagher TH, Garbutt JM, Waterman AD, et al. Choosing your words carefully: how physicians would disclose harmful medical errors to patients. Arch Intern Med. 2006;166:1585-1593.

## Slide 14
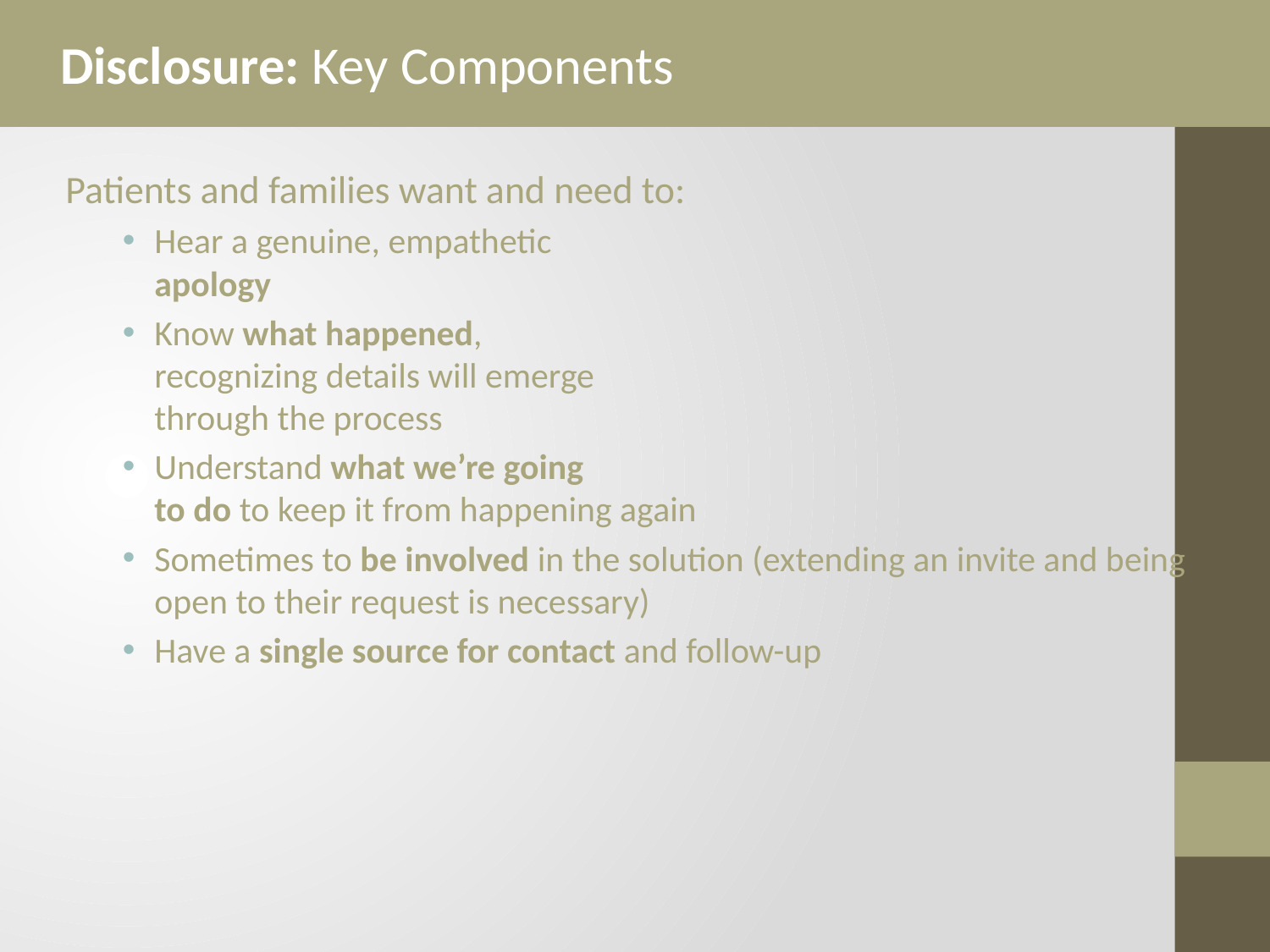

Disclosure: Key Components
Patients and families want and need to:
Hear a genuine, empathetic
apology
Know what happened,
recognizing details will emerge
through the process
Understand what we’re going
to do to keep it from happening again
Sometimes to be involved in the solution (extending an invite and being open to their request is necessary)
Have a single source for contact and follow-up

## Slide 15
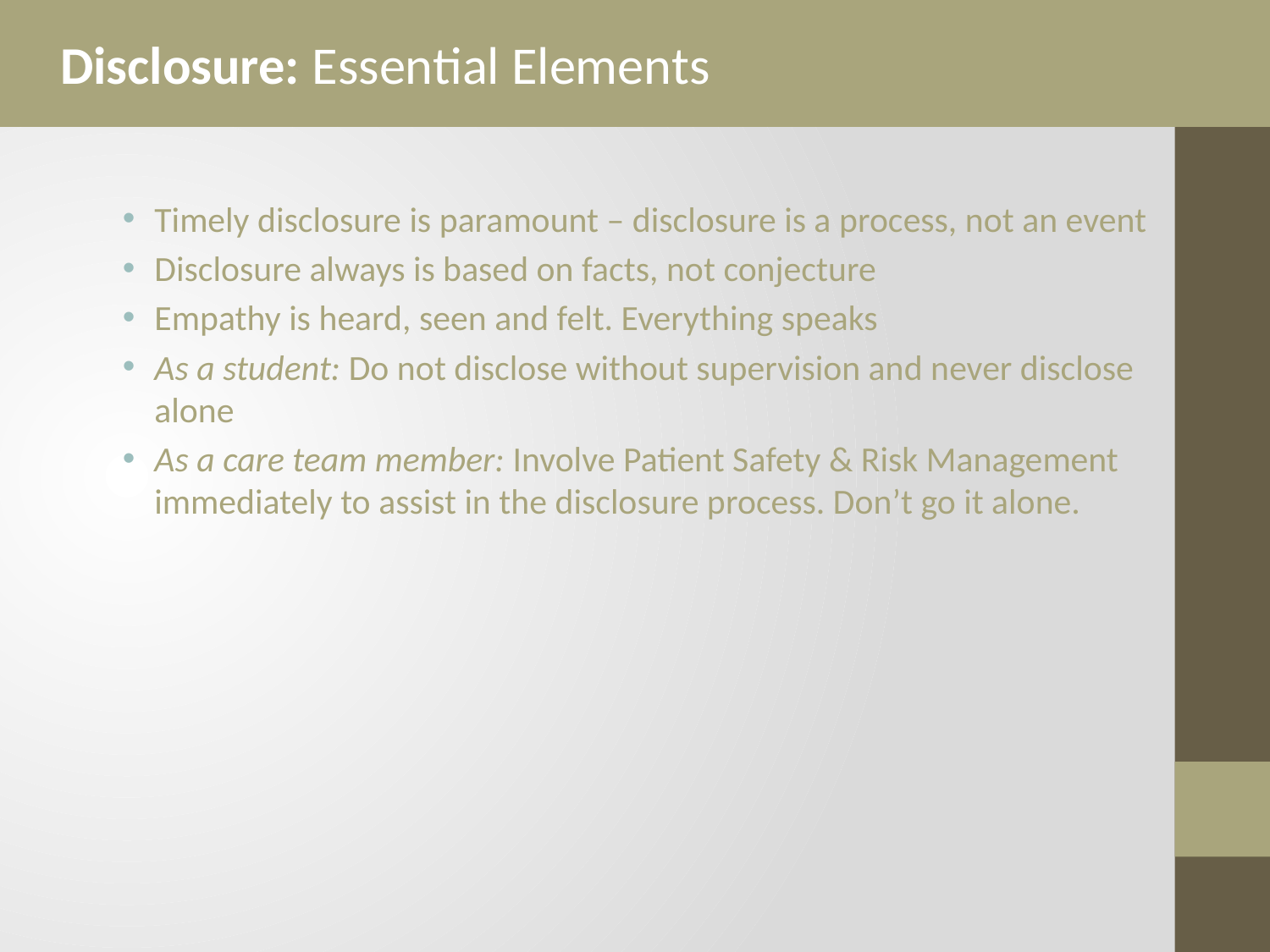

Disclosure: Essential Elements
Timely disclosure is paramount – disclosure is a process, not an event
Disclosure always is based on facts, not conjecture
Empathy is heard, seen and felt. Everything speaks
As a student: Do not disclose without supervision and never disclose alone
As a care team member: Involve Patient Safety & Risk Management immediately to assist in the disclosure process. Don’t go it alone.
