# Supplementary material for: Safety in Numbers: Successful Student-Approved Case-Based Interprofessional Safety Workshop Utilizing Simulated Real-Life Safety Cases
Source: MedEdPORTAL. 2020 Jan 31;16:10874. doi: 10.15766/mep_2374-8265.10874 (PMC7065299; doi:10.15766/mep_2374-8265.10874)
Supplement: Supplementary file 1 — A. Pre- & Postevent Surveys.docx B. IPE Safety Workshop Agenda.docx C. RCA AM Session Facilitator Guide.docx D. RCA AM Session Facilitator Annotated Case Time Line.docx E. RCA AM Session Student Case Time Line.docx F. RCA AM Session Interviewee Scripts.docx G. RCA AM Session Patient Background & EWS Info.docx H. RCA AM Session Media - Radiology.docx I. RCA AM Session Media - Oxygen Tanks.docx J. Corrective Action PM Session Facilitator Guide.docx K. Corrective Action PM Session Effectiveness Chart.docx L. Corrective Action PM Session Worksheet.docx M. Executive Case Summary.docx N. Large-Group Lecture Schedule & Topic List.docx O. PPT 1 - Contributing to a Culture of Safety.pptx P. PPT 2 - Systems Improvement.pptx Q. PPT 3 - Impact of Students and Residents on QI.pptx R. PPT 4 - Presentation of Safety Case.pptx S. PPT 5 - Disclosing Medical Errors.pptx T. PPT 6 - Training for Resilience.pptx U. PPT 7 - Introduction to Improvement Plans.pptx V. Facilitator Postworkshop Survey.docx [file mep-16-10874-s001.zip › T. PPT 6 - Training for Resilience.pptx]

## Slide 1
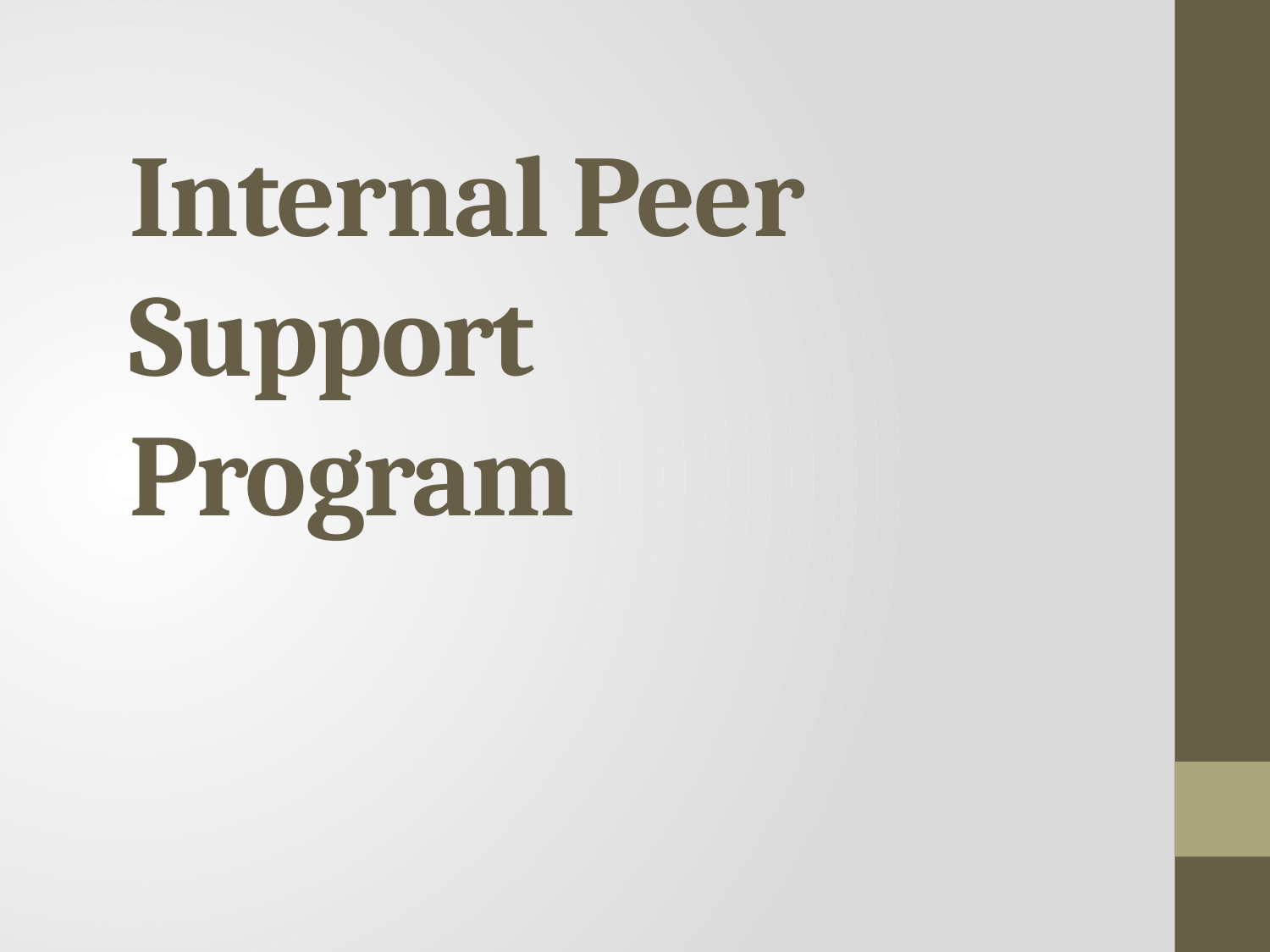

# Internal Peer Support Program

## Slide 2
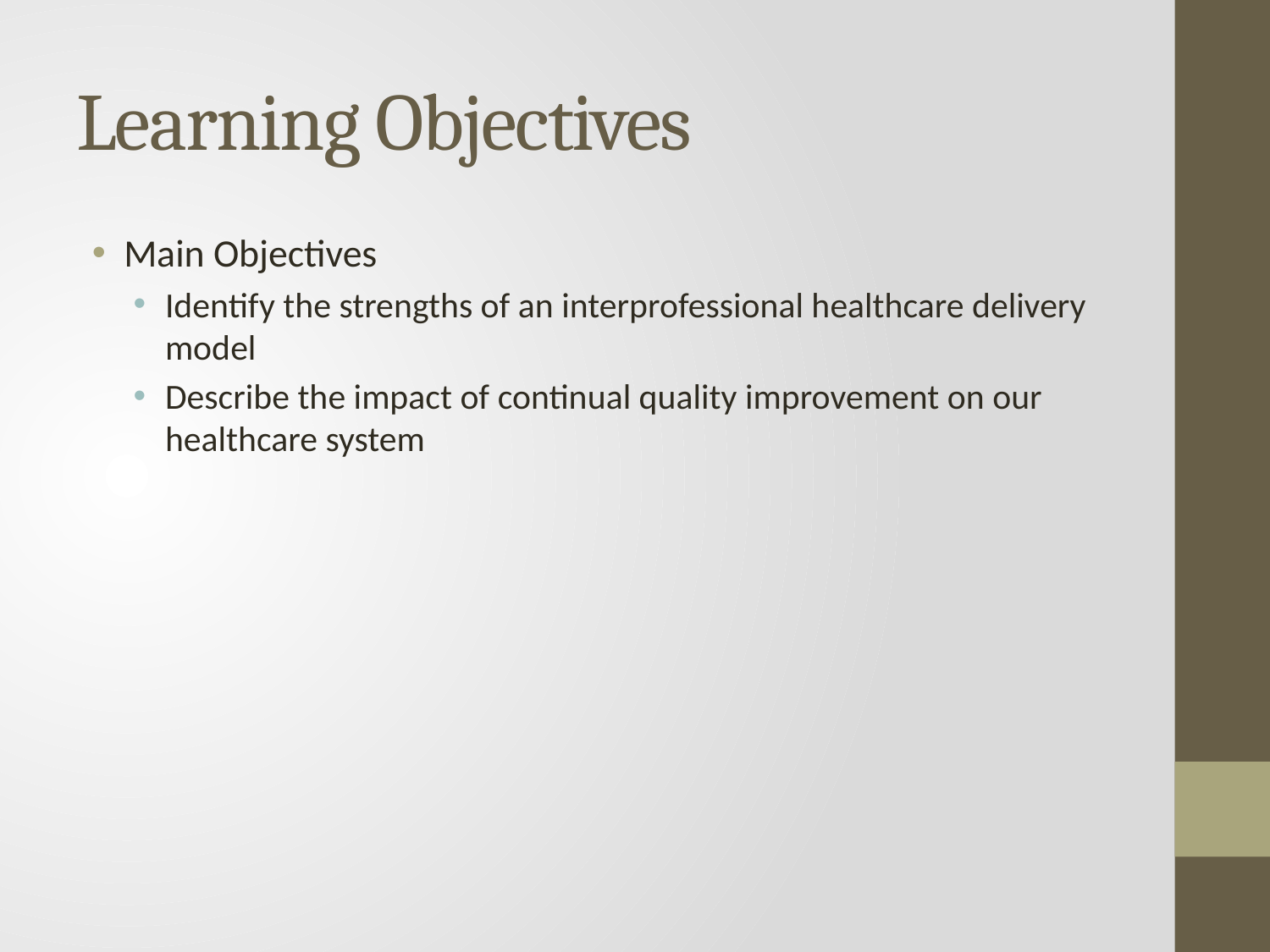

# Learning Objectives
Main Objectives
Identify the strengths of an interprofessional healthcare delivery model
Describe the impact of continual quality improvement on our healthcare system

## Slide 3
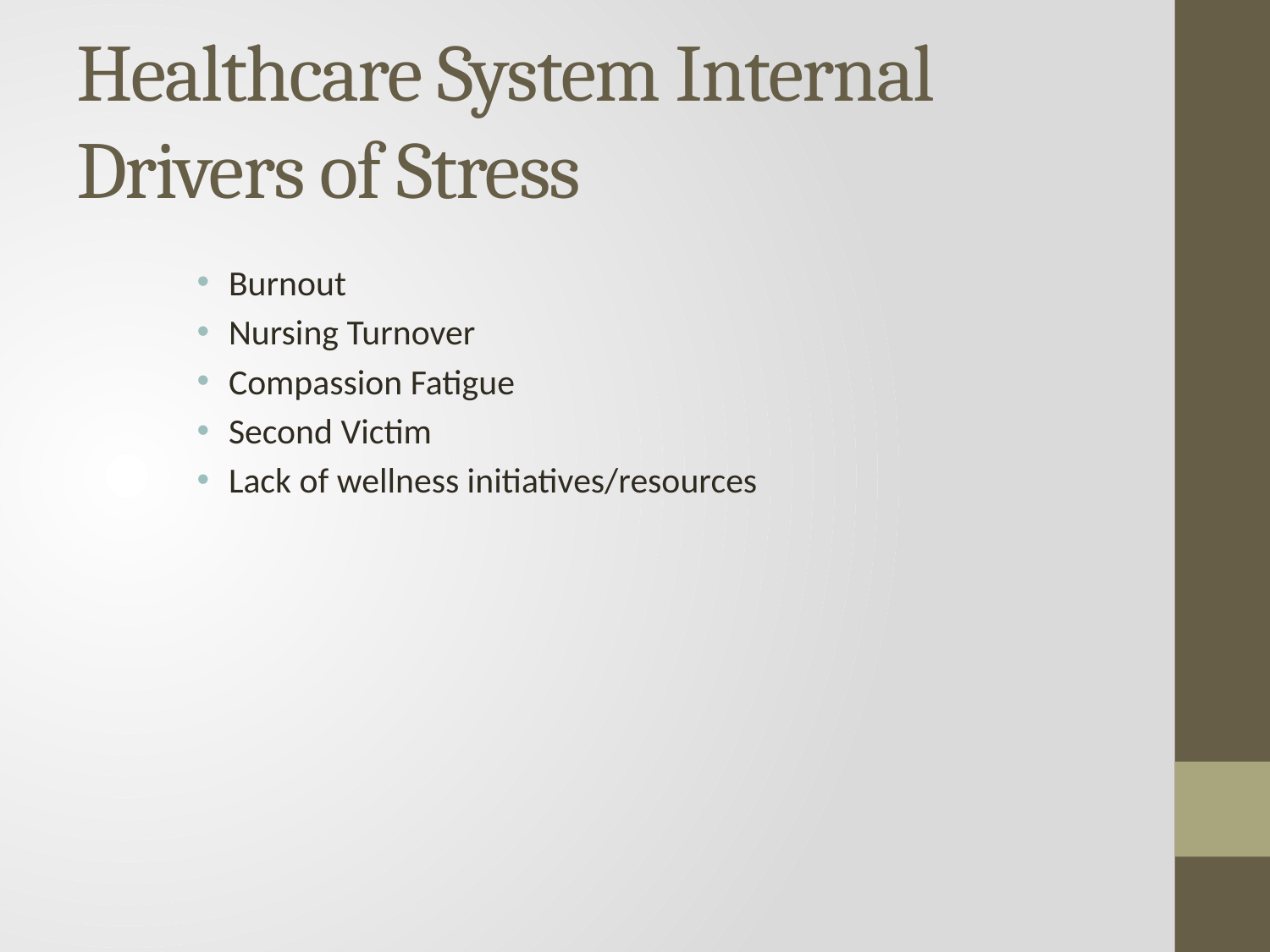

# Healthcare System Internal Drivers of Stress
Burnout
Nursing Turnover
Compassion Fatigue
Second Victim
Lack of wellness initiatives/resources

## Slide 4
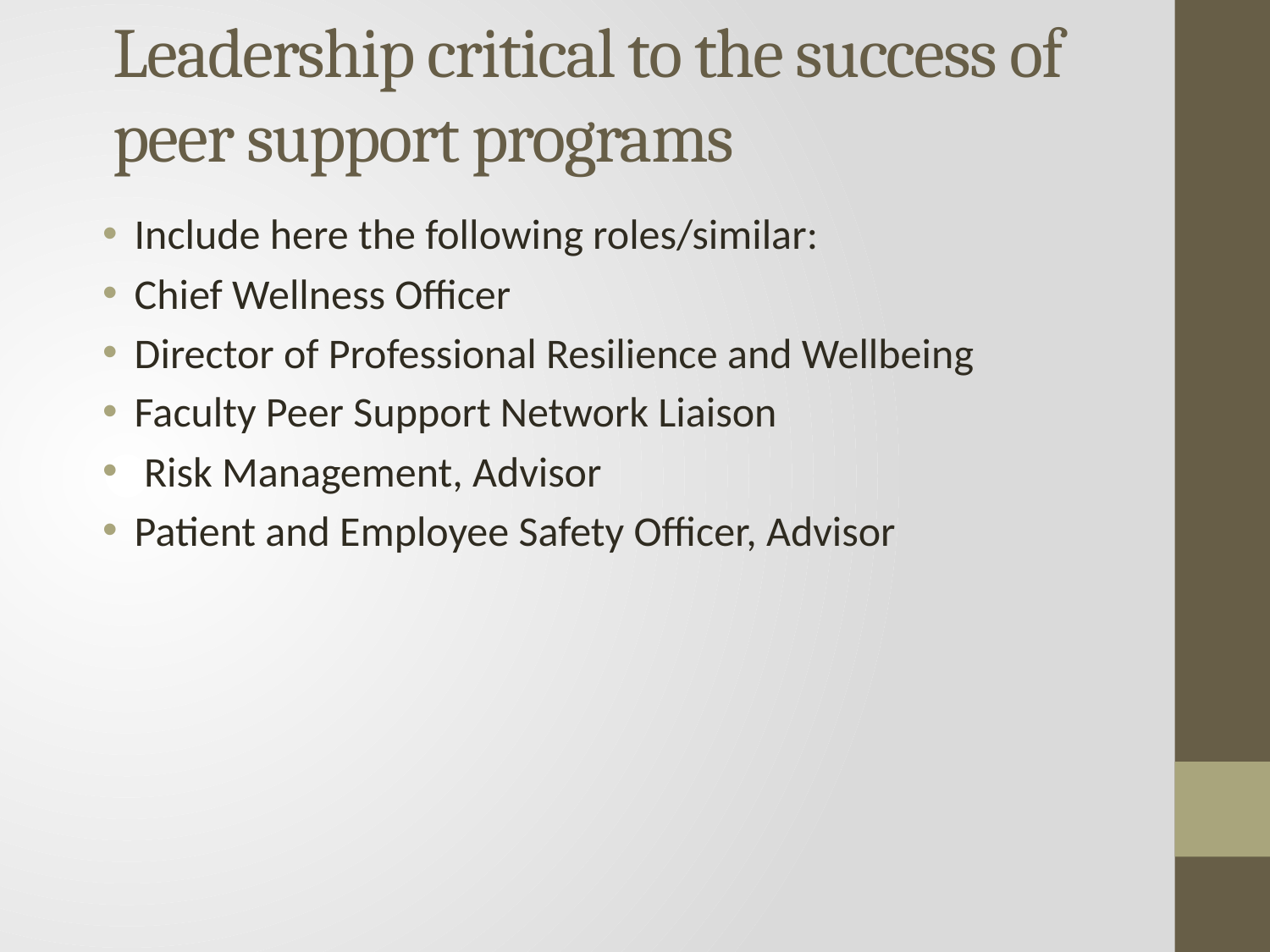

# Leadership critical to the success of peer support programs
Include here the following roles/similar:
Chief Wellness Officer
Director of Professional Resilience and Wellbeing
Faculty Peer Support Network Liaison
 Risk Management, Advisor
Patient and Employee Safety Officer, Advisor

## Slide 5
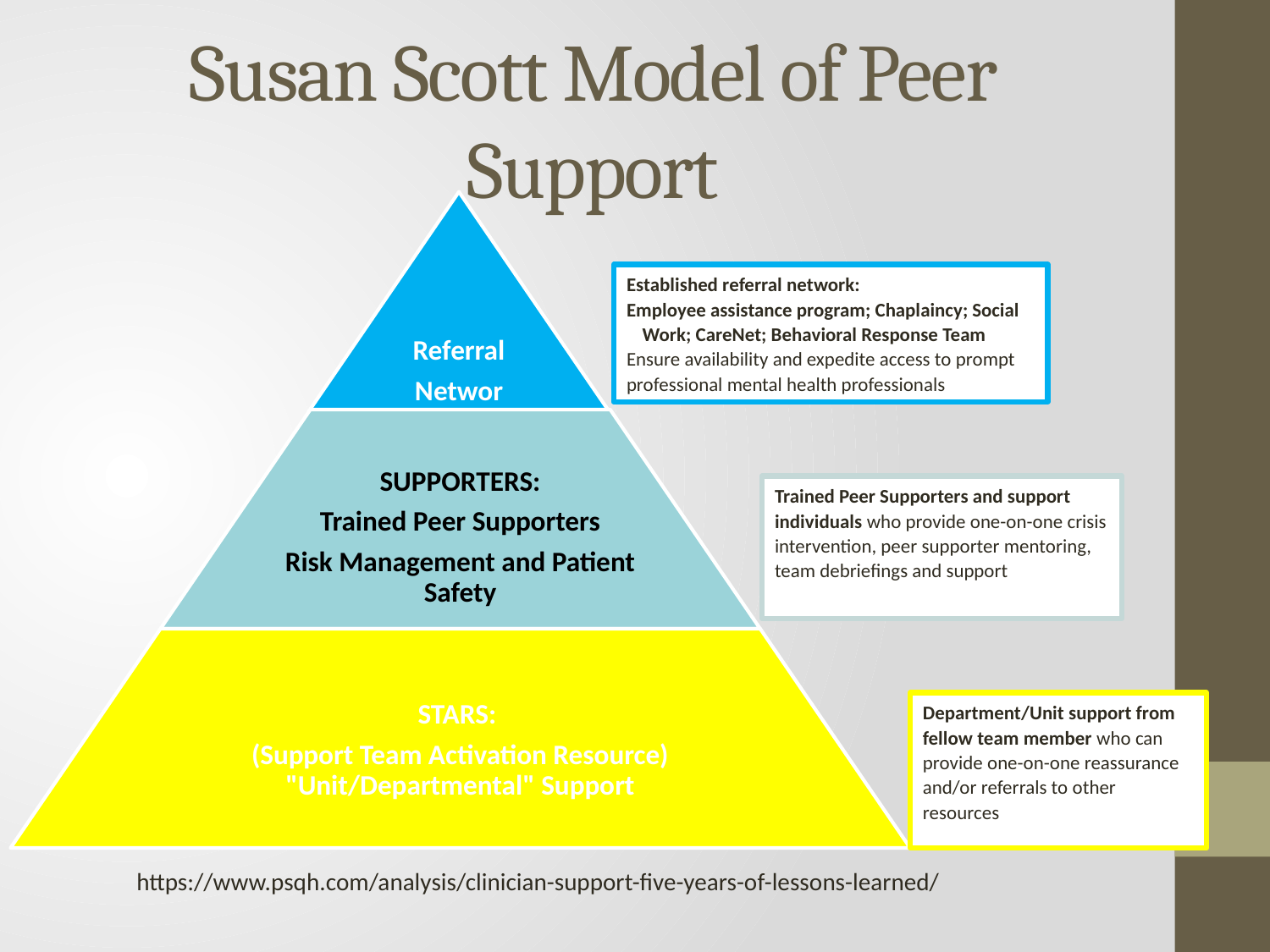

# Susan Scott Model of Peer Support
Established referral network:
Employee assistance program; Chaplaincy; Social Work; CareNet; Behavioral Response Team
Ensure availability and expedite access to prompt professional mental health professionals
Trained Peer Supporters and support individuals who provide one-on-one crisis intervention, peer supporter mentoring, team debriefings and support
Department/Unit support from fellow team member who can provide one-on-one reassurance and/or referrals to other resources
https://www.psqh.com/analysis/clinician-support-five-years-of-lessons-learned/

## Slide 6
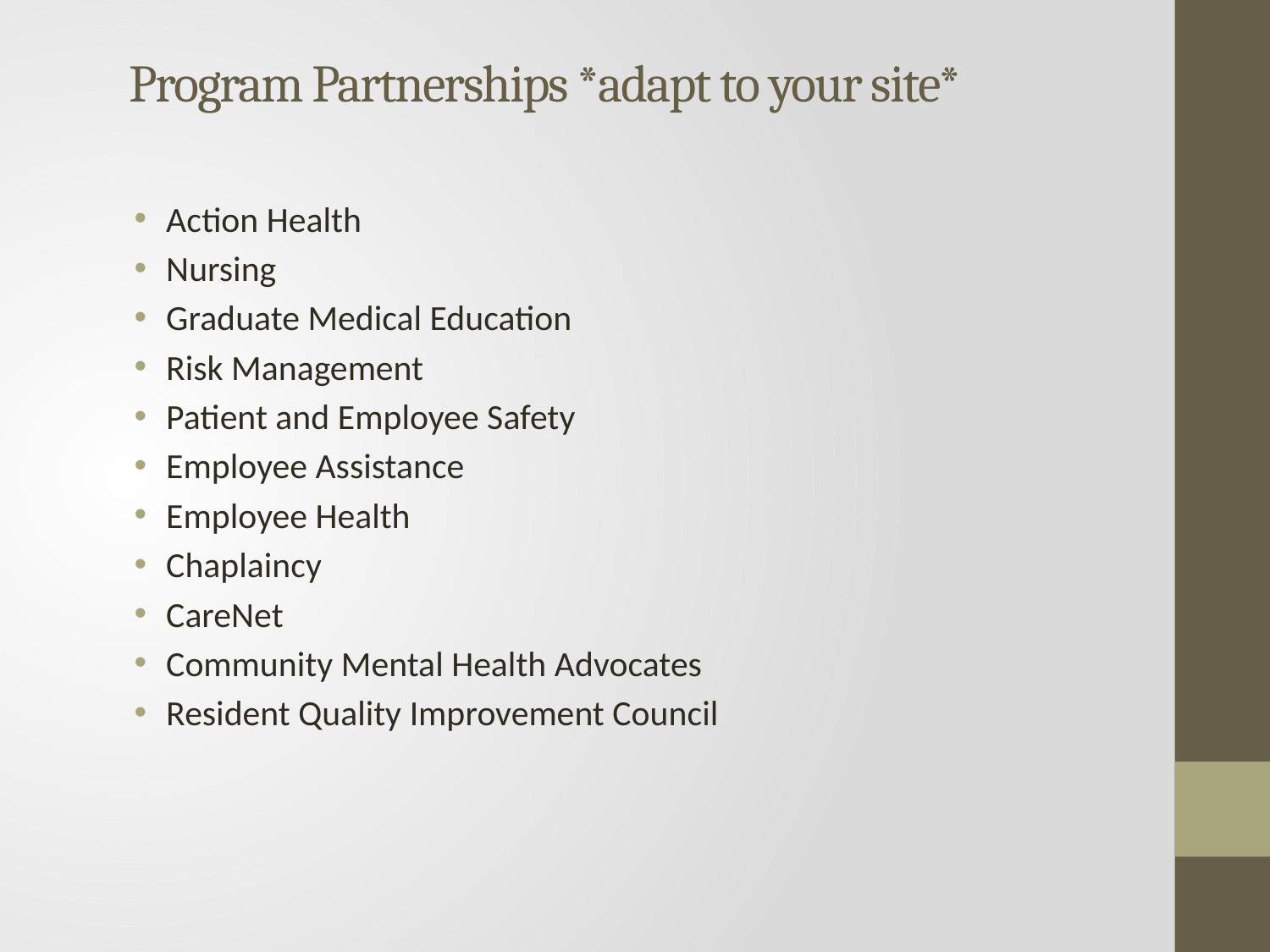

# Program Partnerships *adapt to your site*
Action Health
Nursing
Graduate Medical Education
Risk Management
Patient and Employee Safety
Employee Assistance
Employee Health
Chaplaincy
CareNet
Community Mental Health Advocates
Resident Quality Improvement Council

## Slide 7
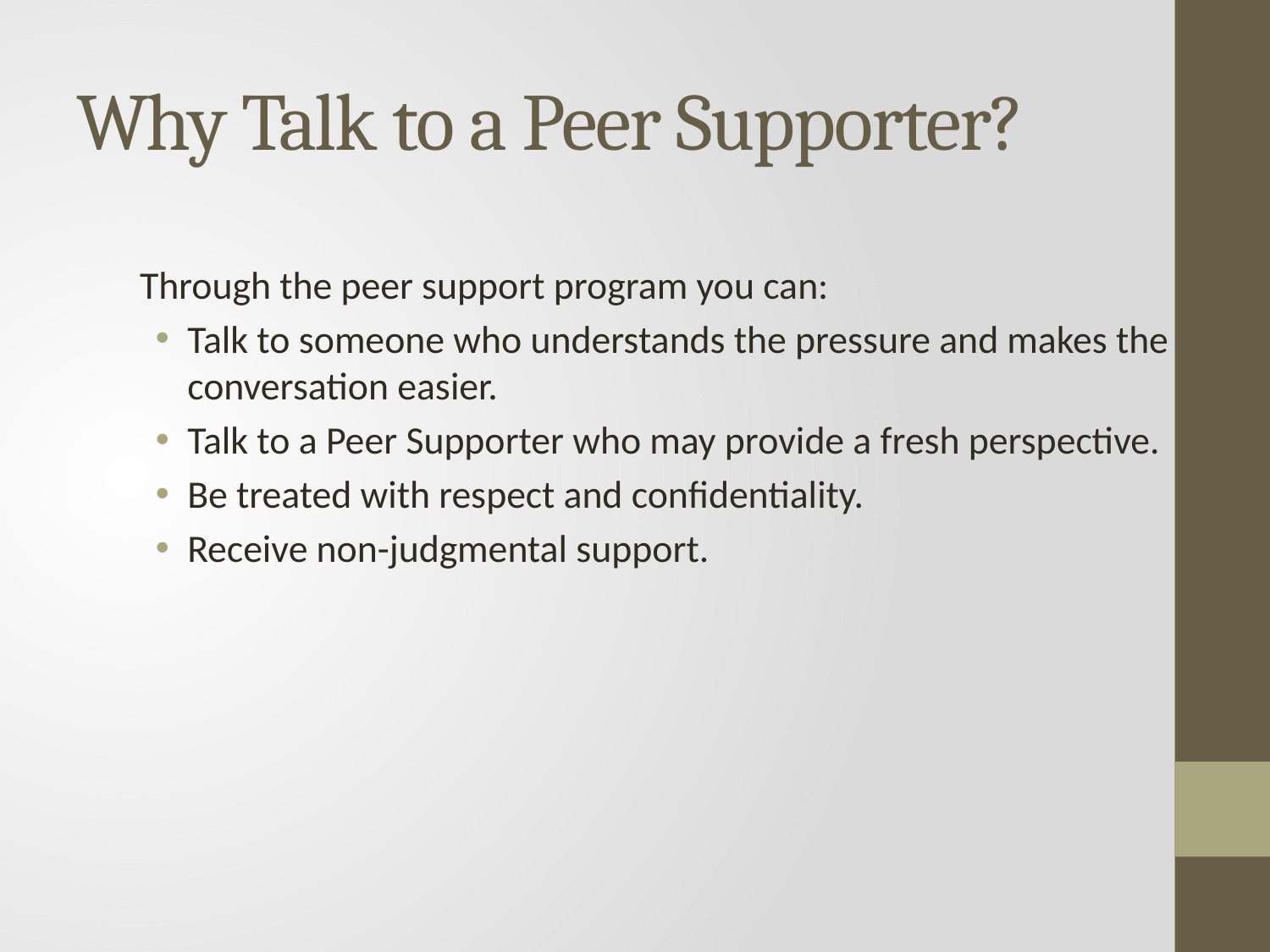

# Why Talk to a Peer Supporter?
Through the peer support program you can:
Talk to someone who understands the pressure and makes the conversation easier.
Talk to a Peer Supporter who may provide a fresh perspective.
Be treated with respect and confidentiality.
Receive non-judgmental support.

## Slide 8
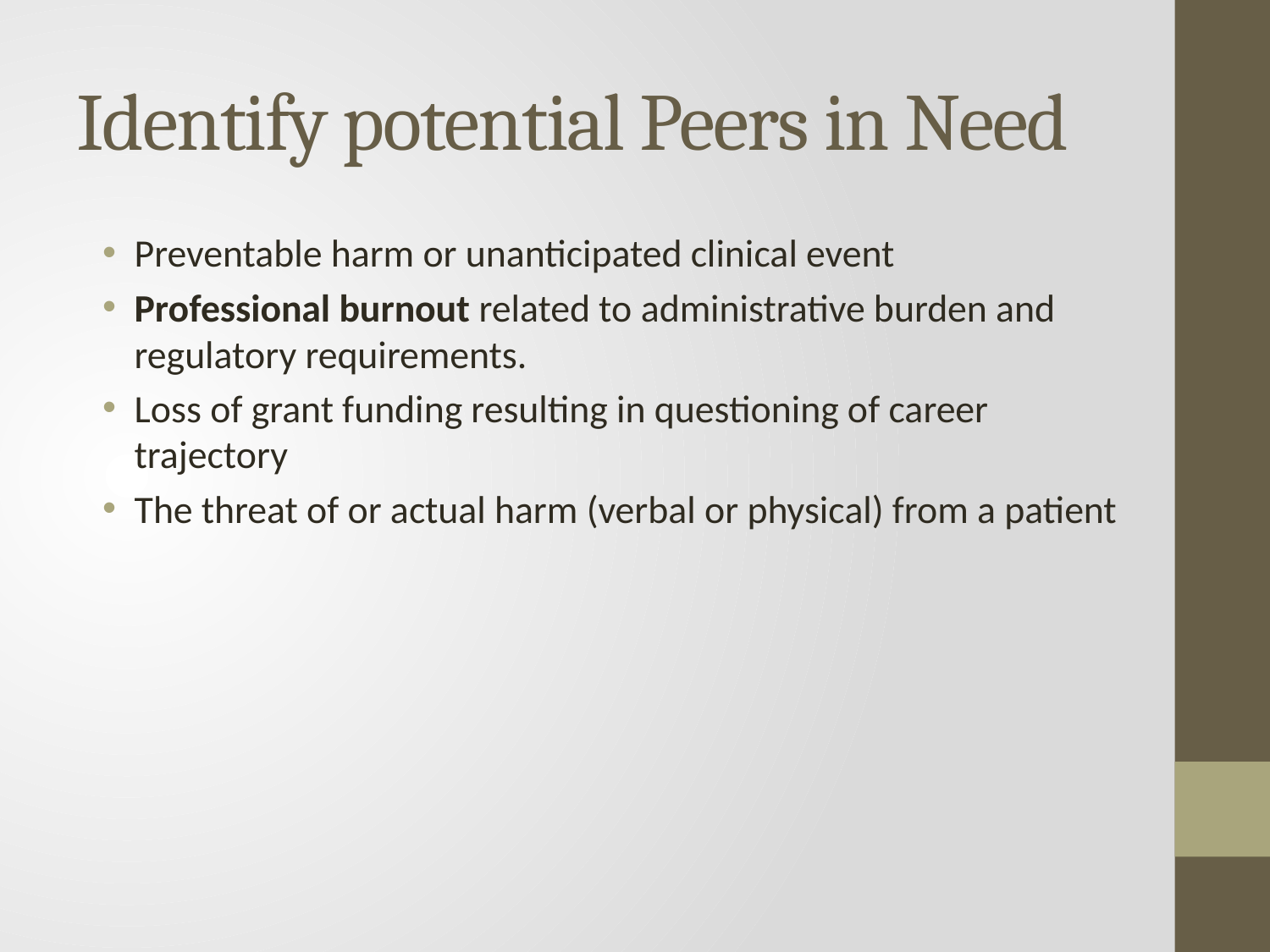

# Identify potential Peers in Need
Preventable harm or unanticipated clinical event
Professional burnout related to administrative burden and regulatory requirements.
Loss of grant funding resulting in questioning of career trajectory
The threat of or actual harm (verbal or physical) from a patient

## Slide 9
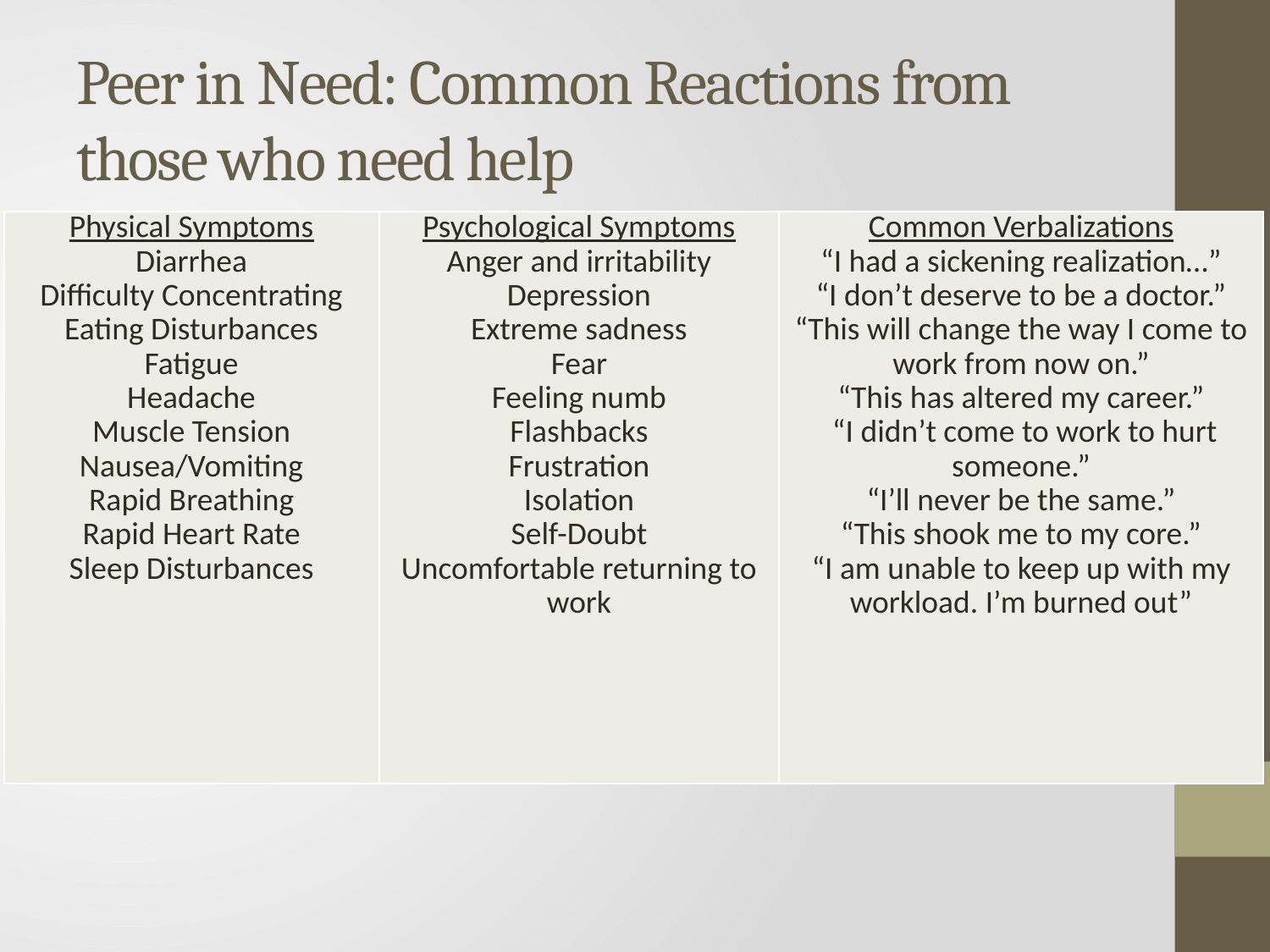

# Peer in Need: Common Reactions from those who need help
| Physical Symptoms Diarrhea Difficulty Concentrating Eating Disturbances Fatigue Headache Muscle Tension Nausea/Vomiting Rapid Breathing Rapid Heart Rate Sleep Disturbances | Psychological Symptoms Anger and irritability Depression Extreme sadness Fear Feeling numb Flashbacks Frustration Isolation Self-Doubt Uncomfortable returning to work | Common Verbalizations “I had a sickening realization…” “I don’t deserve to be a doctor.” “This will change the way I come to work from now on.” “This has altered my career.” “I didn’t come to work to hurt someone.” “I’ll never be the same.” “This shook me to my core.” “I am unable to keep up with my workload. I’m burned out” |
| --- | --- | --- |

## Slide 10
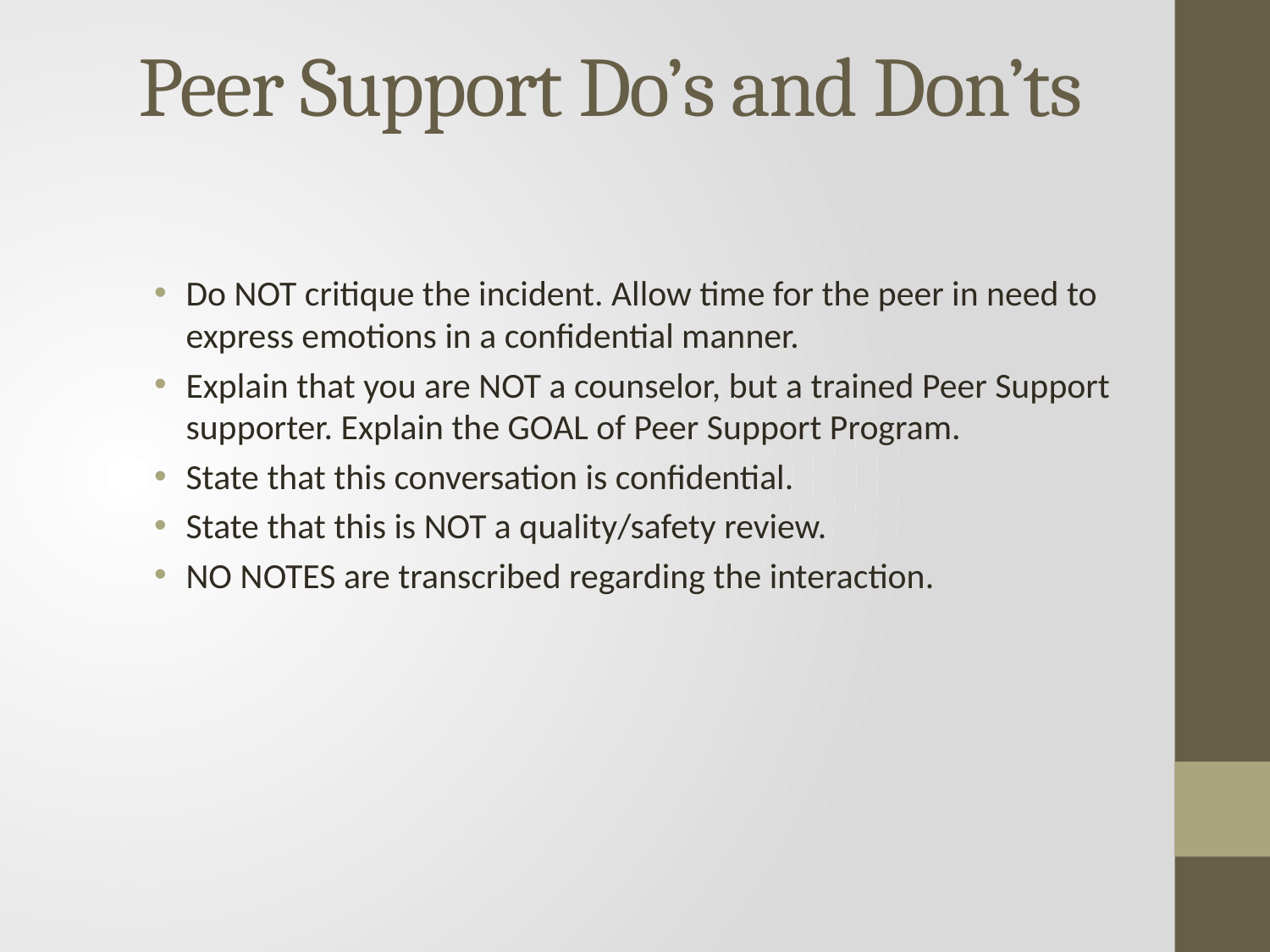

# Peer Support Do’s and Don’ts
Do NOT critique the incident. Allow time for the peer in need to express emotions in a confidential manner.
Explain that you are NOT a counselor, but a trained Peer Support supporter. Explain the GOAL of Peer Support Program.
State that this conversation is confidential.
State that this is NOT a quality/safety review.
NO NOTES are transcribed regarding the interaction.

## Slide 11
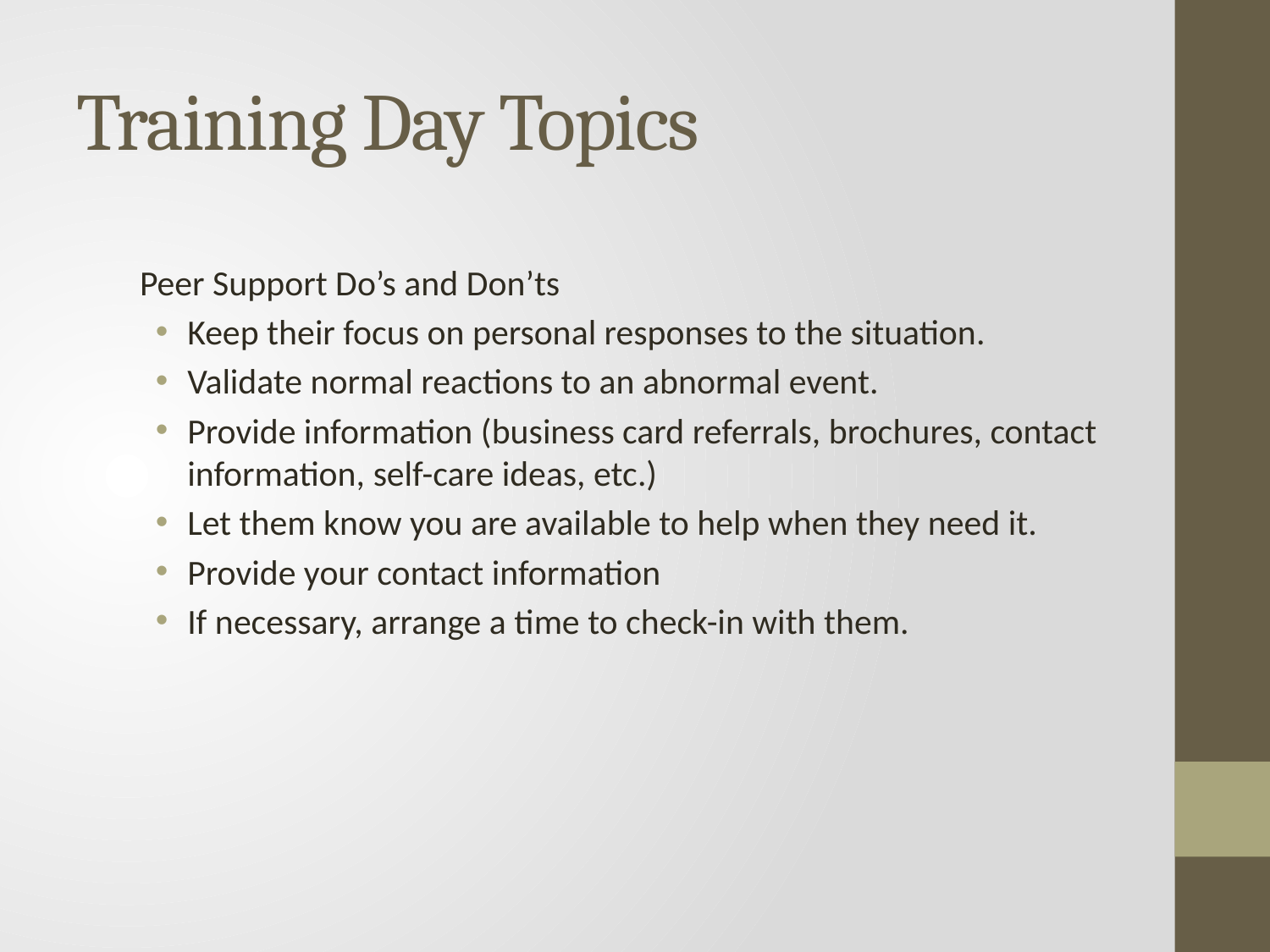

# Training Day Topics
Peer Support Do’s and Don’ts
Keep their focus on personal responses to the situation.
Validate normal reactions to an abnormal event.
Provide information (business card referrals, brochures, contact information, self-care ideas, etc.)
Let them know you are available to help when they need it.
Provide your contact information
If necessary, arrange a time to check-in with them.

## Slide 12
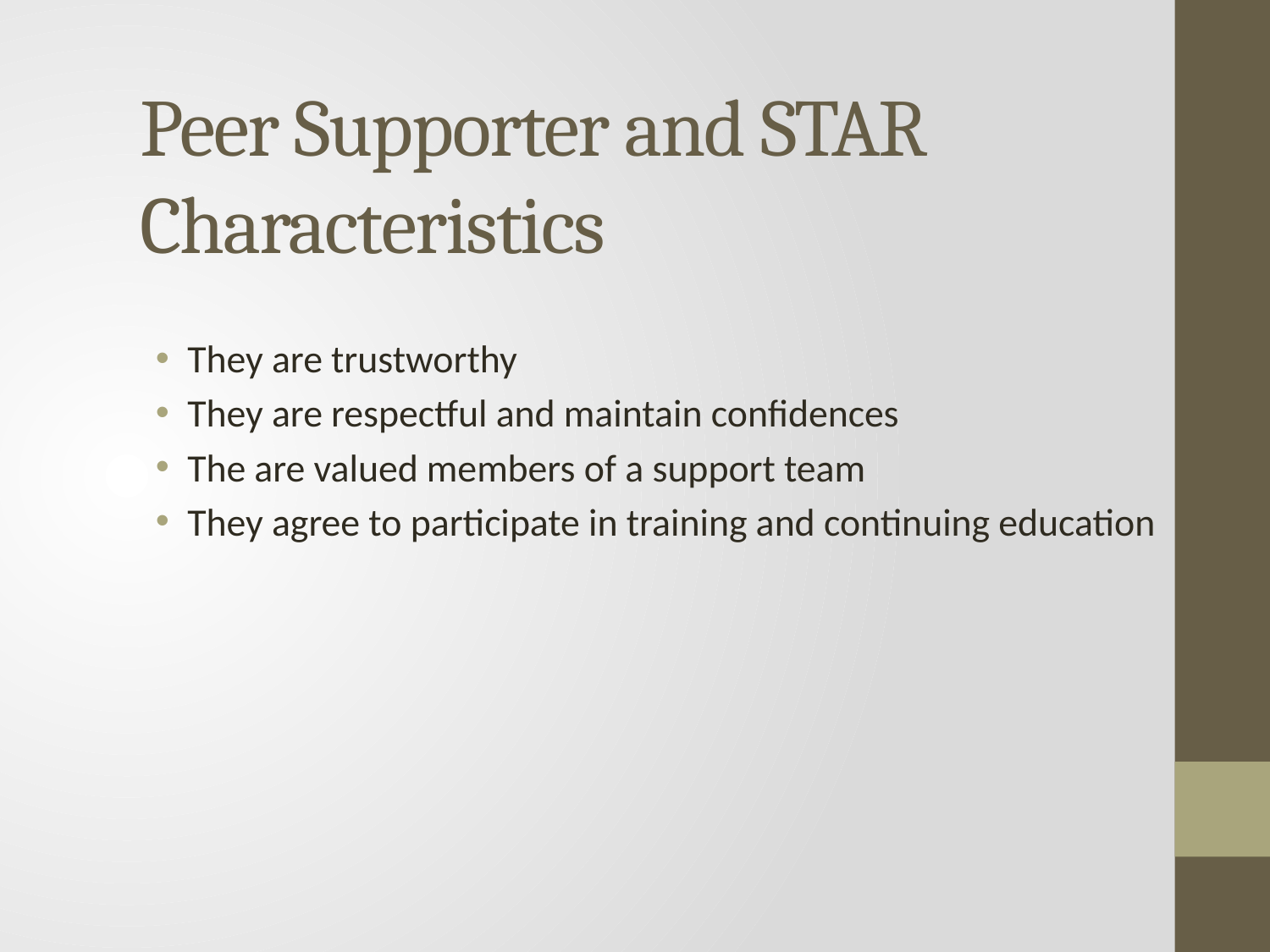

# Peer Supporter and STAR Characteristics
They are trustworthy
They are respectful and maintain confidences
The are valued members of a support team
They agree to participate in training and continuing education

## Slide 13
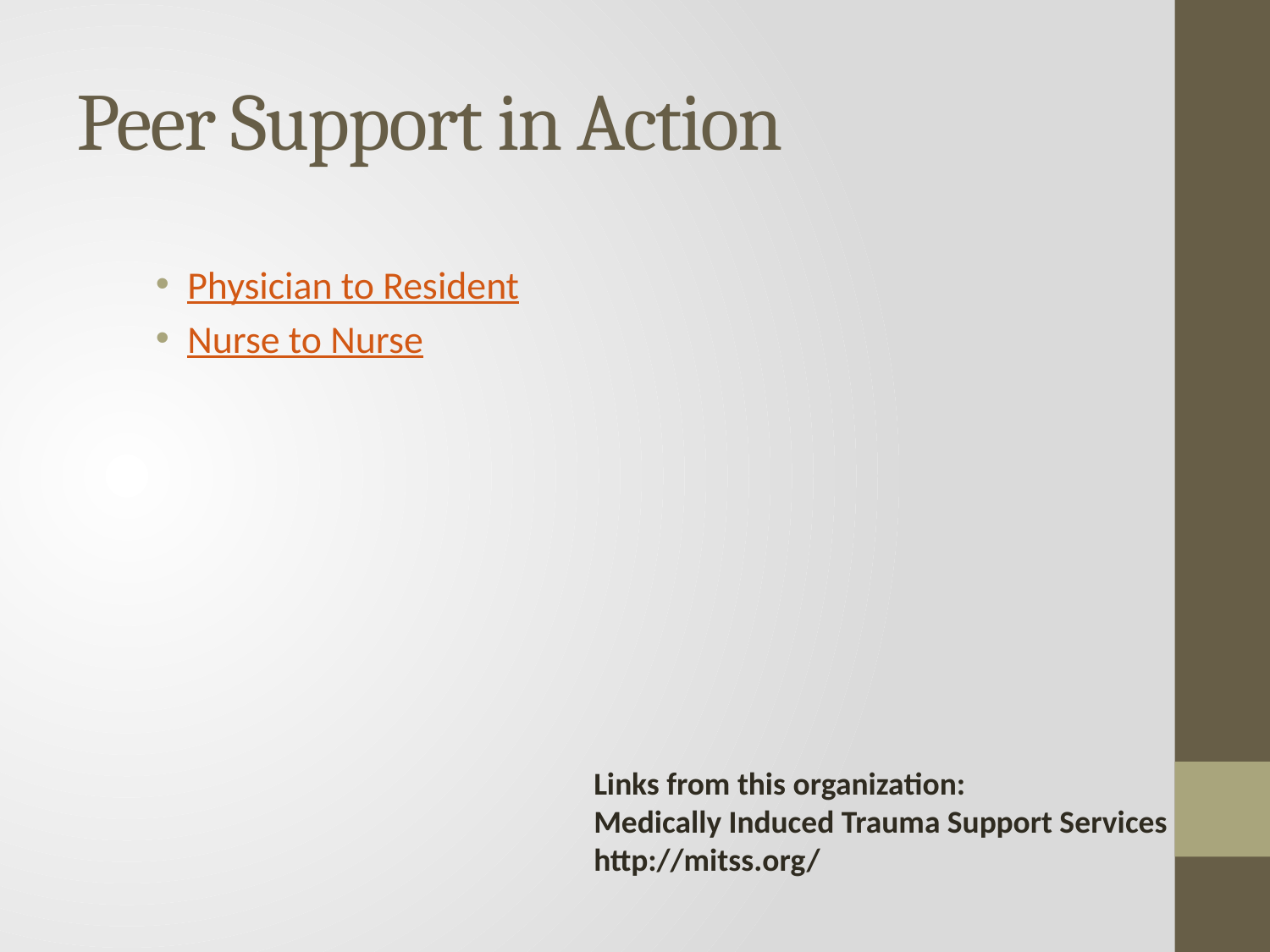

# Peer Support in Action
Physician to Resident
Nurse to Nurse
Links from this organization:
Medically Induced Trauma Support Services
http://mitss.org/

## Slide 14
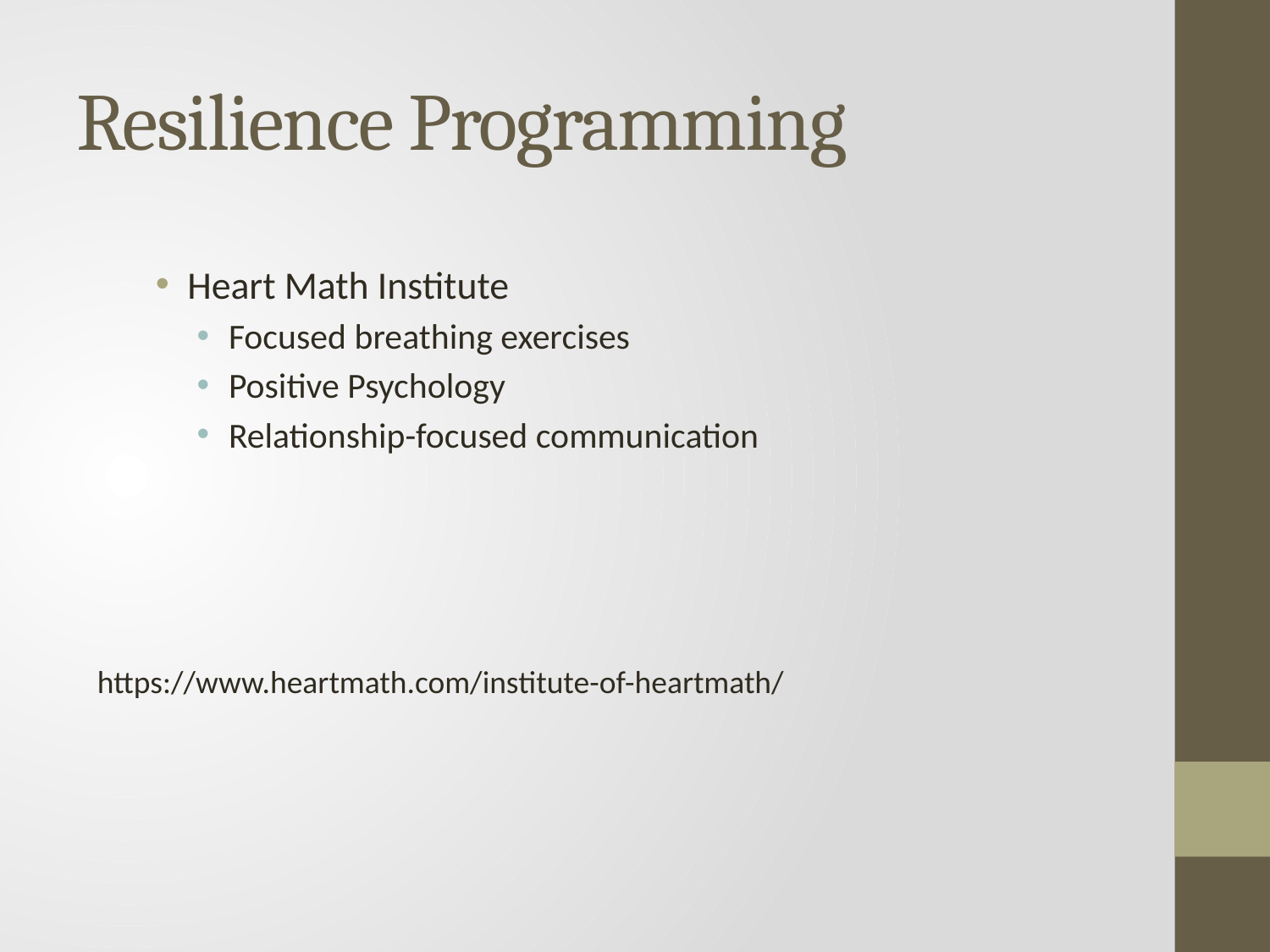

# Resilience Programming
Heart Math Institute
Focused breathing exercises
Positive Psychology
Relationship-focused communication
https://www.heartmath.com/institute-of-heartmath/

## Slide 15
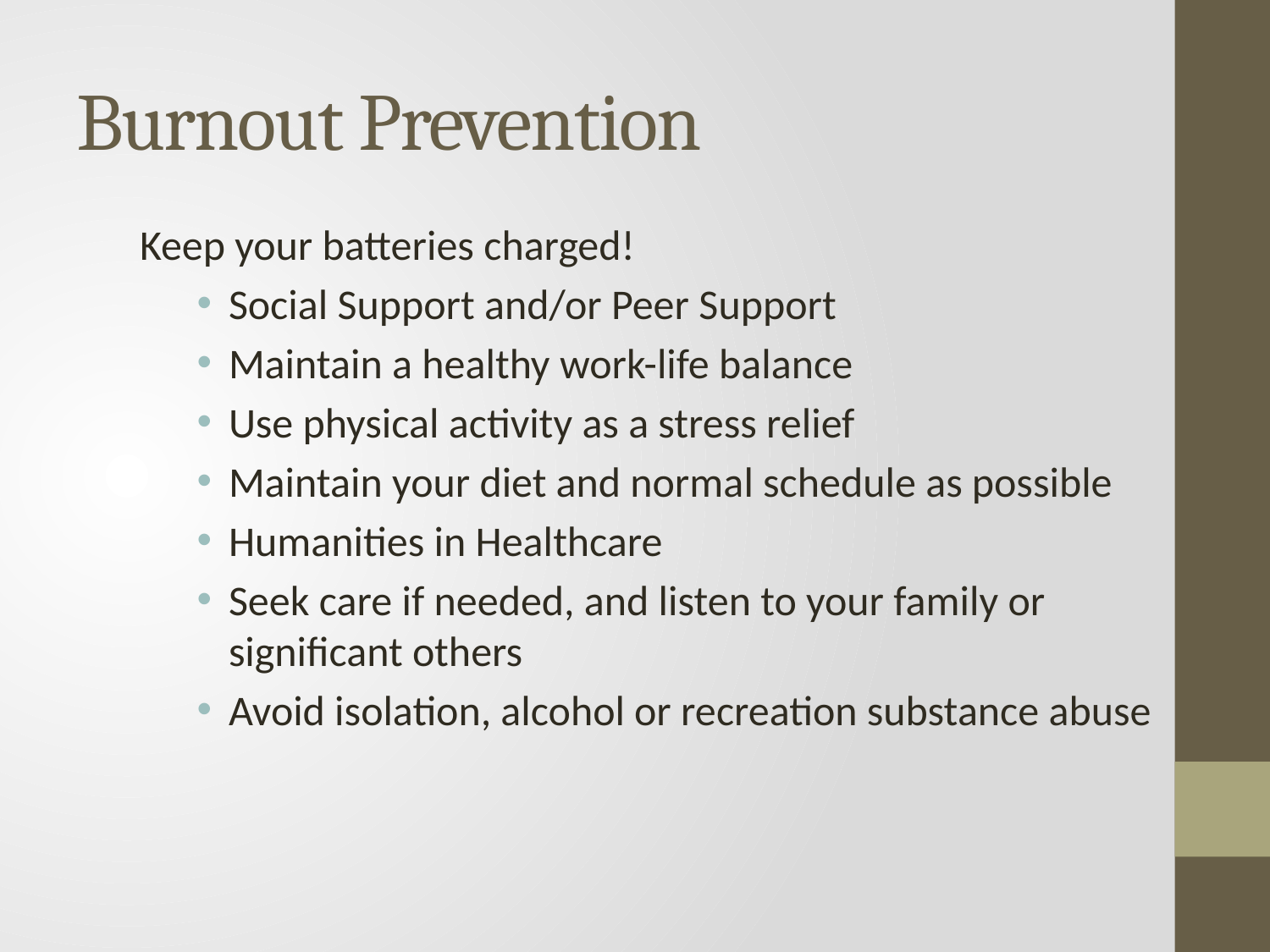

# Burnout Prevention
Keep your batteries charged!
Social Support and/or Peer Support
Maintain a healthy work-life balance
Use physical activity as a stress relief
Maintain your diet and normal schedule as possible
Humanities in Healthcare
Seek care if needed, and listen to your family or significant others
Avoid isolation, alcohol or recreation substance abuse

## Slide 16
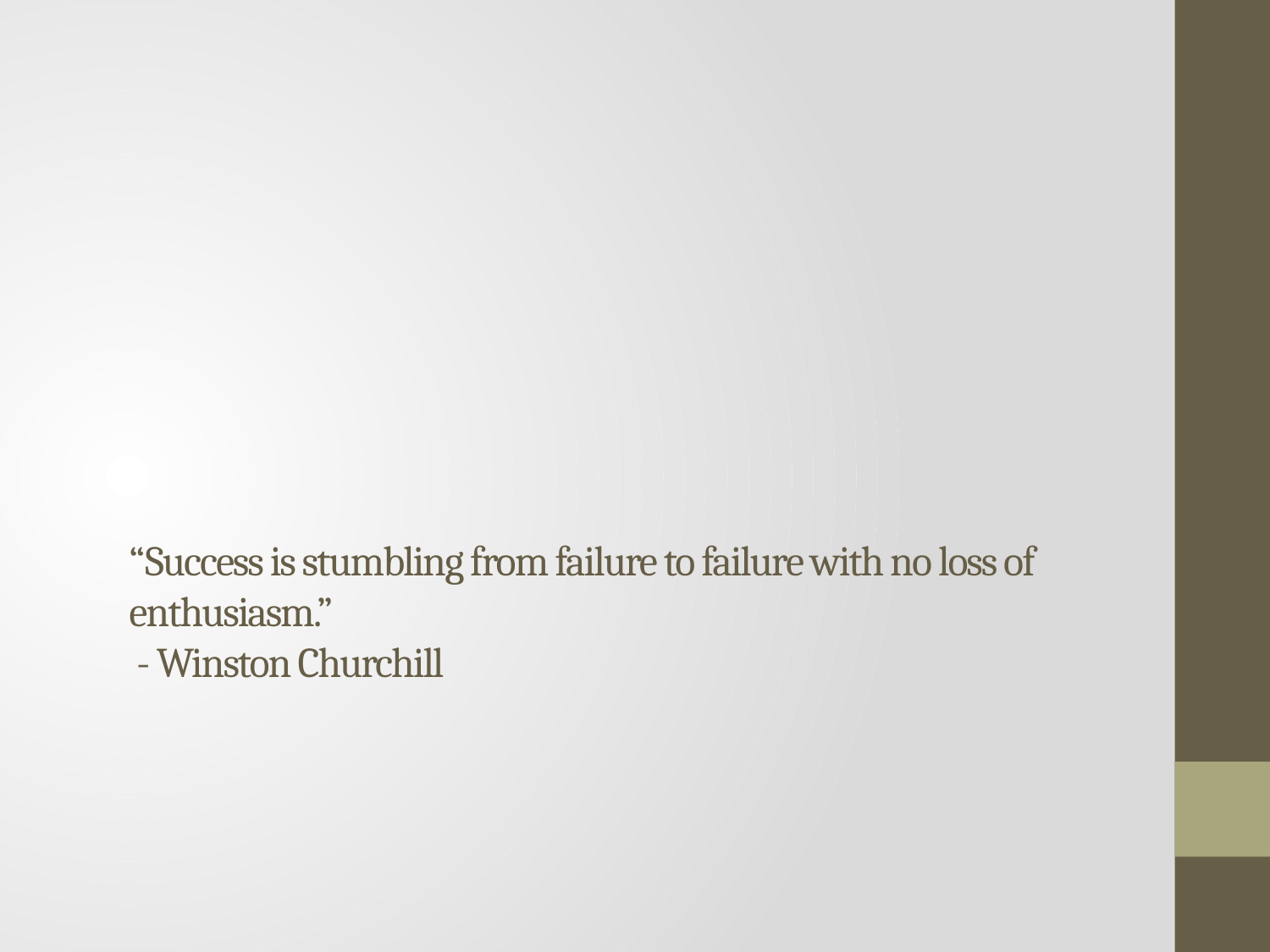

# “Success is stumbling from failure to failure with no loss of enthusiasm.”  - Winston Churchill
