# Supplementary material for: Safety in Numbers: Successful Student-Approved Case-Based Interprofessional Safety Workshop Utilizing Simulated Real-Life Safety Cases
Source: MedEdPORTAL. 2020 Jan 31;16:10874. doi: 10.15766/mep_2374-8265.10874 (PMC7065299; doi:10.15766/mep_2374-8265.10874)
Supplement: Supplementary file 1 — A. Pre- & Postevent Surveys.docx B. IPE Safety Workshop Agenda.docx C. RCA AM Session Facilitator Guide.docx D. RCA AM Session Facilitator Annotated Case Time Line.docx E. RCA AM Session Student Case Time Line.docx F. RCA AM Session Interviewee Scripts.docx G. RCA AM Session Patient Background & EWS Info.docx H. RCA AM Session Media - Radiology.docx I. RCA AM Session Media - Oxygen Tanks.docx J. Corrective Action PM Session Facilitator Guide.docx K. Corrective Action PM Session Effectiveness Chart.docx L. Corrective Action PM Session Worksheet.docx M. Executive Case Summary.docx N. Large-Group Lecture Schedule & Topic List.docx O. PPT 1 - Contributing to a Culture of Safety.pptx P. PPT 2 - Systems Improvement.pptx Q. PPT 3 - Impact of Students and Residents on QI.pptx R. PPT 4 - Presentation of Safety Case.pptx S. PPT 5 - Disclosing Medical Errors.pptx T. PPT 6 - Training for Resilience.pptx U. PPT 7 - Introduction to Improvement Plans.pptx V. Facilitator Postworkshop Survey.docx [file mep-16-10874-s001.zip › U. PPT 7 - Introduction to Improvement Plans.pptx]

## Slide 1
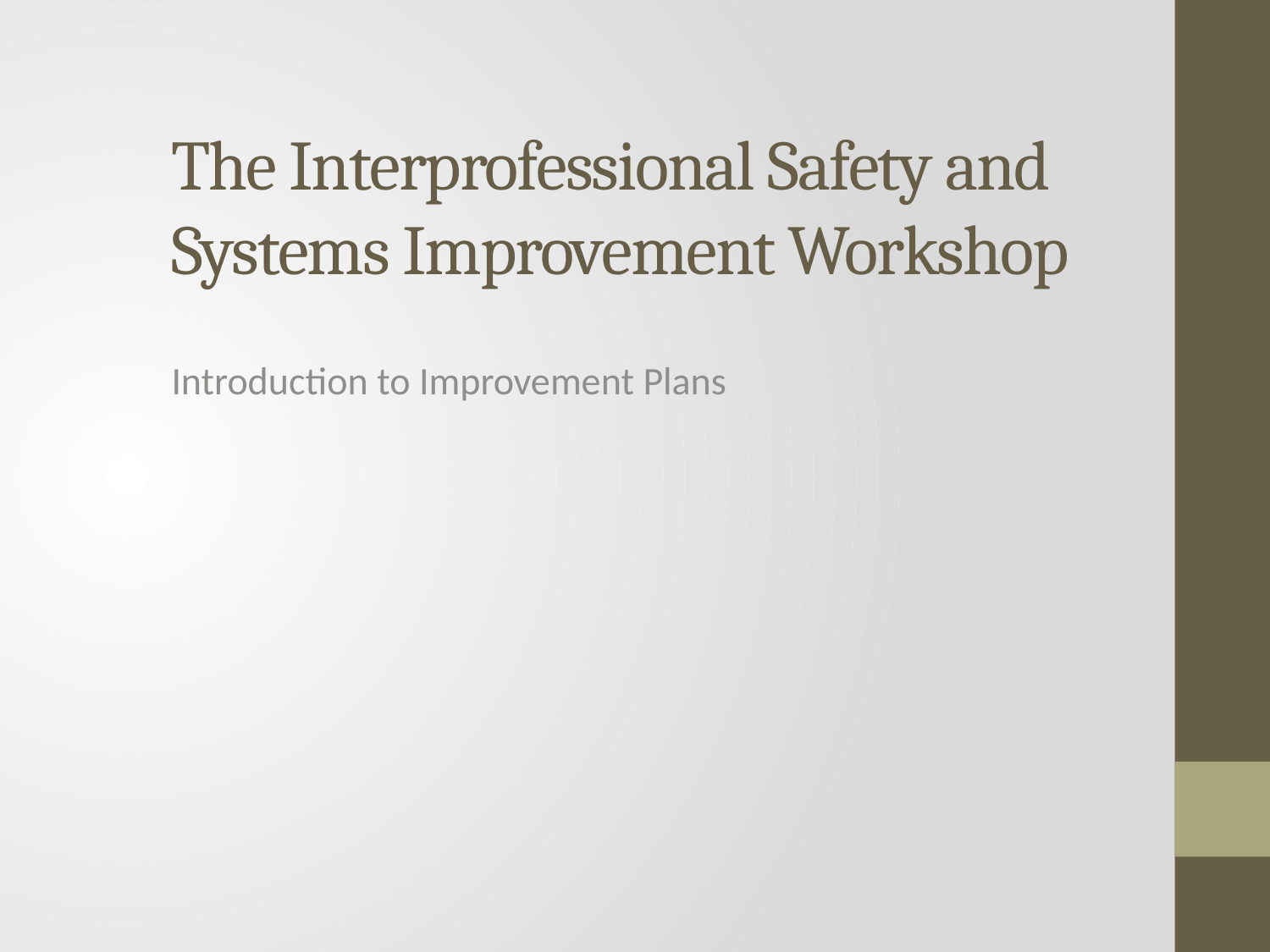

# The Interprofessional Safety and Systems Improvement Workshop
Introduction to Improvement Plans

## Slide 2
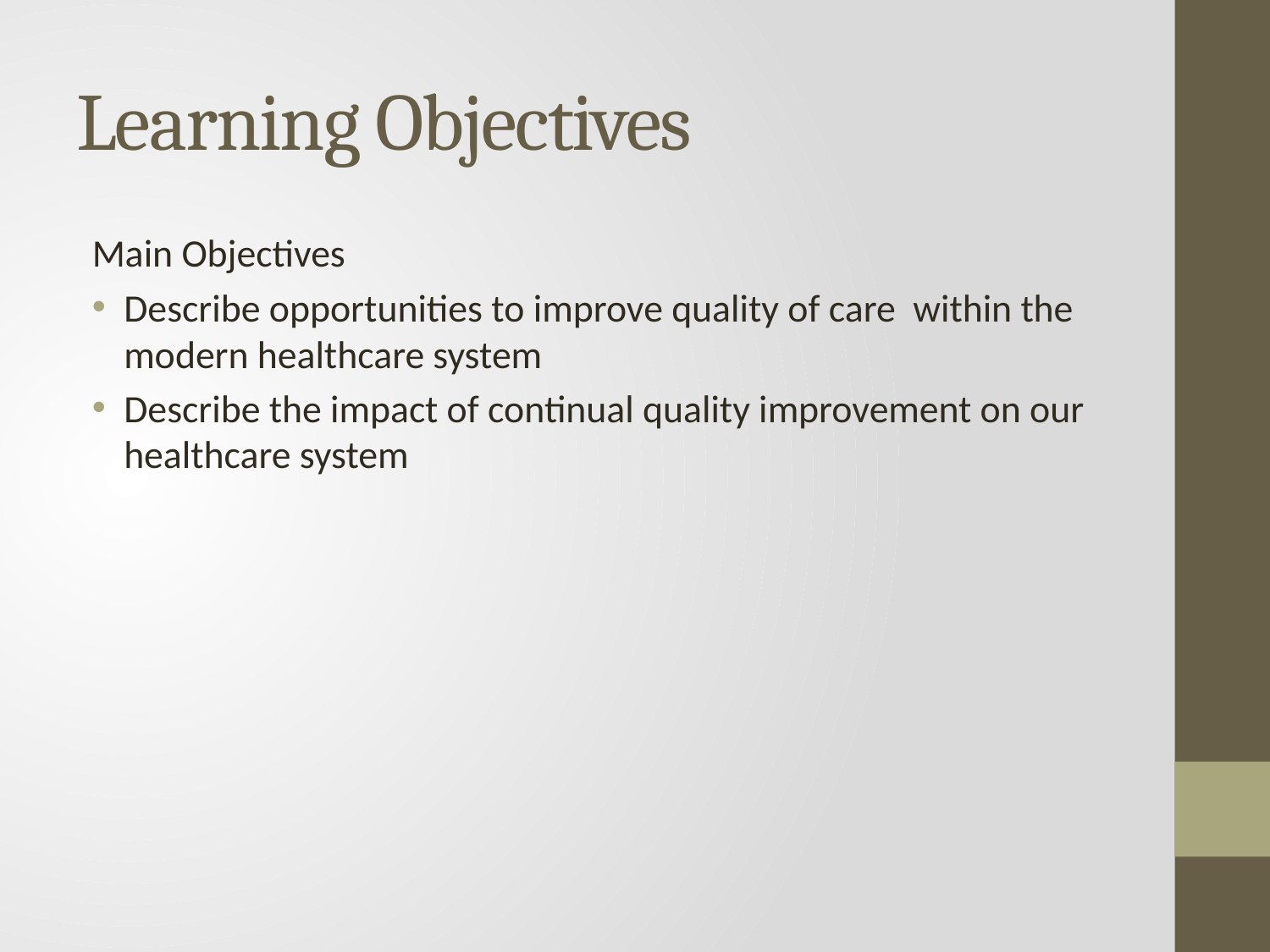

# Learning Objectives
Main Objectives
Describe opportunities to improve quality of care within the modern healthcare system
Describe the impact of continual quality improvement on our healthcare system

## Slide 3
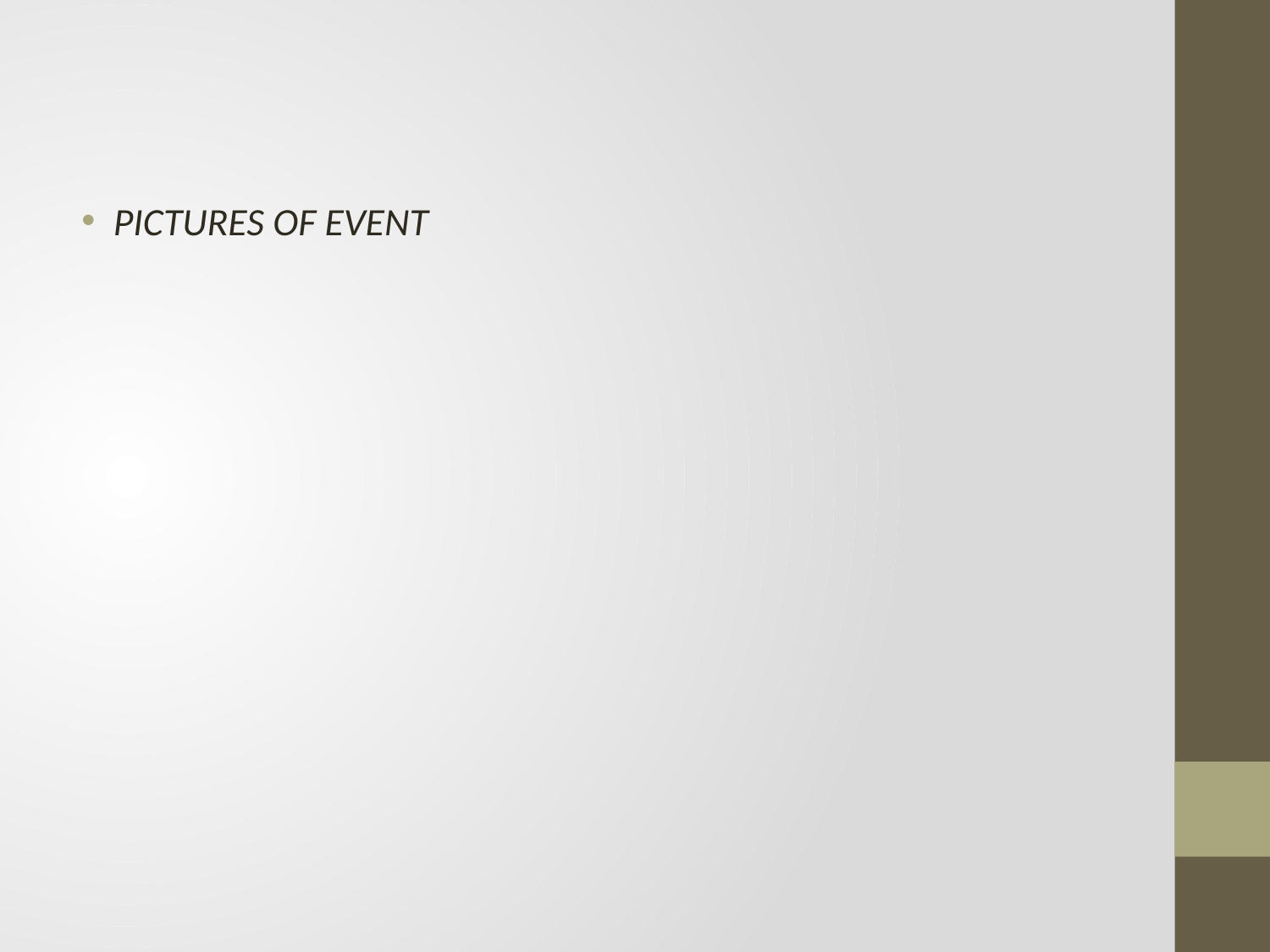

PICTURES OF EVENT

## Slide 4
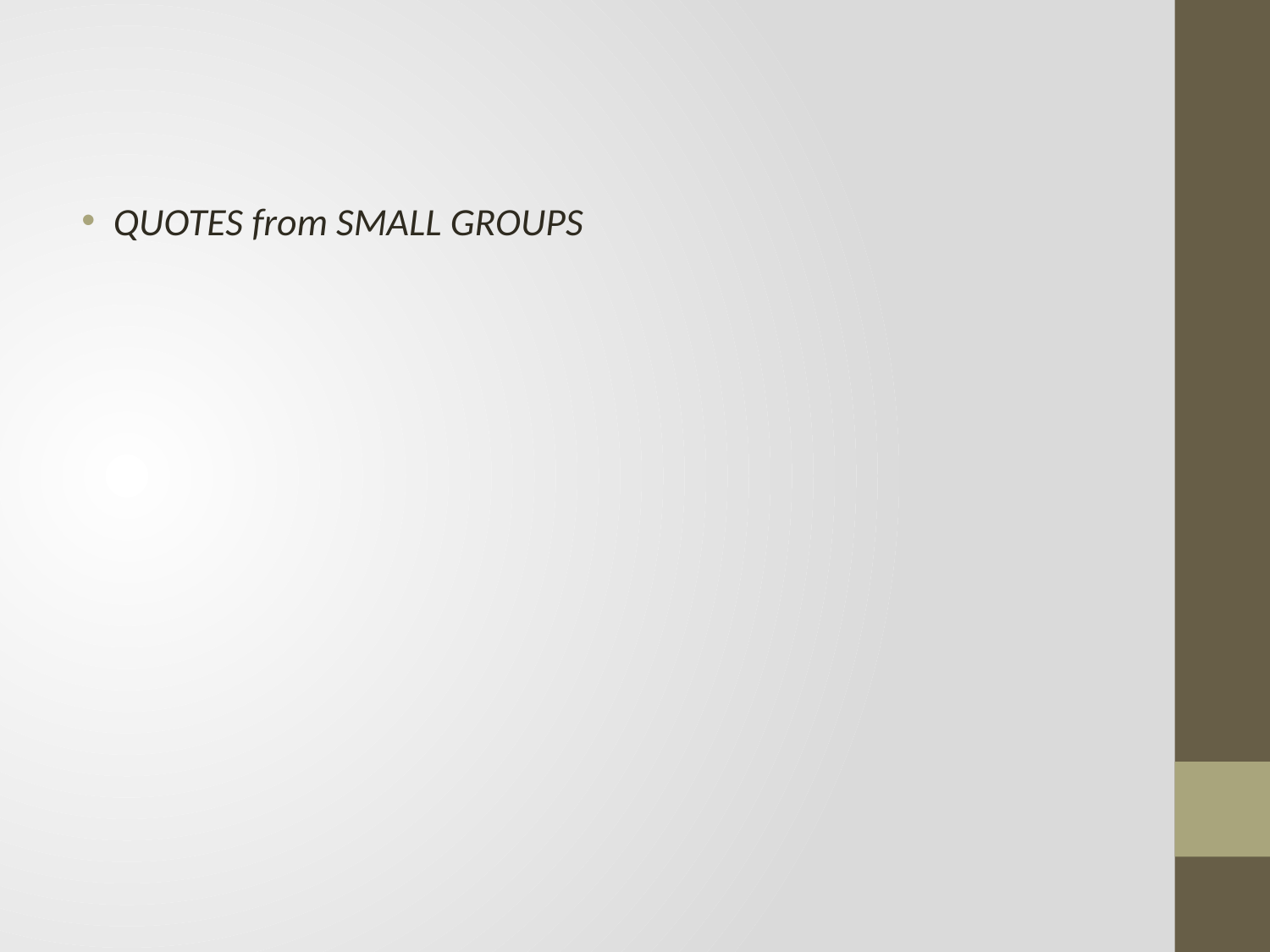

QUOTES from SMALL GROUPS

## Slide 5
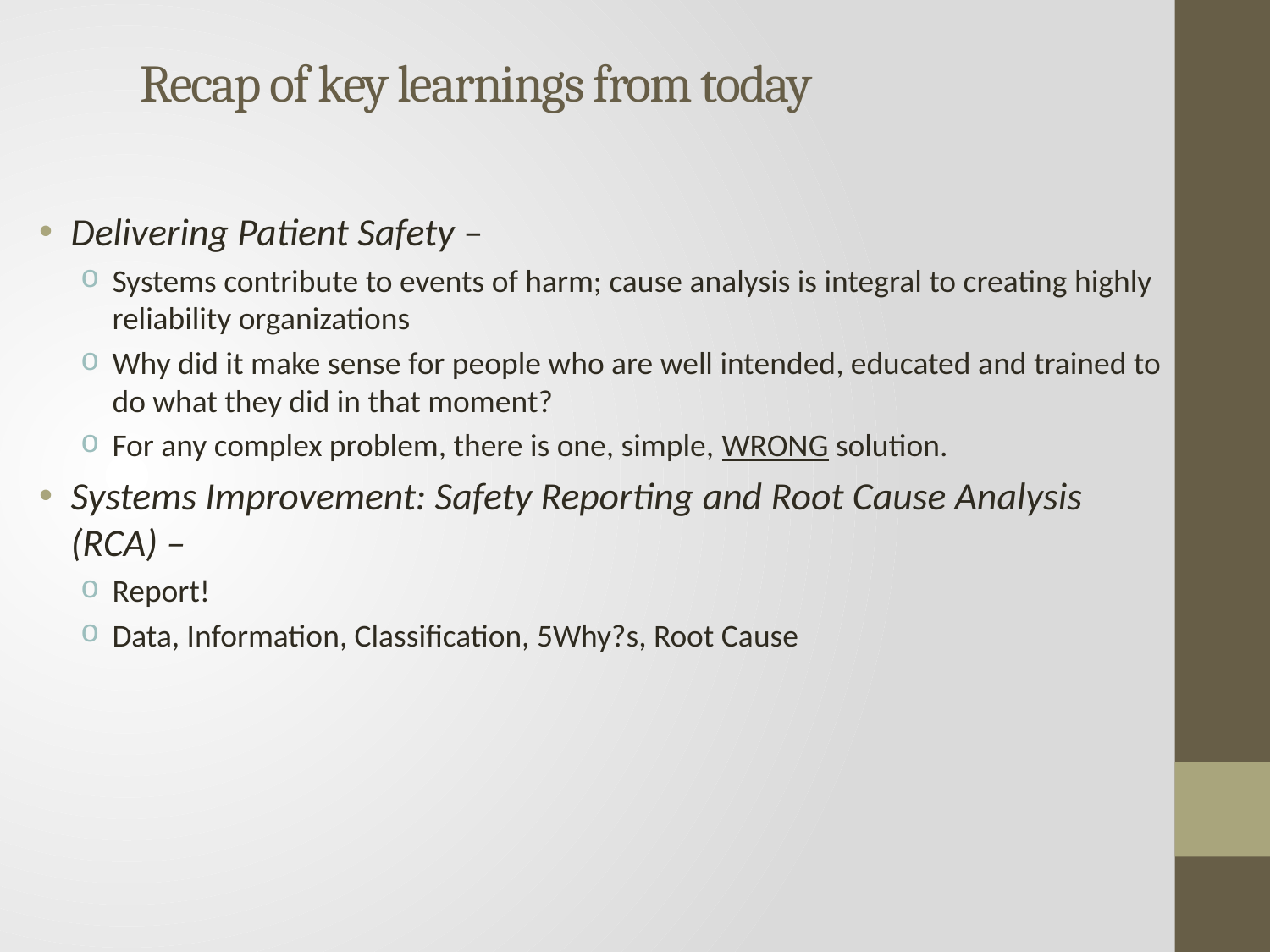

# Recap of key learnings from today
Delivering Patient Safety –
Systems contribute to events of harm; cause analysis is integral to creating highly reliability organizations
Why did it make sense for people who are well intended, educated and trained to do what they did in that moment?
For any complex problem, there is one, simple, WRONG solution.
Systems Improvement: Safety Reporting and Root Cause Analysis (RCA) –
Report!
Data, Information, Classification, 5Why?s, Root Cause

## Slide 6
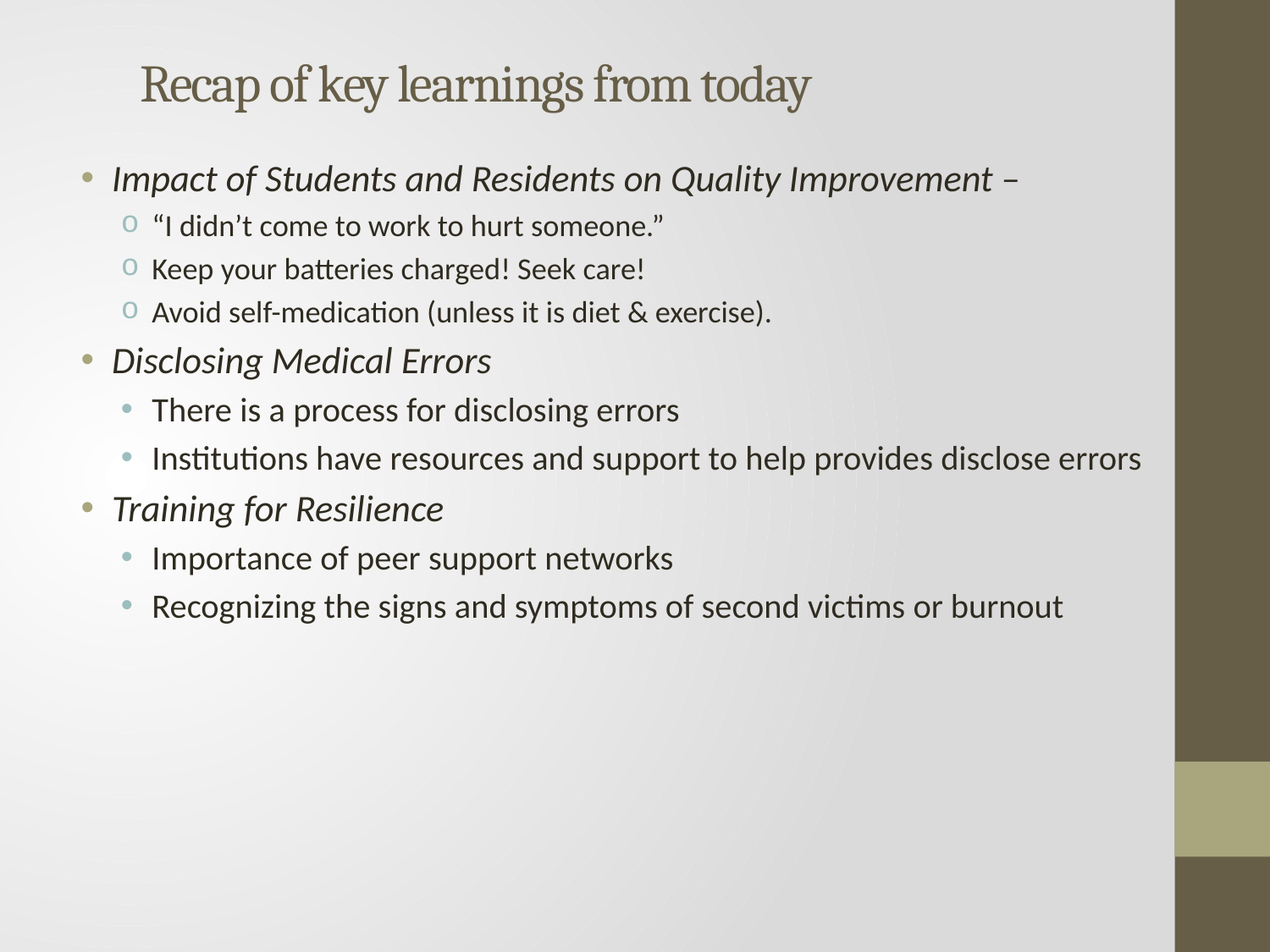

# Recap of key learnings from today
Impact of Students and Residents on Quality Improvement –
“I didn’t come to work to hurt someone.”
Keep your batteries charged! Seek care!
Avoid self-medication (unless it is diet & exercise).
Disclosing Medical Errors
There is a process for disclosing errors
Institutions have resources and support to help provides disclose errors
Training for Resilience
Importance of peer support networks
Recognizing the signs and symptoms of second victims or burnout

## Slide 7
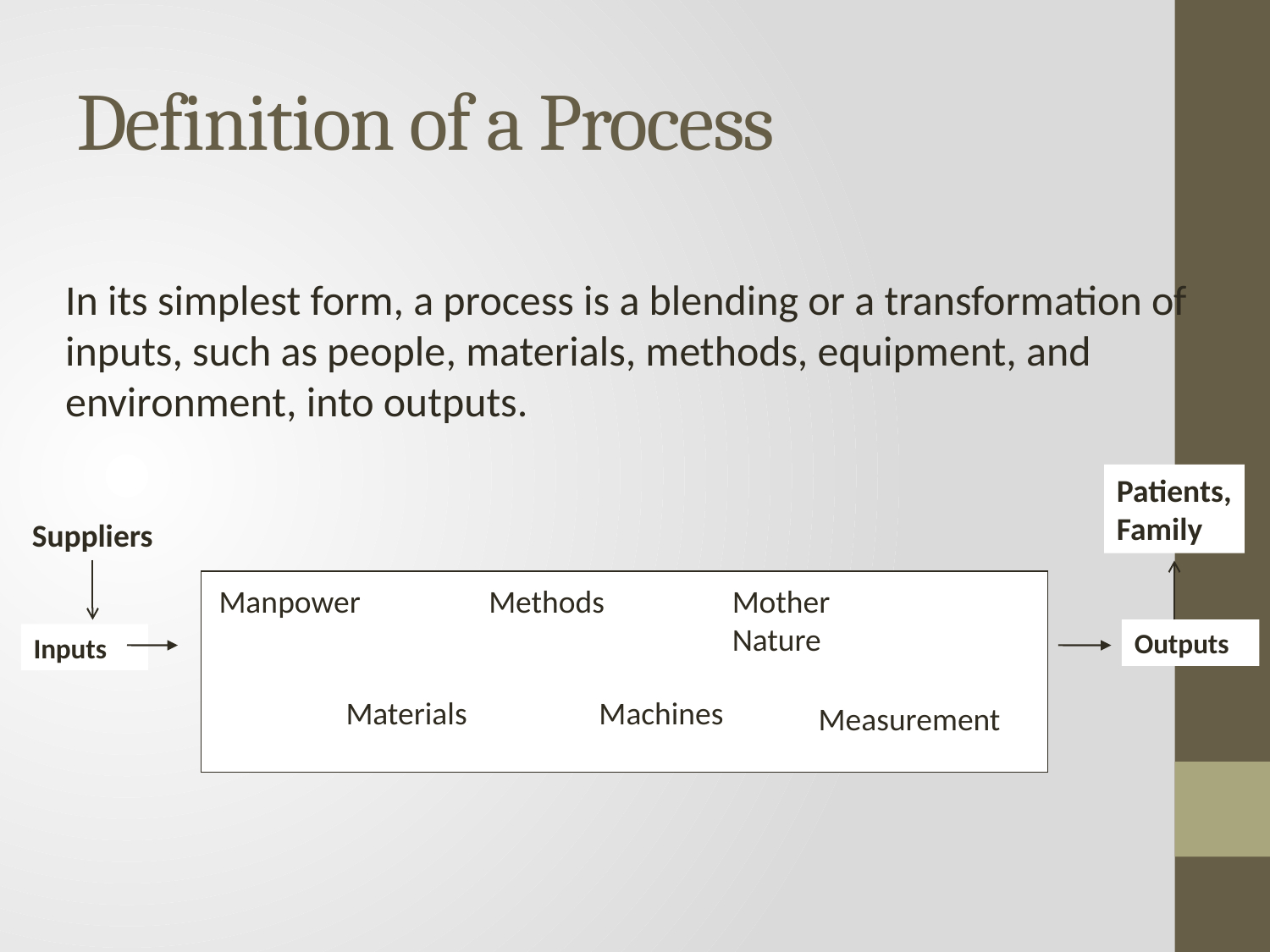

# Definition of a Process
In its simplest form, a process is a blending or a transformation of inputs, such as people, materials, methods, equipment, and environment, into outputs.
Patients,
Family
Suppliers
Manpower
Methods
Mother Nature
Outputs
Inputs
Materials
Machines
Measurement

## Slide 8
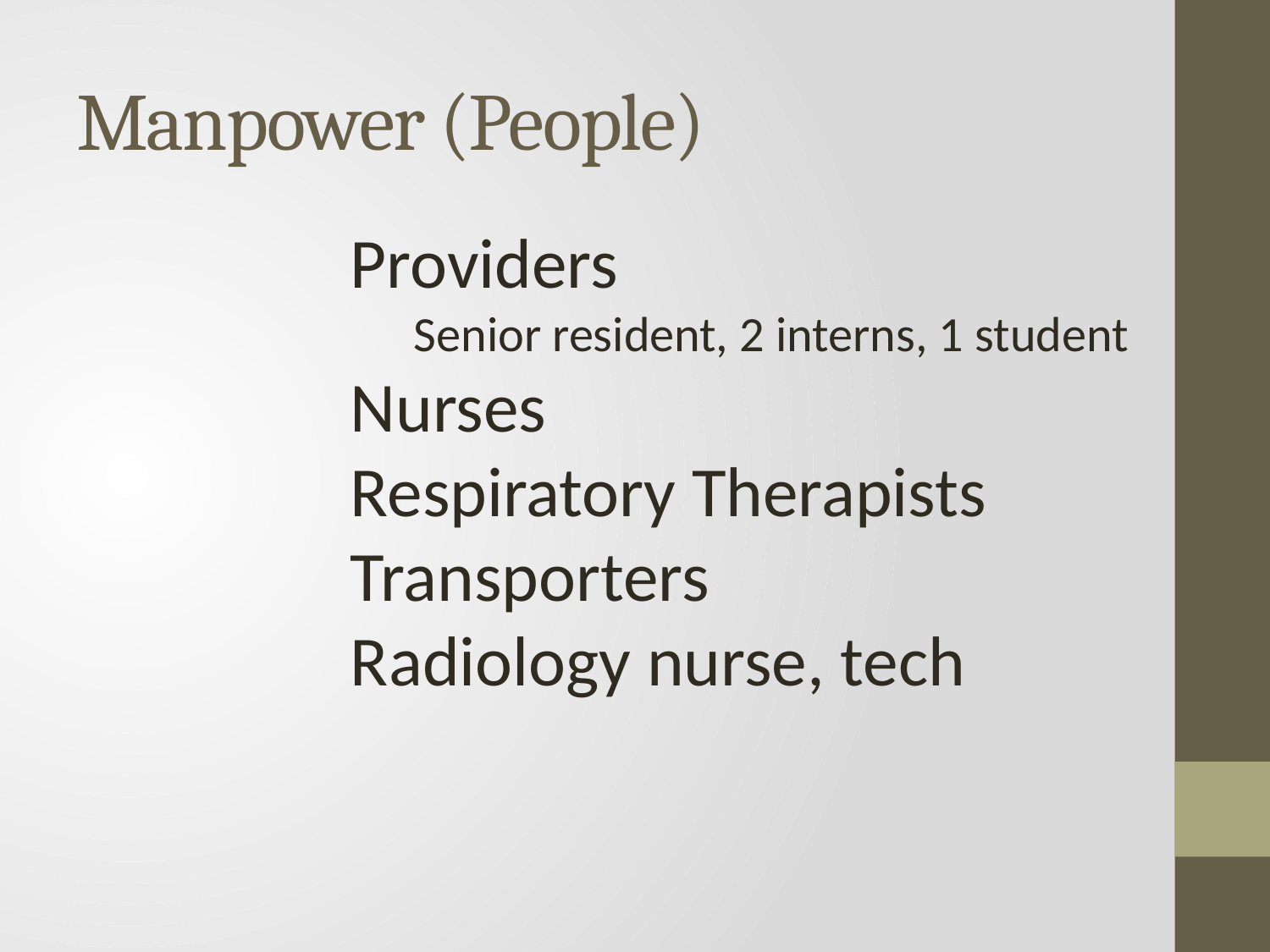

# Manpower (People)
Providers
Senior resident, 2 interns, 1 student
Nurses
Respiratory Therapists
Transporters
Radiology nurse, tech

## Slide 9
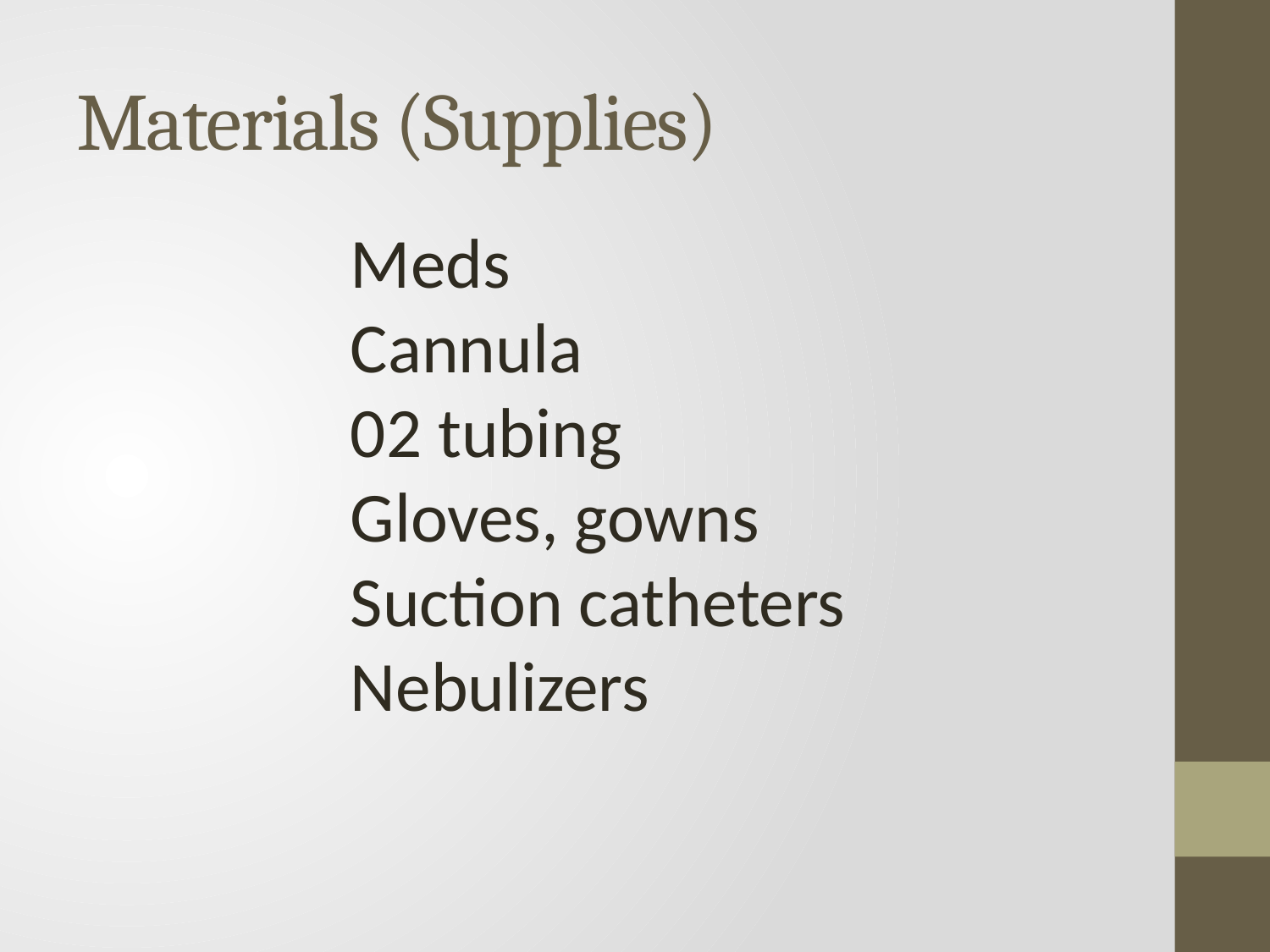

# Materials (Supplies)
Meds
Cannula
02 tubing
Gloves, gowns
Suction catheters
Nebulizers

## Slide 10
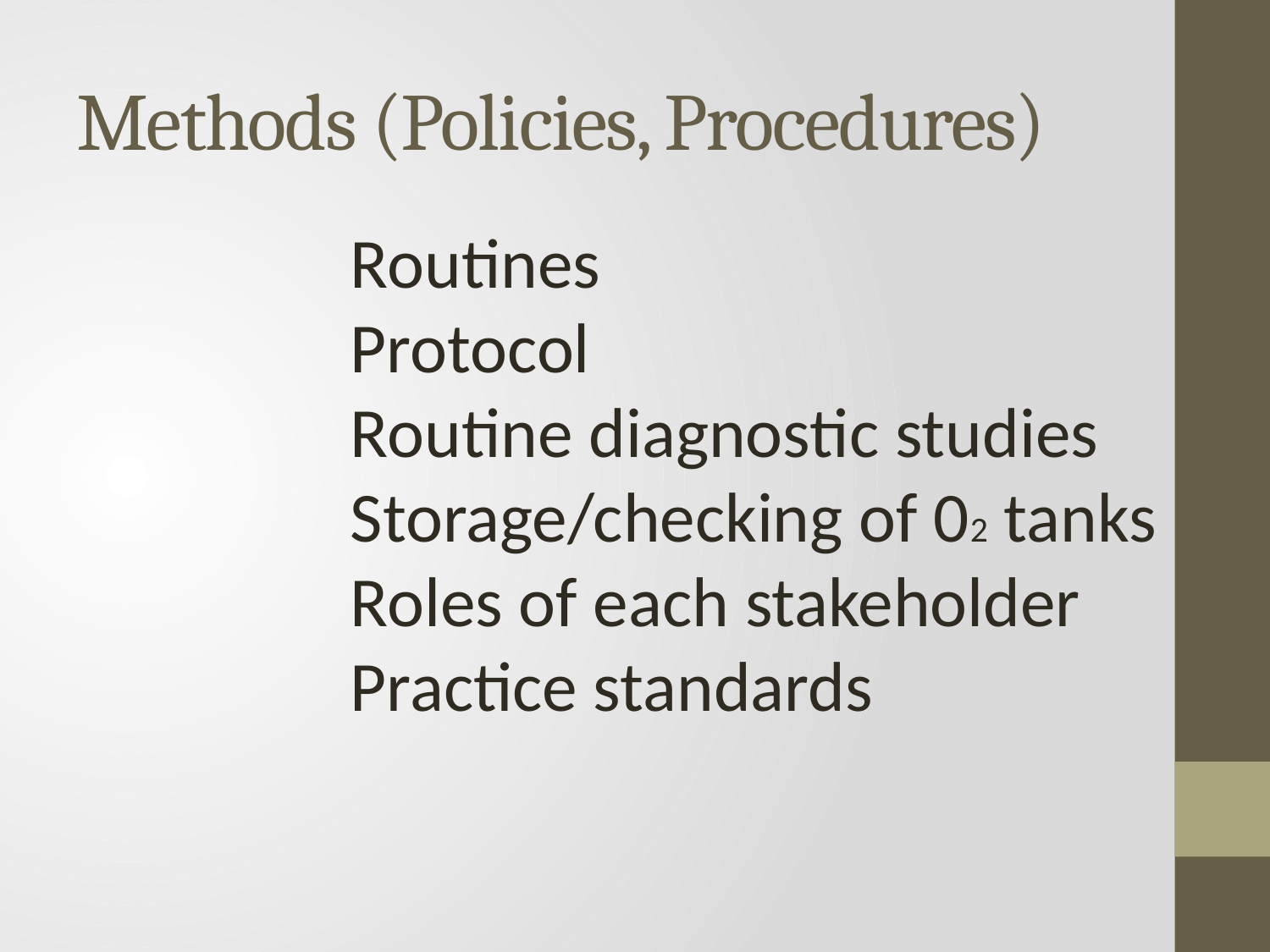

# Methods (Policies, Procedures)
Routines
Protocol
Routine diagnostic studies
Storage/checking of 02 tanks
Roles of each stakeholder
Practice standards

## Slide 11
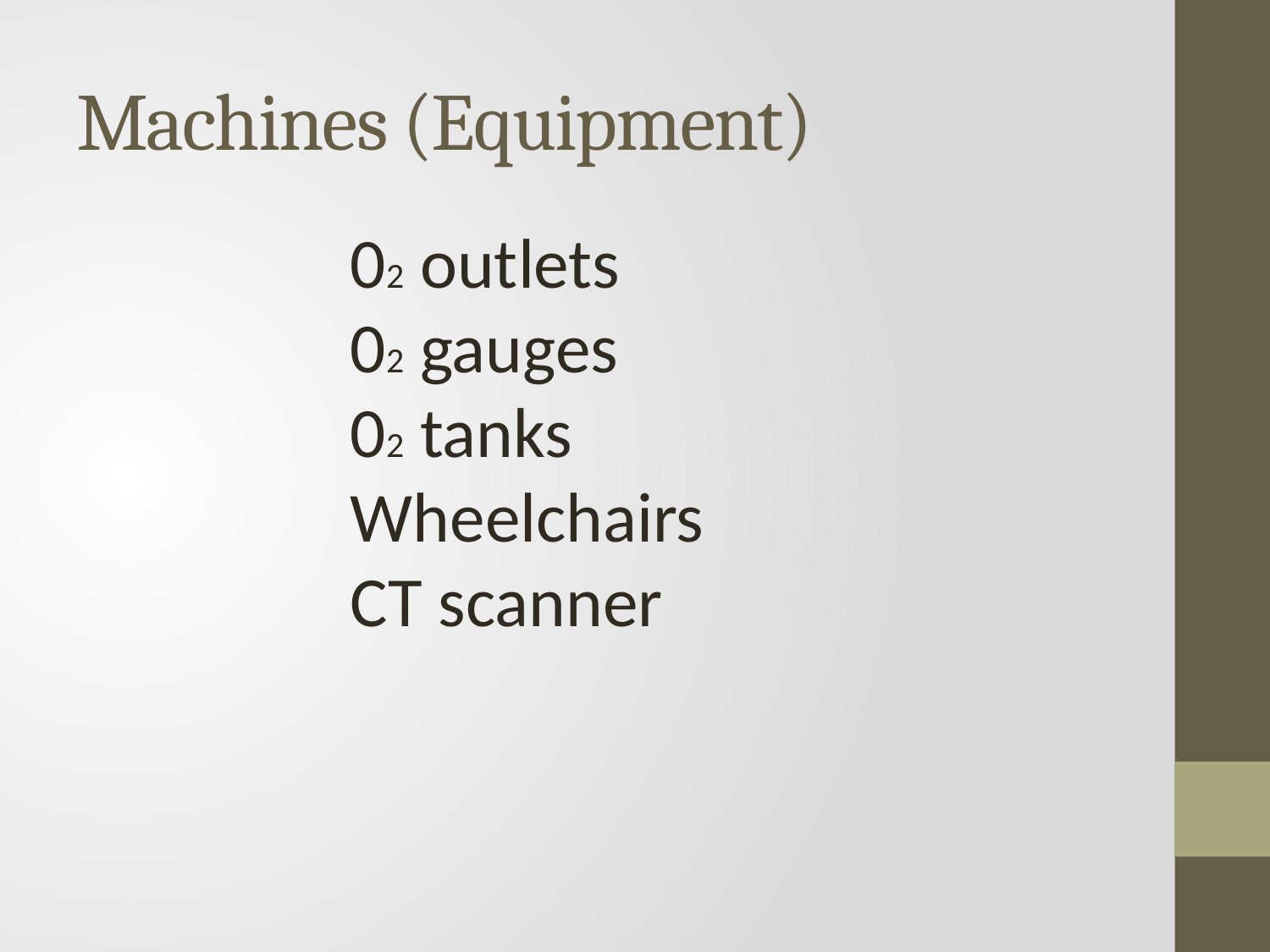

# Machines (Equipment)
02 outlets
02 gauges
02 tanks
Wheelchairs
CT scanner

## Slide 12
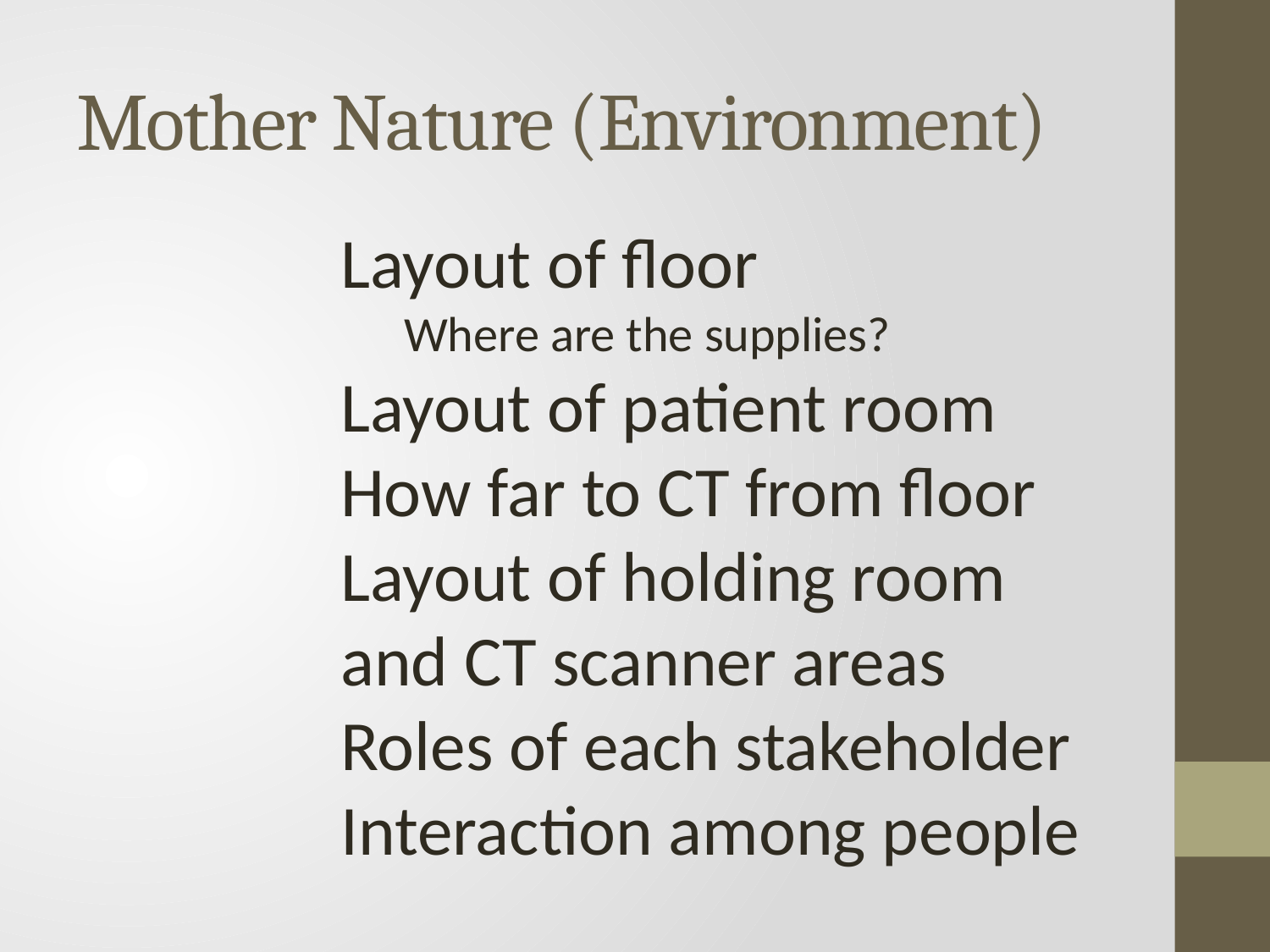

# Mother Nature (Environment)
Layout of floor
Where are the supplies?
Layout of patient room
How far to CT from floor
Layout of holding room
and CT scanner areas
Roles of each stakeholder
Interaction among people

## Slide 13
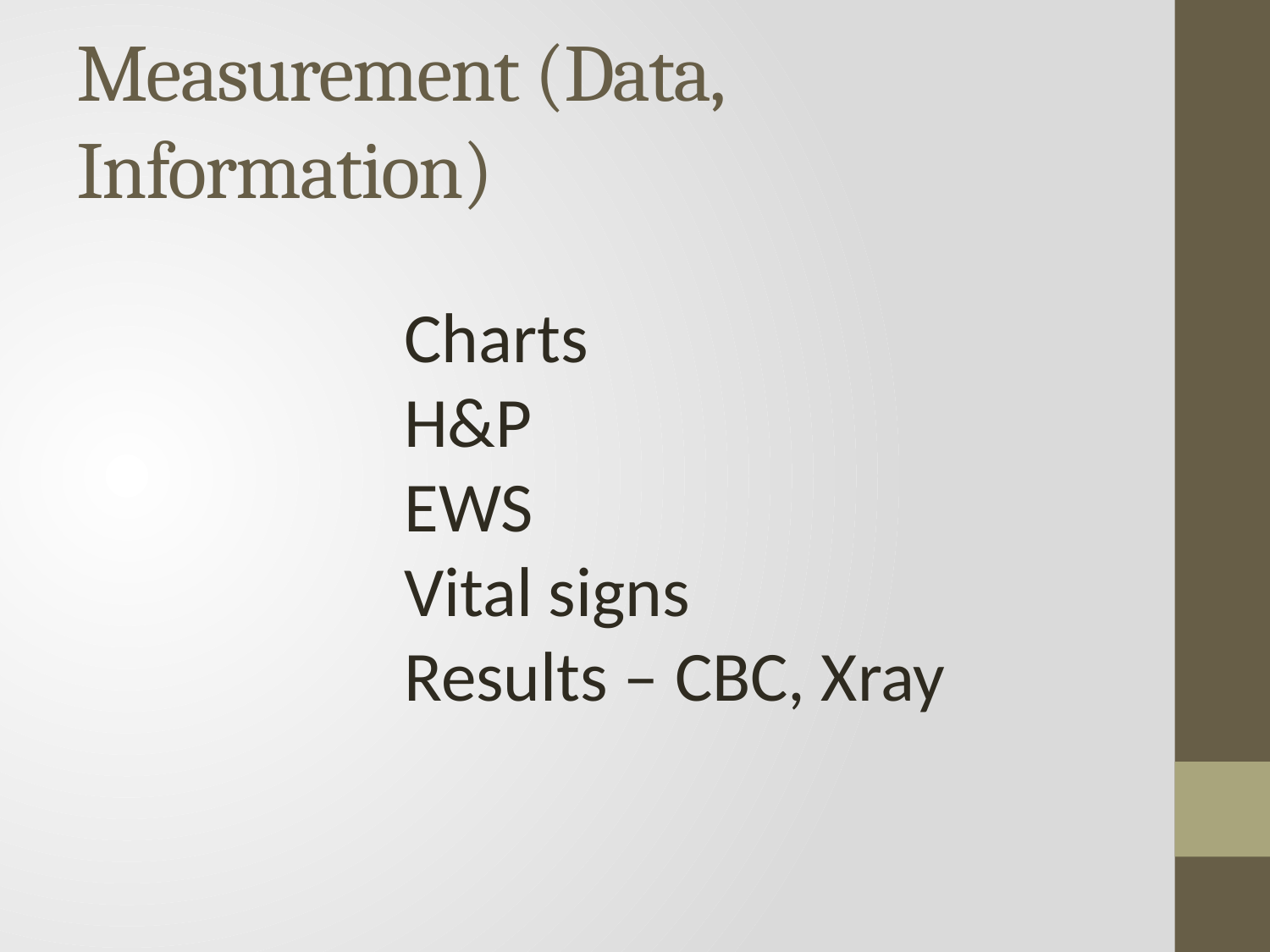

# Measurement (Data, Information)
Charts
H&P
EWS
Vital signs
Results – CBC, Xray

## Slide 14
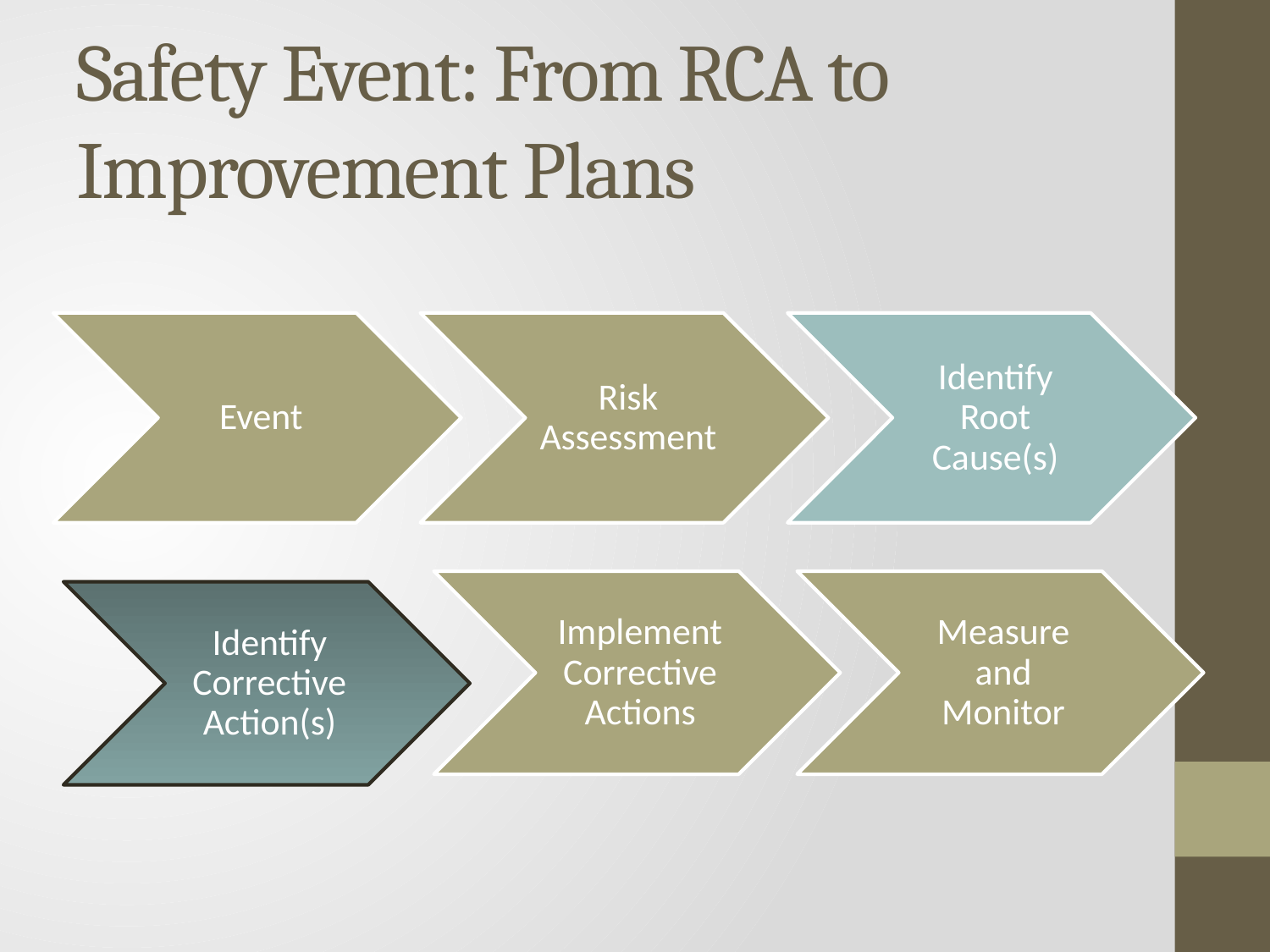

# Safety Event: From RCA to Improvement Plans
Implement Corrective Actions
Measure and Monitor
Identify Corrective Action(s)

## Slide 15
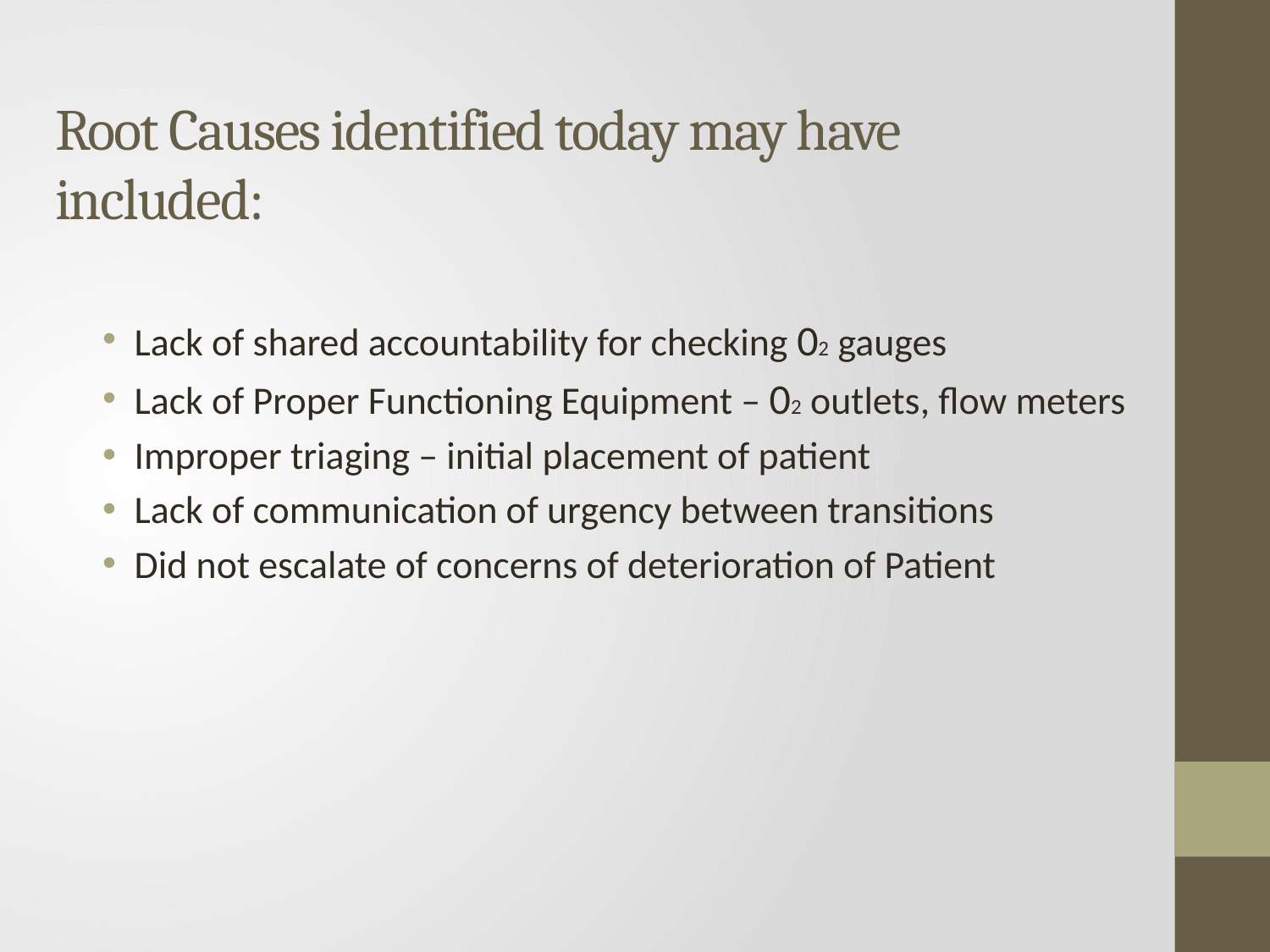

# Root Causes identified today may have included:
Lack of shared accountability for checking 02 gauges
Lack of Proper Functioning Equipment – 02 outlets, flow meters
Improper triaging – initial placement of patient
Lack of communication of urgency between transitions
Did not escalate of concerns of deterioration of Patient

## Slide 16
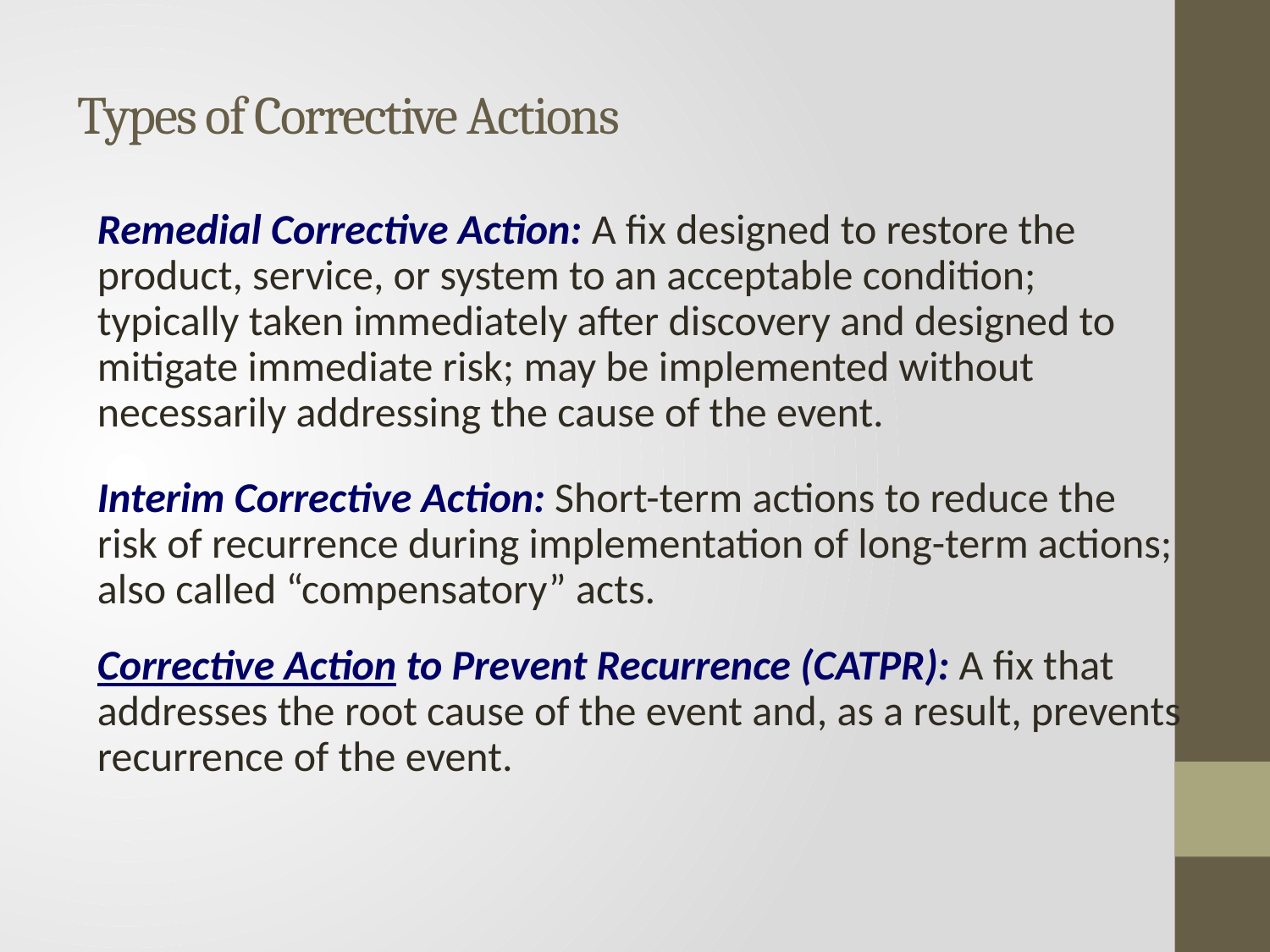

# Types of Corrective Actions
Remedial Corrective Action: A fix designed to restore the product, service, or system to an acceptable condition; typically taken immediately after discovery and designed to mitigate immediate risk; may be implemented without necessarily addressing the cause of the event.
Interim Corrective Action: Short-term actions to reduce the risk of recurrence during implementation of long-term actions; also called “compensatory” acts.
Corrective Action to Prevent Recurrence (CATPR): A fix that addresses the root cause of the event and, as a result, prevents recurrence of the event.

## Slide 17
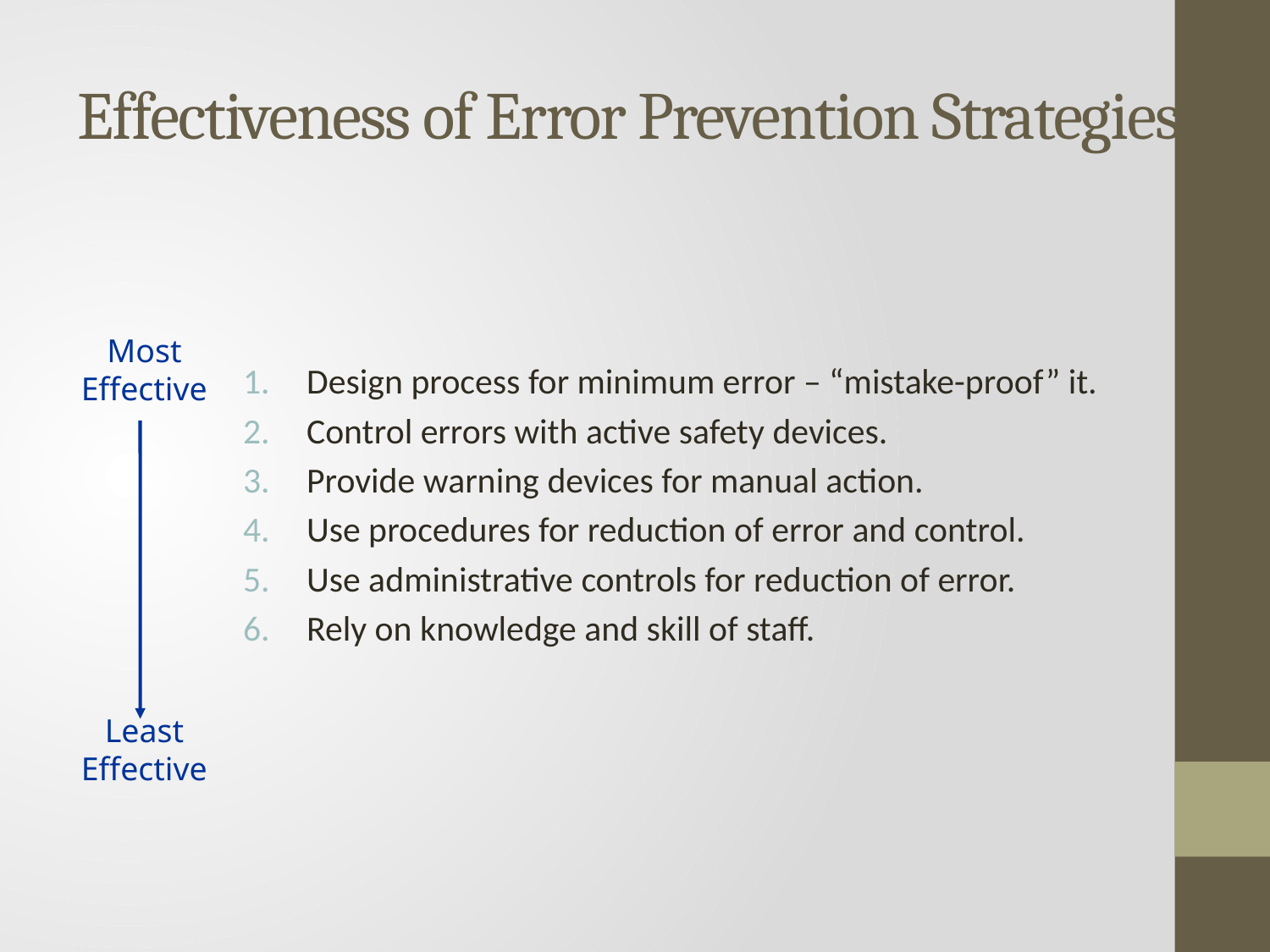

# Effectiveness of Error Prevention Strategies
Most Effective
Least Effective
Design process for minimum error – “mistake-proof” it.
Control errors with active safety devices.
Provide warning devices for manual action.
Use procedures for reduction of error and control.
Use administrative controls for reduction of error.
Rely on knowledge and skill of staff.

## Slide 18
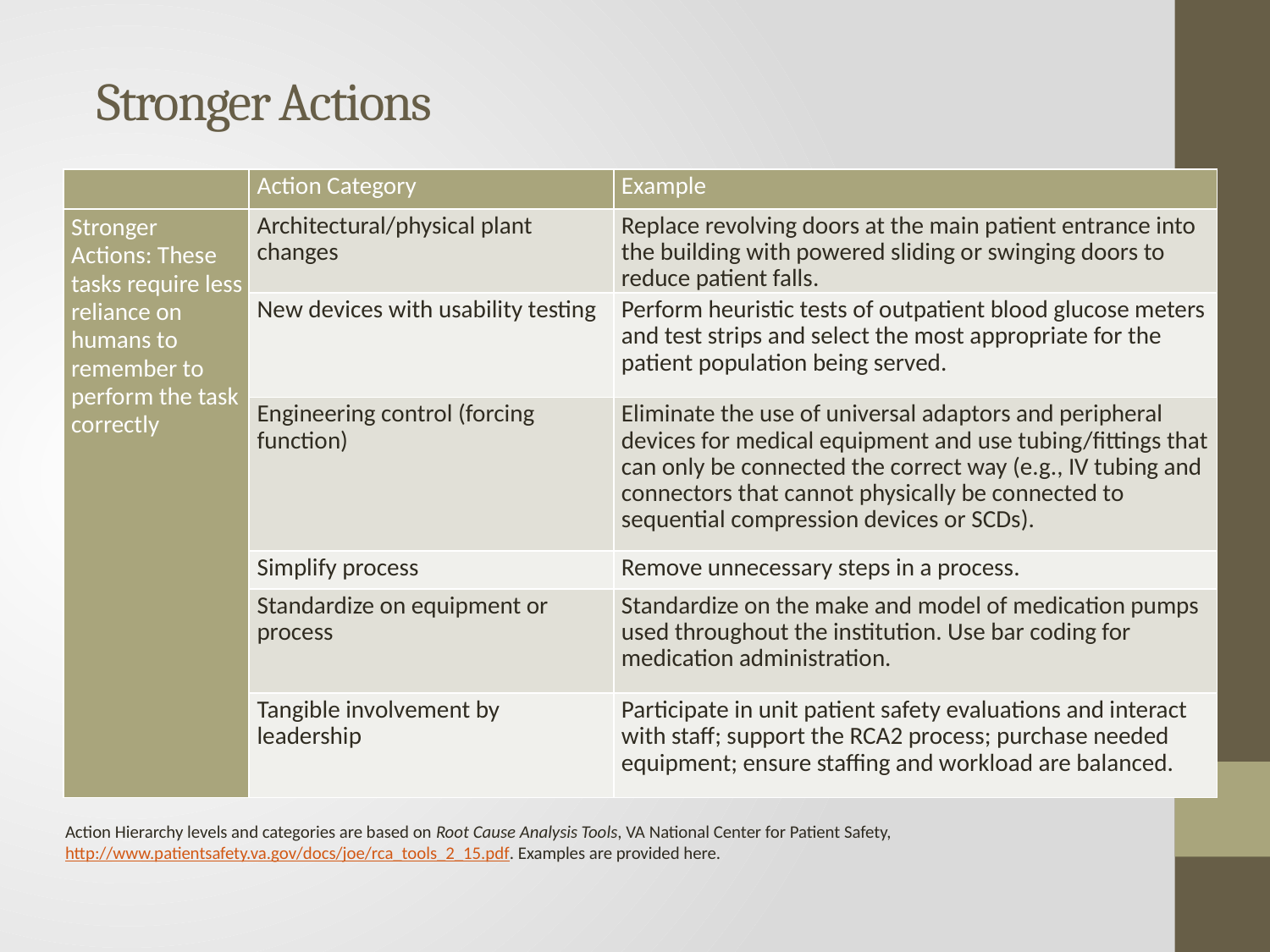

# Stronger Actions
| | Action Category | Example |
| --- | --- | --- |
| Stronger Actions: These tasks require less reliance on humans to remember to perform the task correctly | Architectural/physical plant changes | Replace revolving doors at the main patient entrance into the building with powered sliding or swinging doors to reduce patient falls. |
| | New devices with usability testing | Perform heuristic tests of outpatient blood glucose meters and test strips and select the most appropriate for the patient population being served. |
| | Engineering control (forcing function) | Eliminate the use of universal adaptors and peripheral devices for medical equipment and use tubing/fittings that can only be connected the correct way (e.g., IV tubing and connectors that cannot physically be connected to sequential compression devices or SCDs). |
| | Simplify process | Remove unnecessary steps in a process. |
| | Standardize on equipment or process | Standardize on the make and model of medication pumps used throughout the institution. Use bar coding for medication administration. |
| | Tangible involvement by leadership | Participate in unit patient safety evaluations and interact with staff; support the RCA2 process; purchase needed equipment; ensure staffing and workload are balanced. |
Action Hierarchy levels and categories are based on Root Cause Analysis Tools, VA National Center for Patient Safety, http://www.patientsafety.va.gov/docs/joe/rca_tools_2_15.pdf. Examples are provided here.

## Slide 19
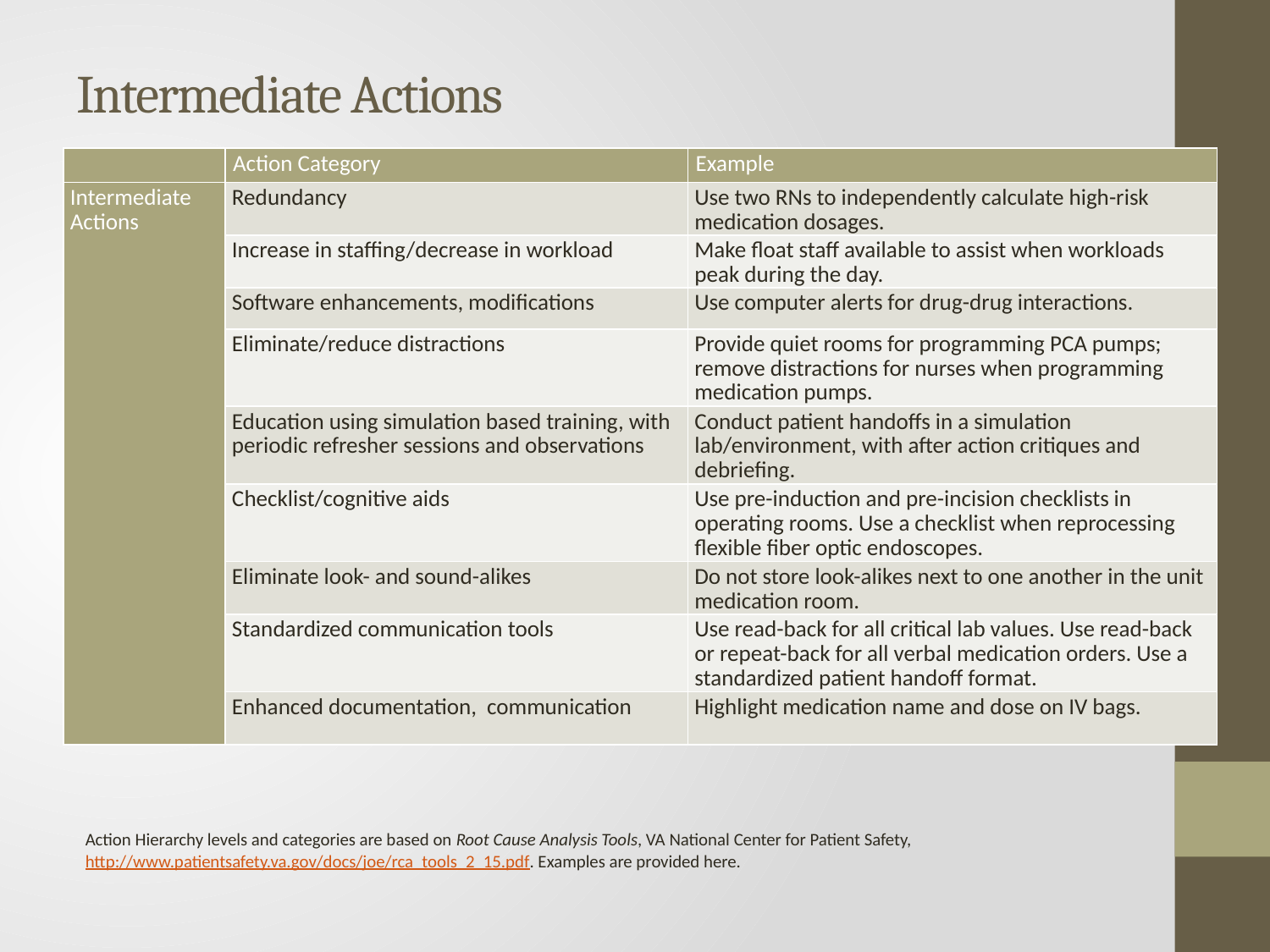

# Intermediate Actions
| | Action Category | Example |
| --- | --- | --- |
| Intermediate Actions | Redundancy | Use two RNs to independently calculate high-risk medication dosages. |
| | Increase in staffing/decrease in workload | Make float staff available to assist when workloads peak during the day. |
| | Software enhancements, modifications | Use computer alerts for drug-drug interactions. |
| | Eliminate/reduce distractions | Provide quiet rooms for programming PCA pumps; remove distractions for nurses when programming medication pumps. |
| | Education using simulation based training, with periodic refresher sessions and observations | Conduct patient handoffs in a simulation lab/environment, with after action critiques and debriefing. |
| | Checklist/cognitive aids | Use pre-induction and pre-incision checklists in operating rooms. Use a checklist when reprocessing flexible fiber optic endoscopes. |
| | Eliminate look- and sound-alikes | Do not store look-alikes next to one another in the unit medication room. |
| | Standardized communication tools | Use read-back for all critical lab values. Use read-back or repeat-back for all verbal medication orders. Use a standardized patient handoff format. |
| | Enhanced documentation, communication | Highlight medication name and dose on IV bags. |
Action Hierarchy levels and categories are based on Root Cause Analysis Tools, VA National Center for Patient Safety, http://www.patientsafety.va.gov/docs/joe/rca_tools_2_15.pdf. Examples are provided here.

## Slide 20
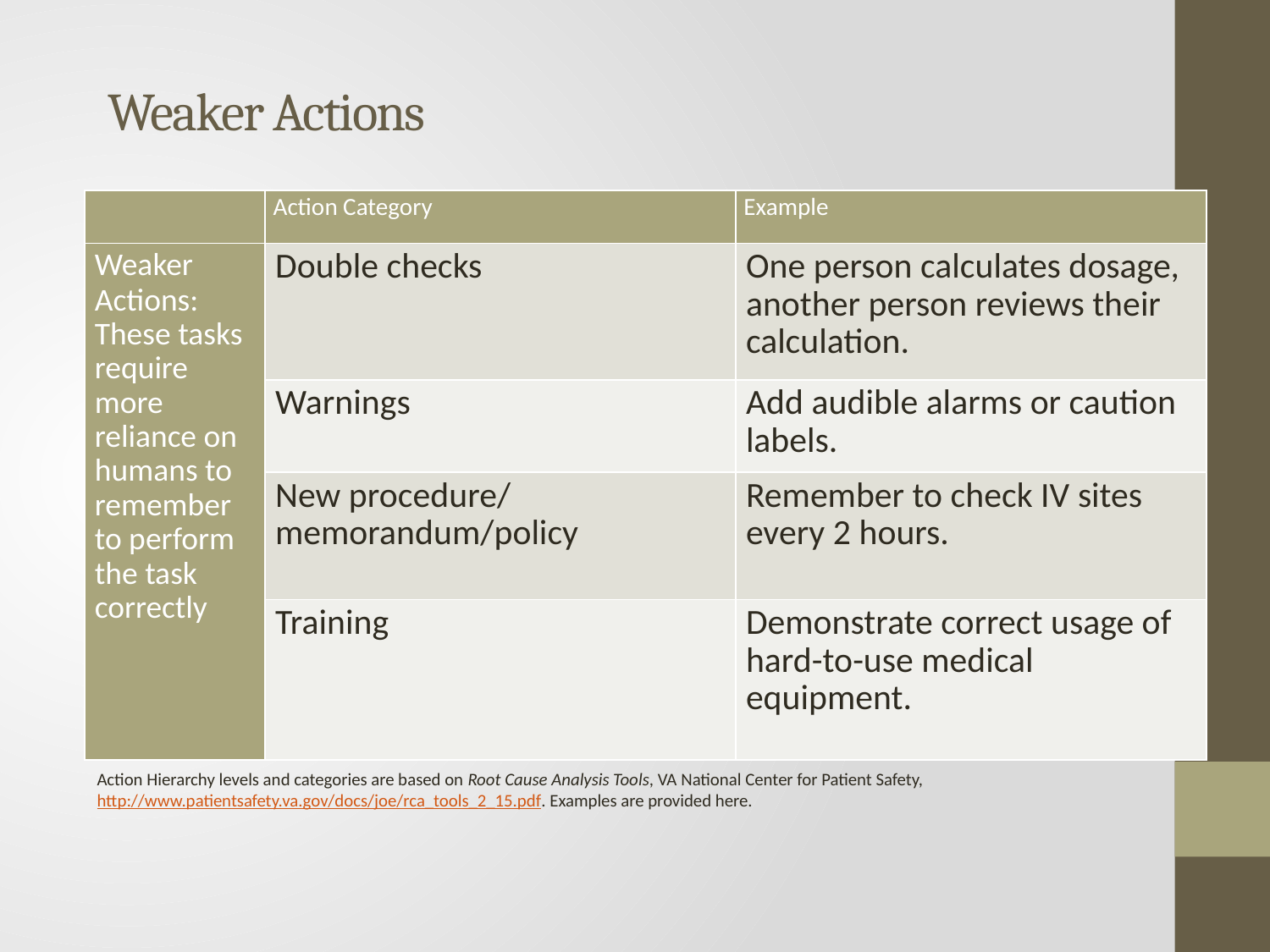

# Weaker Actions
| | Action Category | Example |
| --- | --- | --- |
| Weaker Actions: These tasks require more reliance on humans to remember to perform the task correctly | Double checks | One person calculates dosage, another person reviews their calculation. |
| | Warnings | Add audible alarms or caution labels. |
| | New procedure/ memorandum/policy | Remember to check IV sites every 2 hours. |
| | Training | Demonstrate correct usage of hard-to-use medical equipment. |
Action Hierarchy levels and categories are based on Root Cause Analysis Tools, VA National Center for Patient Safety, http://www.patientsafety.va.gov/docs/joe/rca_tools_2_15.pdf. Examples are provided here.

## Slide 21
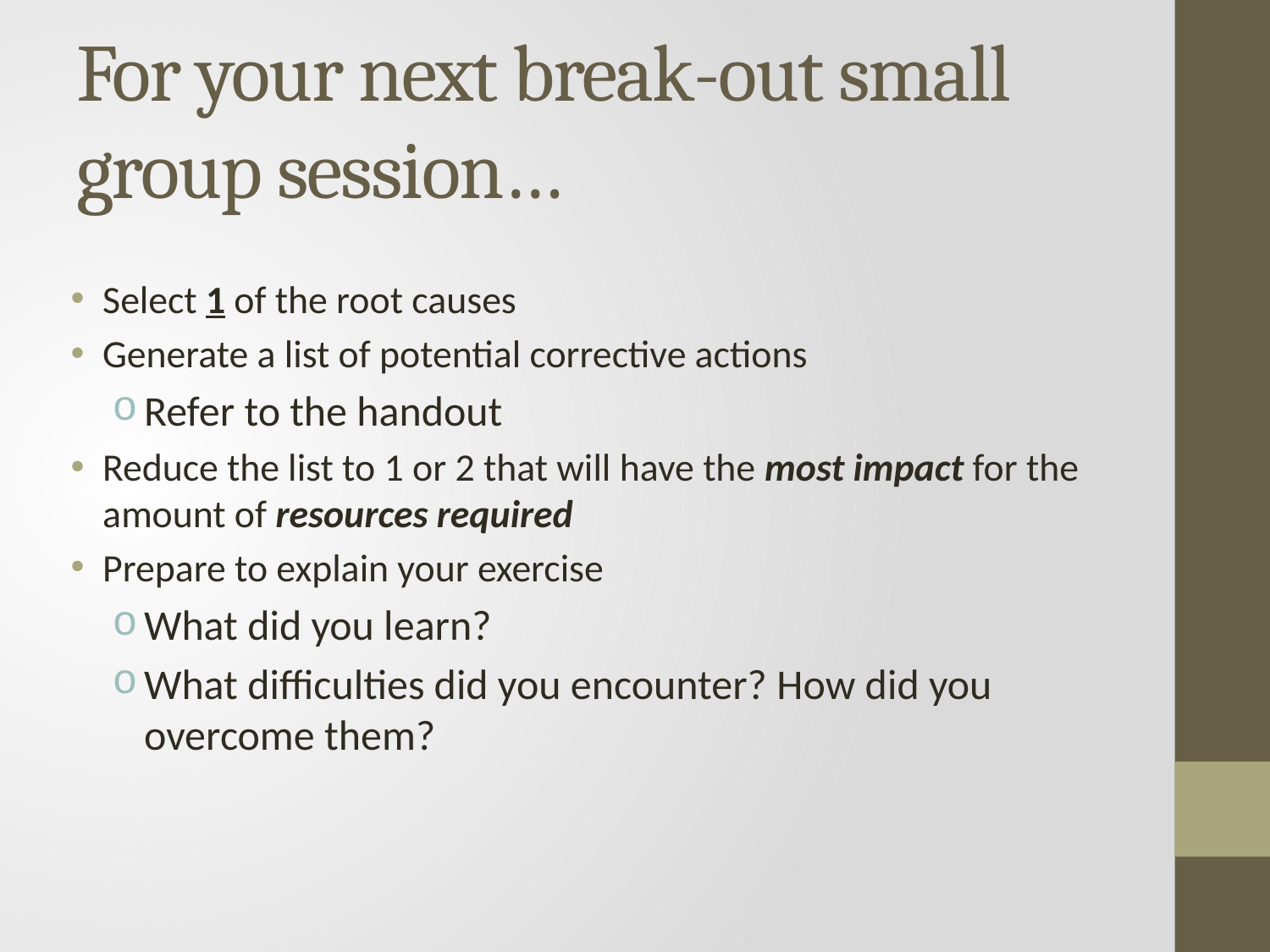

# For your next break-out small group session…
Select 1 of the root causes
Generate a list of potential corrective actions
Refer to the handout
Reduce the list to 1 or 2 that will have the most impact for the amount of resources required
Prepare to explain your exercise
What did you learn?
What difficulties did you encounter? How did you overcome them?

## Slide 22
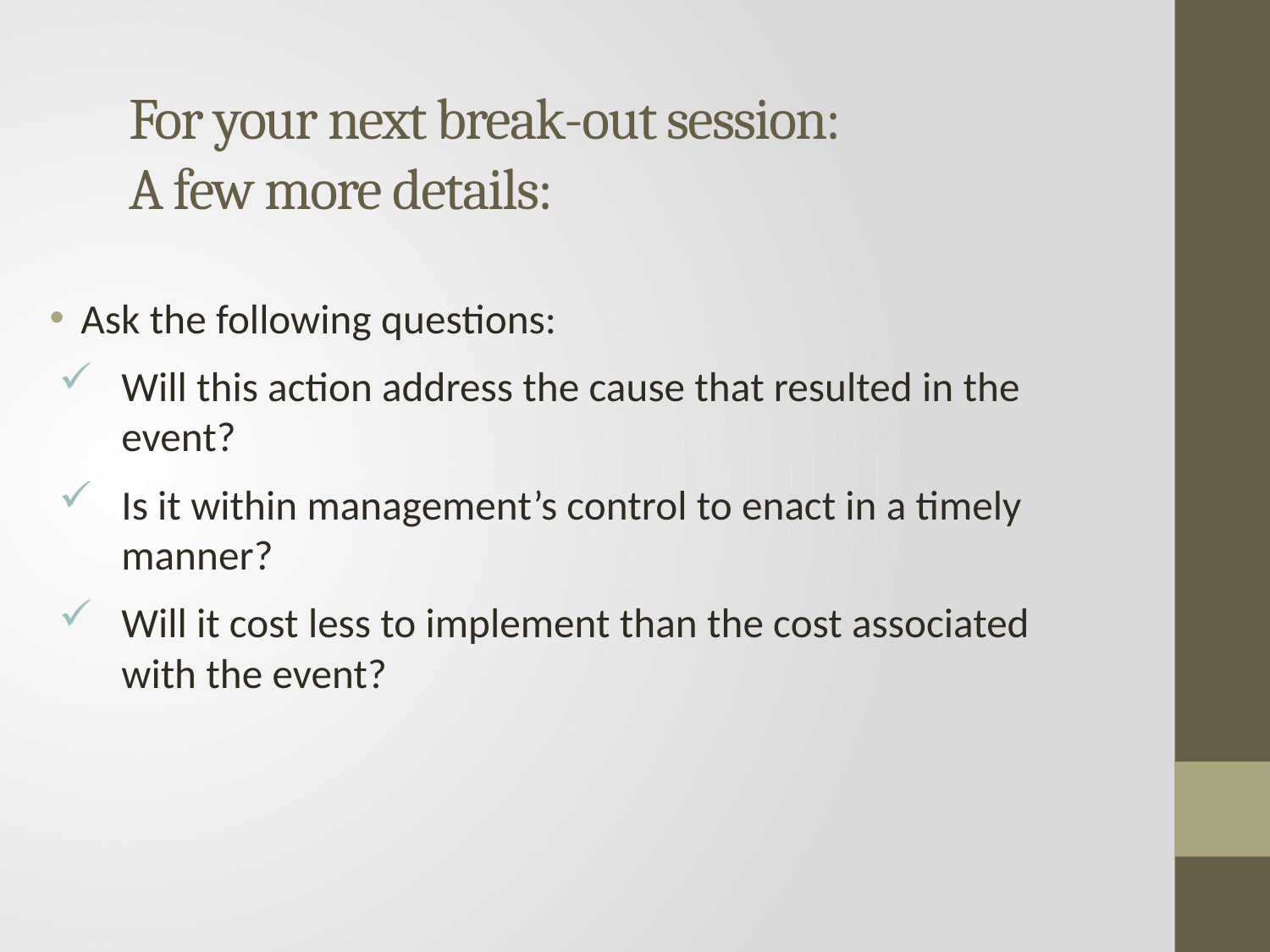

# For your next break-out session: A few more details:
Ask the following questions:
Will this action address the cause that resulted in the event?
Is it within management’s control to enact in a timely manner?
Will it cost less to implement than the cost associated with the event?

## Slide 23
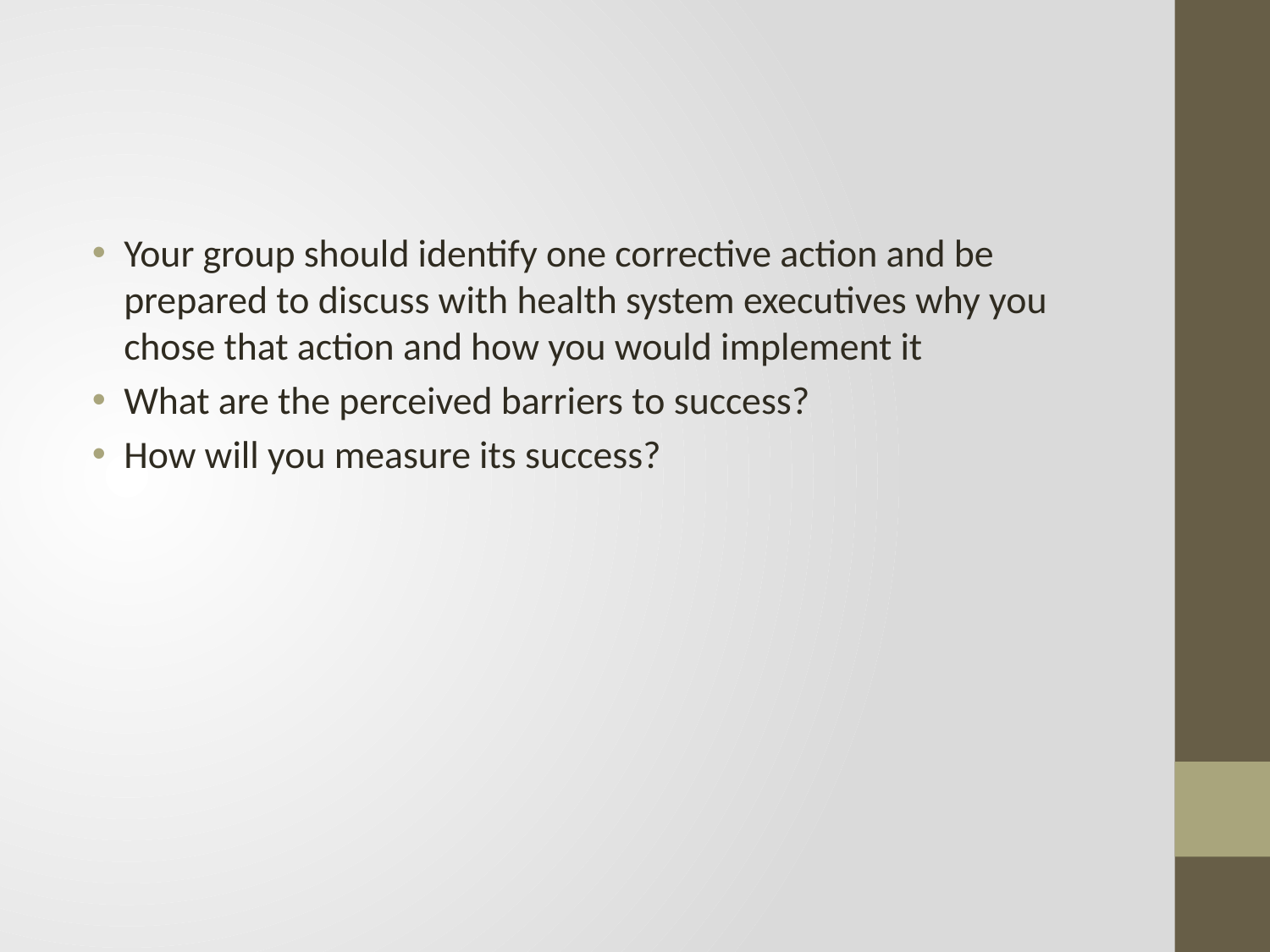

#
Your group should identify one corrective action and be prepared to discuss with health system executives why you chose that action and how you would implement it
What are the perceived barriers to success?
How will you measure its success?
