# Supplementary material for: Safety in Numbers: Successful Student-Approved Case-Based Interprofessional Safety Workshop Utilizing Simulated Real-Life Safety Cases
Source: MedEdPORTAL. 2020 Jan 31;16:10874. doi: 10.15766/mep_2374-8265.10874 (PMC7065299; doi:10.15766/mep_2374-8265.10874)
Supplement: Supplementary file 1 — A. Pre- & Postevent Surveys.docx B. IPE Safety Workshop Agenda.docx C. RCA AM Session Facilitator Guide.docx D. RCA AM Session Facilitator Annotated Case Time Line.docx E. RCA AM Session Student Case Time Line.docx F. RCA AM Session Interviewee Scripts.docx G. RCA AM Session Patient Background & EWS Info.docx H. RCA AM Session Media - Radiology.docx I. RCA AM Session Media - Oxygen Tanks.docx J. Corrective Action PM Session Facilitator Guide.docx K. Corrective Action PM Session Effectiveness Chart.docx L. Corrective Action PM Session Worksheet.docx M. Executive Case Summary.docx N. Large-Group Lecture Schedule & Topic List.docx O. PPT 1 - Contributing to a Culture of Safety.pptx P. PPT 2 - Systems Improvement.pptx Q. PPT 3 - Impact of Students and Residents on QI.pptx R. PPT 4 - Presentation of Safety Case.pptx S. PPT 5 - Disclosing Medical Errors.pptx T. PPT 6 - Training for Resilience.pptx U. PPT 7 - Introduction to Improvement Plans.pptx V. Facilitator Postworkshop Survey.docx [file mep-16-10874-s001.zip › V. Facilitator Postworkshop Survey.docx]

**Facilitator Post-Workshop Survey**

**1. How comfortable were you with patient safety and quality improvement concepts prior to the IPE Safety Workshop?**

**Uncomfortable/Lacked much knowledge in area**

**Somewhat comfortable/Some degree of knowledge**

**Comfortable/I had decent knowledge**

**Expert with these concepts**

**2. How comfortable were you with patient safety and quality improvement concepts after the IPE Safety Workshop?**

**Uncomfortable/Lacked much knowledge in area**

**Somewhat comfortable/Some degree of knowledge**

**Comfortable/I had decent knowledge**

**Expert with these concepts**

**3. How much did facilitating and participating in this workshop increase your knowledge of patient safety and quality improvement?**

**No increase in knowledge**

**Slight increase in knowledge**

**Moderate increase in knowledge**

**I learned a ton!**

**4. Do you feel your participation in this safety workshop event has made you more aware of patient safety and performance improvement at our medical center? w**

**No**

**Unsure**

**Yes**

**5. Based on this experience, would you be willing to participate in future interprofessional educational activities?**

**No, I didn't enjoy this**

**Maybe, unsure right now**

**Yes, I enjoyed the interprofessional educational experience**

**6. Did you think the presented patient safety case was effective in teaching safety and quality improvement principles?**

**No**

**Not sure**

**Yes**

**7. How engaged did you feel your small group students were in the day's activities?**

**Not engaged at all**

**Some were engaged, some were not**

**My students were moderately engaged**

**My students seemed very engaged**

**Question Title**

**8. Any other feedback or comments for the design team?**
